# Insights into the evolution and diversification of the *AT-hook Motif Nuclear Localized* gene family in land plants

**DOI:** 10.1186/s12870-014-0266-7

**Published:** 2014-10-14

**Authors:** Jianfei Zhao, David S Favero, Jiwen Qiu, Eric H Roalson, Michael M Neff

**Affiliations:** Molecular Plant Sciences Graduate Program, Washington State University, Pullman, WA 99164 USA; Department of Crop and Soil Sciences, Washington State University, Pullman, WA 99164 USA; School of Biological Sciences, Washington State University, Pullman, WA 99164 USA; Present Address: Department of Biology, University of Pennsylvania, Philadelphia, PA 19104 USA

**Keywords:** AT-hook motif, *AT-Hook Motif Nuclear Localized* (*AHL*) genes, Diversification, PPC domain, Phylogeny

## Abstract

**Background:**

Members of the ancient land-plant-specific transcription factor *AT-Hook Motif Nuclear Localized* (*AHL*) gene family regulate various biological processes. However, the relationships among the *AHL* genes, as well as their evolutionary history, still remain unexplored.

**Results:**

We analyzed over 500 *AHL* genes from 19 land plant species, ranging from the early diverging *Physcomitrella patens* and *Selaginella* to a variety of monocot and dicot flowering plants. We classified the AHL proteins into three types (Type-I/-II/-III) based on the number and composition of their functional domains, the AT-hook motif(s) and PPC domain. We further inferred their phylogenies via Bayesian inference analysis and predicted gene gain/loss events throughout their diversification. Our analyses suggested that the *AHL* gene family emerged in embryophytes and further evolved into two distinct clades, with Type-I *AHL*s forming one clade (Clade-A), and the other two types together diversifying in another (Clade-B). The two *AHL* clades likely diverged before the separation of *Physcomitrella patens* from the vascular plant lineage. In angiosperms, Clade-A *AHL*s expanded into 5 subfamilies; while, the ones in Clade-B expanded into 4 subfamilies. Examination of their expression patterns suggests that the *AHL*s within each clade share similar expression patterns with each other; however, *AHL*s in one monophyletic clade exhibit distinct expression patterns from the ones in the other clade. Over-expression of a *Glycine max* AHL PPC domain in *Arabidopsis thaliana* recapitulates the phenotype observed when over-expressing its *Arabidopsis thaliana* counterpart. This result suggests that the *AHL* genes from different land plant species may share conserved functions in regulating plant growth and development. Our study further suggests that such functional conservation may be due to conserved physical interactions among the PPC domains of AHL proteins.

**Conclusions:**

Our analyses reveal a possible evolutionary scenario for the *AHL* gene family in land plants, which will facilitate the design of new studies probing their biological functions. Manipulating the *AHL* genes has been suggested to have tremendous effects in agriculture through increased seedling establishment, enhanced plant biomass and improved plant immunity. The information gleaned from this study, in turn, has the potential to be utilized to further improve crop production.

**Electronic supplementary material:**

The online version of this article (doi:10.1186/s12870-014-0266-7) contains supplementary material, which is available to authorized users.

## Background

Genes that regulated essential biological processes in ancient plant species constituted a conserved “gene tool kit”, which tended to be preserved throughout evolution [1-4]. Most of the members in this “tool kit” have generally duplicated and expanded into multi-member-containing gene families with divergent functions in modern land plants [1,5,6]. Understanding their functions as well as evolutionary histories have greatly enhanced our knowledge of plant growth and development, such as the cases of the cytochrome P450s [7], MADS-box transcription factors [8-12], *AP2/EREBP* genes [13-16], the *TALE* homeobox gene family [17-19], NAC transcription factors [20-22], *HD-ZIP* genes [23-25], *Basic/Helix-Loop-Helix* genes [26-28] and the *TCP* gene family [29-31].

However, there are also many gene families that are important to land plant evolution whose functions and evolutionary histories are not well understood. The ancient transcription factor *AT-Hook Motif Nuclear Localized* (*AHL*) gene family has been found in all sequenced plant species, ranging from the moss *Physcomitrella patens*, to flowering plants, such as *Arabidopsis thaliana*, *Sorghum bicolor*, *Zea mays* and *Populus trichocarpa*. High conservation of this gene family throughout land plant evolution suggests that it is important for plant growth and development. Currently we are beginning to understand the biological functions of several *AHL*s. The evolutionary history of this gene family, however, has still barely been explored.

Members of the AHL proteins contain two conserved structural units, the AT-hook motif and the *P*lant and *P*rokaryote *C*onserved (PPC) domain, the latter being also annotated as the *D*omain of *U*nknown *F*unction #*296* (DUF296) [32]. Since the functions of this domain have been partially revealed [33], hereafter, we will refer it only as the PPC domain. The AT-hook motif enables binding to AT-rich DNA and has been identified in various gene families both in prokaryotes and eukaryotes, including the *H*igh *M*obility *G*roup *A* (HMGA) proteins in mammals [34]. The AT-hook motif uses a conserved palindromic core sequence, Arg-Gly-Arg, to bind to the minor groove of AT-rich B-form DNA. Upon binding with DNA, this core sequence adopts a concave conformation with close proximity to the backbone of the DNA, with both arginine side chains firmly inserting into the minor groove [35].

The second functional unit of the AHL proteins is the PPC domain, which is approximately 120 amino acids in length and exists as a single protein in Bacteria and Archaea [32]. Crystal structures of several bacterial and archaeal PPC proteins suggested that the prokaryotic PPC proteins form a trimer [36,37]. In land plants, the PPC domain has been identified in AHL proteins where it is located at the carboxyl end relative to the AT-hook motif(s) [32]. The PPC domain is responsible for the nuclear localization of the AHL proteins as well as protein-protein interactions among AHL proteins and with other common interactors, such as transcription factors. It may suggest a role in regulating transcriptional activation by the AHL proteins in plants [33].

Members of the *AHL* family regulate diverse aspects of growth and development in plants. Most of the studies are from the analyses of *Arabidopsis thaliana*. Several *AHL*s are suggested to regulate the homeostasis of phytohormones, especially gibberellins [38], jasmonic acid [39] and cytokinins [40]. Two members of the *Arabidopsis thaliana AHL* gene family, *SUPPRESSOR OF PHYTOCHROME B-4 #3* (*SOB3/AHL29*) and *ESCAROLA* (*ESC/AHL27*), repress hypocotyl elongation for seedlings grown in the light [41]. As adults, the *AtAHL* over-expression plants develop enlarged organs, such as expanded leaves, flowers and fruits as well as delayed flowering and senescence [41]. Similar functions have also been proposed for *AtAHL22*, and *HERCULES* (*HRC/AHL25*) [42,43]. *Arabidopsis thaliana ESC/AtAHL27* and *AHL20* have also been implicated in the regulation of plant defense responses [44,45].

In this study, we identified members of the *AHL* gene family in the completely sequenced genomes of 19 land plant species, ranging from the moss *Physcomitrella patens* and the lycophyte *Selaginella* to a variety of monocot and dicot species in the Phytozome database [46]. A closer look at their protein sequences revealed that these land plant AHL proteins can be divided into three types (Type-I, −II and -III) based on a combination of the number and composition of its two structural units, the AT-hook motif(s) and the PPC domain. The Type-I *AHL*s form one clade; while the Type-II and -III *AHL*s together form a separate clade. Phylogenetic analysis of the *AHL* genes in basal plants suggests that such divergence between the two clades dated between the appearance of chlorophytes and mosses. In this study, we have further identified that the *AHL* gene family in land plants evolved into 9 phylogenetic sub-families. Finally, we have proposed an evolutionary scenario for the *AHL* gene family in land plants.

## Results

### Early divergence in the land-plant AHL protein family

**Figure 1:**
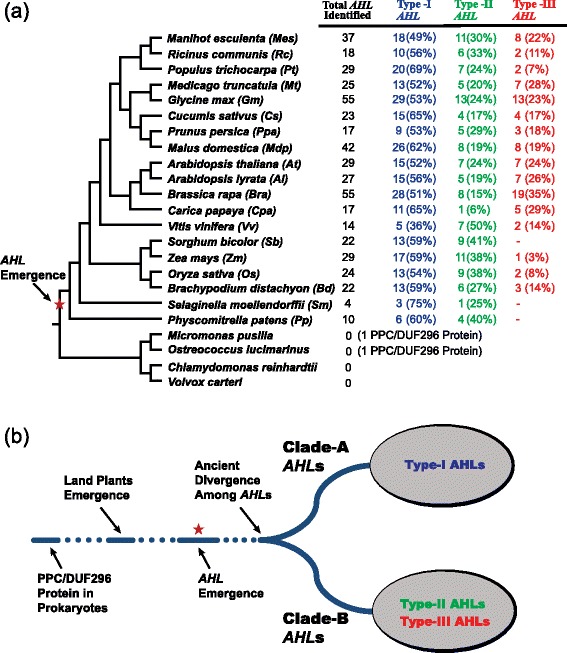
***AHL*** **genes identified in land plant species. (a)** The numbers of the *AHL* genes identified in each sequenced plant genome were listed accordingly. The percentages of each type were also listed in parenthesis. **(b)** *AHL* genes emerged in land plant species and further diverged into two separate monophyletic clades (Clade-A and Clade-B). The red star denoted the time point when the *AHL* genes are likely to have emerged.

Members of the *AHL* gene family contain two functional units, the AT-hook motif and the PPC domain [32]. In order to identify the *AHL* genes in land plant species, we performed searches against the Phytozome database using the *AHL* nucleotide and amino acid sequences from *Arabidopsis thaliana* [46]. We further added the retrieved results as additional queries to perform further searches to identify *AHL* genes from the genomes of 19 plant species (Figure 1a, Additional files 1, 2 and 3).

Initial phylogenetic analysis of the retrieved AHL proteins in this study suggested that all of the land-plant AHL proteins evolved into two major clades (Figure 1b). This distinct division into two monophyletic clades could also be observed in phylogenetic analysis when using just the *AHL* genes from *Arabidopsis thaliana* [32,33,38,41] and *Oryza sativa* [47]. Analysis of all the *AHL* genes identified in this study in the moss and lycophytes reveals a similar distribution into these two clades. This further suggests that the division between these two branches dated before the divergence of mosses from the rest of the land plants.

### Each monophyletic clade defines one type of PPC domain in land plant AHL proteins

Examination of the PPC domains revealed that their protein sequences share unique characteristics within each of the two AHL phylogenetic clades (Figure 1b, Additional file 4). The Clade-A AHL proteins share the same type of PPC domain (hereby named “Type-A PPC domain”). Clade-B AHL proteins share another type of PPC domain (hereby named “Type-B PPC domain”).

In order to further examine the divergence between the PPC domains in AHL proteins, we performed a sequence logo analysis. The Type-A PPC Domain in Clade-A generally starts with Leu-Arg-Ser-His (Additional file 4a); while the Type-B PPC domain in Clade-B generally starts with Phe-Thr-Pro-His (Additional file 4b). Both types of PPC domains in AHL proteins are further followed by stretches of amino acid residues with moderate conservation. Examination of both types of PPC domains in the identified AHL proteins revealed that they contain a consensus conserved Gly-Arg-Phe-Glu-Ile-Leu motif (Additional file 4a, b). It is also interesting to note that the coding sequences of this motif always exists at the immediate beginning of one exon region in the intron-containing Type-B PPC/DUC296 domains. The sequence upstream of the conserved six amino acids in Type-B PPC domains is generally Thr-Tyr-Glu, while it is generally Thr-Lys-His upstream of the six amino acids in Type-A PPC domains. The sequences downstream of the conserved six amino acids in both types of PPC domains are similar to each other.

### Conserved functions of PPC domains in AHL proteins in land plants

**Figure 2:**
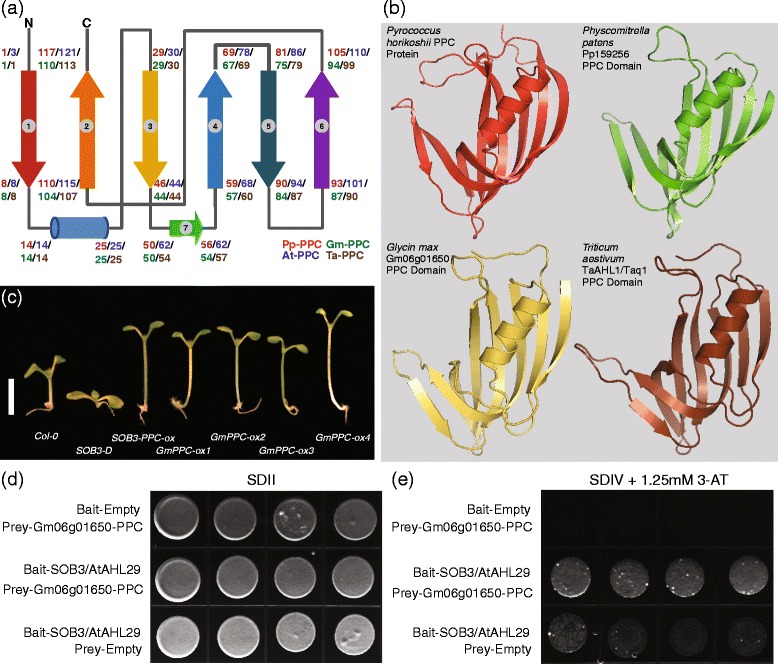
**The AHL proteins comprise AT-hook motif(s) and PPC domain. (a)** Topology of secondary structures of the AHL PPC domains from multiple land plant species. The cylinder denotes an α-helix and the arrows denote β-sheets. The numbers represent positions of the amino acids in the AHL PPC domain at the corresponding secondary structure positions. Pp-PPC, Pp159256 PPC domain. At-PPC, AtAHL29 PPC domain. Gm-PPC, Gm06g01650.1 PPC domain. Ta-PPC, TaAHL1 PPC domain. **(b)** Predicted tertiary structures of the PPC domains from these AHL proteins. **(c)** Hypocotyl growth of *Col-0*, *SOB3-D*, *SOB3-PPC* overexpression and multiple *Gm06g01650-PPC* overexpression lines, growing in 20 μmol∙s^−1^∙m^−2^ white light. Scale bar = 5 mm. **(d and e)** Full length Arabidopsis thaliana SOB3/AtAHL29 interacts with the PPC domain of *Glycine max* Gm06g01650.1 in an yeast two-hybrid assay.

In order to understand the biological functions of the PPC domains in the AHL proteins, we cloned two full-length *AHL* genes from the bread wheat *Triticum aestivum* and one PPC domain from a soybean *Glycine max AHL* gene (*Gm06g01650.1*) (Additional file 5). Although *Gm06g01650.1* is only a partial gene, it together with the cloned wheat *AHL*s and two *Arabidopsis thaliana AHL*s encode proteins that all contain a Type-I AT-hook motif and a Type-A PPC domain (Additional files 5 and 6). They share the same arrangement of secondary structural elements and tertiary structures with each other, as well as with their counterparts in prokaryotes and the moss, *Physcomitrella patens* (Figure 2a and 2b). A careful examination reveals that their PPC domains all exhibit a *β*_1_-*α*-*β*_3_-*β*_7_-*β*_4_-*β*_5_-*β*_6_-*β*_2_ secondary structural arrangement, suggesting possible conserved biological functions of this domain among multiple species.

To test the hypothesis that the PPC domain may share conserved biological regulatory functions, we overexpressed this domain from *Gm06g01650.1* driven by the *35S* constitutive promoter in wild-type *Arabidopsis thaliana*. Multiple homozygous over-expression lines containing single-locus insertions exhibited longer hypocotyls in white light comparing with wild-type controls (Figure 2c). This long-hypocotyl phenotype is similar to the one demonstrated by seedlings over-expressing the PPC domain from *Arabidopsis thaliana* AtAHL29/SOB3 [33], suggesting that shared conserved biological functions exist between *Glycine max* and *Arabidopsis thaliana AHL*s.

*Arabidopsis thaliana AHL*s have been suggested to suppress hypocotyl growth in the light [33,41]. Therefore, the long-hypocotyl phenotype exhibited by over-expressing the *Gm06g01650.1* PPC domain may be conferred through the disturbance of the growth suppression roles of *Arabidopsis thaliana AHL* genes. To test this hypothesis, we examined if the PPC domain of Gm06g01650.1 can physically interact with the *Arabidopsis thaliana* AHL proteins using a targeted *lexA*-based yeast two-hybrid assay (Figure 2d,e). Using 1.25 mM 3-amino-1, 2, 4-triazol that prevented transcriptional auto-activation by SOB3/AtAHL29 in the bait protein, we demonstrated that SOB3/AtAHL29 from *Arabidopsis thaliana* and the PPC domain of *Glycine max* Gm06g01650.1 can interact with each other (Figure 2d,e).

### Type-I and -II AT-hook motifs exist in AHL proteins

**Figure 3:**
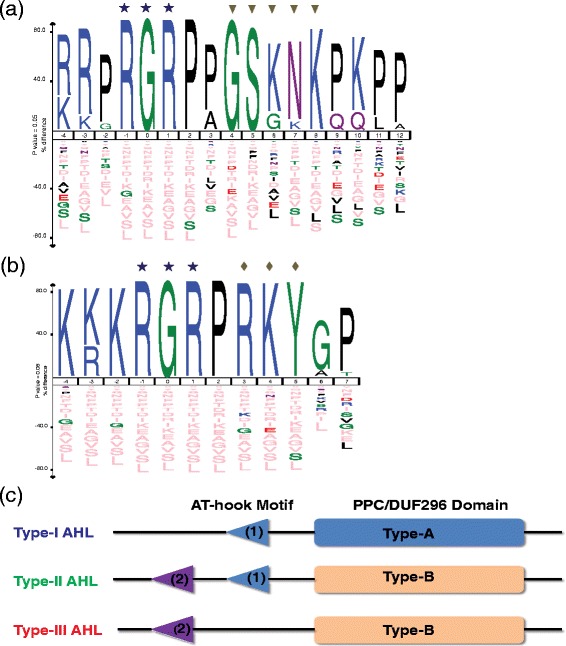
**Type of AHL proteins and their AT-hook motifs in land plants.** Ice-Logo analysis of the Type-I AT-hook motifs **(a)** and Type-II AT-hook motifs **(b)** in land-plant AHL proteins. The star symbol denotes the core sequence of the AT-hook motif. The conserved sequence downstream of the core sequences in Type-I and Type-II AT-hook motifs were pointed out by the triangle and diamond symbols accordingly. **(c)** Topology of three types of AHL proteins identified in land plants based on the combination of AT-hook motifs and PPC domain.

Two types of AT-hook motifs (Type-I and -II) are found in the AHL proteins (Figure 3a,b; Additional file 7) [33,34]. Both types of AT-hook motifs in the AHL proteins share the same conserved Arg-Gly-Arg core and use this conserved palindromic core to bind the minor groove of AT-rich B-form DNA [35]. Clade-A AHLs contain only one copy of the Type-I AT-hook motifs; while, in Clade-B, some of the AHLs contain only one copy of the Type-II AT-hook motifs and the rest contain both types of AT-hook motifs.

A specific consensus sequence, Gly-Ser-Lys-Asn-Lys, was observed at the carboxyl end of the Arg-Gly-Arg core sequence in the Type-I AT-hook motifs (Figure 3a, Additional file 7a,b). The conservation of these downstream sequences is more significant in the AHLs that only contain this type of AT-hook motif. However, these sequences are more variable in other AHLs that also possess a Type-II AT-hook motif (Additional file 7b). Only short consensus amino acid stretches, Arg-Lys-Tyr, could be observed downstream of the conserved Arg-Gly-Arg core sequences of the Type-II AT-hook motifs in clades of both AHLs (Figure 3b, Additional file 7c,d). The conservation of these downstream sequences is similar among the AHLs in either clade (Additional file 7c,d).

### Three types of AHL proteins in land plants

Based on a combination of type and number of the AT-hook motif(s) and the PPC domain, all the AHL proteins identified in this study can be further classified into three types (Type-I, −II and -III AHLs) (Figure 3c). The Type-I AHL proteins contain one Type-I AT-hook motif and one Type-A PPC domain. The Type-II AHL proteins contain two AT-hook motifs (one additional Type-II AT-hook motif at the N-terminus of the Type-I AT-hook motif) and one Type-B PPC domain. Finally, the Type-III AHL proteins contain one Type-II AT-hook motif and one Type-B PPC domain. Clade-A is comprised of the Type-I *AHL* genes, while Clade-B is comprised of the Type-II and -III *AHL* genes. Both clades have *AHL* genes from *Physcomitrella patens* (moss) forming a sister clade to the rest of the members of the clade, indicating an early divergence between the Type-I *AHL*s and the other two types of *AHL* genes.

### Type-I and -II *AHL*s found in flowering plants were present in early-diverged land plants

In order to understand the evolutionary origin of the *AHL* genes, we also performed searches for *AHL* genes in chlorophytes. Neither any *AHL* genes nor genes encoding the PPC domain could be identified in the current release of the *Chlamydomonas reinhardtii* and *Volvox carteri* genomes (Figure 1a) [46,48,49]. Surprisingly, we were able to identify only one *PPC* gene that encodes only the PPC domain without an associated AT-hook motif(s) in *Micromonas pusilla CCMP1545* [50] and *Ostreococcus lucimarinus* [51] (Additional file 8). To further examine the presence of the *PPC* gene in picoeukaryotic species, we further examined the genome of an additional picoeukaryotic strain *Ostreococcus tauri* [52]. Similarly, only a single copy of the *PPC* gene could be identified (Additional file 8). This is similar to the case observed in bacterial and archaeal genomes, where each species contains only one *PPC* gene which encodes a single protein (Additional file 8) [32].

We further examined the genomic sequences of the *AHL* genes and found that the Type-II and -III *AHL* genes generally contain introns, while the Type-I *AHL* genes lack introns in their genomic sequences. This suggests that it is likely that the intron-less Type-I *AHL* genes in land plants is the ancestral form from which the two intron-containing types are derived. In each species, there are generally more Type-I *AHL* genes in number than either of the other two types (Figure 1a). Compared to other families, the Poaceae species have a lower percentage of Type-III *AHL* genes, including *Zea mays* [53], *Oryza sativa* [54,55] and *Brachypodium distachyon* [56]. Notably, in *Sorghum bicolor* [57] we could not detect any Type-III *AHL*s (Figure 1a). It is likely that the Type-III *AHL*s arose latest since the moss *Physcomitrella patens* and lycophyte *Selaginella moellendorffii* contain only Type-I and -II *AHL*s (Figure 1a).

Plant introns have been suggested to play important roles in regulating the expression of their associated genes through alternative splicing [58-60], nonsense-mediated mRNA decay [61], or intron-mediated transcriptional enhancement [62]. In order to understand the biological functions of the introns in Type-II and -III *AHL*s, we extracted the intron sequences from *Arabidopsis thaliana AHL*s and examined their capabilities to enhance the transcription of their associated genes using the IMEter 2.0 server [63]. The first introns of several *AtAHL*s demonstrated at least a moderate ability to enhance the transcription of their genes (Additional file 9a-c). Particularly, the first introns in *AtAHL4*, *6* and *14* are predicted to strongly enhance their transcription.

### Monophyletic Clade-A contains type-I *AHL*s

**Figure 4:**
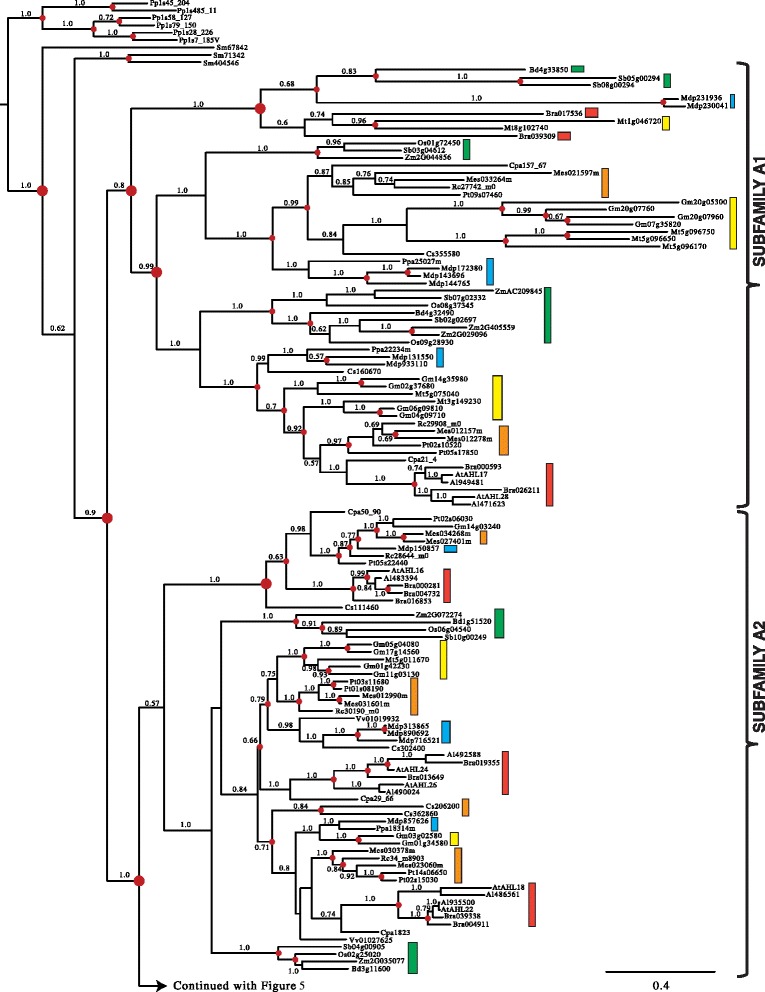
**Phylogeny of the Clade-A** ***AHL*** **gene family in land plants using Bayesian analysis.** Clade-A *AHL*s are separated into 5 subfamilies (A1, A2, A3, A4 and A5). Two *AHL* genes (*TaAHL1* and *TaAHL3*) were cloned from *Triticum aestivum* and shown in red. Green boxes represent *AHL* genes from Poaceae, yellow boxes denote genes from Fabaceae, blue boxes denote genes from Rosaceae, orange boxes denote genes from Malpighiales, and red boxes denote genes from Brassicaceae. Numbers near the branches indicate the Bayesian posterior probabilities for given clades. The red dots at internal nodes denote where gene duplication events have occurred.

**Figure 5:**
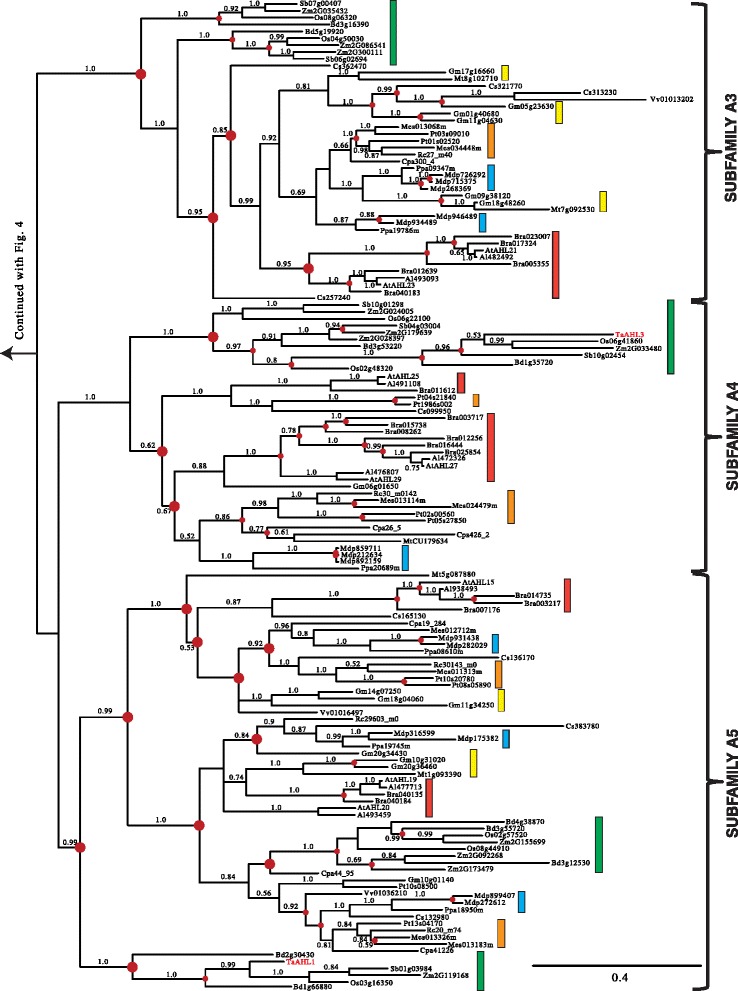
**Phylogeny of the Clade-A** ***AHL*** **gene family in land plants using Bayesian analysis.** Clade-A *AHL*s are separated into 5 subfamilies (A1, A2, A3, A4 and A5). Two *AHL* genes (*TaAHL1* and *TaAHL3*) were cloned from *Triticum aestivum* and shown in red. Green boxes represent *AHL* genes from Poaceae, yellow boxes denote genes from Fabaceae, blue boxes denote genes from Rosaceae, orange boxes denote genes from Malpighiales, and red boxes denote genes from Brassicaceae. Numbers near the branches indicate the Bayesian posterior probabilities for given clades. The red dots at internal nodes denote where gene duplication events have occurred.

The early divergence between and significant divergence within the two *AHL* clades made analyzing them separately necessary to obtain reliable amino acid alignments. We first performed Bayesian inference analysis on the retrieved Clade-A *AHL*s. The Clade-A *AHL*s in land plants is comprised of Type-I *AHL*s that we have organized for discussion convenience into five subfamilies (Subfamilies A1, A2, A3, A4 and A5) (Figures 4 and 5).

In order to better understand the evolutionary events which occurred among these five subfamilies, we reconciled the obtained Bayesian tree with the land-plant species tree and inferred whether the internal nodes within the Clade-A Bayesian tree were associated with gene duplication, gene loss, or lineage divergence events. Since their emergence in land plants, the *AHL*s within this clade have undergone multiple gene duplication events in the early plant lineages. The Subfamily A1-A5 *AHL*s emerged from lineage divergence events after the divergence of lycophyte *AHL*s and from the rest of vascular plants and further expanded via a series of gene-duplication/divergence events in angiosperms. The emergence of Subfamily A1, A3 and A5 *AHL*s started via gene-duplication events; while, Subfamily A2 and A4 *AHL*s emerged via speciation events.

Within each subfamily of Clade A, *AHL* genes from Euphorbiaceae, Salicaceae, Fabaceae, Rosaceae, Brassicaceae and Poaceae families could all be observed, suggesting they may have evolved from one subfamily-specific most common ancestral gene and later functional divergence occurred among these subfamilies. In the extant plant species, the *AHL* genes have undergone extensive gene-duplication/loss events (Table 1). The gene duplication events in several extant plant species, such as *Glycine max* [64] and *Malus domestica* [65], are probably associated with their recent whole genome duplication events. On the contrary, in several other plant species including *Ricinus communis*, *Carica papaya*, *Vitis vinifera* and monocot species, the *AHL* gene phylogenies show drastic gene loss events.

**Table 1:** **Numbers of gene duplication and loss event of the** ***AHL*** **genes in extant land plant species**

**Extant land plant species**	**Clade-A** ***AHL*** **s (Type-I)**		**Clade-B** ***AHL*** **s (Types-II/-III)**	
	**No. of gene duplication**	**No. of gene loss**	**No. of gene duplication**	**No. of gene loss**
*Manihot esculenta* (*Mes*)	5	4	4	3
*Ricinus communis* (*Rc*)	0	7	0	10
*Populus trichocarpa* (*Pt*)	6	7	2	9
*Medicago truncatula* (*Mt*)	3	10	2	5
*Glycine max* (*Gm*)	12	3	13	2
*Cucumis sativus* (*Cs*)	1	8	0	7
*Prunus persica* (*Ppa*)	0	3	0	3
*Malus domestica* (*Mdp*)	13	0	7	2
*Arabidopsis thaliana* (*At*)	0	3	1	1
*Arabidopsis lyrata* (*Al*)	0	3	0	2
*Brassica rapa* (*Bra*)	3	7	4	2
*Carica papaya* (*Cpa*)	0	8	0	6
*Vitis vinifera* (*Vv*)	0	20	0	16
*Sorghum bicolor* (*Sb*)	1	9	0	3
*Zea mays* (*Zm*)	1	5	2	2
*Oryza sativa* (*Os*)	0	12	0	5
*Brachypodium distachyon* (*Bd*)	0	12	1	8
*Selaginella moellendorffii* (*Sm*)	1	0	0	1
*Physcomitrella patens* (*Pp*)	5	0	3	1

### Monophyletic Clade-B contains type-II and -III *AHL*s

**Figure 6:**
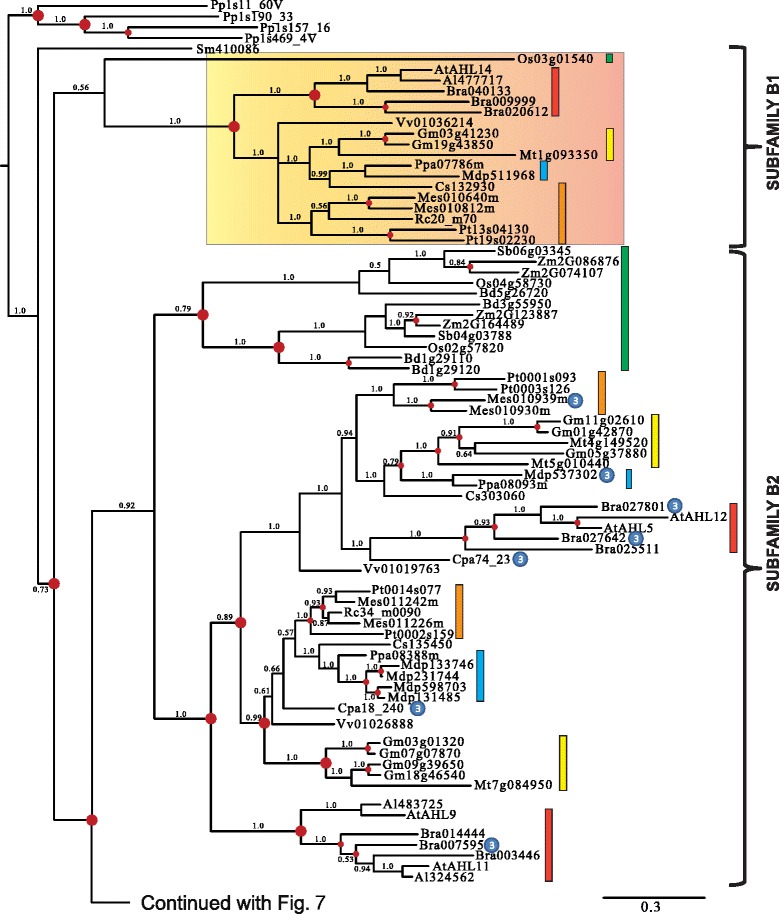
**Phylogeny of the Clade-B** ***AHL*** **gene family in land plants using Bayesian analysis.** Clade-B *AHL*s could be distinguished into 4 subfamilies (B1, B2, B3 and B4). Type-III *AHL*s that form sub-clades have been highlighted by gradient boxes (in Subfamilies B1 and B4) or pointed out by ③ if within Type-II *AHL* sub-clades. Type-II *AHL*s are pointed out by ② if within a Type-III AHL sub-clades (within Subfamily B4). Green boxes represent *AHL* genes from Poaceae, yellow boxes denote genes from Fabaceae, blue boxes denote genes from Rosaceae, orange boxes denote genes from Malpighiales, and red boxes denote genes from Brassicaceae. Numbers near branches indicate the Bayesian posterior probabilities for given clades. The red dots at internal nodes denote where gene duplication events have occurred.

**Figure 7:**
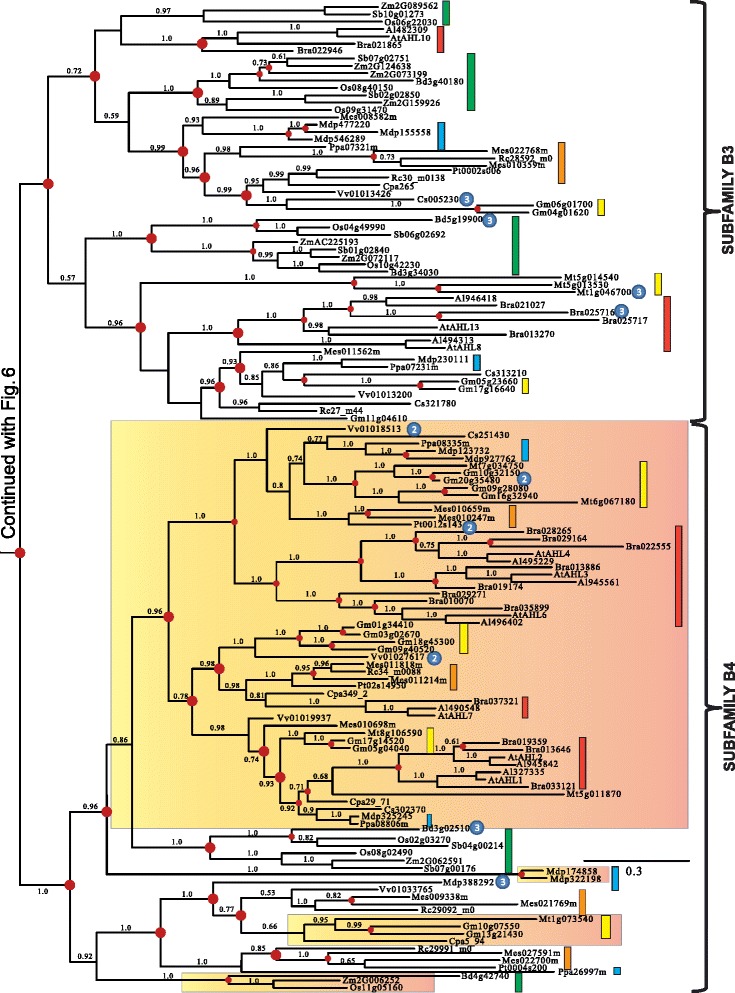
**Phylogeny of the Clade-B** ***AHL*** **gene family in land plants using Bayesian analysis.** Clade-B *AHL*s could be distinguished into 4 subfamilies (B1, B2, B3 and B4). Type-III *AHL*s that form sub-clades have been highlighted by gradient boxes (in Subfamilies B1 and B4) or pointed out by ③ if within Type-II *AHL* sub-clades. Type-II *AHL*s are pointed out by ② if within a Type-III AHL sub-clades (within Subfamily B4). Green boxes represent *AHL* genes from Poaceae, yellow boxes denote genes from Fabaceae, blue boxes denote genes from Rosaceae, orange boxes denote genes from Malpighiales, and red boxes denote genes from Brassicaceae. Numbers near branches indicate the Bayesian posterior probabilities for given clades. The red dots at internal nodes denote where gene duplication events have occurred.

Clade-B of the *AHL* gene family is comprised of Type-II and Type-III *AHL*s (Figures 6 and 7). The Type-II *AHL*s from the early diverging moss *Physcomitrella patens* and lycophyte *Selaginella moellendorffii* constitute a clade at the base of the phylogenetic tree (Figure 6). The angiosperm portion of Clade-B can be divided into four subfamilies (Subfamilies B1, B2, B3 and B4).

In Subfamilies B1 and B4, members of the Type-III *AHL*s tend to group together and form Type-III *AHL* sub-clades (highlighted with gradient shaded box). Individual members of Type-II *AHL*s can be observed within the Subfamily B4 Type-III AHL sub-clades. This indicates possible regaining of the Type-I AT-hook motif within this subfamily, suggesting that not all Type-I AT-hooks are homologous. Individual Type-III *AHL*s also exist within the Type-II *AHL* sub-clades (such as Subfamilies B2, B3 and B4). This suggests an independent loss of the Type-I AT-hook motifs by AHL proteins within these subfamilies. Taken together, this indicates there are close evolutionary relationships between these two types of *AHL*s with, apparently, multiple transitions from Type-II to Type-III *AHL*s, and from Type-III to Type-II *AHL*s. The genomes of the moss *Physcomitrella patens* and lycophyte *Selaginella moellendorffii* do not contain Type-III *AHL*s, suggesting that the loss of the Type-I AT-hook motif in Clade-B occurred after lycophytes diverged from the rest of vascular plants (Figures 1a and 6).

Similar to their counterparts in Clade A, the Clade B *AHL*s also experienced multiple gene duplication and loss events during angiosperm diversification (Figures 6 and 7). Subfamily B1-B4 *AHL*s emerged from lineage divergence events and further expanded via multiple gene duplication/loss/divergence events (Table 1). In each extant plant species, Clade-B *AHL*s experienced similar numbers of gene duplication/loss events as their counterparts in Clade-A, suggesting shared evolutionary pressure between the two clades.

### Members of each *AHL* monophyletic clade share similar expression patterns

To test the hypothesis that Clade-A and -B *AHL*s evolved independently, we examined the expression patterns of the *AHL*s in *Arabidopsis thaliana* using Genevestigator V3 [66]. Based on their expression patterns across various tissues at different developmental stages, the 29 *Arabidopsis thaliana AHL*s can be clearly distinguished into two groups (Additional file 10). A careful examination reveals that the Type-II and -III *AtAHL*s tend to share similar expression patterns. Type-II and -III *AtAHL*s, which constitute the Clade-B *AHL*s, are primarily expressed during seed and flower development. They are only moderately expressed in other tissues. On the other hand, Type-I *AtAHL*s, which constitute the Clade-A *AHL*s, are primarily expressed during vascular tissue and root development, which are distinctly different from the expression patterns observed for Type-II and -III *AHL*s. Such distinct expression patterns between the two clades of *AHL*s can also be observed in *Zea mays* (Additional file 11).

## Discussion

The *AHL* gene family was first described about 10 years ago, as a group of plant-specific genes encoding proteins containing one or two copies of the AT-hook motif and a 120-amino-acid PPC domain [32]. In this study, AHL proteins have been identified in various plant species, including the early diverging mosses and lycophytes, as well as several angiosperm families [46]. We have further classified the AHL proteins into three types based on the number and composition of these two domains. Accordingly, both the AT-hook motifs and PPC domains of the AHL proteins can be classified into two types based on the phylogenetic analysis performed in this study.

### From the prokaryotic PPC proteins to the AHL proteins in land plants

The PPC domain found in the AHL proteins exists by itself as a single protein in prokaryotes [32]. Individual strains of Bacteria and Archaea contain one gene encoding a PPC protein (Additional file 8). This observation suggests a role for the PPC domain in fundamental biological processes that has been conserved since prokaryotes throughout evolution. It is intriguing to note that even in the eukaryotic photosynthetic phytoplankton, such as *Micromonas pusila* [50] and *Ostreococcus lucimarinus* [51], the PPC protein still exists as a single gene. This observation indicates that the association with an AT-hook motif is not necessary for the functions of the PPC protein/domain in prokaryotes and early eukaryotes.

The appearance of the AHL proteins may have occurred between the emergence of the embryophytes and tracheophytes (pointed out by the red star in Figure 1a). The primitive AHL proteins emerged when the AT-hook motif fused with the PPC protein between the divergence of picoeukaryotes and the moss *Physcomitrella patens*. These primitive proteins later diversified and evolved into two monophyletic clades that comprise the three types of modern AHL proteins found in land plants. However, the evolutionary history of the expansion and later diversifications of these *AHL* genes are yet unexplored.

### Ancient events on the AHL evolutionary timeline in land plants

**Figure 8:**
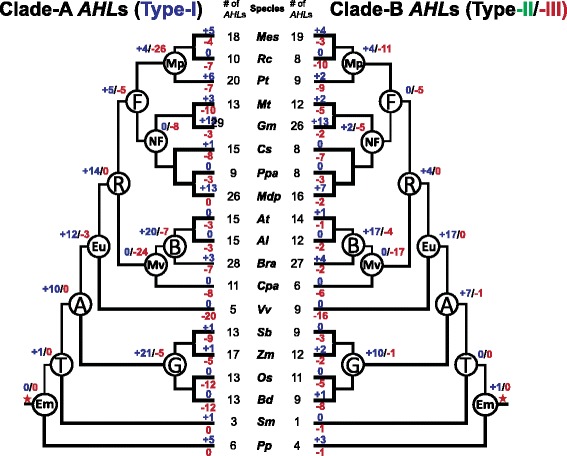
**Evolutionary events of the** ***AHL*** **gene family in land plants.** Numbers of gene duplication (shown in blue after “+”) and loss (shown in red after “-”) events were inferred for each internal node as well as for current extant species. The numbers of the *AHL* genes were also listed accordingly. The red star denotes when the *AHL* genes emerged. *Al*, *Arabidopsis lyrata. At*, *Arabidopsis thaliana. Bd*, *Brachypodium distachyon. Bra*, *Brassica rapa. Cpa*, *Carica papaya. Cs*, *Cucumis sativus. Gm*, *Glycine max. Mdp*, *Malus domestica. Mes*, *Manihot esculenta. Mt*, *Medicago truncatula. Os*, *Oryza sativa. Pp*, *Physcomitrella patens. Ppa*, *Prunus persica. Pt*, *Populus trichocarpa. Rc*, *Ricinus communis. Sb*, *Sorghum bicolor. Sm*, *Selaginella moellendorffii. Vv*, *Vitis vinifera. Zm*, *Zea mays*. A, Angiosperms. B, Brassicaceae. Em, Embryophyta. Eu, Eudicots. F, Fabidae (Eurosids I). G, Grasses. Mp, Malpighiales. Mv, Malvidae (Eurosids II). NF, Nitrogen fixing. T, Tracheophyta (vascular plants).

In order to better understand the expansion of the land-plant-specific *AHL* genes, we hypothesized the evolutionary events (duplications and deletions) that occurred at common ancestors across land plants (Figure 8). In the embryophytes and tracheophytes, there were few gene duplication/loss events occurring after the emergence of *AHL* genes in both *AHL* clades. However, both Clade-A and -B *AHL*s later experienced rapid expansion in angiosperms, which may be responsible for their large numbers in extant angiosperm species. During the emergence of the grass lineage, Clade-A *AHL*s exhibited more gene duplications than those in Clade-B. However, during the emergence of eudicots, Clade-B *AHL*s duplicated more rapidly. *AHL*s in Clade-B expanded in eudicots mainly through numerous gene duplication events; while those in Clade-A were also coupled with a few gene loss events. With the emergence of rosids, Clade-A *AHL*s duplicated more than their counterparts in Clade-B. Both clades later experienced dramatic gene losses during the emergence of Malvidae (Eurosids II).

The most dramatic difference between Clade-A and -B *AHL*s appears within the emergence of Fabidae (Eurosids I). Clade-A *AHL*s showed rapid birth-and-death events; while the Clade-B copies experienced only gene loss events. This is in direct contrast to the *AHL* genes in the emergence of nitrogen fixing species. Clade-A *AHL*s endured rapid gene losses; while Clade-B copies experienced birth-and-death events. In Malpighiales and Brassicaceae, both clades also emerged through gene birth-and-death events.

### A model for the evolutionary history of the *AHL* gene family in land plants

**Figure 9:**
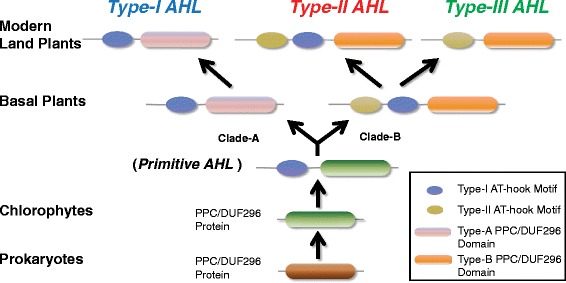
**Evolution scenario of the** ***AHL*** **gene family in land plants.** In prokaryotes and picoeukaryotes, the PPC domain exists by itself as a PPC protein. The *AHL* gene family emerged in Embryophytes by incorporating a Type-I AT-hook motif at the N-terminus of the PPC domain, forming primitive Type-I AHL protein(s). The *AHL* genes were further duplicated and gradually evolved into two clades (Clade-B with newly emerged Type-II *AHL*s by incorporating one Type-II AT-hook and Clade-A with Type-I *AHL*s). Along the evolutionary division into the two clades, the PPC domains in the *AHL* gene family were also evolved into two types. Through the evolution of modern land plants, members of the Type-II *AHL*s lost the Type-I AT-hook motif and gradually evolved into the Type-III *AHL*s.

Based on the results in this study, we propose a model to describe the evolutionary history of the *AHL* gene family in land plants (Figure 9). In this model, the *PPC* gene existed by itself and encoded a PPC protein in prokaryotes as well as in early Viridiplantae. Prior to the divergence of extant embryophytes, the PPC domain became associated with a Type-I AT-hook motif to form a primitive intron-less *AHL* gene. Another Type-II AT-hook motif was further acquired by this type of primitive *AHL* before the divergence of mosses from the rest of land plants to form a second type of *AHL* gene. This new type of *AHL* further acquired introns in their genomic sequences. The emergence of both types of *AHL*s occurred somewhere between the divergence of picoeukaryotes and mosses. These two types of primitive *AHL*s duplicated, differentiated and further developed independently into members of Type-I and -II *AHL*s in early land plants, defining the two clades (Clade-A and -B). This model is supported by the observation that only these two types of *AHL*s could be found in mosses and lycophytes (Figure 1). Members of the intron-containing Type-II *AHL*s further diversified, some losing the Type-I AT-hook motif while retaining the type-II AT-hook motif, forming the intron-containing Type-III *AHL*s. While we have a general idea of when these events occurred, more detailed sampling among green algae, particularly the streptophyte algae, and more land plant lineages (liverworts, hornworts, ferns, gymnosperms, and monocots other than grasses) is needed to fully resolve the timing of gains and losses of the AT-hook motifs and duplication/deletion events.

### AHL genes belong to the conserved “Gene Tool Kit” in plant evolution

Since they originated and diversified early in land plant evolution, the *AHL* genes also belong to the conserved “gene tool kit” of ancient plants. Throughout the evolution of land plants, the *AHL*s co-evolved with other “tool kit” members to regulate essential biological processes. The proposed co-evolution is supported by the observed genetic interactions with other ancient plant gene families, such as the NAC transcription factors and the MADS-box genes [33,67]. This hypothesis is also supported by the observed physical interactions of AHL proteins with histones (H2B, H3 and H4), TCPs (TCP4, TCP13, and TCP14), ATAF2 and DELLA proteins [33,68].

The observed physical interactions of the AHL proteins with other non-AHL transcription factors as well as with themselves led to a recently proposed “enhanceosome” molecular model [33]. In this model, it is proposed that the AHL proteins interact with each other to form homo-/hetero-trimer complexes via their PPC domains [33,69]. A conserved 6-amino-acid motif in the PPC domain from each monomer AHL protein acts together with those from the other two monomers to compose a quaternary domain. This domain in turn may mediate physical interactions with other transcription factors. In this study, over-expression of one *Glycine max* AHL PPC domain recapitulated the long-hypocotyl phenotype reminiscent of over-expressing its *Arabidopsis thaliana* counterpart (Figure 2). This indicates that the AHL PPC domains may serve evolutionarily conserved roles in regulating biological processes in multiple plant species. The AHL proteins share similar secondary and tertiary structures (Figure 2). In particular, the 6-amino-acid motif, Gly-Arg-Phe-Glu-Ile-Leu, is highly conserved in the PPC domains of AHLs from all land plants (Additional file 4). Therefore, it is possible that the functional conservation of the AHL proteins is achieved through the preservation of interacting partners among different plant species. In this study, we showed that *Arabidopsis thaliana* SOB3/AtAHL29 can physically interact with the *Glycine max* Gm06g01650.1 PPC domain (Figure 2d,e). This observation supports the hypothesis that the AHL proteins from different species can interact with each other via their PPC domains. It would be interesting to test if the preservation of physical interactions between AHL proteins is also conserved among those from more distantly related plant species, such as between AHLs from the moss *Physcomitrella patens* and angiosperms, or from monocot and dicot plants. In addition, we have predicted the orthologous and homologous *AHL* genes in the examined plant species (Additional files 12 and 13). It would be intriguing to examine if the orthologous/homologous AHLs share similar interactions, genetic and/or physical, with other non-AHL orthologous/homologous partners.

### Biological functions of the AHL proteins at AT-rich chromosomal DNA

Besides the potential for shared physical interacting partners, the AHL proteins in the land plant species examined in this study also contain either one copy of Type-I or -II AT-hook motifs or both types. These two types of AT-hook motifs have also been found in the mammalian HMGA proteins [34]. Mammalian HMGA1 binds to AT-rich DNA and serves as an architectural protein which alters the local chromatin state and modulates gene expression through both protein-protein and protein-DNA interactions [70-73]. The similar possession of AT-hook motifs by both AHLs and HMGAs suggest that they may share binding affinities for AT-rich DNA.

This association of the AT-hook motif with the PPC domain is likely to physically direct these plant PPC domains to AT-rich chromosomal regions. This notion is supported by the observation that *Arabidopsis thaliana* AHL1 binds to the AT-rich scaffold/matrix attachment regions (S/MARs) and its AT-hook motif is indispensable for AHL1’s DNA binding capacity [32]. The S/MARs have been suggested to primarily localize near the transcription start sites [74,75] or correlate with the origins of DNA replication [76]. Several *Arabidopsis thaliana* AHL proteins bind to gene promoter regions and serve as transcriptional regulators [38,77]. Therefore, it is likely that the potential targeting of the PPC protein to the S/MARs is correlated with functions in gene transcriptional regulation. It would be interesting to examine and compare the biological functions of both PPC proteins in Bacteria and Archaea with the AHL proteins in land plants in order to shed light on the potential evolutionarily conserved functions of this domain.

In this study, we proposed an evolutionary hypothesis for the diversification of *AHL* genes, from a prokaryotic single-copy gene encoding the PPC protein lacking an AT-hook motif, to three types of land plant AHL proteins incorporating two types of PPC domains and two types of AT-hook motifs. However, the biological functions of these three types of AHL proteins still need to be determined. Further experiments need to be performed to reveal their binding sites along plant chromosomes and the corresponding biological regulatory roles. It should be noted that the inferred evolutionary events in this study are based on the retrieved full-length *AHL* sequences available from current releases of completely sequenced plant genomes. Further analysis should incorporate sequences from additional plant species (particularly ferns and gymnosperms) to improve our understanding of the diversification and functional evolution of the three types of AHL proteins.

## Conclusion

In this study, over 500 full-length *AHL* genes have been identified from 19 fully sequenced plant genomes, ranging from the early diverging *Physcomitrella patens* and *Selaginella* to a variety of monocot and dicot flowering plants. Our analyses suggest that the *AHL*s can be classified into three types (Type-I/-II/-III) based on the number and composition of their functional domains, the AT-hook motif(s) and PPC domain. We further inferred their phylogenies in land plants via Bayesian inference analysis. The *AHL* genes emerged in embryophytes and have evolved into two distinct clades with Type-I *AHL*s diversifying in Clade-A and the other two types together diversifying into Clade-B. Our study indicates that Clade-A and -B *AHL*s diverged before the separation of moss *Physcomitrella patens* from the vascular plant lineage. In angiosperms, Clade-A *AHL*s expanded into 5 subfamilies; while, the ones in Clade-B expanded into 4 subfamilies.

Examination of their expression patterns suggests that the *AHL*s within each clade share similar patterns of expression with each other. While, the *AHL*s between the two clades exhibit distinct expression patterns from each other, suggesting potential conserved biological functions within each clade since their divergence along land plant evolution.

Manipulating the *AHL* genes has been suggested to have tremendous effects to positively affect agriculture through increasing seedling establishment, plant biomass and improving plant immunity [33,42,78,79]. Our analyses suggest that the *AHL* genes from different land plant species may share conserved functions in regulating plant growth and development. Over-expression of a *Glycine max* AHL PPC domain in *Arabidopsis thaliana* recapitulates the phenotype observed when over-expressing its *Arabidopsis thaliana* counterpart. Our study further suggest that such functional conservation may be due to conserved physical interactions among the PPC domains of AHL proteins. In the end, our analyses reveal a possible evolutionary scenario for the *AHL* gene family in land plants, which will facilitate the design of new studies probing their biological functions and subsequently lead to improvements in crop biomass production.

## Methods

### Data retrieval

The amino acid sequences as well as coding sequences for the members of the *Arabidopsis thaliana AHL* gene family were retrieved from the TAIR website [32,41,80] and were further used as queries for gene search using BLAST, TBLASTN, BLASTP and PSI_BLAST for *AHL* genes in the Phytozome database [46] within the related plant species with a cut-off E value set at 1e^−2^. The obtained results were further used as additional queries. Only intact gene sequences comprised of both AT-hook motif(s) and PPC domain were included and used as additional queries to perform in-depth gene searches in the Phytozome database. For further phylogenetic analysis, only protein sequences were used.

### Cloning of *AHL* genes from *Glycine max* and *Triticum aestivum*

Genomic DNA as well as mRNA were prepared from *Triticum aestivum* seedlings using DNeasy plant mini kit (Qiagen) and RNeasy plant mini kit (Qiagen). cDNA was further prepared using iScript Advanced cDNA Synthesis Kit for RT-PCR (Bio-RAD). Primer pairs (*TaAHL1*: 5′-ATG GGG AGC ATG GAC GGC CAC CC-3′ and 5′-CTA GAA TGA CGT CGG CGG AGG CCG C-3′; TaAHL3: 5′-ATG GCC ACC GGC AGC AGC AAG TGG TG-3′ and 5′-TCA GAT GCC GCC TCC CTG GTG GCC TC-3′) were used to clone *TaAHL1* and *TaAHL3* from both prepared genomic DNA and cDNA, correspondingly, and examined to be free-of-introns. Amino acid sequences of TaAHL1 and TaAHL3 proteins were predicted from their coding sequences and were used for further phylogenetic analysis. The nucleotide sequences of *TaAHL1* and *TaAHL3* have been deposited into NCBI GenBank (Accession numbers: *TaAHL1/Taq1*, KJ461850; *TaAHL3/Taq3*, KJ461851). Genomic DNA of *Gm06g01650.1* was prepared from 6 day-old seedlings using ZR Plant DNA MiniPrep kit (Zymo Research). Coding sequence of its PPC domain was cloned using the primer pair (5′-TCC CCC CGG G A TGA AGC CAC CCG TCA TAG TCA CGC GCG AC-3′ and 5′-AAC TGC AGT CAA TCA TCA TCA TGC TGA TTC AAG G-3′). The amplicon and binary vector *pCHF3* were digested by XmaI with PstI and ligated together. The resulted plasmid was subsequently transformed into agrobacterium *GV3101* and further transformed into *Arabidopsis thaliana Col-0* by the floral dipping method [81]. Surface-sterilized seeds were sown on 0.5× Linsmaier and Skoog modified basal medium (1.0% w/v phytogel and 1.5% w/v sucrose) and grown for 5 days at 25°C under 25 μmol∙s^−1^∙m^−2^ white light in a Percival E-30B growth chamber.

### Yeast two-hybrid assay

A *lexA*-based Y2H system was used to test protein-protein interactions in yeast. The targeted yeast two-hybrid assay was performed as described in [33].

### Sequence alignment and phylogenetic analyses

The amino acid sequences of the Type-I AHL proteins were aligned using MUSCLE [82,83] and were further manually adjusted. Bayesian inference analysis was performed with the MrBayes 3.2.1 on XSEDE tool on CIPRES Science Gateway for 20 million generations with convergence at 0.022 [84]. The amino acid sequences of the Type-II and -III AHL proteins were aligned and manually adjusted. Bayesian inference analysis was performed with the MrBayes 3.2.1 for 10 million generations with convergence at 0.017. Generations were both sampled every 10,000 generations and the first 25% was set as burn-in.

### Secondary and tertiary structure prediction

The amino acid sequences of the PPC domains were retrieved from the coding sequences of *Gm06g01650.1, TaAHL1* (*Taq1*) and *TaAHL3* (*Taq3*). The secondary and tertiary structures were predicted using the RaptorX Structure Prediction Server [85,86]. The tertiary structure figures were prepared using Pymol version 1.3 (The PyMOL Molecular Graphics System, Schrodinger, LLC).

### Inference of gene duplication and loss event

The plant species tree was adapted from the one in the Phytozome database [46]. The gene trees obtained from Bayesian inference analysis for each of the two AHL clades were reconciled with the plant species tree individually by Notung 2.6 [87] with default parameters. The orthologous and paralogous genes were further inferred by the Notung 2.6 program [87].

### Availability of supporting data

The wheat *AHL* genes, *TaAHL1* and *TaAHL3* were deposited into NCBI GenBank (Accession numbers: *TaAHL1/Taq1*, KJ461850; *TaAHL3/Taq3*, KJ461851). All supporting data are included as additional files and have been uploaded to LabArchives, LLC. DOI: 10.6070/H4PC30B2.

## Additional files

Additional file 1:
**Chromosomal Locations of** ***AHL*** **Genes Identified in** ***Arabidopsis thaliana*** **.** The *AHL* genes that are resulted from gene duplication were paired with red (adjacent pairs) and blue (distant pairs) lines. Additional file 2:
**Chromosomal Locations of** ***AHL*** **Genes Identified in** ***Oryza sativa*** **.**
Additional file 3:
**Amino acid sequences of the AHL proteins used in the analysis.**
Additional file 4:
**Sequence logo analysis of PPC domain of AHL proteins.** Sequence logo analysis of the Type-A PPC domain **(a)** and Type-B PPC domain **(b)** in land-plant AHL proteins. The conserved six-amino-acid region was pointed out by the red boxes. Additional file 5:
**Amino acid sequences of wheat TaAHL1, TaAHL3 and soybean Gm06g01650.1.** The AT-hook motif is underlined with green. The PPC domain is underlined with blue. Additional file 6:
**Alignment of two** ***Arabidopsis thaliana*** **AHLs, AtAHL27 and AtAHL29, with soybean Gm06g01650.1.** The AT-hook motif is underlined with green. The PPC domain is underlined with blue. Additional file 7:
**Sequence logo analysis of the AT-hook motifs.** Sequence logo analysis of the Type-I AT-hook motif from **(a)** the land-plant AHLs that only contain Type-I, not Type-II AT-hook, and from **(b)** the AHLs that also contain Type-II AT-hook. Sequence logo analysis of the Type-II AT-hook motif in **(c)** the AHLs that only contain Type-II, not Type-I AT-hook, and **(d)** the AHLs that also contain Type-I AT-hook. The star symbol represents the core sequence of the AT-hook motif. The conserved sequence downstream of the core sequences in Type-I and Type-II AT-hook motifs were pointed out by the triangle and diamond symbols accordingly. Additional file 8:
***PPC/DUF296*** **Genes Identified in Selected Picoeukaryotes and Prokaryotes.**
Additional file 9:
**Intron-mediated transcriptional enhancement of** ***AHL*** **genes in** ***Arabidopsis thaliana*** **.** **(a)** Topology of the exon and intron arrangement of *Arabidopsis thaliana AHL* genes. **(b)** The intron-mediated enhancement scores of the first/second/third/fourth intron were shown. The grey line represents a score of 10, which indicates moderate capability of transcriptional enhancement. **(c)** The intron-mediated enhancement scores of each intron in *AtAHL*s were listed. The abilities of transcriptional enhancement were categorized with strong (orange color), relatively strong (light orange), moderate (light blue) and weak (no color). Additional file 10:
**Expression analysis of the** ***AHL*** **genes in** ***Arabidopsis thaliana*** **using Genevestigator V3.0.** Similarities between expression profiles of *AHL* genes were calculated using Manhattan Distance method (www.genevestigator.com) [67]. Type-I *AHL*s were labeled in blue color. Type-II *AHL*s were labeled in green color. Type-III *AHL*s were labeled in red color. Additional file 11:
**Expression Analysis of the** ***AHL*** **Genes in** ***Zea mays*** **Using Genevestigator V3.0.** Similarities between expression profiles of *AHL* genes were calculated using Pearson Correlation method (www.genevestigator.com) [67]. Type-I *AHL*s were labeled in blue color. Type-II *AHL*s were labeled in green color. Type-III *AHL*s were labeled in red color. Additional file 12:
**Inferred orthologs and paralogs of the Clade-A** ***AHL*** **genes in land plant species.**
Additional file 13:
**Inferred orthologs and paralogs of the Clade-B** ***AHL*** **genes in land plant species.**

## Abbreviations

AHL: AT-hook motif nuclear localized
PPC/DUF296: Plant and prokaryote conserved/domain of unknown function #296
SOB3: Suppressor of phytochrome B-4 #3
ESC: ESCAROLA
HRC: HERCULES
*Al*: *Arabidopsis lyrata*
*At*: *Arabidopsis thaliana*
*Bd*: *Brachypodium distachyon*
*Bra*: *Brassica rapa*
*Cpa*: *Carica papaya*
*Cs*: *Cucumis sativus*
*Gm*: *Glycine max*
*Mdp*: *Malus domestica*
*Mes*: *Manihot esculenta*
*Mt*: *Medicago truncatula*
*Os*: *Oryza sativa*
*Pp*: *Physcomitrella patens*
*Ppa*: *Prunus persica*
*Pt*: *Populus trichocarpa*
*Rc*: *Ricinus communis*
*Sb*: *Sorghum bicolor*
*Sm*: *Selaginella moellendorffii*
*Vv*: *Vitis vinifera*
*Zm*: *Zea mays*
A: Angiosperms
B: Brassicaceae
Em: Embryophyta
Eu: Eudicots
F: Fabidae (Eurosids I)
G: Grasses
Mp: Malpighiales
Mv: Malvidae (Eurosids II)
NF: Nitrogen fixing
T: Tracheophyta (vascular plants)
