# Supplementary figures and images for: Insights into the evolution and diversification of the AT-hook Motif Nuclear Localized gene family in land plants
Source: BMC Plant Biol. 2014 Oct 14;14:266. doi: 10.1186/s12870-014-0266-7 (PMC4209074; doi:10.1186/s12870-014-0266-7)

# *Arabidopsis thaliana*

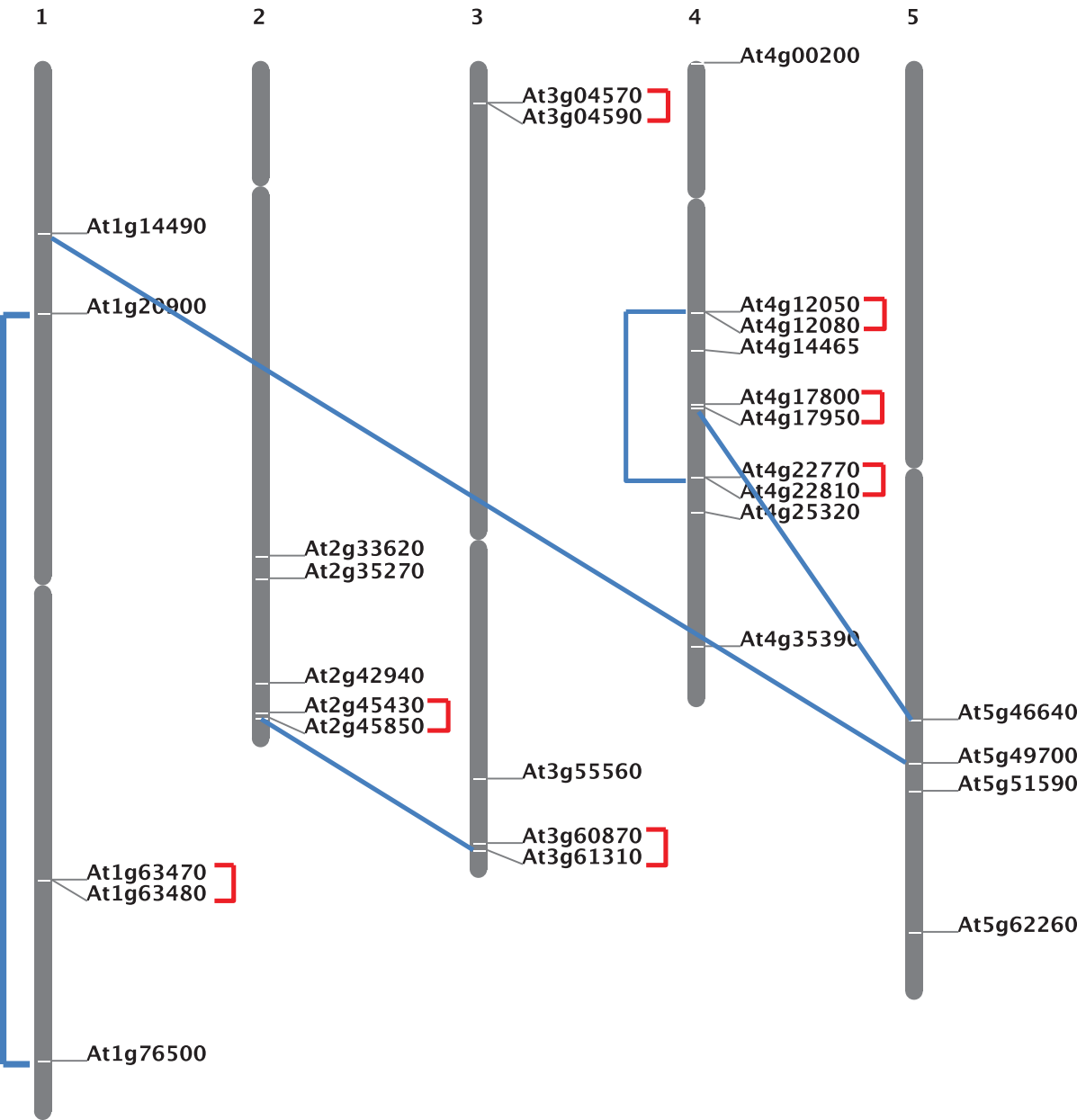

Supplement: Additional file 1: — Chromosomal Locations of AHL Genes Identified in Arabidopsis thaliana . The AHL genes that are resulted from gene duplication were paired with red (adjacent pairs) and blue (distant pairs) lines. [file 12870_2014_266_MOESM1_ESM.pdf]

# *Oryza sativa*

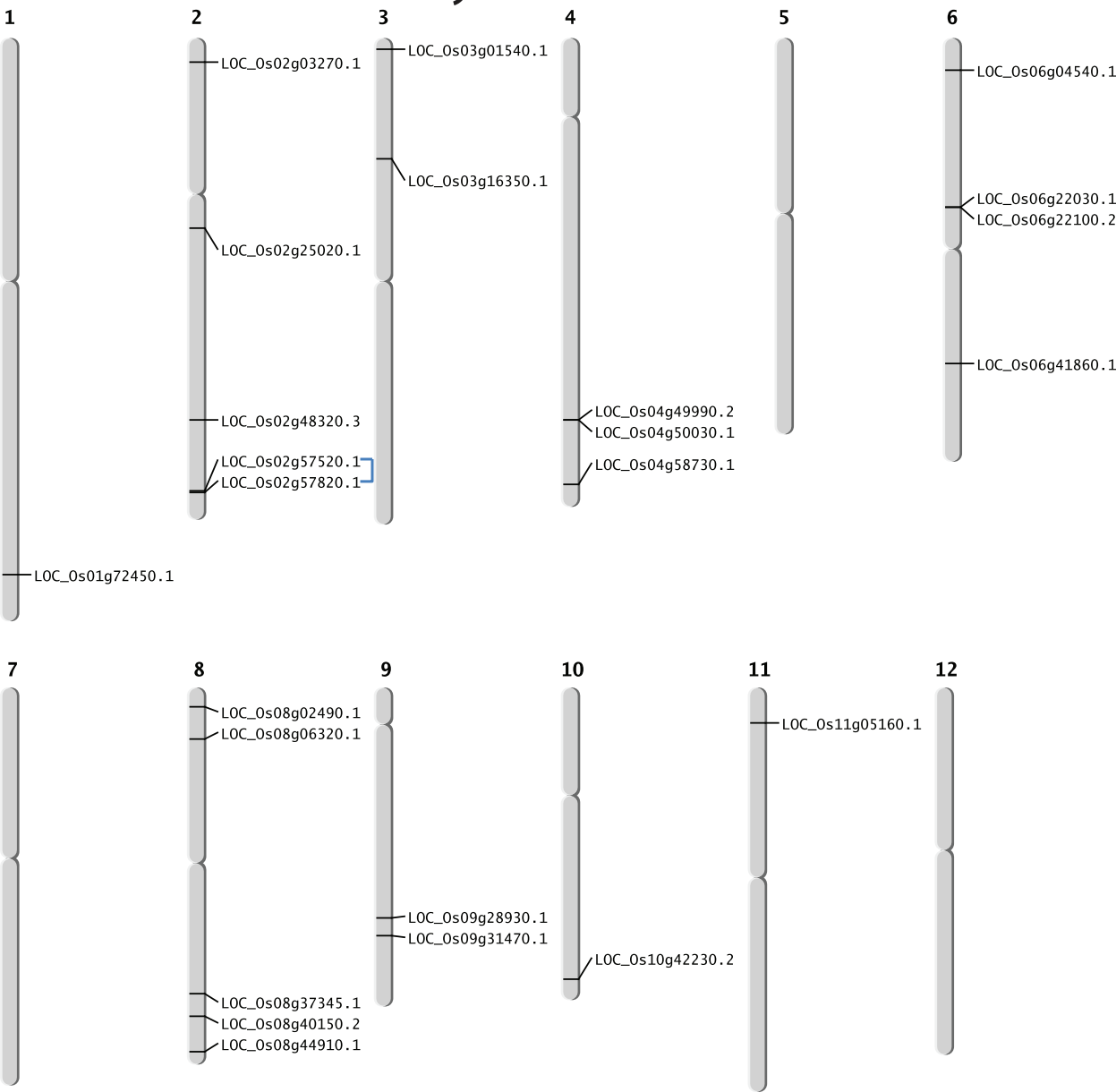

Supplement: Additional file 2: — Chromosomal Locations of AHL Genes Identified in Oryza sativa . [file 12870_2014_266_MOESM2_ESM.pdf]

(a)

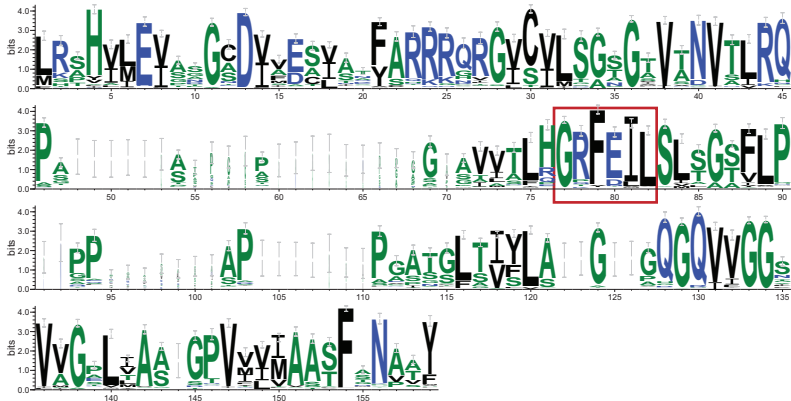

(b)

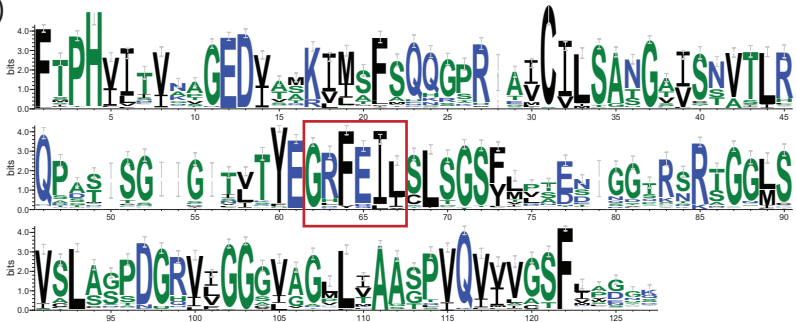

Supplement: Additional file 4: — Sequence logo analysis of PPC domain of AHL proteins. Sequence logo analysis of the Type-A PPC domain (a) and Type-B PPC domain (b) in land-plant AHL proteins. The conserved six-amino-acid region was pointed out by the red boxes. [file 12870_2014_266_MOESM4_ESM.pdf]

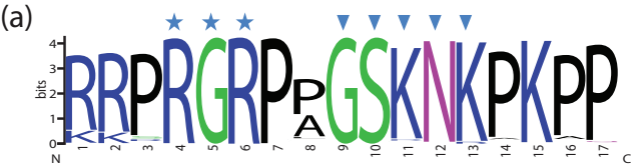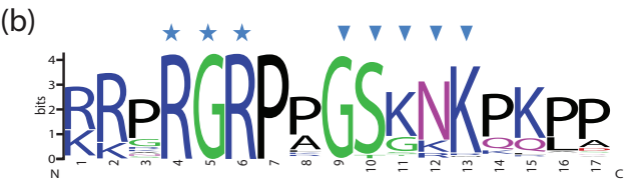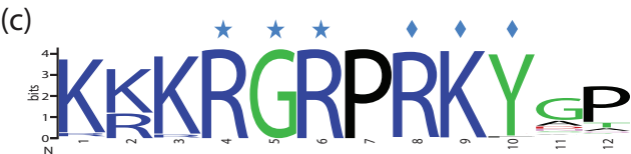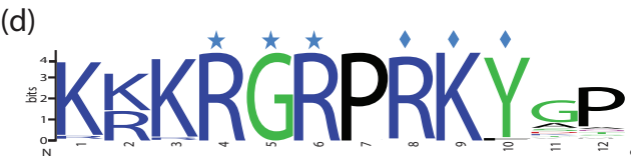

Supplement: Additional file 7: — Sequence logo analysis of the AT-hook motifs. Sequence logo analysis of the Type-I AT-hook motif from (a) the land-plant AHLs that only contain Type-I, not Type-II AT-hook, and from (b) the AHLs that also contain Type-II AT-hook. Sequence logo analysis of the Type-II AT-hook motif in (c) the AHLs that only contain Type-II, not Type-I AT-hook, and (d) the AHLs that also contain Type-I AT-hook. The star symbol represents the core sequence of the AT-hook motif. The conserved sequence downstream of the core sequences in Type-I and Type-II AT-hook motifs were pointed out by the triangle and diamond symbols accordingly. [file 12870_2014_266_MOESM7_ESM.pdf]

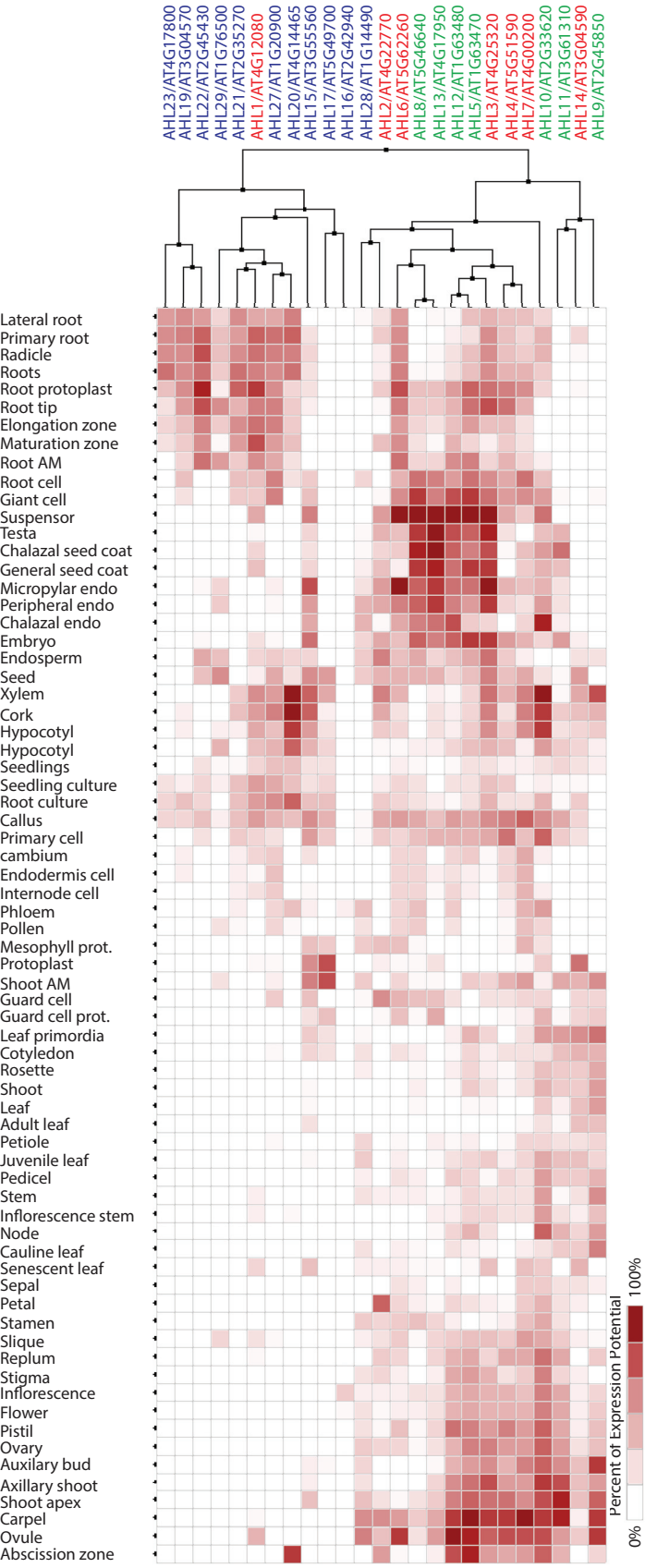

Supplement: Additional file 10: — Expression analysis of the AHL genes in Arabidopsis thaliana using Genevestigator V3.0. Similarities between expression profiles of AHL genes were calculated using Manhattan Distance method (www.genevestigator.com) [67]. Type-I AHLs were labeled in blue color. Type-II AHLs were labeled in green color. Type-III AHLs were labeled in red color. [file 12870_2014_266_MOESM10_ESM.pdf]

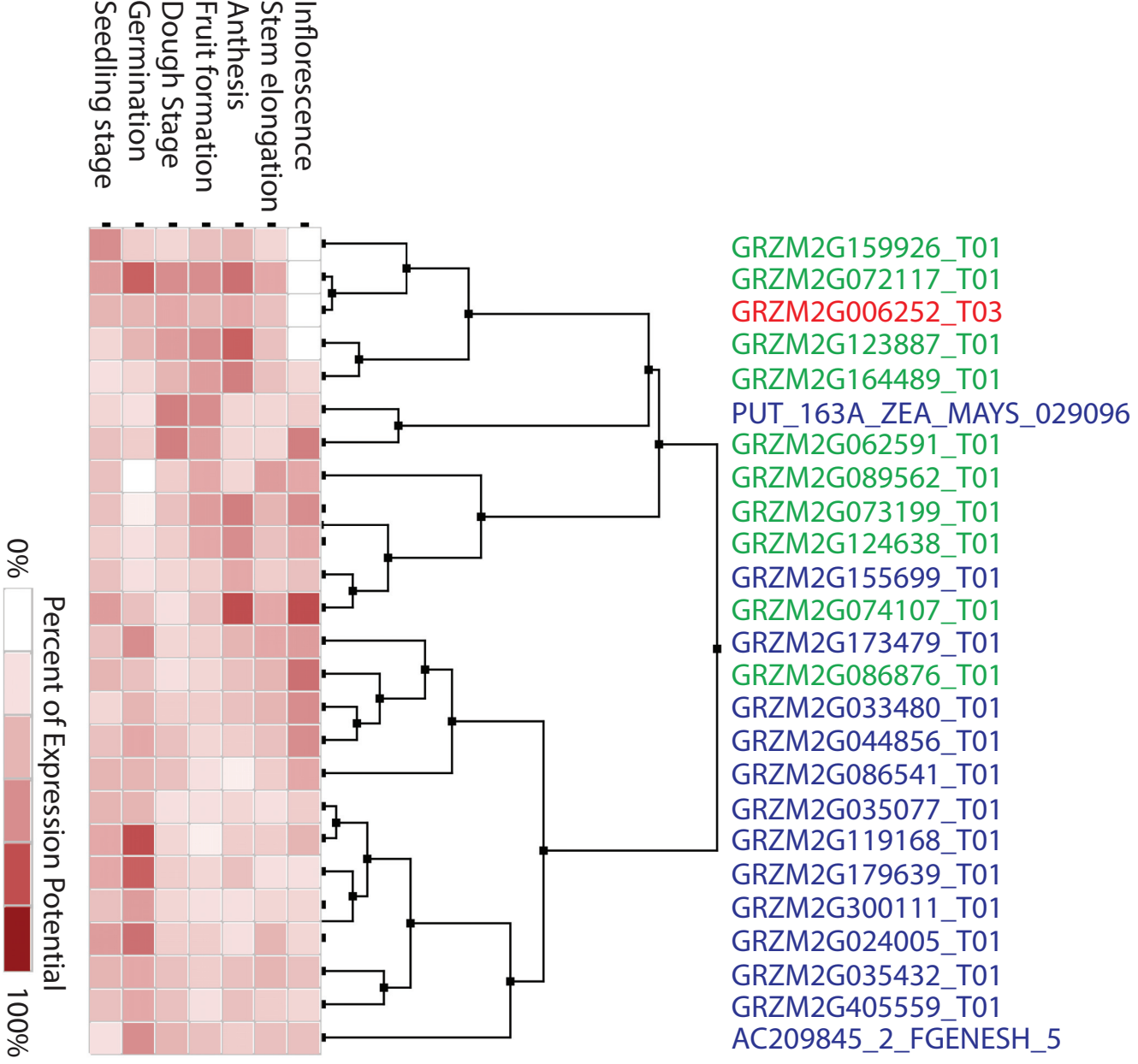

Supplement: Additional file 11: — Expression Analysis of the AHL Genes in Zea mays Using Genevestigator V3.0. Similarities between expression profiles of AHL genes were calculated using Pearson Correlation method (www.genevestigator.com) [67]. Type-I AHLs were labeled in blue color. Type-II AHLs were labeled in green color. Type-III AHLs were labeled in red color. [file 12870_2014_266_MOESM11_ESM.pdf]
