# Supplementary material for: Insights into the evolution and diversification of the AT-hook Motif Nuclear Localized gene family in land plants
Source: BMC Plant Biol. 2014 Oct 14;14:266. doi: 10.1186/s12870-014-0266-7 (PMC4209074; doi:10.1186/s12870-014-0266-7)
Supplement: Additional file 3: — Amino acid sequences of the AHL proteins used in the analysis. [file 12870_2014_266_MOESM3_ESM.docx]

**Additional File 3. Amino acid sequences of the AHL proteins used in the analysis.**

>Al483394

MAGGTALTPTSVGSKSVPMRNNEAAERGNNNNNNNNLKALPKAVQPVSSIEGEMAKRPRGRPAGSKNKPKPPIIVTHDSPNSLRANAVEISSGCDICETLSDFARRKQRGLCILSANGCVTNVTLRQPASSGAIVTLHGRYEILSLLGSILPPPAPLGITGLTIYLAGPQGQVVGGGVVGGLIASGPVVLMAASFMNAVFDRLPMDDDEAASMQNQQYYQNGRSRPLDDIHGLPQNLLTNGNSASDIYSWGPAQRAMSKP

>Al493459

MANPWWTNQSGLAGMVDHSASSGHHQNHHHQSILTKGDLGIAMNQSQDNDQDEDDDPREGAVEVVNRRPRGRPPGSKNKPKAPIFVTRDSPNALRSHVLEISDGSDVAETIAHFSRRRQRGVCVLSGTGSVANVTLRQAAAPGGVVSLQGRFEILSLTGAFLPGPSPPGSTGLTVYLAGVQGQVVGGSVVGPLLAIGSVMVIAATFSNATYERLPMEEEEDGGGSRQIHGGGDSPPGIGSSLPDLSGMAGPGYNMPPHLIPNGAGQLGHEPYTWVHARPPY

>Al493093

MAGLDLGTAFRYVNHQLHRPDLHLHHNSSSDDVTPGAGMGHFTVDDEDNNNNNNHQGLDLASGGGSGSSGGGGGHGGGGDVVGRRPRGRPPGSKNKPKPPVIITRESANTLRAHILEVTNGCDVFDCVATYARRRQRGICVLSGSGTVTNVSIRQPSAAGAVVTLQGTFEILSLSGSFLPPPAPPGATSLTIFLAGGQGQVVGGSVVGELTAAGPVIVIAASFTNVAYERLPLEEDEQQQQLGGGSNGGGNLFPEVAAGGGGGLPFFNLPMNMQPNVQLPVEGWPGNSSGRGPF

>Al938493

MANPWWVGNVAIGGVESPVTSSAPSLHHRNSNNPPTMTRSDPRLDHDFTTNNCGSPNTQTQSQEEQNSRDEQPAVEPGSGSGSTGRRPRGRPPGSKNKPKSPVVVTKESPNSLQSHVLEIATGADVAESLNAFARRRGRGVSVLSGSGLVTNVTLRQPAASGGVVSLRGQFEILSMCGAFLPTSGSPAAAAGLTIYLAGAQGQVVGGGVAGPLIASGPVIVIAATFCNATYERLPIEEEQQQEQPLQLEDGKKQKEENDDNESGNNGNEGSMQPPMYNMPPNFIPNGHQMAQHDVYWGAP

PPRAPPSY

>Al476807

MDGGYDQTGGASRYFHNLFRPELQHQLQPQPQLQPLPQPQQQQSDDESDSNKDPGSDPVTSGSTPGKRPRGRPPGSKNKPKPPVIVTRDSPNVLRSHVLEVSSGADIVESVTTYARRRGRGVSILSGNGTVANVSLRQPAAAHGANGGTGGVVALHGRFEILSLTGTVLPPPAPPGSGGLSIFLSGVQGQVIGGNVVAPLVASGPVILMAASFSNATFERLPLEDEGGEGAGGEVGEVGEGGGGGGGPPTATSSSPPSGAGQGQLRGNMSGYDQFSGDPHLLGWGAAAAPRPPF

>Al935500

MDQVSRSLPPPFLSRDLHLHPHHQFQHQQQQQQNHGHDIDQHRIGGLKRDRDADIDPNEHSSAGKDQNTPGSGGESGGGGGGDNHITRRPRGRPAGSKNKPKPPIIITRDSANALKSHVMEVANGCDVMESVTVFARRRQRGICVLSGNGAVTNVTIRQPASVPGGGSSVVNLHGRFEILSLSGSFLPPPAPPAASGLTIYLAGGQGQVVGGSVVGPLMASGPVVIMAASFGNAAYERLPLEEDDQEEQTAGAVANNIDGNATMGGGTQTQTQTQQQQQQQQQLMQDPTSFIQGLPPNLMNSVQLPAEAYWGTPRPSF

>Al486561

MDEVSRSHPPHFLSSDLHHYHHQNAGLKRDREEDDVEPNNDGKDQDTTPSEGGNIKKRRPRGRPAGSKNKPKAPIIVTRDSANAFRCHVMEITNGCDVMESLAVFARRRQRGVCVLTGNGAVTNVTVRQPGGGVVSLHGRFEILSLSGSFLPPPAPPAATGLTVYLAGGQGQVIGGSLVGPLMASGPVVIMAASFGNAAYERLQLEEEETDREIDGNASMAVGTQTQKQLMHDATSFVQGSPSNLINSVSLPSEAYWGTQRPSFQDNILII

>Al492588

MDHGTQNSLPAPFHARDFQLHLQQQQQEFFLHHHQQQRNQTDDDQQGGSGGNRQIKMDREETSDNIANNSGSEGKDIDLHGGSGEGGGGSGGDHQMTRRPRGRPAGSKNKPKPPIIVTRDSANALRTHVMEIGDGCDLVESVATFARRRQRGVCVMSGTGNVTNVTIRQPGSHPSPGSVVSLHGRFEILSLSGSFLPPPAPPTATGLSVYLAGGQGQVVGGSVVGPLLCAGPVVVMAASFSNAAYERLPLEEDEMQTPVHGGGGGGGGSMESPPMMGQQLQHQQQAMSGHQGLPPNLLGSVQLQQQHDQSYWSTGRPPY

>Al490024

MDPVQSHGSQSSLPPPFHARDFQLHLQQQQQQQQHQQQQQFFLHHHQQPQRNPDQDHEQQGGSIMNRSIKMDREETSDNMDNIANNNSGSEGKEMSLHGGEGGSGEQMTRRPRGRPAGSKNKPKAPIIITRDSANALRTHVMEIGDGCDIVDCMATFARRRQRGVCVMSGTGNVTNVTIRQPGSPPGSVVSLHGRFEILSLSGSFLPPPAPPAATGLSVYLAGGQGQVVGGSVVGPLLCSGPVVVMAASFSNAAYERLPLEEDEMQTPVHGGGGGGGEGGGMGSPPMMGQQQAMAAMAAAQGLPPNLLGSVQLPPPQQNDQQYWSTGRPPY

>Al482492

MAGLDLGTTSRYVHNVDGGGGGQFTTDNHHEDDGGAGGNHHHHHNNHNHHQGLDLIASNDNSGLGGGGGGGSGDLVMRRPRGRPAGSKNKPKPPVIVTRESANTLRAHILEVGSGCDVFECISTYARRRQRGICVLSGTGTVTNVSIRQPTAAGAVVTLRGTFEILSLSGSFLPPPAPPGATSLTIFLAGAQGQVVGGNVVGELMAAGPVMVMAASFTNVAYERLPLDEHEEHLQVQSGGGGGGGNMYSEATGGGGGLPFFNLPMSMPQMGVESWPGNHAGAGRAPF

>Al477713

MANPWWTGQVNLSGLETTPPGSSQLKKPDLHISMNMAMDSGHNNHHHHQEVDNNNNDDDRDNLSGDDHEPREGAVEAPTRRPRGRPAGSKNKPKPPIFVTRDSPNALKSHVMEIASGTDVIETLATFARRRQRGICILSGNGTVANVTLRQPSTAAVAAAPGGAAVLALQGRFEILSLTGSFLPGPAPPGSTGLTIYLAGGQGQVVGGSVVGPLMAAGPVMLIAATFSNATYERLPLEEEEAAERGGGGGSGGVVPGQLGGGGSPLSSGAGGGDGNQGLPVYNMPGNLVSNGGSGGGQMSGQEAYGWAQARSGF

>Al491108

MAGYMHPLLGQELHLQRPEDSRTPPDQNNMELNRSEADEAKAETTPTGGAASSATASGSSSGRRPRGRPAGSKNKPKPPTIITRDSPNVLRSHVLEVTSGSDISEAVSTYATRRGCGVCIISGTGAVTNVTIRQPAAPAGGGVITLHGRFEILSLTGTALPPPAPPGAGGLTVYLAGGQGQVVGGNVAGSLIASGPVVLMAASFANAVYDRLPIEEEETPPPRATGVQQQQPEASQSSEVTGSGAQACESNLQGGNGGGGVAFYNLGMNMNNFQFSGGDIFGMSGSNGGGGGGLTRPAF

>Al472326

MEGGYEQGGGASRYFHNLFRPEIHHQQLQPQGGINLIDQHHHQHQQHHQQQQQQQKSDDSRESDHSNKDHHQQGRPDSDPNTSSSAPGKRPRGRPPGSKNKAKPPIIVTRDSPNALRSHVLEVSPGADIVESVSTYARRRGRGVSVLGGNGTVSNVTLRQPVNPGNGGGVSGGGGVVTLHGRFEILSLTGTVLPPPAPPGAGGLSIFLAGGQGQVVGGSVVAPLIASAPVILMAASFSNAVFERLPIEEEEEEGGGGGGGGGGGPPQMQQAPSASPPSGVTGQGQLGGNVGGYGFSGDPHLLGWGAGTPSRPPF

>Al949481

MKGEYREQKSNEMFSKLPHQQQQQHSLTSHFHLSSTATPTIDDSSIEVVRRPRGRPPGSKNKPKPPVFVTRDTDPPMSPYILEVPSGNDVVEAINRFCRRKSIGVCVLSGSGSVANVTLRQPSPAAPGSTITFHGKFDLLSVSATFLPPPPRTSLSPPVSNFFTVSLAGPQGQIIGGFVAGPLISAGTVYVIAASFNNPSYHRLPAEEEQKHSAGTGEREGQSPPVSGGGEESGQMAGSGGESCGVSMYSCHMGGSDVIWAPTARAPPPY

>Al471623

METVGRPRGRPQGSKNKPKAPIFVTIDPPMSPYILEVPSGNDVVEALNRFCRRKAIGFCVLSGSGSVADVTLRQPSPAAPGSTITFHGKFDLLSVSATFLPPPPQTSLPPPFSNFFTVSLAGPQGQVIGGFVAGPLVAAGTVYVVATSFNNPAYHRLPAAEEEQRDSAEGEGQSPPVSGSGGESMYSCHMGGSDVIWSPNAKAPPSH

>AtAHL17

MKGEYREQKSNEMFSKLPHHQQQQQQQQQQHSLTSHFHLSSTVTPTVDDSSIEVVRRPRGRPPGSKNKPKPPVFVTRDTDPPMSPYILEVPSGNDVVEAINRFCRRKSIGVCVLSGSGSVANVTLRQPSPAALGSTITFHGKFDLLSVSATFLPPPPRTSLSPPVSNFFTVSLAGPQGQIIGGFVAGPLISAGTVYVIAASFNNPSYHRLPAEEEQKHSAGTGEREGQSPPVSGGGEESGQMAGSGGESCGVSMYSCHMGGSDVIWAPTARAPPPY

>AtAHL28

METVGRPRGRPRGSKNKPKAPIFVTIDPPMSPYILEVPSGNDVVEALNRFCRGKAIGFCVLSGSGSVADVTLRQPSPAAPGSTITFHGKFDLLSVSATFLPPLPPTSLSPPVSNFFTVSLAGPQGKVIGGFVAGPLVAAGTVYFVATSFKNPSYHRLPATEEEQRNSAEGEEEGQSPPVSGGGGESMYVGGSDVIWDPNAKAPSPY

>AtAHL15

MANPWWVGNVAIGGVESPVTSSAPSLHHRNSNNNNPPTMTRSDPRLDHDFTTNNSGSPNTQTQSQEEQNSRDEQPAVEPGSGSGSTGRRPRGRPPGSKNKPKSPVVVTKESPNSLQSHVLEIATGADVAESLNAFARRRGRGVSVLSGSGLVTNVTLRQPAASGGVVSLRGQFEILSMCGAFLPTSGSPAAAAGLTIYLAGAQGQVVGGGVAGPLIASGPVIVIAATFCNATYERLPIEEEQQQEQPLQLEDGKKQKEENDDNESGNNGNEGSMQPPMYNMPPNFIPNGHQMAQHDVYWG

GPPPRAPPSY

>AtAHL16

MAGGTALTPTSVGSKSVPMRNHEATERGNTNNNLRALPKAVQPVSSIEGEMAKRPRGRPAGSKNKPKPPIIVTHDSPNSLRANAVEISSGCDICETLSDFARRKQRGLCILSANGCVTNVTLRQPASSGAIVTLHGRYEILSLLGSILPPPAPLGITGLTIYLAGPQGQVVGGGVVGGLIASGPVVLMAASFMNAVFDRLPMDDDEAASMQNQQYYQNGRSRPLDDIHGLPQNLLTNGNSASDIYSWGPAQRVMSKP

>AtAHL18

MDEVSRSHTPQFLSSDHQHYHHQNAGRQKRGREEEGVEPNNIGEDLATFPSGEENIKKRRPRGRPAGSKNKPKAPIIVTRDSANAFRCHVMEITNACDVMESLAVFARRRQRGVCVLTGNGAVTNVTVRQPGGGVVSLHGRFEILSLSGSFLPPPAPPAASGLKVYLAGGQGQVIGGSVVGPLTASSPVVVMAASFGNASYERLPLEEEEETEREIDGNAARAIGTQTQKQLMQDATSFIGSPSNLINSVSLPGEAYWGTQRPSF

>AtAHL19

MANPWWTGQVNLSGLETTPPGSSQLKKPDLHISMNMAMDSGHNNHHHHQEVDNNNNDDDRDNLSGDDHEPREGAVEAPTRRPRGRPAGSKNKPKPPIFVTRDSPNALKSHVMEIASGTDVIETLATFARRRQRGICILSGNGTVANVTLRQPSTAAVAAAPGGAAVLALQGRFEILSLTGSFLPGPAPPGSTGLTIYLAGGQGQVVGGSVVGPLMAAGPVMLIAATFSNATYERLPLEEEEAAERGGGGGSGGVVPGQLGGGGSPLSSGAGGGDGNQGLPVYNMPGNLVSNGGSGGGGQMSGQEAYGWAQARSGF

>AtAHL20

MANPWWTNQSGLAGMVDHSVSSGHHQNHHHQSLLTKGDLGIAMNQSQDNDQDEEDDPREGAVEVVNRRPRGRPPGSKNKPKAPIFVTRDSPNALRSHVLEISDGSDVADTIAHFSRRRQRGVCVLSGTGSVANVTLRQAAAPGGVVSLQGRFEILSLTGAFLPGPSPPGSTGLTVYLAGVQGQVVGGSVVGPLLAIGSVMVIAATFSNATYERLPMEEEEDGGGSRQIHGGGDSPPRIGSNLPDLSGMAGPGYNMPPHLIPNGAGQLGHEPYTWVHARPPY

>AtAHL21

MAGLDLGTTSRYVHNVDGGGGGQFTTDNHHEDDGGAGGNHHHHHHNHNHHQGLDLIASNDNSGLGGGGGGGSGDLVMRRPRGRPAGSKNKPKPPVIVTRESANTLRAHILEVGSGCDVFECISTYARRRQRGICVLSGTGTVTNVSIRQPTAAGAVVTLRGTFEILSLSGSFLPPPAPPGATSLTIFLAGAQGQVVGGNVVGELMAAGPVMVMAASFTNVAYERLPLDEHEEHLQSGGGGGGGNMYSEATGGGGGLPFFNLPMSMPQIGVESWQGNHAGAGRAPF

>AtAHL22

MDQVSRSLPPPFLSRDLHLHPHHQFQHQQQQQQQNHGHDIDQHRIGGLKRDRDADIDPNEHSSAGKDQSTPGSGGESGGGGGGDNHITRRPRGRPAGSKNKPKPPIIITRDSANALKSHVMEVANGCDVMESVTVFARRRQRGICVLSGNGAVTNVTIRQPASVPGGGSSVVNLHGRFEILSLSGSFLPPPAPPAASGLTIYLAGGQGQVVGGSVVGPLMASGPVVIMAASFGNAAYERLPLEEDDQEEQTAGAVANNIDGNATMGGGTQTQTQTQQQQQQQLMQDPTSFIQGLPPNLMNSVQLPAEAYWGTPRPSF

>AtAHL23

MAGLDLGTAFRYVNHQLHRPDLHLHHNSSSDDVTPGAGMGHFTVDDEDNNNNHQGLDLASGGGSGSSGGGGGHGGGGDVVGRRPRGRPPGSKNKPKPPVIITRESANTLRAHILEVTNGCDVFDCVATYARRRQRGICVLSGSGTVTNVSIRQPSAAGAVVTLQGTFEILSLSGSFLPPPAPPGATSLTIFLAGGQGQVVGGSVVGELTAAGPVIVIAASFTNVAYERLPLEEDEQQQQLGGGSNGGGNLFPEVAAGGGGGLPFFNLPMNMQPNVQLPVEGWPGNSGGRGPF

>AtAHL24

MDPVQSHGSQSSLPPPFHARDFQLHLQQQQQEFFLHHHQQQRNQTDGDQQGGSGGNRQIKMDREETSDNIDNIANNSGSEGKDIDIHGGSGEGGGGSGGDHQMTRRPRGRPAGSKNKPKPPIIITRDSANALRTHVMEIGDGCDLVESVATFARRRQRGVCVMSGTGNVTNVTIRQPGSHPSPGSVVSLHGRFEILSLSGSFLPPPAPPTATGLSVYLAGGQGQVVGGSVVGPLLCAGPVVVMAASFSNAAYERLPLEEDEMQTPVHGGGGGGSLESPPMMGQQLQHQQQAMSGHQGLPPNLLGSVQLQQQHDQSYWSTGRPPY

>AtAHL25

MSSYMHPLLGQELHLQRPEDSRTPPDQNNMELNRSEADEAKAETTPTGGATSSATASGSSSGRRPRGRPAGSKNKPKPPTIITRDSPNVLRSHVLEVTSGSDISEAVSTYATRRGCGVCIISGTGAVTNVTIRQPAAPAGGGVITLHGRFDILSLTGTALPPPAPPGAGGLTVYLAGGQGQVVGGNVAGSLIASGPVVLMAASFANAVYDRLPIEEEETPPPRTTGVQQQQPEASQSSEVTGSGAQACESNLQGGNGGGGVAFYNLGMNMNNFQFSGGDIYGMSGGSGGGGGGATRPAF

>AtAHL26

MDPVQSHGSQSSLPPPFHARDFQLHLQQQQQHQQQHQQQQQQQFFLHHHQQPQRNLDQDHEQQGGSILNRSIKMDREETSDNMDNIANTNSGSEGKEMSLHGGEGGSGGGGSGEQMTRRPRGRPAGSKNKPKAPIIITRDSANALRTHVMEIGDGCDIVDCMATFARRRQRGVCVMSGTGSVTNVTIRQPGSPPGSVVSLHGRFEILSLSGSFLPPPAPPAATGLSVYLAGGQGQVVGGSVVGPLLCSGPVVVMAASFSNAAYERLPLEEDEMQTPVQGGGGGGGGGGGMGSPPMMGQQQAMAAMAAAQGLPPNLLGSVQLPPPQQNDQQYWSTGRPPY

>AtAHL27

MEGGYEQGGGASRYFHNLFRPEIHHQQLQPQGGINLIDQHHHQHQQHQQQQQPSDDSRESDHSNKDHHQQGRPDSDPNTSSSAPGKRPRGRPPGSKNKAKPPIIVTRDSPNALRSHVLEVSPGADIVESVSTYARRRGRGVSVLGGNGTVSNVTLRQPVTPGNGGGVSGGGGVVTLHGRFEILSLTGTVLPPPAPPGAGGLSIFLAGGQGQVVGGSVVAPLIASAPVILMAASFSNAVFERLPIEEEEEEGGGGGGGGGGGPPQMQQAPSASPPSGVTGQGQLGGNVGGYGFSGDPHLLGWGAGTPSRPPF

>AtAHL29

MDGGYDQSGGASRYFHNLFRPELHHQLQPQPQLHPLPQPQPQPQPQQQNSDDESDSNKDPGSDPVTSGSTGKRPRGRPPGSKNKPKPPVIVTRDSPNVLRSHVLEVSSGADIVESVTTYARRRGRGVSILSGNGTVANVSLRQPATTAAHGANGGTGGVVALHGRFEILSLTGTVLPPPAPPGSGGLSIFLSGVQGQVIGGNVVAPLVASGPVILMAASFSNATFERLPLEDEGGEGGEGGEVGEGGGGEGGPPPATSSSPPSGAGQGQLRGNMSGYDQFAGDPHLLGWGAAAAAAPPRPAF

>Bd3g11600.1

MDPVTASIHGHHLPPPFNTRDFHHHLQQQQLLHLKTEDDQGGGTPAAGALGGRGTKRDHHDDDENSGNHGDGGGGELALIPTCANSGGGGSGENGSRRPRGRPAGSKNKPKPPIIITRDSANTLRTHVMEVAGGCDISESITAFARRRQRGVCVLSGAGTVTNVTLRQPASQGAVVALHGRFEILSLSGSFLPPPAPPEATGLTVYLAGGQGQVVGGSVVGALTAAGPVVIMAASFANAVYERLPLEDEDLLAQGGDSGAGMLPGAGGQMDPNLFQGLPPNLLGNVQLPPPEAAAGGYGWNPGAAGGRPSPF

>Bd3g12530.1

MANRWWDEGRDLGPPAPSAGEPSSLSPPPLGAAKNEEDGTESPNGADAGAGPGAVVTGNRRPRGRPAGSKNKPKPPIFVTRDSPNALRSHVMEVAGGADIADAIAAFARRRQRGVCVLSGAGTVADVALRQPAAGSVVALRGRFEILSLTGTFLPGPAPPGSTGLTVYLAGGQGQVVGGSVVGALTAAGPVMVIASTFANATYERLPLDEVDAAGAEEDGHGNGMPPPHSEATMASMMPVHGVDPSMFGGGMPAMAGGAGLQLGHDLAWAQAQHQHQHARPPPPY

>Bd3g16390.1

MAGLDLGTVSYLHHHQQQQLHLLQGHDDGGGSDGGGQDELSPGSGGAAAPGGGGIGGVGGEVVGRRPRGRPPGSKNKPKPPVIITRESANALRAHILEVAAGCDVFEALTAYARRRQRGVCVLSAAGAVANVTIRQQPSNSSSSSSPVVATLQGRFEILSLAGSFLPPPAPPGATSLAAFLAGGQGQVVGGSVAGPLVAAGPVVVVAASFSNVAYERLPLEDDADEVAPPPPSAMDPFGASADPSTGGGGGHGGGLPFFNQLPPGLGMPPPPMAMDGHNGWPAGVGRPPFS

>Bd3g53220.1

MAGMDPGPGGGSPGGASSSRYFHHLLRPQQQQQQPSPLSPTSHVKMEHSKLTSPDNNNSPAGGDAAADAGGGSGDQPSSSAMAPDGSGGSGGPTRRPRGRPAGSKNKPKPPIIVTRDSPNALHSHVLEVAAGADIVDCVAEYARRRGRGVCVLSGGGAVVNVALRQPGASPPGSVVATLRGRFEILSLTGTVLPPPAPPGASGLTVFLSGGQGQVIGGSVVGSLVAAGPVVLMAASFANAVYERLPLEGEEEDQAAAAAAAAAAGAEAQQDQVAQSAGPHGQQPAASQSSGVTGGDAAGGMSLYNLAGNVGNYQLPGDNFGGWGGGGGGAGGVRPPF

>Bd3g55720.1

MIKDPEPGDSNNADSGSGSGGGNGTTNNGAEPRGGDPGTVVLPAPNRRPRGRPPGSKNKPKPPIFVTRDSPNALRSHVMEVAGGADVADAIAHFSRRRQRGVCVLSGAGTVANVALRQPSAPGGAVVALHGRFEILSLTGTFLPGPAPPGSTGLTVYLAGGQGQVVGGSVVGALTAAGPVMVIASTFANATYERLPLEEEDEGPVQGGGGVEQQLGMAGGHGHGVPVDPSAAMPMAPMFNGQLGGGGGDGFPWAPHARPPY

>Bd4g32490.1

MSFGKGDMSKENMYHDPNKDMPAIRRFAAPPPPPPQPMHQHHGGHGEQPHPHPQQQQLECFSDEVDSRGTPEPKKEPAGGGGGSGAHLASGGGGDGSSIEVAKKRRGRPPGSKNKPKPPVVITREAEPAAAMRPHVIEIPGGRDIAEALSRFAGRRGLGICVLAGTGAVANVSLRHPCSPATAALAPPGLAAPAAVVVVQGRYEILSISATFLPPAMAAAMDMAPQAAAAMAAAGISISLAGPHGQIVGGAVAGPLYAATTVVVVAAAFTNPTFHRLPIADEDASVSVSGSADAVADEHRAHPHPHQQHQPEPPEQRHLRRQPPHLAASTSGAPPVEPCGVSIYGCHAQPQPQEVIWPPAAHQAPRPPPPY

>Bd4g38870.1

MAAKQADGEHSGGGISGHDDDPEPKEGAVVVPANRRPRGRPPGSKNKPKPPIFVTRDSPNALRSHVMEVSSGADIADSIAHFSRRRQRGVCVLSGAGAVADVALRQPAAPGGAVVALRGRFEILSLTGTFLPGPSPPGSTGLTVYLAGGQGQVVGGSVVGTLTAAGPVMVIASTFANATYERLPLDDEAEEDRHELAGRGVPGSTGQMAPGVPPMMMGDHSAGMSMYGLPASLMPGGGGGHAQAAAEQGINAWAPQHARPPY

>Bd5g19920.1

MAGLDLGTAATRYVHQFHHLHPDLQLQNNSYAKQQHEPSDDHDNGNNNYGAGQQYGADNNNNNDGGSSSSGPGAGGGDGTPGSGGDVVARRPRGRPPGSKNKPKPPVIITRESANTLRAHILEVGSGCDVFECVSTYACRRQRGVCVLSGSGVVTNVTLRQPSAPAGAVVTLQGRFEILSLSGSFLPPPAPPGATSLTVFLAGGQGQVVGGNVVGALYAAGPVIVIAASFANVAYERLPLEDEEQQQAAAAAAAAGGMQQMQQAGDADGPGGGGGMGGVPGFPPDPSAAGLPFFNHLPINHMGAGGAGSQLPPDGHGWAGPRPHF

>Bd2g30430.1

MAWWAGGMGGHGLDLGNHLAQQFGGSGVMAEQAPTTPNSSGSNNNHHDESSGAGAQGQDSPSAGAGETSPTPNATSGGSGGGGGSSSGRRPRGRPPGSKNKPKPPIIITRESPNTLRSHVLEIASGADIMDAVATFARRRQRGVSVLSGSGVVGNVTLRQPAAPPGAVVTLHGRFEILSLSGAFLPSPCPPGATGLAVYLAGGQGQVVGGTVVGELVASGPIMVVAATFSNATYERLPLVDEELAAATGDAVPTGSDGMQQLPEGPPPPGAGGNGAVMGGGLPPDSASMPFYSSLPPNLIPNGQMPQHDVFASFRPPPPAF

>Bd1g35720.1

MPPPSSTAPAAPPAAAASAQSQDQPAPGAAGAMVPLRKPRGRPLGSKNKPKPPVIITRDSPDALHSHVLEVSPGADVSACVAQYARARGRGVCVLGASGTVADVAVRVPGAPAAGALPLTLPGRFELLSVTGTVLPPPAPAEASGLAVLLAAGQGQVLGGRVVGPLVAATPVTLFAATFANAVYERLPLQDDAVVDVKPNVSAADAQQAQQPQELPLAMSQAMAMGGAAGYPVVHRASPPYAWGGGHGHGSGGI

>Bd1g51520.1

MACLPENPRPLAPRGSSAAEAKPKHKPRLALASRTFASFDLAIRGRRRMDPVAAHGGGGGRHHFGPPVGSPFHSPFHGSHGHGAGGQFQQQAAPQFQAYELHGHQAQMLANSMGGGGGNSGSSMLAKQELVDESTINSAGSNSAGEQGMGSAEPQIMGQTQAGGVGGGGEDPHQQHGAAGLRQGVMRRPRGRPAGSKNKPKPPVIITRDSASALRAHVLEVAPGCDVVDAVADFARRRQVGVCVLSATGSVAGISVRQPGGGGGSNGNGNGGVVSIAGRFDILTLSGSFLPQPAPPSATGLTVYVSGGSGQVVGGAVAGALVATGGPVVIMAASFGNASYERLPLDDEPPQSAAPDLAPLPAPLHQQQQQQQSLAMMNAIQLPGDEDEAGGYGGWASAGAGSSRVGPY

>Bd1g66880.1

MGSMDGHSLHHHGGGGGYTHVAASGPAGSNNDEQQQDDASPPPSGASGGGGGGSAGRRPRGRPPGSKNKPKPPVVVTRESPNAMRSHVLEIASGADIVEAIAAFSRRRQRGVSVLSGSGAVTGVTLRQPAGMAGNGAPAVALRGRFEILSLSGAFLPAPAPPGATGLAVYLAGGQGQVVGGSVMGELLASGPVMVIAATFGNATYERLPLDEASQADAEEAGAVLSGSSEGGGAAQLLEQQGSGGGTAVAHPPMYASVPQPTPTHDMFGQWGQQARPPPPTSF

>Bd4g33850.1

MMEVRASSEQQGVMAGREPFGLPKTPPPSSNPMQQNMHLAYTAEGRPYYAQTAQNQSGGGDGAAGPDADAAEGNGSPEHQGNMEEMARKKSGQPSNEDSDGSMSAALVPVPNPAEVTPGASGTLSPAARNTAGTVPSAAPVGMKKRGRPKGSTNKVKKQKSVPDTTGFVGAHFTPHAICVNAGEDVAAKIMSFSQHGSRGVCVLSANGAISNVTIRQADTSGGTVTYEGRFEILSLSGSFLESENGGHRSRTGGLSVSLASSNGRVLGGGVAGLLTAATPIQIIVGSFDTATEKKAPKKQRAPSDPSSSSAPPQMAPVIASAAMAVPAVTTPVAEPIAPVPLSVAMAAGPSGESSSAAGNQLNHGATANDNTQNQGLSSMSWK

>Cs302400.1

MDSHSLPPPFHTRDFHLQQHPFHTNTNNNNSEEEHSTTTTRLKRDRDDDTNNSNPNSAGDPTPDGEITRRPRGRPAGSKNKPKPPIIITRDSANALRTHVIEVTDGCDIVDSVATFARRRQRGVCIMSGTGTVTNVTLRQPASPGAIVNLHGRFEILSLAGSFLPPPAPPAATTLTIYLAGGQGQVVGGSVVGTLIASGPVVIMAASFSNAAYERLPLEEDDQPQLPSLQGGGGIGSPDEVGQSQITAQTAHHQQQQQNQQQQQQLLNDGNAPLFHGLPPNLLNSIQMPPSESPYWATARPPY

>Cs165130.1

MANRWWAENIPTTTDSSTSNPYSTPLKQSLEVADEENNSGSHERAEPGTSSSTRRPRGRPPGSKNKPKPPVVVTKESPDALRSHVLEIGSGSDIVESISNFAQRRQRGVSVLSGNGVVANVTLRHPGASGGVITLQGRFDILSLSGAFLPAPAPPGATGLTVYLAGGQGQVVGGIVVGALVATGPVIVIAATFTNATFERLPLEDEEVAAGDKSGTSQNNSTSQSMGEQQQQQQQPSMGVYNMTPNLVTNGQVSSHDMIWSLPRAPPPF

>Cs362470.1

MATLNLGSASHFVDQLQQQRPDLHLDSPPSSDHVNHFNGSGGSGGSGDVMVRRPRGRPAGSKNKPKPPVIITRESANTLRAHILEVGGGCDVFEAVAGYARRRQRGICVLSGSGIVNNVSLRQPAAAGSVLTLQGRFEILSLSGSFLPPPAPPGATSLTIFLAGGQGQVVGGNVVGALIASGPVIVIASSFSNVAYERLPLDEEEMPMQAGGGDGDGGEGGGEGHNNPFPDASSGLPFLNLPMNMPNQNQFFG

>Cs362860.1

MDGITPHNRPLPPPFLSKDLHLHHGLFHAHHQNSDDDHTPGPKRDRDSDDNPTMDDDTKELSNSSSRRPRGRPAGSKNKPKPPIIITRDSANALRSHLIEISTASDIVDSLATFARRRQRGVCILSATGTVANVTLRQPSSPGAVITLPGRFEILSLSGSFLPPPAPPAASGLTVYLAGGQGQVVGGNVIGPLSASGPVIIMAASFGNAAYERLPIDDEDETSPAPDQMAGQQAAAAPPPPQLLGDPNGGLFHGMAQNVVNSSCQLPGEAAAAFWGGGRPPY

>Cs099950.1

MSDLNSAVHGASSRNANQFLGSDLQLQNFSQTHLPDPNSADSTTPTTGGSSSRRPRGRPAGSKNKPKPPVIVTRDSPNSLRSHVLEVSPGSDVVESISTYVTRRRYGVCILGGTGAVTNVNLRQPMSPSGSVMTLHGTFEIVSLTGTALPPSGAGGLTIYLADRQRQGHVVGGSVVGPLRASSPVTLMVASFTNAVYDRLPVEEAEPPVQAQASASPSSDITGGGGQLRGIESFNLRDDGYGWNNNASRPPTTL

>Cs313230.1

MAGLDLGNRYIHQLQTPNFNLPIGHHHHHHHPDSQSRHDDDDSPHHPLEFVPTTTTDMVASRRPRGRPPGSKNKPKPPVIITRESANTLRAHILEVGSGCDVFDCIATYARRRQRGICILSGNGMVTNVNLRQPTATGSVLTLQGRFEILSLSGSFLPPPAPPGATSLTIYLAGGQGQVVGGNVVGELVAAGPVTIIAASFTNVAYERLPLEDQEQEQQEQEQDQMQQPTSQGGGNNVGGGGSNNPFPDPSSGLPFFNLPINMQNQIQLPF

>Cs206200.1

MDRATAAHGRPLPPPFISRDFHLNPHHHFLHQNPDQHYNENGSSGSGGGDGEVLRRPRGRPAGSKNKPKPPTIITRDSANALRCHVIEIANANDVIETLTIFARQRQRGICVLTGAGAVTNVTLKQPVSTAGAVISLPGRFEILSLSGSFLPPPAPAAASGLTVYLSGGQGQVVGGSVVGPLMSSGPVVITAASFGNAAYERLPVEDDDVEAADAGSSPIRSPENAVQQQQFLPDFHGLAPNLMNTCQLPTEPYWGTGRTPPF

>Cs383780.1

MFKTYHTSKSNFRLPLPFSIFANLANSNPWWVSPVGFPTTAPISTTVDGSGRDKDEEEDEAKGGGVEVGNRRSRGRPPGSKNKRKSPIIVTRDSPHTLSTHVIEIVGGADVADSINQFCCRRQRGVCVLSGSGTVVDVTVRQSAGSGAVIQLRGRFEILSVSGSFLPGRDPPCSTGLTVYLAGGQGQVIGGTVVGPLLAGGPVILIAATFANATYERLPLQHHHNYEEREVSPATTSAGELEEPLPYPRIETSIYDLIPPNNNNNHALDGYAWTHDRPSLV

>Cs321770.1

MAGLDLGTRYLTHLQPQVDGGHFSSGDHQDDDDPHQTLGGTGDVVGRRPRGRPPGSKNKPKPPVIITRESANTLRVHILEVGSGCDVFDCVASYARRRQRGICILSGSGNVTNVGLRQPAAAGVLTLQGRFEILSLSGSFLPPPAPPGATSLTIFLAGGQGQVVGGTVAGELTAAGPVILIAASFTNVAYERLPLDEEDQQQTPAGGGGSGGGVGNNNNNNPFQDPSNSGLPFLNLPLHMQNVQLPPF

>Cs160670.1

MLSKFHLSPHPFTHHPPPQPPVDEPIAALPSPFKHHTDLTSTADGSTIEVVRRPRGRPPGSKNKPKPPLVVTREPEPAMRPYVLEVPGGNDVVEAISRFSRRKNLGLCVLNGSGTVANVSLRQPSATPGATVTFHGRFEILSISATVFPQSTPLPLPNGFSISLAGPQGQIVGGLVAGALIAAGTVFVVASSFNNPFYHRLPDEEEIKNLGSGGGSGGGEVHSPHVSGGGDSSGQGHGHGQIAETCGMAMYSCHAPSDVIWAPTARQPPPPY

>Cs257240.1

MAGLDLGSASHFVHQLQHRSVDLHLQHQTDVDDGSDHQPNSGGEIVARRSRGRPPGSKNKPKPPVIITRESANTLRAHILEVNTGCDVFDSVATYARKRQRGVCILSGTGAVTNVTLRQPSSTGGAITLPGRFEILSLTGSFLPPPAPPGATSLTIFLAGGQGQIVGGNVVGSLIASGPVIVIASSFTNVAYERLPLDEEEQPPNGGGSLSNPFPDPSVGLPLFNMPSSNMAGNQNQLPVDGWGGGNSGGRASY

>Cs111460.1

MAGGDQADMAVHSLGSKLSLISDHEPEKCGNNNVTNNHQQRPCFDPSLVSPKVVPPISVAAESDQALRRPRGRPAGSKNKPKPPIIVTRDSANALRAHAIEVSTGCDVNESLSNFARRKQRGVCILSGSGCVTNVTLRQAASSGAIVTLHGRFEILSMLGSILPPPAPSGITGLTIYLSGAQGQVVGGVVVGALIASGPVVIMAATFMNATFDRLPSDDEEVAATMQSQHYGQNGRSHHHLDVSDLYGVPQNLITNSSLPPELYSWATAGRTMSKT

>Cs136170.1

MANWGGIGNLPTTPLEFQKTEQQHSTKNYEDGDGVKPKRRPRGRPRGSKNKPKPPILIAKITPNTLQTHVFEIATATDIADSIFTFTQRRRRGVSILSATGLVTDITLRQPPGVITLHQRFEILSLSGAFLPTPSPHGTSALTVYLAGDQGRVVGGLVAGPIIAAGPVVVVAASFTNAMYEKLPMEENEEKTEEDKQLEENINGNKNSMGESSSLAAASSLGVHNLIPNTQISQETFWAPPPPPSY

>Cs355580.1

MADYGGAISLSHQPPTSSSSSDDHSPPTRPQTKPSRTPTSGGAASSVDTSTMKKPRGRPPGSKNKPKPPIVITKENESSMKPVVIEISAGNDVVDTLLHFARKRHVGLTVLSGSGSVSNVTLRHPMSHSTSLSLHGPFSLVSLSGSFLANTTPFSSKPHSLSPSPSPSPSSSFGICLAGAQGQVFGGIVGGKVTAASLVVVVAATFINPVFHRLPSETTEGEDDRVDMAKPTINATDESPVTATTTSSATPMTVCVYNAPSPPDHAMPWVPSSRSSY

>Cs132980.1

MMANRWWTSGQMGLPGVDHTSTSSSAMRKPDLGISMNDNGGPVHSGGDDDDDRDNGGDEPKEGAVEVPTRRPRGRPPGSKNKPKPPIFVTRDSPNALKSHVMEISNGADIAESVAQFARRRQRGVSVLSGSGTVTNVTLRQPSAPGAVLALQGRFEILSLTGTFLPGPAPPGSTGLTIYLAGGQGQVVGGSVVGPLTAAGPVMVIAATFSNATYERLPLEEEEEGGGVGAQGHTSAGGGGAGDGSPQGIGGGVGDPSAMTPLYNLPPNLLPNGGGGQLNQEAYSWAHGGRPSF

>Gm06g09810.1

MKGEYLEQQQQHPKSETTPPSMFSKLQPQHHPFPHHPFQLSAEEENRVGAATTPSTVQKANSSGGDGATIEVVRRPRGRPPGSKNKPKPPVIITRDPEPAMSPYILEVSGGNDVVEAIAQFSRRKNMGICVLTGSGTVANVTLRQPSTTPGTTVTFHGRFDILSVSATFLPQQSGASPAVPNGFAISLAGPQGQIVGGLVAGGLMAAGTVFVIAASFNNPAYHRLPPEEEGASAGDGHSPQVSGGGDSGHGQAESCGMSMYSCHLPSDVIWAPTARPQPPPPPY

>Gm10g01140.1

MDLSINERSLVRERQEEEEEEEDERDNGDEPKEGAVEAGTRRPRGRPPGSKNKPKPPIFVTRDSPNSLRSHVMEVAGGADVAESVAQFARRRQRGVCVLSGSGSVANVTLRQPSAPGAVVALHGRFEILSLTGAFLPGPAPPGATGLTVYLAGGQGQVVGGSVVGSLVAAGPVMVIAATFANATYERLPLEEEEDDGGGSVQGGSTLGGSPHGIGSSGGGGGSGGGHLPGGIPGPSSLPLYNLPPNLLPNGGQVGHEAFAWAHGHGRPPY

>Gm10g31020.1

MANRWWTGSVGLENSGHSMKKPDLGFSMNESTVTGNHIGEEDEDRENSDEPREGAIDVATTRRPRGRPPGSRNKPKPPIFVTRDSPNALRSHVMEIAVGADIADCVAQFARRRQRGVSILSGSGTVVNVNLRQPTAPGAVMALHGRFDILSLTGSFLPGPSPPGATGLTIYLAGGQGQIVGGGVVGPLVAAGPVLVMAATFSNATYERLPLEDDDQEQHGGGGGGGSPQEKTGGPGEASSSISVYNNNVPPSLGLPNGQHLNHEAYSSPWGHSPHARPPF

>Gm03g02580.1

MDPVAAQGRPLPPPFLTRDLHLHPHHQFQPHHNNQNTEDEAGNGRGQKRDRDENVGGGGGATTPPHGGGEGKEPGSEDGGGSDMGRRPRGRPAGSKNKPKPPIIITRDSANALRSHVMEITNGCDIMESVTAFARRRQRGICLLSGSGTVTNVTLRQPASPSAVVTLHGRFEILSLSGSFLPPPAPPAASGLAIYLAGGQGQVVGGSVVGPLVASGPVVIMAASFGNAAYERLPLEEEETPVPYHGTEGLGSPGIPGTQQQSQSQPQQQQQQLVGDPNSSSLFHGVPQNLLNSIQLPAEGYWGGSARPPF

>Gm20g34430.1

MGPSKHHMLATVANPWWTGQGGLSGVDHPGTHSPGLGKRPSDLGISENSGGHNREEDEDNRDEPKEGAVEVGTRRPRGRPPGSKNKPKPPIFVTRDSPNTLRSHVMEVTGGADVAESVAQFARRRQRGVCVLSGSGSVANVTLRQPSAPGAVVALHGRFEILSLTGTFLPGPAPPGSTGLTVYLTGGQGQIVGGSVVGSLVAAGPVMVIAATFANATYERLPLDEDDEGPSSAAGAQGGGSSPPPPLGIGSSGGGQLQGGMPDPSSMPLYNLPPNGGVGQVGHEALAWAHGRAPF

>Gm20g36460.1

MANRWWTGSVGLENSGDSMKKPDLGFSMNESAVTGNHTGEEEEKENSDEPREGAIDVSTTRRPRGRPPGSKNKPKPPIFVTRDSPNALRSHVMEIAAGADIADCVAQFARRLQRGVSILSGSGTVVNVTIRQPTAPGAVMALHGRFDILSLTGSFLPGPSPPGATGLTIYLAGGQGHVVGGGVVGPLLAAGPVLLMAATFSNATYERLPLEDDDQEHGGGGGSPPGITGGPGEASSSISVYNNNVPPNLGLSNGQHLNHEAYSSSPW

>Gm01g34580.1

MPIIPHHQFQPHHNHQNTEDEAGNGRGQKRDRDENAGGGGGATTPPQGGGEGKESGSGDGGGSDMGRRPRGRPAGSKNKPKPPIIITRDSANALRSHVMEIANGCDIMESITAFARRRQRGVCVLSGSGTVTNVTLRQPASPGAVVTLHGRFEILSLSGSFLPPPAPPAASGLAIYLAGGQGQVVGGSVVGPLVASGPVVIMAASFGNAAYERLPLEEEETPVAVAGNGGLGSPGIPGTQQQPQQQQQQQLVGDPNSSSLFHGMPQNLLNSVQLPAEGYWGGSARPPF

>Gm01g40680.1

MAGLDLGTASRFVQSLHRPDFNQQQESEEDAKPQDGPQQGDVVGRRPRGRPAGSKNKPKPPVIITRESANALRAHILEVASGCDVFESVASYARRRQRGICILSGSGTVTNVSLRQPASAGAVATLHGRFEILSLTGSFLPPPAPPGATSLSIYLAGGQGQVVGGSVVGELTAAGPVIVIAASFTNVAYERLPLEEEEEQVQISGAAANNNNNSNPYHDPSSGLPFFNLPMNVQLPVDAWAGNSTTRSPF

>Gm01g42230.1

MDPITAHGHSLPPPFHAGRDLHLHQQQHQFHSLQEDEQSGSSGGLNLAHKREHEENNNNNSSDGKEGGGAGSGETEISRRPRGRPAGSKNKPKPPIIITRDSANALKTHVMEVADGCDIVDSVSAFARRRQRGVCIMSGTGTVTNVTLRQPASSGAVVTLHGRFEILSLAGSFLPPPAPPAASGLTIYLAGGQGQVVGGSVVGALIASGPVVIMSASFSNAAYERLPLEDEDPSMALQGGGSIGSPGGGGGGGGGVGQQQPSQQLMGDSTAPLFHGLNPNLLNSVQMPSETFWATGRSPY

>Gm17g16660.1

MAGLDLGSASRFVQNLHRPDLHLQQNFQQHQDQQHQRDLEEQKTPPNHRMGAPFDDDSDDRSPGLELTSGPGDIVGRRPRGRPPGSKNKPKPPVIITRESANTLRAHILEVGSGSDVFDCVTAYARRRQRGICVLSGSGTVTNVSLRQPAAAGAVVTLHGRFEILSLSGSFLPPPAPPGATSLTIYLAGGQGQVVGGNVIGELTAAGPVIVIAASFTNVAYERLPFREMNNSSNNSSFRFSHWNDVVSRKQQQQ

>Gm05g04080.1

MDPISAHGHSLPPPFHTRDLHLHHQQQQHQFQSLNQATEDENSGSSGAQKREREENSNNNEGAGEAEITRRPRGRPAGSKNKPKPPIIITRDSANAMRTHMMEVADGCDIVESVSEFARKRQRGVCIMSGTGTVNNVTLRQPASSGSVVTLHGRFEILSLSGSFLPPPAPPAASGLTIYLAGGQGQVVGGSVVGTLVASGPVVIMAASFSNAAYERLPLEDEDPSLQMQGGSIGSPGASGVGQSQLLGGDATAPLFHGLPPNLLNSVQMPSEPFWAASARPPF

>Gm05g23630.1

MAGLDLGSASRFVQNLHLPDLHLQQNYQQPRHKRDSEEQETPPNPGTALAPFDNDDDKSQGLELASGPGDIVGRRPRGRPSGSKNKPKPPVIITRESANTLRAHILEVGSGSDVFDCVTAYARRRQRGICVLSGSGTVTNVSLRQPAAAGAVVRLHGRFEILSLSGSFLPPPAPPGATSLTIYLAGGQGQVVGGNVVGELTAAGPVIVIAASFTNVAYERLPLEEDEQQQQQLQIQSPATTSSQGNNNNNPFPDPSSGLPFFNLPLNMQNVQLPPF

>Gm18g04060.1

MANRWWAGNVGMIREQELMENSNNNNNNNNATTTTPTNSSNSNTNANTNTNTTEEEVSRDNGEDQNQNLGSHEGSEPGSSGRRPRGRPAGSKNKPKPPIVITKESPNALRSHVLEIASGSDVAESIAAFANRRHRGVSVLSGSGIVANVTLRQPAAPAGVITLHGRFEILSLSGAFLPSPSPSGATGLTVYLAGGQGQVVGGNVAGSLVASGPVMVIAATFANATYERLPLEDDQGEEEMQVQQQQQQQQQQQQQQQQQQSQGLGEQVSMPMYNLPPNLLHNGQNMPHDVFWGAPPRPPPSF

>Gm18g48260.1

MAGIDLGSASHFVHHRLERPDLEDDENQQDQDNNLNNHEGLDLVTPNSGPGDVVGRRPRGRPPGSKNKPKPPVIITRESANTLRAHILEVSSGCDVFESVATYARKRQRGICVLSGSGTVTNVTLRQPAAAGAVVTLHGRFEILSLSGSFLPPPAPPGATSLTVFLGGGQGQVVGGNVVGPLVASGPVIVIASSFTNVAYERLPLDEEESMQMQQGQSSAGGGGSGGGVSNNSFPDPSSGLPFFNLPLNMPQLPVDGWAGNSGGRQSY

>Gm11g03130.1

MDPITAHGHSLPPPFHTARDLHLHHQQQQHQFHSLQEDEQSGSSGGLNLAAHKREREENNNSDGKEGGAGSAETEISRRPRGRPAGSKNKPKPPIIITRDSANALKTHVMEVADGCDIVESVSAFARRRQRGVCIMSGTGTVTNVTLRQPASSGAVVTLHGRFEILSLAGSFLPPPAPPEASGLTIYLAGGQGQVVGGSVVGALIASGPVVIMSASFSNAAYERLPLEDEEPSIGLQGGGSIGSPGGGGGVGQSQAPHQLMGDSTTAPLFHGLNPNLLNSVQMPSTETFWATGRSPYC

>Gm11g04630.1

MAGLDLSTASPFVQSLHRPDFNQQHDSGEDAKQSAEDGPHHQGDVVGRRPRGRPAGSKNKPKPPVIITRESANTLRAHILEVANGCDVFESVASYARRRQRGICILSGSGTVTNVSLRQPASAGAVVTLHGRFEILSLTGSFLPPPAPPGATSLSIYLAGGQGQVVGGSVVGELIAAGPVIVMAASFTNVAYERLPLEEEEEQVEISGVAGNNSNPFPDPSSGLPFFNLPMNVQLPVDAWAGNSTTRSPF

>Gm11g34250.1

MANRWWTGNVGMIREQELMENSNNNNATTTPTNSSNSNTNANTNTTEEEVSRDNGEDLQNQNLGSHEGSEPGSSGRRPRGRPPGSKNKPKPPIVITKESPNALRSHVLEITSGSDVAESIAAFANRRHRGVSVLSGSGIVANVTLRQPAAPAGVITLHGRFEILSLSGAFLPSPSPPGATGLTVYLAGGQGQVVGGTVAGSLVASGPVMVIAATFANATYERLPLEDEQGEEGMQVQQQQQQQQQQQSQGLGEQVSMPMYNLPPNLLHNGQNMPHDVLWGAPPRPPPSF

>Gm14g03240.1

MFGEMDRKQEQDKGNSNMSGIDVALISPKVPKAVSPVSSAAAEGDTLRRPRGRPAGSKNKPKPPIIVTRDSANALKAHAMEVSSGCDVNESLLNFARRKQRGLYILNGTGCVTNVTLRQPGSAGAIVTLHGRFEILSLLGSILPPPAPPGITGLTIYLAGAQGQVVGGAVVGALIASGPLVIMAASFMHATFDRLPLEDDELAAAMQNQHYQNGRTHHLDISDLYAIPQNLLMNGTMPPEIYSWAPGRNFSKT

>Gm14g07250.1

MANRWWAGSATVKPSEQDNGDNNGTPTNSGNSNTKVNAVLEDDANTNERDGEEQTLSGGRRPRGRPPGSKNKPKPPVVITKESPNALCSHILEISDGSDVAECIAIFATRRHRGVSVLSGNGFVTNVTLRQPAAPGGVITLQGRFEILSLSGAFLPAPSPPEATGLTVYLAGGQGQLLPFSLMLLITGCLCRRNLEEDMQEVNEGDGGGTSPPQPPPPPPPRSQGEQQVSMPVYNLVPNNGDVFWGPPSSPSNY

>Gm14g35980.1

MFSKLHSQHSQQPQPFQFSRECQSSKEDDIPSTCGLNLITAQKSGLSGDQNNETTSEIMRRPRGRPSGSKNRPKPPLIITCEPEPVMSPFILEIPGGSGVVEALARFSRRKNTGLCVLTGSGTVANVTLRQPSFTPAGASVATVTFHGRFNILSMSATFLHHGSPAAIPNALAVSLSGPQGQIVGGLVAGRLLAAGTVFVIAASFNNPSYHRLSSEEDAQNTSDGGGDRQSPAESSMYSCHRPSDVIWAPTPRPPF

>Gm02g37680.1

MFSKLQPQHQQQHQPFQFSRECQTSEDDDSRSSGGPNTAVAQKSVLGGGGCSDGATIEVVRRPRGRPPGSKNRPKPPLIITREPEPAMSPFILEIPGGSDVVEALARFSRRKNTGLCVLTGSGTVANVTLRQPSFSPAGATVATVTFHGRFDILSMSATFLHHASPAAIPNAFAVSLSGPQGQIVGGFVAGRLLAAGTVFVIAASFNNPSYHRLSSEEEAQNNSGGGAGDAQSPPVSGGGLESGHVPAESFMYSCHLPSDVIWAPTPRPPF

>Gm09g38120.1

MAGIDLGSASHFVHHRLQRPDLEDDENQQDQDNNLNDHEGLDLITPNPGPGDVVGRRPRGRPPGSKNKPKPPVIITRESANTLRAHILEVSTGCDVFESVATYARKRQRGICVLSGSGTVTNVTLRQPAAAGAVVTLHGRFEILSLSGSFLPPPAPPGATSLTVFLGGGQGQVVGGNVVGPLVASGPVIVIASSFTNVAYERLPLDEDESMQMQQGQSSAGDGSGDHGGGVSNNSFPDPSSGLPFFNLPLNMPQLPVDGWAGNSGGRQSY

>Gm04g09710.1

MKGEYVEQQQQHPKSETPPSMFSKLQPQHHPFPHHPFQLSAEDATTITPSTAQKANSSGGDGATIEVVRRPRGRPPGSKNKPKPPVIITRDPEPAMSPYILEVSGGNDVVEAIAQFSHRKNMGICVLTGSGTVANVTLRQPSTTPGTTVTFHGRFDILSVSATFLPQQSGASPAVPNGFAISLAGPQGQIVGGLVAGGLMAAGTVFVIAASFNNPAYHRLPPEEEGASAGDGHSPPVSGGGDSGHGQAESCGMSMYSCHLPSDVIWAPTARPPPPPPPPY

>Gm06g01650.1

MTPAALHLQPQSDDDDGEGPFSTQRRPRGRPMGSKNKPKPPVIVTRDSPNVLRSHVLEVSSGADVVESLSNYARRRGRGVSVLSGSGTVANVVLRQPAGSVLTLHGRFEIVSMTGTVLPPPAPPGSDGLSVYLSGAQGQVVGGVVVAPLVASSHVVLVAASFANAMFERLPLPLNQHDDDDQGEVFGWGGTGTTSSTSTAPPKTHPF

>Gm20g05300.1

MDSSSQHFPPPTLTTANQPLMAPPTKNPRGRPRGSKNKPKSTSLLSQPVEPSVKLVTINVAPGMDVIETILDVARRDHVSLIILNASGMIKNATVCDSPQGVPTLLIGPFSLFSLTGFYLYNNQYALHPGATPPPPFSFGIKLCASHGQVFNGLIGGSVIAGDNSYVSACVLRLPCMVVFRILIEYFVGVIGGNEGSGCLLFFIFGPWWFSEFSLSAWRCRWLKL

>Gm20g07760.1

MPRGRPSGSKNKPKTTSLLVAQPVEPSMKLVIINVDRGKDIMQTILNVAHQGCVSLTVLSASGTVTSVTLCNSPNDGGGALMLHGPFTLLSINGSYFYNNNQYNLHSGATRSPPVSFGIHLSTSKGKILGGAIGGNVIAGDDVSITLSTFSHPEIYMYVPKDEEEDNDEKKQ

>Gm17g14560.1

MDPISAHGHSLPPPFHTRDLHLHHQQQQQQHQFQSLNQATEDENSGSSGAQKREREENSNGNNEGAGEAEITRRPRGRPAGSKNKPKPPIIITRDSANAMRTHMMEVADGYDIVESVSEFARKRQRGICIMSGTGTVTNVTLRQPASSGSVVTLHGRFEILSLSGSFLPPPAPPAASGLTIYLAGGQGQVVGGSVVGTLVASGPVVIMAASFSNAAYERLPLEDEDPNSLQMQGGSIGSPGASGVGQSQLLGGDATAPLFHGLPPNLLNSVQMPSEPFWAASTRPPF

>Gm07g35820.1

MTTNSMAISISQNSFSSDLDSTSSWDYLTGSSSQFPRCPPSPIANQPLENLPIATPPTKKPRGRPPGSKNKPKTTSFPVGQPAEPSMKLVIVNVTPGSDIIESILDVARRGHVSLTILSASGTISKVTLHNSIHGVAALTLRGPFTLLSLNGSYLHNNHYTLHPGATPPPPLSFGISFSTSQGQVFGGAIGGRVIAGDDVSLTISTFKNPVMYKYVPTDKERNGDDNNNNHYNNISKNFNGGNELLGFNMVGCRVRGW

>Gm20g07960.1

MTNNSFAISHSQNFSFDSRSTFFWDNHTGSSSQCPHHPPPLAGTNQPQENLLLPTPHPRKPRGRPPGSKNKQKIISFPVAQPSEPFVRIVIINVDPGRDIMESILDVARQGHVNLTVLSTSGTVTKVTLQNSLHGAAALTLHGPFTLLSLNGSYLINNHHNHNSGATLPPPSSFGIHLSTSGGQAIGGAIGGQVIAGDNVKITVSTFWNPEMYKYIPEGNKGGNDDNNDRENNYNNNPIDCNGGWKPIGVQYG

>Mt5g011670.1

MDQITSHGHSLPPPFHTARDLHLHHQHQQQQQQQQHHQFHTLQQQQQTTDQDEQSGSSSGGGLNLTNREENSNNKFSTDFSPKLESGGGGSGGDTDSMTRRPRGRPAGSKNKPKPPIIITRDSANALKTHVMEVADGCDVVESVNNFARRRQRGVCIMSGTGTVTNVTLRQPASPGAVVTLHGRFEILSLAGSFLPPPAPPAASGLTIYLAGGQGQVVGGSVVGALIASGPVVIMSASFSNAAYERLPLEDDDGSSIQQLQGGGGGGSPSGGGGGVQQQQQQLLGDSTAPLFQAMHPPNSNPNLNPNLLGQMPSDNFWPTGRSPY

>Mt5g075040.1

MFSNFQQQQHHNLFQPSRECQTSEEDETRSSGGPSPNKPSCNDGATIEIGRRPRGRPPGSKNKPKAQSQIIINHSSDPAMSPHILEIPEGSDVVEAISRFSNRRKTGLCVLTGSGTVANVTLRQPSGPPGTTVTFHGRFNILSISATFFSPLESSPPMNKEFSISLAAPQGQIVGGFVVGPLLAAGTVFVIAASFNNPSYHRLPLEEDVRNNSVSGGYEEKSPPQLSGGESCMYSSQLPSDVIWAPTARTNF

>Mt5g087880.1

MSNRWWSGRQTEANPVEGENRNGPTRIILRRESRRASNGSVNGNVTGGNVTPTRSNTSNTGTGNSNGHVNDELENSNGRSGDQTARSGRRPRGRPPGSKNKPKPPLMITKETPNALSSVILEVANGADIAHSISSYANRRHRGVSVLSGTGYVTNVTLRQDNAPGGMISLQGRCHILSLSGAFLPPPSPPDATGLTVYLAGGQGQVVGGLVIGSLIASGPVMVVAATFANATYERLPLEDEDEGDEENFQEVDNINLVVNNGNHVANGDGGSGSLSGSASGSGGGGGGGATSHGLGEYSFNPSMIQNGNDSGHGHGHDVFWRPHPPPY

>Mt5g096170.1

MAPSSLSPNSLENNLVRVEGGNMVSHITTTNNNNNDTLMPTPIPSHLPQKKPRGRPPGSKNKPKPPVNIEENMDNNMKMIYIEIPSGKDIVGEIINCAHRYQASITVSRGYGLVTNVTLLNPKTHFPTPPMIGPFEMTSLLGTYVNINCRRNTLNHPPCSCFSILLSGHGAVVYGGTVGGTIIAASNVWIQATLCKNLDHYQSISNNNNNHDNNVVNLSTFDDVATFPNH

>Mt5g096750.1

MAEKEFAPFSQSSNSKNLEFLENKSERFYAPSGSSSAIVGEIIGHSTSLPNTTTTNNNNEQLVMPTSPPRAPSSKRSRGRPKGSKNKPKTPAVVMVEPQTLMKQIFIEIPAGYDVLESIIKMAWRHEADITVLRGFGIVSDITIHSSLSHTPPLTIEGPVQMTSLSGTYVNPNVDNVPSEVIANPACSSFSIFLSGSHGQVYGGIVVGKVMTSSVVMISATLMKKTKFYMVA

>Mt5g096650.1

MAAEKELAPFSQSSNTENEFSQNNYVRFSTSLLNTTTNIGTENLVMPKSPPGGAPSSGMLIDLPQNSREDLITTSSKRSRGRSKGSKNKPKPPVVITVEPESFMKQIFIEISAGCDVVESIIKMAWRHQADISVMRGSGLVSNITIRNSTSHSPALTIEGPIKMMSLSGTYINPNSDTVPSEFITNPNHSSFSIFLSGNGNEGQVYGGIVIGKIMASGNVMITATLQKKPKFYRVT

>Mt8g102710.1

MQNIHRQNLHLQQQHHHQNQHDSEEQESNRASVGGGAPFSSNEEDDRSQGLELGSAAGPGDVVGRRPRGRPPGSKNKAKPPVIITRESANTLRAHILEVAGGSDVFECVSTYARRRQRGICVLSGSGTVTNVSIRQPAAAGGVVTLHGRFEILSLSGSFLPPPAPPGATSLTIYLAGGQGQVVGGSVVGELIAAGPVIVIAASFTNVAYEKLPLEEEQLQMQAEAGGGSQGSGGGGGGNNNNNPFPDPSSGLPFFNLPLNMQNVHQLPVDGWAGNNNNSASRQPF

>Mt8g102740.1

MTVLLLRRRLGRRNVAGRGNTRLMVTLLLVLAPVSSPVAATSAASAGDSGNADAPPKKHRGRPPGSGKKQLDALGAGGTGFTPHVILVESGEDITEKVMAFSQTGPRTVCILSAIGAISSVILRQPASGSIARYEVQLVNGQFEIVSLSGPMPLSENNGEQSRTSSLYVSVAGADGRVLGGAVAGELTAASTVQVIVGSFIVDRKKSSSSMVKSGPSSAPTSQMLNFGAPTTPTSPTSQGPSTESSEENDHNSNFSRGPGLYNNANQPVHNNMQQMYHHPLWAGQTHP

>Mt7g092530.1

MNTFLVVRMVASSTTLFVSLSLSFPFQSHPIPFSLQYSIDPFVFLFYMAGIDLGSASQHFVHRLQRPDLEVHDESQDQDGNNNNNHEGLDLVSPNHGLGDVVGRRPRGRPPGSKNKPKPPVIITRESANTLRAHILEVSSGCDVFDSVATYARKRQRGICVLSGSGTVTNVTLRQPAAAGSVVTLHGRFEILSLSGSFLPPPAPPGATSLSVFLGGGQGQVVGGNVVGPLVASGPVIVIASSFTNVAYERLPLDEDESLQMQQGQSSAGGGGGGGDGVNNSFPDPSSGLPFFNLPLNMPQLPVDGWAGNSGGRQSY

>Mt1g093390.1

MANRWWTGPVGLGGMDNSVTSSPLGKPDLGFSMNQSAVTGVNNMNNNNNEEEEDEKENSDEHKGGAIETNTSTRRPRGRPSGSKNKPKPPIFITRDSPNALRSHVMEVATGTDISDSIVQFARKRQRGICILSASGTVVNVSLRQPTGPGAVVALPGRFDILSLTGSVLPGPSPPGATGLTIYLSGGQGQVVGGGVVGPLVAAGPVMLMAATFSNATYERLPVEDGDDQEGHQGGGGDDESPTRAAGMGQLAIGSVGEGSSIPPGYNNVGGNLGVSNGGQQQLLNNHEAYNNSPWGHASHGRPPY

>Mt1g046720.1

MDSHEPQQPHPHPHPDHLQLENVVLVDPNPFTNTVLTTMMEPITARFPQLHMNTNQPPHSEPLNNNIPSTLKPCVTASSGSGSIHKKKGRPRKYFPDGNIALVSSPALDATITSHSSSIANKSTRGRGRPRGSLNKKKKVEVSGVSGTGFSQHVITVNPGETLMMLRRWLLMYVEMDIVMKLKTFCQGGPNTDMCILSAHGLVGTVALHQSGTIVLREGRFEILSLSGMLEEFDNKNGFKTMGYFKVSLVDPNLNVLGGVVADKLIAASFVKVIVGSFTLDGKNCSSSNLKLGSSSMTISQFAAPRTPTSAAASQGPSSMSYGNNENIPFDQVLGIYNNDSEPIPTLSMYQQIDSPNSK

>Mt3g149230.1

MKGEYVEQHSSNPNKGNETPPPSLLFSKPFHPFQLSHHECQPIGEDDNNNTSGGVATTQKPNTSGDGATIEVSRRPRGRPPGSKNKPKPPIIITRDPETVMSPFILDISGGNDVVEAISEFSRRKNIGLCVLTGSGTVANVTLRQPSTTPGTTVTFHGRFDILSITATFVPQQHGVSPAIPSNFSISLAGPQGQIVGGIVAGNLIAAGTVFVIASSFNNPSYHRLPLEEDEGGNSVSGGGEGNSQNVSGAVDSGQGQGGGGESCGMSMYNCHLPSSDVIWAPSARPPPPY

>MtCU179634_7.1

MDFNHYSLGLMRGESDSDAGASSGGGAPNRRPRGRPPGSKNKPKPPIIVTRDTPNALRSHVLEVSTDVDIMESISNYARRRGRGVCILSGSGTVTNVNLRQPAASVVTLHGRFEILSLSGTVLPPPAPPASSGISIFLSGGQGQVVGGSVVGPLIASGPVVLMAASFANAVFERLPLEEDDEAPANVPTTQVQPAASQSSGVTGGGEAAGTAHPAEGNMAFGNNYSFSAELLGWGGNAANERPPF

>Os02g25020.1

MDPVTASIHGHHLPPPFNTRDFHHHLQQQQHQLHLKTEDDQGGGTPGVFGSRGTKRDHDDDENSGNGHGSGGDGGDLALVPPSGGGPDGAGSESATRRPRGRPAGSKNKPKPPIIITRDSANTLRTHVMEVAGGCDISESITTFARRRQRGVCVLSGAGTVTNVTLRQPASQGAVVALHGRFEILSLSGSFLPPPAPPEATGLTVYLAGGQGQVVGGSVVGALTAAGPVVIMAASFANAVYERLPLEDDELLAAQGQADSAGLLAAGQQAAQLAGGAVDPSLFQGLPPNLLGNVQLPPEAAYGWNPGAGGGRPAPF

>Os02g48320.3

MAGMDPGGGGAGAGSSRYFHHLLRPQQPSPLSPLSPTSHVKMEHSKMSPDKSPVGEGDHAGGSGSGGVGGDHQPSSSAMVPVEGGSGSAGGSGSGGPTRRPRGRPPGSKNKPKPPIIVTRDSPNALHSHVLEVAGGADVVDCVAEYARRRGRGVCVLSGGGAVVNVALRQPGASPPGSMVATLRGRFEILSLTGTVLPPPAPPGASGLTVFLSGGQGQVIGGSVVGPLVAAGPVVLMAAS

>Os02g57520.1

MGLPEQPSGSSGPKAELPVAKEPEASPTGGAAADHADENNESGGGEPREGAVVAAPNRRPRGRPPGSKNKPKPPIFVTRDSPNALRSHVMEVAGGADVADAIAQFSRRRQRGVCVLSGAGTVANVALRQPSAPGAVVALHGRFEILSLTGTFLPGPAPPGSTGLTVYLAGGQGQVVGGSVVGSLIAAGPVMVIASTFANATYERLPLEEEEEGSGPPMPGGAEPLMAGGHGIADPSALPMFNLPPSNGLGGGGDGFPWAAHPRPPY

>Os06g04540.1

MDPVTAAAAHGGGHHHHHHFGAPPVAAFHHHPFHHGGGAHYPAAFQQFQEEQQQLVAAAAAAGGMAKQELVDESNNTINSGGSNGSGGEEQRQQSGEEQHQQGAAAPVVIRRPRGRPAGSKNKPKPPVIITRDSASALRAHVLEVASGCDLVDSVATFARRRQVGVCVLSATGAVTNVSVRQPGAGPGAVVNLTGRFDILSLSGSFLPPPAPPSATGLTVYVSGGQGQVVGGTVAGPLIAVGPVVIMAASFGNAAYERLPLEDDEPPQHMAGGGQSSPPPPPLPLPPHQQPILQDHLPHNLMNGIHLPGDAAYGWTSGGGGGGRAAPY

>Os06g22100.2

MAGMDPTGGGGGGGVAAHYLHMLRAQQHQPLSPAGDVKAERSMLSPDESPGADADLGSDHPTSSAMVAAEDSGGGSGSGGPMRRPRGRPLGSKNKPKPPIIVTRDSPNAFHSHVLEVAAGTDIVECVCEFARRRGRGVSVLSGGGAVANVALRQPGASPPGSLVATMRGQFEILSLTGTVLPPPAPPSASGLTVFLSGGQGQVVGGSVAGQLIAAGPVFLMAASFANAVYERLPLDGEDPEAEAAAATPPGDAAQPTGPPPPQQQPTASQSSEVTAGDGGGGGGLGMYLGGHVGSYQQQQQQLPGPGDNFGSWSGSIRPPPF

>Os06g41860.1

MAASNNKWWQAALDFPPPPPPVNVPAAAPAGAASPESKQQAAAGAIVPLRRPRGRPLGSKNKPKPPVIITRDSPDALHSHIIEVAPGADVAACVAEYARRRGRGVCLMGASGAVADVAVRGAAAPLPGRFELLSVTGTVLPPPAPPGASGLSVLLSAGQGQVVGGCVVGPLVAAGPVTLFAATFANAVYERLPLADAADVADVKPDLSSAAAAATSTSAPQEVQQQQLPLPPSSHHPQAMPATYPDHRSPPYAWAGGV

>Os04g50030.1

MAGLDLGTAATRYVHQLHHLHPDLQLQHSYAKQHEPSDDDPNGSGGGGNSNGGPYGDHDGGSSSSGPATDGAVGGPGDVVARRPRGRPPGSKNKPKPPVIITRESANTLRAHILEVGSGCDVFECVSTYARRRQRGVCVLSGSGVVTNVTLRQPSAPAGAVVSLHGRFEILSLSGSFLPPPAPPGATSLTIFLAGGQGQVVGGNVVGALYAAGPVIVIAASFANVAYERLPLEEEEAPPPQAGLQMQQPGGGADAGGMGGAFPPDPSAAGLPFFNLPLNNMPGGGGSQLPPGADGHGWAGARPPF

>Os08g06320.1

MAGLDLGTSYLHHHQSLHLRHDDGGAGSDDGGHDDLSPGSGGGGGPSSTAGGAGIGGGEVVARRPRGRPPGSKNKPKPPVIITRESANALRAHILEVAAGCDVFEALTAYARRRQRGVCVLSAAGTVANVTLRQPQSAQPGPASPAVATLHGRFEILSLAGSFLPPPAPPGATSLAAFLAGGQGQVVGGSVAGALIAAGPVVVVAASFSNVAYERLPLEDGDEVVPPAPAGSDQGGGGSGGMPPLGVDPSGGAATGGLPFFNMPFGMPPMPVDGHAGWPGAGVGRPPFS

>Os08g37345.1

MERRSQLGGGEGDGKLVVGGGAMGEKKQQLECFSDEVDSRDGGGGAAEETTAGGGGEGVAAVVVVGKRRRGRPPGSKNKPKPPVVVTREAAAAEPAAAAAMRSHVLEIPGGGDVAGALAGYARRRGLGICVLAGTGAVANVSLRHPLPSGAAAEIGGGAAAAVVVFHGRYEILSISATFLPPAMAAAAPRAALGGLSISLAGPHGQIFGGAVAGPLVAATTVVVVAAAFASPTFHRLPAEYDDAPAPVSGSGADADEHRGRRRTEPPEHHHLTPLHPRGIALATATTTTTTQPVYASACQHEEVWPPAAAAAAASAPRPRPPYQ

>Os08g44910.1

MASKEPSGDHDHEMNGTSAGGGEPKDGAVVTGRNRRPRGRPPGSKNKPKPPIFVTRDSPNALRSHVMEVAGGADVAESIAHFARRRQRGVCVLSGAGTVTDVALRQPAAPSAVVALRGRFEILSLTGTFLPGPAPPGSTGLTVYLAGGQGQVVGGSVVGTLTAAGPVMVIASTFANATYERLPLDQEEEEAAAGGMMAPPPLMAGAADPLLFGGGMHDAGLAAWHHARPPPPPPY

>Os03g16350.1

MGSIDGHSLQQHQGYSHGGGAGGSNEEEEASPPPGGGSATGSAGRRPRGRPPGSKNKPKPPVVVTRESPNAMRSHVLEIASGADIVEAIAGFSRRRQRGVSVLSGSGAVTNVTLRQPAGTGAAAVALRGRFEILSMSGAFLPAPAPPGATGLAVYLAGGQGQVVGGSVMGELIASGPVMVIAATFGNATYERLPLDQEGEEGAVLSGSEGAAAQMEQQSSGGAVVPPPMYAAVQQTPPHDMFGQWGHAAVARPPPTSF

>Os09g28930.1

MSFCERDMNKESMYQERDDMAGIRFATPPLPQQQQQQQLVECFSDEVDSRGSGGEMKDAVGSGSGQLVVVGGGDGASIEVAKKRRGRPPGSKNKPKPPVVITREAEPAAAMRPHVIEIPGGRDVAEALARFSSRRNLGICVLAGTGAVANVSLRHPSPGVPGSAPAAIVFHGRYEILSLSATFLPPAMSSVAPQAAVAAAGLSISLAGPHGQIVGGAVAGPLYAATTVVVVAAAFTNPTFHRLPADDDASVSVSVSLSGSGDADEHRGHQHKPEPQEPRQLRRPPPHLSAAAAVSAAQPVEPCGAPMYACHPQPQEVMWPPPARTPHPPPPPPY

>Os01g72450.1

MADEGSSRAELIEASPAPALDLPSPPRKPRGRPLGSKNKPKPPVVVTRESEAAMRPVVLELGAGCEVAAAVAAFARRRRVGVSVLCGRGTVAAVTLRLPTSPPAAVKLHGRFEVLSLSGTVLPSAAGEGAAPPPPFSVSLAGAGGQVIGGTLAGEMTTADGLVVVAATFGSAEVHRLPADEDDEATGSRGGEERRHPQQQPPQTVAATSAVDVGLLGYGGGVGVAGGASGGQVGRHQQQQQQAEMVLWAQSPGSVGPAHPATSRY

>Pp1s7_185V6.1

MAGVEEVGARPSGAPASEDEQSGSSGRGRKRGKQSGGNNQLAVLEPGRKPRGRPPGSKNKPKPPIIIMRENGQAMRPHILEVAGGCDVSDSVASFSRRRQRGVCVMGASGTVSNVTLRQPTTAGATITFHGRFEIISLSGAFLPHPSSQPTTGLTVSLAGAAGQVLGGSVVGTLMAAGPVVVIAASFMGPTFVRLPLDADDEGPSGGLTIVGGSSNQPPPLAIQAPELNYPPGLQSLYNIVTPQNPNLNNPQQQQQLSTQDVLSHWASSGGGGSGGHQHGHGHQRGHYS

>Pp1s45_204V6.1

MAGVEGGAEMGSRPSPPGGNSSDEEQSGSSVPGRPRKRSKGKSKPPPGPGEPSRKPRGRPPGSKNKPKPPVIITRENGNAMRPHILEVASGHDVWESVADFARRRQRGICVMGGSGTVTNVTLRQSTTPGATVTIHGRFEIISLSGSYLPPPSPTPPAGLTTGLTISFAGASGQVLGGCVVGALMAASPVLVVATSFTGATYDRLPLAEDEPPMLQVTVASAAPTMQSPDLSSYNPSTALQNLYNIVPQSNPNLNSQQAAQLATPQDVINPWATGSGHTQRPQPY

>Pp1s79_150V6.1

MAGLEDVGARPSGAPSDDEQSGSSGRGRKRGKQPAENQLAILEPGRKPRGRPPGSKNKPKPPVIITRENGNAMRPHILEVAGGCDVSDSVASFSRRRQRGVCVMGASGTVSNVTLRQPTTPGATVTFHGRFEIISLSGAFLPHPSSAPTTGLTVSLAGAAGQVLGGSVVGTLMAAGPVLVIAASFIGPTFERLPLDNDDEGLTMGGGSSSQQPPLAIQAPELNYPAGLQSLYSIVTPQNPNLSNQPQQLSTQDVLSQWAGGGGGGHPHHPHAGHAGHVHHQRPPQYS

>Pp1s485_11V6.1

MTQIDAPWGIVRARYEKPWEDKHKQAAPENFGHVCMAGVEGGTEAGSRPSPQGGNSSDEDQSGSSVPGRPRKRSKGKSRPPPGPGEPSRKPRGRPPGSKNKPKPPVIITRENGNAMRPHVLEVASGHDVWESVTDFARRRQRGVCVMGGSGTVTNVTLRQPTTPGATVTIHGRFEIISLSGSYLPPPAPSPPTGLTISFAGASGQVLGGCVAGALTAASPVLVIATSFTGATYDRLPLADDEPPIMQVTTASGAQAMQAPDLSSYNPSTALQNLYNIVPQTNPNLASQQAAQDVLNQWASGSGHTQRPQQY

>Pp1s58_127V6.2

MAGVEEVGARPSGAPSDDEQSGSSGRGRKLGKLPTGNQLAILEPGRKPRGRPPGSKNKPKPPVIITRENGNAMRPHILEVAGGCDVGDSVASFSRRRQRGICVMGASGTVSNVTLRQPTTPGATVTFHGRFEIISLSGAFLPHPSSAPTTGLTVSLAGAAGQVLGGSVVGTLMAAGPVLVIAASFIGLTYERLPLDNDDDGLTMGPGSSTQQNPLAIQAPELNYPAGLQSLYSIVTPQNPNLNNQAQQLSTQDVLGHWAGGGGSSHPHHSHGGHGGHGHHQRPSQYS

>Pp1s28_226V6.1

MAGLEDVGQRPSSNPASDDEQSGSSGGGRKRGKQPALSNQLAVLESARKPRGRPPGSKNKPKPPIIITRENGQAMRPHILEIAGGCDVGDSVASFSRRRQRGVHVLGASGIVSNVTLRQPTTPGATVTFHGRFEIISLSGAFLPHLTSQPTTGLTVTLAGAAGQVLGGSVVGTLMAAGPVLVIAASFLGPTYERLPLDPDDEGPSGGLIIGGGSSNQPPPLAIQAPGLNYPGGLQSLYNIVTAQNPNLSNQHQQLSTQDVLSQWAPSGGGGGSGQQHGHSHHQRGQYS

>Pt05s17850.1

MNMKGEYVERHQAKHENTPNMFSELNPHHQHLPFSQHFQLSRESEEEDTRSTGAATTPNPIPTSQKLNELNSSGGTDGATIEVVRRPRGRPPGSKNKPKPPVIITREPEPAMSPYILEVPGGNDVVEALSRFCRRKNMGICVLTGTGTVANVTLRQPSTTPGSTITFHGRFDILSISATFLPQTTSYPLPNSFTISLAGPQGQIVGGIVAGGLVAAGTVFVVAASFNNPSYHRLQVEEEGRNSGSGGGGGEGRSPVSGAGGGESGHAASGGGGGGESCGMAMYSCHLPANDVIWAPSARQPPPSPY

>Pt05s27850.1

MAGFEGNNSRYVHGQNHNNLLRPELHLIQRPSSIPSSDSRDNNNTPSPPDHANQTAHHHPDSSATTSSGGGTNPNRRPRGRPAGSKNKPKPPIIVTRDSPNALRSHVIEISNGADIVESVSTYARKRGRGVCVLSGSGTVANVTLRQPASPAGSVLTLHGRFEILSLSGTVLPPPAPPGAGGLSIFLSGGQGQVVGGNVVGPLMAAGPVVLMAASFANAVFERLPLDDQEEAGAVQVQPTASQSSGVTGSGGQMGDGGGGSGTGGAGSGFFNMAGGAHHGNYPFSGDLFGPWGGSAARPPF

>Pt05s22440.1

MAGDSERIMERNQESEKGHDRRPNMEAILVASKLPKAVPPISSARGGETLRRPRGRPAGSKNKPKPPIIVTRDSANALRAHAMEVSSGCDVCESLANFARRKQRGISVLSGSGCVTNVTLRQPASSGAIVTLHGRFEILSLLGSVLPPPAPQGITGLTIYLAGAQGQVVGGVVVGALIASGPVVIMAASFMNASFDRLPLDEDGITAAVQNQHYQNGRRHHLDIPDLYGMPQKLLTNGAVTPEIYSWAPARTMSKS

>Pt02s00560.1

MAGYESTSTGNNSRYLHHNHNLLRPELHLIQRPSTIPSSDSKENNTPSPDHAKPIATSDHHPDRTTSGTSSGGGGTNPSSRPRGRPAGSKNKPKPPIIVTRDSPNALRSHVLEVSSGADIVESVSNYARKRGIGVCVLSGSGSVANVTLRQPASPAGSVLTLHGRFEILSLSGTVLPPPAPPGAGGLSIFLSGGQGQVVGGNVVGLLMAAGPVVLMAASFANAVFERLPLDDQEEAGAVQVQPTASQNSGVTGSGGQMGDGGGGSSTGGGGFFPMGGAHHGTYPFSADLFGSWGGNASRPPF

>Pt02s15030.1

MDPVAAHGRPLPPPFHTRDFHLHQFQHQQQQNSEDEQSGNGNLNRGQKREHAEIATNNNNTAEGKELVPSSAGGEGEITRRPRGRPAGSKNKPKPPIIITRDSPNALRSHVMEIATGCDIMESVSTFARRRQRGVCILSATGTVTNVTLKQPASPGAVVTLHGRFEILSLSGSFLPPPAPPAASGLTIYLAGGQGQVVGGSVVGPLLASGPVVIMAASFGNAAYERLPLEEDESQTPVPGTGPLGSPGVSSIGQQNQQQHQLMQDPNTSLFQGLPQNLLNSVQLPSEAYWGTGGRPPY

>Pt02s06030.1

MAGAADLTFPSLGSERIMDRSQGLEKVNNHRPSIEAILMTPKLPKAVPPVSSAPDGETMIRRPRGRPAGSKNKPKPPIIVTRDSANALRAHAMEVSSGCDVCESLANFARRKQRGISVLSGSGCVTNVTLRQPTSSGAIVTLHGRFEILSLLGSVLPPPAPQGITGLTIYLAGAQGQVVGGGVVGALIASGPVVIMAASFMKATFDRLPLDDDEITAAVQNQHYQNGRHHHLDISDLYGMPQNLLTNGAVTPEIYSWTPGRTMSKS

>Pt14s06650.1

MDPVSAHGRPLPPPFHTRDFHLHQFQHHQQQNSEDEQSGNGDLNRGQKREHDEINNNNNTVEGLELVPSSSGGEGEISRRPRGRPAGSKNKPKPPIIITRDSANALRSHVMEIATGSDIMESVSTFARRRQRGVCILSGTGTVTNVTLKQPASPGAVVTLHGRFEILSLSGSFLPPPAPPAASGLTVYLAGGQGQVIGGSVAGPLLASGPVVVMAASFGNAAYERLPLEEDIESQTPMLGSGPLGSPGINNIGQQQQNQQQQQLMQDPKTSLFQGLPQNLLNSVQLPAEAYWGTGGRPPY

>Pt01s08190.1

MDPVTAHGHSLPPPFHTRDFQLHHQQQQQHQFHHQQQQNSEDEQSGSSSGLNKSLKRERDESNNSMGNREGQELITSGDGDGEITRRPRGRPAGSKNKPKPPIIITRDSANALRTHLMEVADGCDIVESVATFARRRQRGVCIMSGTGTVTNVTLRQPASPGAIVTLHGRFEILSLAGSFLPPPAPPAATGLTIYLAGGQGQVVGGSVVGTLTASGPVVIMAASFSNAAYERLPLEEEDPQMPMQGGGMGSPGGVGQQQQQPQQHQVMAEQNAQLFHGLPPNLLNSIQLPAEAYWATGRPPY

>Pt01s02520.1

MAGLDLGTTSRYVHQLHHRPDLHLQHQPDPEDHDSNRAGGGLGGGSGGHFSTDHHHDDGSHQGLDLVAAAANSGQGDLVGRRPRGRPAGSKNKPKPPVIITRESANTLRAHILEVGNGCDVFECVANYARRRQRGICILSGAGTVTNVSIRQPAAAGAIVTLHGRFEILSLSGSFLPPPAPPGATSLTIFLAGGQGQVVGGSVVGELTAAGPVIVIAASFTNVAYERLPLDEDDQLQMQSGGGGGGGAGGGGVGNSPFNESGTPSGGLPFFNLPLNMTANVQLPVDGWGGNSGGRVPF

>Pt10s08500.1

MKRVQRERLRATDHSTILANPWWTGQVGLPGLDSSSNSPSLGKINRELSINETSNRSGGRDEDDDDRDTGDEAKEGAVEVGNRRPRGRPPGSKNKPKPPIFVTRDSPNALRSHVMEIAGGADVAESVAQFARRRQRGVCVLSGSGSVANVTLRQPAAPGAVVALHGRFEILSLTGAFLPGPAPPGSTGLTVYLAGGQGQVVGGSVVGSLIAAGPVMVIAATFANATYERLPLEDDEEAGSGAIGSSGQQAGLPDPSSMPVYLPPNLMQSGAQQLGHDAYAWAHAARPPY

>Pt10s20780.1

MANRWWAGNVAMRGVDPVSSSPSLHLGNLEEDSTPPGLNRLGPRREQDFTDTNTSSPKTTTTATPPSTQNQEEHEDSRDNTNNQESGDHTALETIEPGSGSTSRRPRGRPAGSKNKPKPPIVITKESPNSLHSHVLEISSGSDIVESIATFSHRRHRGVSILSGSGIVNNVTLRQPAAPGGVITLHGRFEILSLSGSFLPAPSPPGATGLTVYLAGGQGQVVGGTVMGELIAAGPVMVIAATFSNATYERLPLEEQEQEGMQLQQQVNSPGTNNGNAAAGGGASSGGGNNSVTQSSQGLGEHVSIPGYNLPPNLLPNGQVPHDMFWCPPPRPPPSY

>Pt03s11680.1

MDPVTAHGHSLPPPFHTRDFQLHHHQQQQQHQFHHQQQQNSEDEQSGSSSGLNKSLKRERDENNNSMGNSEGKELITSGSGEGEITRRPRGRPSGSKNKPKPPIIITRDSANALRTHLMEVADGCDIVESVATFARRRQRGVCIMSGTGTVTNVTLRQPASPGAIVTLHGRFEILSLAGSFLPPPAPPAATGLTIYLAGGQGQVVGGSVVGTLTASGPVVIMAASFSNAAYERLPLEEEDPQMPMQGGEMGSPGAVGQQQQQPQQQQVMAEQNAQLFHGLPPNLLNSIQLPAEAYWATGRPPY

>Pt03s09010.1

MAGLDLGTTSRYVHQLHHRPDLQLQHQPDPEDEDPNRAGDGLGSVGRFSTDHNPDDGLHQGLDLVATAANSGPGDIMARRPRGRPPGSKNKEKPPIIITRESANTLRAHILEVGSGCDVFECVGNYARRRQRGICILSGAGTVTNVSIRQPAAAGSIVTLHGRFEILSLSGSFLPPPAPPGATSLTIFLAGGQGQVVGGSVVGELTAAGPVIVIAASFTNVAYERLPLDEDDQLLMQSGGGGGGGAGGGIGVGNNGPFNEAGAASGGLPFFNLPLNMPSNVQLPVNGWAGNSGGRAPF

>Pt08s05890.1

MANRWWAGNVAMSGGDPVSSIPSLHLRNLEEESTPPGSNRLGPRREQNFIDTNTNSPKTTTTTTATAPSAQNQSQNREEQEDSRDNTNNQESGDHIAHETIEPGSGSTSRRPRGRPAGSKNKPKPPVVITKESPNSLRSHVLEISSGSDIVDSIANFSHRRHHGVSILSGSGIVDNVTLRQPAAPGGVITLHGRFEILSLSGSFLPAPSPPGATRLTVYLAGAQGQVVGGTVMGELVAAGPVMVIAATFSNATYERLPLEEQEQEGMQLQQQVDSSGTNNGNAAAGGGNNSGTQSSQGLGEHGSIPVFNLPPNLLPNGQMPHDVFWGPPPRPPPSY

>Pt1986s00200.1

MSGLEASPGAGAGSRYAAHQLLGPELQLQRDAKTPQPGNFKDDINDPESATTSSSGAGNSSSGRRPRGRPAGSKNKPKPPIIIARDTPNALRSHLLEISPGSDIVESISNYARRRAHGVCILSGSGAVTNVTLRQPGGGGSSAVMTLHGRFEILSLTGTSLPSPAPPEAGGLSISLAGGQGQVVGGRVVGPLMASSLVVLMAASFANAMYDRLPVEEDRESVPAVEVQQQQRPAASQSSGVAGSGGGQVGGGGNNGSSGGGGVPFYNLGVNGMGSYPFAGHGEGDHMFSSAAGLHFK

>Pt13s04170.1

MANRWWTGQVGLPGMDTSTSSSSPMKKPDLGISMSNNNREATESGAGKEDEQEDERENSDEPREGAIDIASRRPRGRPPGSKNKPKPPIFVTRDSPNALKSHVMEIASGSDIAENLACFARKRQRGVCVLSGSGMVTNVTLKQPSASGAVMALHGRFEILSLTGAFLPGPAPPGATGLTIYLAGGQGQVVGGSVVGSLVASGPVMVIAATFSNATYERLPLEDEEEGSGGAQGQLGGGNGSGEGNGGGMGDPATSMPVYQLPNMVPNGQLNHEGYGWAHGRPPY

>Pt09s07460.1

MADHFGGAISLSRELSHTSDDSSSDHSPRSVPTLSATPVTADSPSRLGRPHNNTSPDDHIHHNNIQSVQIQRKPRGRPPGSKNRPKPPIIITKDCESSMKPAILEISAGSDVIETIVNFARRNHAGISVISATGSVANVTLRHPVSHTPSLSLHGPFNLLALFGSVVGSLATNKASCASSPPGSAVHSCSSFGISLAGAQGQVFGGIVAGKVIAATQVVVVAATFLNPTFHRLPGENDEADQETKPSVGGGGGGGGPASESCVSTGMSMAVYGVANPTPVNCQMSPPEIMHWPGPPSRPSY

>Pt02s10520.1

MKGEYVEHHQSKHENTPNMFSKLHPRHHQHLPFSQQYQFSRESEEEDTRSTGAAATPNLTPTTQKQKLNEPNSSGGTDGATIEVVRRPRGRPPGSKNKPKPPVIITRESEPSMSPYILEVPGGNDVVEALSRFCRRKNMGICVLTGSGTVANVTLRQPSATPGATITFHGRFDILSISATFLPQTASYPVPNSFTISLAGPQGQIVGGIVAGSLVAAGTVFVVAASFNNPSYHRLPLEEEGRTSGSDGGGEGQSPAVSGAGGGESGHAASGGGGGESCGIAMYSCHMPNDVIWAPAARPPPPPPY

>Pt04s21840.1

MSGLEASPGAGAGSRYAAHQLLGPELQLQRDAKTPQPGNFKDDINDPESATTSSSGAGNSSSGRRPRGRPAGSKNKPKPPIIIARDTPNALRSHLLEISPGSDIVESISNYARRRAHGVCILSGSGAVTNVTLRQPGGGGSSAVMTLHGRFEILSLTGTSLPSPAPPEAGGLSISLAGGQGQVVGGRVVGPLMASSLVVLMAASFANAMYDRLPVEEDRESVPAVEVQQQQRPAASQSSGVAGSGGGQVGGGGNNGSSGGGGVPFYNLGVNGMGSYPFAGHGEGDHMFSSAAGLHFK

>Rc30.m014241

MAGYNNEQSATGTGSRYVHQLLRPELHLQRPSFPSQPSSDSKDNNISPQSKDHNKFSDSEAAAATSSGSNRRPRGRPAGSKNKPKPPIIVTRDSPNALRSHVLEVSTGSDIMESVSIYARKRGRGVCVLSGNGTVANVTLRQPASPAGSVVTLHGRFEILSLSGTVLPPPAPPGAGGLSIFLSGGQGQVVGGSVVGPLMASGPVVLMAASFANAVFERLPLDEEDGTVPVQSTASQSSGVTGGGGGAGQLGDGGGGGGAGLFNMGGNVANYPFSGDLFGWGVNAARPPF

>Rc30190.m011053

MDPVTAHGHSLPPPFHTRDFQLHHHQQQQQQFSHHHQQQNSEDEQSGSSSGAGLNKSQKRERDEGSEGKELIPSGGSGEITRRPRGRPAGSKNKPKPPIIITRDSANALRTHLMEVADGCDIVESVATFARRRQRGVSIMSGTGTVTNVTLRQPASPGAVVTLHGRFEILSLAGSFLPPPAPPAATGLTIYLAGGQGQVVGGSVVGTLIASGPVVIMAASFSNAAYERLPLEEEDAQLPMQGGAIGSPGAVGQQQQHQQQQQVLGEANAQLFQGLQPNLLNSIQLPAEAYWATGRPPY

>Rc34.m8903

MDPVAAHGRPLPPPFHTRDLHLHPHHQFQHHHQQQQQQQNSEDEQTGNGSINRGQKREHDEITTPEGKELVPTTGGGDGEMTRRPRGRPAGSKNKPKPPIIITRDSANALRSHVMEIANGSDIMESVSTFARRRQRGVCILSGTGTVTNVTLRQPASPGAVVTLHGRFEILSLSGSFLPPPAPPAASGLTIYLAGGQGQVVGGSVVGPLLASGPVVIMAASFGNAAYERLPLEEDDGQVPVPGSGPLDSPGVVGQTQPQQPQQLMQDPNPPLFQGLPPNLLNSVQLPAEAYWGAARPPF

>Rc29603.m000533

MRTSSSSSSPLLSSQSATLANPWWTGQIGLAGLDPASNSPSLNKANREISINDNSNSRGEDDDDRDTGDEPKEGAVEVGTRRPRGRPPGSKNKPKPPIFVTRDSPNALRSHVMEVVGGADVAECVAQFARRRQRGVCVLSGSGSVANVTLRQPAAPGAVVALHGRFEILSLTGAFLPGPAPPGSTGLTVYLAGGQGQVVGGSVVGSLIAAGPVMVIAATFANATYERLPLEDDEEAASAGQGHIQGGSNNSPPPIGSTGQQPGLPDPSALPVYNLPPNLIPNGGQLGHDAYAWAHGRPPYQ

>Rc30143.m001173

MANRWWAGNVAMRGVDPISSASSLHLRNPEEVEAIGLNRLGPRREQQDVIDTNTNTNNNTNSPKTTSTSTTTPTKNQSQDEQEDSRDNNQESEDHNLALETVEPGSGSSSRRPRGRPPGSKNKLKPPIVVTKESPNALRSHVLEISSGTDIVGSISNFAQRRHRGVSILSGSGIVTNVTLRQPAAPGGVITLHGRFEILSLLGSFLPPPSPPGATTLTVYLAGGQGQVVGGTVMGQLVAAGPVMVIAATFTNATFERLPLEEQEQEGMQLQQQVTSGTNNSNTAGGGSSGGGDGNNSGSQSSQAMAEHGSMPVYNLPPNLLPNGQMPHEVFWGPPPRPPPSF

>Rc28644.m000914

MAGGADLAVPPIGSKNIMESNQEPNRGNYRRPGIEAILMSPKLPKSVPPVSSAVEGETIRRPRGRPAGSKNKPKPPIIVTRDSANALRAHAMEVSSGCDVSESLANFARRRQRGICVLSGSGCVTNVTLRQPASSGAIVTLHGRFEILSLLGSILPPPAPPGITGLTIYLAGAQGQVVGGGVVGALIASGPVVIMAASFMNATFDRLPLDEDEIAAAAAAVQSQHYQNSKLHHHHHHHLDISDLFGVPQNLLTNGTLRQEIYSWAPGRSITQMNHDECS

>Rc27.m40

MAGLDLGTTRYVHQLHHRPDLHLQRQLESEDHDSNRPGGGHFSTDHHQQHEDGSQQGLDLVAAATNSGDIVARRPRGRPPGSRNKPKPPVIITRESANTLRAHILEVGNGCDVFECISNYARRRQRGICILSGAGTVTNVSIRQPAAAGAVVTLHGRFEILSLSGSFLPPPAPPGATSLTIFLAGGQGQVVGGSVVGELTAAGPVIVIAASFTNVAYERLPLDEDEQLQMQSGSSGGGGGSGVGNNPFPDGAATSGGLPFFNLPLTMPSNVQLPVDGWAGTNSGSRAPF

>Rc20.m74

MANRWWAGQVGLPGMDTSTSSTSPMKKPDLGISMSNSSHRETTERDHHHQHHHQEIQEEEREHSDEPKEGAIEVATHRRPRGRPAGSKNKPKPPIFVTRDSPNALKSHVMEIANGSDIAESLACFARKKQRGVCVLSGSGMVTNVTLKQPSAPGAVMALHGRFEILSLTGAFLPGPAPPGATGLTIYLAGGQGQVVGGSVVGSLTATGPVMVIAATFSNATYERLPLEEEEEGGSGGGQGQLGGGGGSSEGGGGGSGGIGEPGASAPPGYNLPPNLQVPNGGQLNLDAYGWAHGGRPPY

>Rc29908.m006250

MKGEYVDQQHHHHHHPKHESTNMFSKLHHHHHPLPFSTHFQLSRDSEDDDTRSTGAATTAITTATATATTTTPTRPTEPPNSSGGTDGATIEVVRRPRGRPPGSKNKPKPPVIITRDPEPAMSPYILEVCGGSDVVEAISRFCRRKNIGICVLTGSGTVANVTLRQPSTTPGSTITFHGRFDILSISATFMPQTVSYPVPNTFTISLAGPQGQIVGGLVAGSLIAAGTVYIMAATFNNPSYHRLPVDDEGRNSGSGGGGGEGQSPAVSGAGGGGDSGGGGGDSGGGMVMYSGHLPSDVIWAPTARPPPPY

>Rc27742.m000250

MAHHRKSAISSPDNYQHHLHHHHTAIEIQRKPRGRPPGSKNKPKPPIVITKDSDSAMKPVILEISAGSDIIDSIINFARRNHSGISVISATGSVSNVTLRHPLSHAPSLSLHGPFNILSLSGTFLGSFTPKQSAGSSSVGSPSCCFGISLAGAQGQVFGGIVAGKVLAASQVVVVAATFLNPTFHRLPSDHNDNEVEETEPSLGGPASANESCVSTGMSMTVYAVANPTPINSQMSPPDIMHWPGPPRPHY

>Sb10g002490.1

MDPAVAAAAAHGGGHHFAQPVAPFHHPFGGLGGHHHHLFPGQQHPAFQHFQEQQRQLELLGAGGGVPGGVNKQELGVGGDDSNNTINSAGSNGSGDGAVAGADQQAGGGDGDQQQQQHALVMRRPRGRPAGSKNKPKPPVIITRDSASALRAHVLEVAAGCDVVDSIAGFARRRQVGVCVLSASGSVANVCIRHSGAAPGAVVTMAGCFDILSLSGSFLPPPAPPAATGLTVYLSGGQGQVVGGTVAGPLLASGPVVIVAACFGNAAYERLPLDDDEPPQQQLLQQALPPPQGMAGASSSSPPPPLSLPGQHPLADQLPHSLMNGLPLPADAYAWANPGGSGAGRVAPY

>Sb10g012980.1

MAGMDPGGGGGTASHYLELLRAQQLQHQQPSAPLSPSSHVKMERSAPSPENVDPGGDQPALEGSGGSGGPMRKPRGRPPGSKNKPKPPIIITRDSPNALHSHVLEVAAGADIVECVSEYARRRCRGVCVLSGGGAVSNLALRQPGAEPPGSLVATLRGQFEILSLTGTVLPPPAPPGASSLSVYVAGGQGQVMGGSVVGQLIAAGPVVLMAASFANAVYERLPLEAEEEEAATAAAAAAAATETQGAAEPAEGQPQQQEASQSSGVTGGDGGGGGIGHGMSLYDLGGNAAGYQLPGENFGTWSGGMRPPF

>Sb10g024540.1

MASSKWWEQAALDFPSPQPHQAVPMPPALAPAVASPEGGGKQQQQPQQAGAGAAGAIVPLRRPRGRPLGSKNKPKPPVIITRDSPDALHSHVLEVAPGADVSACVAEYARRRGRGVCVLGASGAVGDVAVRGATAPLRGRFELLSVTGTVLPPPAPPEASGLAVLVSAGQGQVLGGCVVGPLVAAGPVTIFAATFANAVYERLPLADAPELDVKPDLSTATSAGGQDVQQQQQQQPQLPMAPSHQQPPAAMGAGGYADHRSPPYPWGGGV

>Sb07g004070.1

MAGLDLGTSSYLHQQHQHHHQVPLHLHHDDGVGAGGGGSDDGQDSLSPGSGGGGGGTPSSAGGAGIGGGEIVARRPRGRPPGSKNKPKPPVIITRESANALRAHILEVAAGCDVFEALTAYARRRQRGVCVLSAAGTVANVTLRQPQSSQTGPTSPAVATLHGRFEILSLAGSFLPPPAPPGATSLAAFLAGGQGQVVGGSVAGALIAAGPVVVVAASFSNVAYERLPLEDGDEVAPPPPAGGDQQQPGVPFDPAAAAAAGGLPFFNLPMGMPPMPMDGHAGWPAGAPGGGVGRPPFS

>Sb06g026940.1

MAGLDLGTAATRYVHQLHHLHPDLQLQHSYAKQPEPSEDDPHGSGGGGGNSNNGGPYGEHDGGSSSSGPGAGDAPGGSGGNGEMVARRPRGRPPGSKNKPKPPVIITRESANTLRAHILEVGSGCDVFESVSTYARRRQRGVCVLSGSGVVTNVTLRQPSAPTGAVVTLHGRFEILSLSGSFLPPPAPPGATSLTIFLAGGQGQVVGGNVVGALYAAGPVIVIAASFANVAYERLPLEEEEAQAAPPGLQMQPPGGGVDGAGGGMGGGPFPPDPSAAGLPFFNLPLNNMAGGGSQLPPGADGHGWAGARPPF

>Sb04g009050.1

MDPVTASIHGHHLPPPFNTRDFHHHLQQQQQQQHHQLQLKTEDDHGSGGPGVFGSRGNIKGDHHEHNDENSGNSNGSGGGGGGGDELALIPTSGGGGPDGGGDGTPRRPRGRPAGSKNKPKPPIIITRDSANTLRTHVMEVAGGCDISESITAFARRRQRGVCVLSGAGTVTNVTLRQPASQGAVVALHGRFEILSLSGSFLPPPAPPEATGLTVYLAGGQGQVVGGSVVGALTAAGPVVIMAASFANAVYERLPLEEDDMLAAQAQADSAGILAGAQQAAQLAAAGAVDPSLFQGLPPNLLGNVQLPPEAAYGWNPGAAGGRPAPF

>Sb04g030040.1

MPGMDPGGGGSSRYFHQLLRPQQQQQPSPLSPNSHVKMEHHHKMSPDKSPVGGEAEAGGSGGGGDQPSSSALVPVEGGSGGGGGSGSGTPTRRPRGRPPGSKNKPKPPIIVTRDSPNALHSHVLEVAAGADVVDCVAEYARRRGRGVCVLSGGGAVVNVALRQPGASPPGSMVATLRGRFEILSLTGTVLPPPAPPGASGLTVFLSGGQGQVIGGSVVGPLVAAGPVVLMAASFANAVYERLPLEGEEEETAAAAAGAEPQDQVAQSAGPPGQQPTASQSSGVTGGGDAGGGGMSLYNLAGNVGAYQLPGDNFGGWSGGGGGGVRPPF

>Sb01g039840.1

MGSLDGHSLQQGHHHGYAHSHVGAGPDSSGNNNNDEDDASPPPAGAAAGGGGGPRRPRGRPPGSKNKPKPPVVVTRESPNAMRSHVLEIASGADIVDAIAGFSRRRQRGVSVLSGTGAVTNVTLRQPAGAGAAAIALRGRFEILSMSGAFLPAPAPPGATGLAVYLAGGQGQVVGGSVMGELIASGPVMVIAATFGNATYERLPLEQDAEEGAVLSGSSEGGATAQQLEQQQSSGGPVVPPSSMYAVPQTPPHDMFGQWGHAAVTRPPPTSF

>Sb02g026970.1

MSLGKRDMSQERLYQDRKDVPPVHFTTPPPPSHHHQQQLECFSDEVDSRGSAEQKEPASGGAGALVVSGGGGDEASMELSKKRRGRPPGSKNKPKPPVVITREAEPAAAMRPHVIEIPCGCDVADALARFAARRNLGICVLAGTGAVANVSLRHPMSGGVAVGGGGGGAPTTAIVFHGQYEILSISATFLPPAMSAVAPQAAAAAACLSISLAGPHGQVVGGAVVGPLYAASAVVLVAAAFTNPTFHRLPLPPDDDAAVSVSVSLSAGSGGDHAADEHHRGVAHQHQHPGEQPPPQEHHRPLAVRRQAPPHLASASAAQPVEPCGGPPAVPIYTACHPQPQDVIWPPPPPPPPY

>Sb03g046120.1

MAGEEASGGAGLMEPAPAPARALMPVTARKPRGRPLGSKNKPKPPVVVTRESDAAMRPVVLELAAGCDVVSAVAAFARRRRVGVSVLCGRGAVAAVTLRLAAAEDTASAVTLHGRFEVLALSGTVLPSYSPSLAPAFSVSLAGLGGQVIGGTLAGEMTAADGVVVVAAVFRTAEVHRLPAAGAEDGDGDGGREEGRQLQVAASGADAAGLGGYGGGGHVGQHAEQLPEMGLWGQQPTPTRGPVHPLQNRF

>Sb05g002940.1

MQMEGGREGIAVAPGGGGHESGGHGMFRAAAAAADIAMAEAQEEAAVKGYQSSPSTSPTPSPPPPAAAAAGHAGDAAATPLAWSLGGEEKPSGAAGDNNGMQTAGQSGEHASLSSGRRRGRPRGSGRRQILATLGEWYAMSAGGSFTPHVIIVGTGEDVAARIMSFSQKGPRSVCILSANGTISNVTLRQPDASGSTFTYEGRFEILQLMGSFTMAEEGRRRTGGLSVSLAGPDGRVVGGVVAGMLRAASPIQVIVGSFLPNSLKQHQRRMSMQQQPSPVSPALPALVAPPPVLTTATPISQAGPGNGFHAPPPSATPPQPHASAMNLNSTGFTMVGWPASSPQPLAYRASPDINVSLTPQE

>Sb08g002940.1

MGGREGIAVAGGHESGHGLFRADISMTEAQEAAAKGYQFSLSSPSTSPTPSPPPPAAAGDGGDATPVPLAWSLGGDKPSEASGNNGVQTAAQTEHANLSSGRRRGRPRGSGRRQILATLGEWYALTAGGSFTPHVIIVGTGEDVAARIMSFSKKGPRSVCILSANGTISNVTLRQPDPSGSTFTYEGLFEILQLTGSFTMAEEGRKRTGGLSVSLAGPDGRVVGGVVAGMLRAASPIQVIVGSFLPNSLKQHQRRMTLQQQQQQPPAFPAPPAPPAPVAPPPVLTAAMPISQAGFGNGFHAPPPSVVPPQPHASTEHGAMNLNSTTGFTMVGWPASSPPMPHRASPDINVSLTPQE

>Sb07g023325.1

MDREGLYGGRGDGSRLLVVQDLHERPMECFSDEVNSRNGCGDDEEEEEGNGGTGSGSGSRPLPAPNGAGGGGGDGPVSVETGKRRRGRPPGSKNKPKPPPVVTRDVEPAAAMRPHVLEIPSGGDVARALAGFARRRGLGICVLAGTGAVADVSLRHPAASSSADGGGGGAAAAAAAVVVFRGRYEILSISATFLAPSMSAAVPARSAVSRDLSISLAGPHGQIVGGAVVGPLVAATTVVVLAAAFTDLNFHRLPLEDDASAASVSGSGEAGEHRHRGHHGQHQQREHHDASGLHPQTMVAPATTQPVPLYARQSQELWPPAAASAQRPRPPYQ

>Vv01036210001

MANRWWAGQVGLQGVDTSSASPAMKKPDLGISMNENGGSGSGGGGEEEEEKENSDEPREGAIEVATRRPRGRPPGSKNKPKPPIFVTRDSPNALRSHVMEVANGSDITESIAQFARRRQRGVCVLSASGTVMNVTLRQPSAPGGAVMALHGRFEILSLTGAFLPGPAPPGSTGLTIYLAGGQAQVVGGSVVGSLIAAGPVMVIAATFSNATYERLPLEDEEEAGSAAQEQLAGGGGGGMADPSSMPVYNLPPNLLPNGGQLNHDAYGWAHGRQPY

>Vv01019932001

MECWRRQKREMERERWGPIHRPYARKISRRPRGRPAGSKNKPKPPIIITRDSANALRTHVMEIADGCDIVESVATFARRRQRGVCIMSGTGTVTNVTLRQPASPGAIVTLHGRFEILSLSGSFLPPPAPPAATGLTIYLAGGQGQVVGGSVVGQLLASGPVVIMAASFSNAAYERLPLEEEDPALPMPGGSLGSPGGQPQQPQQLLADPNAPLFHGLPPNLLNSIQLPAEAYWATGRPPY

>Vv01016497001

MTHRWWAGNVAMRDPMSSAPSLHLRNTEDDQGGLNRLGPRREQEFMDNNNNNTTTTTNTTATNSNSEQNAGGHEIAEPSSAGRRPRGRPPGSKNKPKPPIVITKESPNALRSHVLEISSGSDIAESIANFAQRRHRGVSVLSASGIVNNVTLRQPAAPGGVITLQGRFEILSLSGAFLPAPSPPGATGLTVYLAGGQGQVVGGSVVGALMASGPVIVIAATFSNATFERLPLEDEPANEGIQMPQTSGVNSGTGGTSAPQSHGLVDPSSMPIYNLPPNLLPNGQMPHDVFWAPPPRPPPY

>Vv01027625001

MDPVTAHGRPLPPPFHTRDLQLHHHHQYQHHPQANSEDEQSGSSSLNRAQKRDRDESNATNNTSPIDGKEFGTSSGDGEITRRPRGRPAGSKNKPKPPIIITRDSANALRSHVMEIATGCDIMDSLNTFARRRQRGICILSGSGTVTNVTLRQPASPGAVVTLHGRFEILSLSGSFLPPPAPPAASGLTIYLAGGQGQVVGGSVVGPLLASGPVVIMAASFGNAAYERLPLEDEEPQVPIPGSGPLGSPGMVGQQPQQQQQQQQLLPDPNASLFQGLPPNLLNSCQLPAEAYWGTARPPY

>Vv01013202001

MIRPLGVQSITFFFSLFFYIFLPQFPRNKLNRKTNRALVFLILFRVFYIFPSFLFSFDVGMAGLDLGTASRYIHQLHRSDLHLQRPQDSDEDNNTNRGGAQYSGDHQDDVAHHGLELVSANAGPGDIVARRPRGRPPGSKNRPKPPVIITRESANTLRAHILEVGNGCDVFDCVATYARRRQRGICILSLSGSFLPPPAPPGATSLTIFLAGGQGQVVGGSVVGELTAAGPVIVIAASFTNVAYERLPLDEEEPLQMQQQSSCCSSSNLLFFIRNGLRIFSLIYSTLLYIQNLIGFHK

>Zm2G173479_T01

MAPSSKDGATEQPTSGGSGDDRENGTGEPKEGAVVTGNRRPRGRPPGSKNKPKPPIFVTRDSPNALRSHVMEVAGGADVAESIAHFARRRQRGVCVLSGAGTVTDVALRQPTAPGAVVALRGRFEILSITGTFLPGPAPPGSTGLTVYLAGGQGQVVGGSVVGTLIAAGPVMVMASTFANATYERLPLDDAEEDPGQAQLPPGPGGGPPLIMGGIADPSAMPMFGGGGGGVPPSLMMPGGAAASGAGLQLGHEGLAWARARPPPY

>Zm2G179639_T01

MPGMDPGGGGSSRYFHQLLRPQQQQPSPLSPNSHVKMEHHRMSPDKSPVVGEADAGGSGAGGDQPSSSAMVPVEGGGSGTGTPTRRPRGRPAGSKNKPKPPIIVTRDSPNALHSHVLEVAAGADVVDCVAEFARRRGRGVCVLSGGGAVANVALRQPGASPPGSMVATLRGRLEILSLTGTVLPPPAPPGASGLTVFLSGGQGQVVGGSVVGPLVAAGPVVLMAASFANAVYERLPLEGEEEEDETAAGAAGAEPQDQVAQSSAGPPGQQPTASQSSGVTGGDAGMSLYNLAGNVGAYQLPGDSLGGWSGGGVRPPF

>Zm2G035077_T01

MDPVTASIHGHYLPPPFNTRDFHHHLQQQQQQHHHQQHLQLKTEDDHGGGAPGLFGDRGTQGDHENNDENSGNSNGSGGGELALVPPSGGGGPEGGDATPRRPRGRPAGSKNKPKPPIIITRDSANTLRTHVMEVAGGCDISESVTAFARRRQRGVCVLSGAGTVTNVTLRQPASQGAVVALHGRFEILSLSGSFLPPPAPPEATGLTVYLAGGQGQVVGGSVVGALTAAGPVVVMAASFANAVYERLPLEDDDLLAAHQAPQAQADGVLAAAQLAAGSVDPTLFQGLPPNLLGDVQMPPEPAYGWNPPGAAGGARPAPF

>Zm2G035432_T01

MAGLDLGTSSYLHHHHHQSLHLHHEDGGGRAGGSDDGQDSLSPGSGGGGGAPPSTIGGGGEVVGRRPRGRPPGSKNKPKPPVIITRESANALRAHILEVAAGCDVFEALTAYARRRQRGVCVLSAAGTVANVTLRQPQSSQAGPASPAVATLHGRFEILSLAGSFLPPPAPPGATSLAAFLAGGQGQVVGGSVAGALIAAGPVVVVAASFSNVAYERLPLEDGDEVAPPPPAGADQQQPGGVPFDPAAAAASAGLPFFNLPLGMPPMPMVGHGGWPAGSPGGAGVGRPPFS

>Zm2G086541_T01

MAGLDLGTAATRYVHQLHHLHPDLQLQHNYAKQPEPSEDDPNGSGGGNSNNGGPYGDHDGGSSSSGPAGDGHGGSGGNGEMVARRPRGRPAGSKNKPKPPVIITRESANTLRAHILEVASGCDVFESVSTYARRRQRGVCVLSGSGEVTNVTLRQPSAPTGAVVTLHGRFEILSLSGSFLPPPAPPGATSLTIFLAGGQGQVVGGNVVGALYAAGPVIVIAASFANVAYERLPLEEEEAAQQAAPPGLQMQPPSGGVDGAGGMGGGPFPPDPSAAGLPFFNLPLNNMTGGGSQIPPGADGHGWAGARPPF

>Zm2G300111_T01

MAGLDLGTAATRYVHQLHHLHPDLQLQHNYAKQPEPSEDDPNGGGGGNSNNGGPYGEHDGGSSSSGPAGDAPGGSGGNGEMVVRRPRGRPPGSKNKPKPPVIITRESANTLRAHILEVASGCDVFESVSTYARRRQRGVCVLSGSGVVTNVTLRQPSAPAGAVVTLHGRFEILSLSGSFLPPPAPPGATSLTIFLAGGQGQVVGGNVVGALYAAGPVIVIAASFANVAYERLPLEEEEAQAAPPGLQMQPPGGGVDGAGGMGGGPFPPDPSAAAGLPFFNLPLNNMSGGGSPLPPGADGHGWAGLREHEDIKKKAMDGTMVKGGLQKVQLIVFELYKGVTCLCVQQQ

>Zm2G405559_T01

MSLGKRDMSQERLYQDRKDVPPIHFTTPPPPPSHHHPQQHGRHGEQQQQLECFSDEVDSRGSAERKEPASGGAPVVPGGGDDGASIELSKKRRGRPPGSKNKPKPPVVVTREAEPAAAMRPHVIEIPCGCDVADALARFAARRNLGICVLAGTGAVANVSLRHPSPGGPAVMFHGQYEVLSISATFLPPAMSAVAPQAAAAAACLSISLAGPHGQIVGGAVAGPLYAASTVVLVAAAFTNPTFHRLPADDDASVSVSVSVSLSAGSGDPADEHRGGHQDRREQPPQEHRPLAVRRQPAPHPASAAQTVEPCGPPDVPIYAACHPQPHDVMWPPPPRAPQMPPPY

>Zm2G028397_T01

MPGMDPGGGGSSRYFHQLLRPQQQQSSPLSPNSHVKMEHHRMSPDKSPVGEAEAGGSGAGADQPSSSAMVPVEGGGSGSGTGGTPTRRPRGRPPGSKNKPKPPIIVTRDSPNALHSHVLEVAAGADVVDCVAEYARRRGRGVCVLSGGGAVVNVALRQPGASPPGSMVATLRGRFEILSLTGTVLPPPAPPGASGLTVFLSGGQGQVIGGSVVGPLVAAGPVVLMAASFANAVYERLPLEGEEEETAAAAAAGAEPQDQVAQSAGPPGQQPTASQSSGVTGGDAAGGGGMSLYNLAGNVGAYQLPGDNFGGWSGGGGGGVRPPF

>Zm2G155699_T01

MKDPEPSSPAVGAPGDHGADETNESGGAVVAAPSRRPRGRPPGSKNKPKPPIFVTRDSPNALRSHVMEVAGGADVADAIAQFSRRRQRGVCVLSGAGTVANVALRQPSAPTAVVALRGRFEILSLTGTFLPGPAPPGSTGLTVYLAGGQGQVVGGSVVGTLIAAGPVMVIASTFANATYERLPLEEEDEGPAPPMASGGGADPLMGGGLGIADPSALPMFNLPPSNGQLGGGDGFPSWAHPRPPY

>Zm2G119168_T01

MGSLDGGHSLQQGRHHGYAHSHSHVGAGPDSSNNNNDEDDASPPPPAGAGGGPRRPRGRPPGSKNKPKPPVVVTRESPNAMRSHVLEIASGADIVDAIAGFSRRRQRGVSVLSGTGAVTNVTLREPAGAGGAAAVALRGRFEILSMSGAFLPAPAPPGATGLTVYLAGGQGQVVGGSVMGELIASGPVMVIAATFGNATYERLPLDQADAEEGAVLSGSSEGATAQQLEQQQSSGGPVVPPSMYAVPQTPPHPHDMFGQWGHAAVTRPPPTSF

>ZmAC209845.4_FGT010

MDREGHYGGRGGGRLLECFSDEVSSGNGGGEERANDGGGPRPPAPGSGVVGAWKRRRGRPPGSKNKPKPQAAAAAAAVARDVEPASSAMRPHVLEVPSGGDVARALAGFARRRGLGICVLAGTGAVADVSLRHPSSSADGAGGSAAVFRGRYEILSISATFLAPSTPAAVARATVRDLSVSLAGPHGQVVGGAVVGPLVAATAVVVLAAAFTDLTFHRLPLEDDAPASVSDIAGADERRGHGRHQYPEQHDAGGSSHPQAMAPATQPVPLFARESHELWPAAPASAQRPRSPYQ

>Zm2G072274_T01

MDPVAAAHGGGHHFAPPVAPFHHAFGGGHHLFPGQQHPAFQHFQEHQLELMGGVPAGAKQELGDDSNNTINSAGSNGSGDGDQQQQQQQAGGDVDNHHQHHHHQQAAVMRRPRGRPAGSKNKPKPPVIITRDSASALRAHVLEVAAGCDVVDSVAGFARRRQVGVCVLSGAGSVANVCVRQPGAGAGAVTLPGRFEILSLCGSFLPPPAPPAATGLTVYLSGGQGQVVGGSVAGPLLASGPVVIVAACFGNAAYERLPLDDDDLPQQQQHLPPPPPIPQGMPGAAGAGGQSSSPPPPPLPLPGQHPLADQLPHGLINGLPLPADAYGWANPGGGAGRVAPY

>Zm2G024005_T01

MAGMDPGGGGGAASHYLELLRAQQQQLLQQHQQAPLSPSSHVKMERSAPSPENVDPGGDQPASEGSGGSGGPTRKPRGRPLGSKNKPKPPIIITRDSPNALHSHLLEVAAGADIVECVSEYARRRCRGVCVLSGGGAVSNLALRQPGADPPGSLLATLRGQFEILSLTGTVLPPPAPPGASNLSVYVAGGQGQVMGGSVAGQLIAAGPVVLMAASFANAVYERLPLEEEEEEAATAAAAAAAVTETQGAAVEPAEGQQQEASQSSGVTGGDVGGIGHGMSLYDLGGNAAGYELPGENFGTWSGGIRPPF

>Zm2G033480_T01

MASSKWWEQAALDFPPPQQHHHQQPQPQQQPHQAVPMPPALATAPAGAVGASPEGKQQGQQQAGAIVPLRRPRGRPLGSKNKPKPPVIITRDSPDALHSHVLEVSPGADVCACVAEYARRRGRGVCVLGASGAVGDVAVRGAAAPLRGRFELLSVTGTVLPPPAPPEASGLAVLVSAGQGQVLGGSVVGPLVAAGPVTIFAATFANAVYERLPLADAPELEVKPDLSTATSAGGQDVQPQLPMAPSHQQPPDMGAGYAGHRSPPYPWGGDV

>Zm2G092268_T05

MAPSSKDGATATEQPTSGDDDRENGGTGEPKEGAVVAGNRRPRGRPPGSKNKPKPPIFVTRDSPNALRSHVMEVAGGADVAESIAHFARRRQRGVCVLSGAGTVADVALRQPAAPGAVVALRGRFEILSLTGTFLPGPAPPGSTGLTVYLAGGQGQVVGGSVVGTLTAAGPVMVMASTFANATYERLPLDDADEEPAGQQAAQLPPGPGGGQPMVMGGMADPSAVPMFGGAGGVPPSLMPAGAAAASSGAGLQLGHDRLAWAHARPPPY

>Zm2G029096_T02

MSLGKTDMSQERLYHQDRMDVPPVHFSTPPPPPCQQQQQHGGHGEQQKLERFSDEVDSRLSAEQKEPASGGALVVSVSGGGDGASIELSKKRRGRPPGSKNKPKPPVVITREAEPAAAMRPHVIEIPCGCDVADALARFAARRNLGICVLAGTGAVANVSLRHPMPCGGGGAPTAIMLHGQYEILSISATFLPPAISAVAPQAAAAAACLSISLAGPHGQIVGGAVAGPLYAASTVVVVAAAFTNPTFHRLPADDDASVSVSVSLSAGSGDPADEHRGGHQHPVPVEPPPRERHRPHVVRRKPAPHLASAAPSVEPCGPPAAPIYAACHPQPQDVMWPPPNRAPHPPPPPY

>Zm2G044856_T01

MAGEKTASSGATDLVDPATATATAQKKPRGRPLGSKNKPKPPVVVTRESEAAMRPVVLELAAGCDVVGAVAAFARRRRVGVSVLCGRGAVAAVTLRLAASSAAVTLHGRFEVLALSGTVVPSSSSASASAPAFSVSLAGEGGQVIGGTLAGEMTAADGVVVVAAVFGSAQVHRLPAAGAEDEDSGGGREEGMRAQVAVGLGGTGTGGGLVVVGRQHAQQLPEKYKFSFFQYTTPVASTTRLAYTGSHDRLASFTHIGHL

>Bra003217

MANPWWVGNVAIEGVESPVTSSAPSLHHRSSNNPNMTRSDPRLDHDFTNNSGSPNTHTQNSQEEQDELPAVEHGSGSGSTGRRPRGRPPGSKNKPKNPVVVTKESPNSLQSHVLEIATGADVAESLNAFARRRGRGVSVLSGSGLVTNVTLRQPAASGGVVSLRGQFEILSMCGAFLPTSGSPAAAAGLTIYLAGAQGQVLGGGVAGPLIASGPVIVIAATFCNASFERLPIEDEQQQPQVEEAKEKENDDNKSGNDGTEESMQPMYNMTPNFMPNGHQMAQHDVFWGAPPARAPPSY

>Bra007176

MANPWWVESPVTSSAPSLHHRSNNPSMTLSDPRLDHDFTNNSGSPNTQTQNSQEEQTSRDELPAVEPGSGSGSTGRRPRGRPPGSKNKPKNPVVVTKESPNSLQSHVLEIAAGADVAESLNAFARRRGRGVSVLSGSGLVTNVTLRQPSASGGVVSLRGQFEILSMCGAFLPTSGSPAAAAGLTIYLAGAQGQVVGGGVAGPLIASGPVIVIAATFCNATFERLPIEDEQQGEQQQPQVEEAKKETDDNESGNDGNDGSMQAQQMYSMGPSFVPNGHQMGQQDVFWGAPPPRGPPSY

>Bra014735

MANPWWVGNVAMGGVESPVTSSAPSMHHRSNNPSMPRSDPRLDHDFANNSGSPNTQTQTQNSQEEPNSRDEVLAIEPGSGSGSTGRRPRGRPPGSKNKPKNPVVVSKESPNSLQSHVLEIATGTDVAEALNAFARRRGRGVSVLSGSGLVTNVTLRQPAVSGGVLSLRGQFEILSMCGAFLPTSGSPAAAAGLTVYLAGAQGQVLGGGVAGPLTASGPVIVIAATFSNATYERLPIEDDQQQPQLEEAKKEKEKDDNESGNDGNEGSMQPLPPPMYNMPPGFMPNGQQMAQHDVYWGAPP

PRGPPSY

>Bra040184

MANPWWTGQVNLSGLEATPPSSSQLKKPDLHISMNMAMDSDHNNHHHHQEVDTNNNNNEDDRDNLSGDDHEPREGAVEAPTRRPRGRPAGSKNKPKPPIFVTRDSPNALKSHVMEIASGTDVIETLATFARRRQRGICILSGNGTVANVTLRQPSAATVPVPPGGAAVLALQGRFEILSLTGSFLPGPAPPGSTGLTIYLASGQGQIVGGSVVGPLMAAGPVMLIAATFSNATYERLPLDEEEAAEGGGGGVVPGQLGGVGSPLSSGGGRGDGNQGLPVYSMPENLVSSGGGSGGGGQMSGQEAYGWAQARSGF

>Bra012639

MAGLDLGTSFRYVNHQLHRPDLHLHHNSSSDDVTPGVGIGHFTADDEDNNHQGLDLASGGGGSGSSGGGHGGGGGDGVGRRPRGRPPGSKNKPKPPVIITRESANTLRAHILEVTNGCDVFDCVATYARRRQRRICVLSGSGTVTNVSIRQPSAAGAVVTLQGTFEILSLSGSFLPPPAPPGATSLTIFVAGGQGQVIGGSVVGELTAAGPVIVIAASFTNVAYERLPLEEDEQQHLGGGGGANGGGNLFPEVAGGGGGGLPFFNLPMNMQPNVQLPVEGWPGNSGGRGPF

>Bra040183

MAGLDLGTAFGYVNHQLHRPDLHLHHSSASDDVTPGAGLGHFTVDDDDNNHQGLDLASGGGSGSSGGGGGDGGGGGNVVGRRPRGRPPGSKNKPKPPVIITRESANTLRAHILEVTSGCDVFDCVATYARRRQRGICVLSGSGTVTNVTIRQPSAAGAVVTLQGTFEILSLSGSFLPPPAPPGATSLTIFLAGGQGQVVGGSVVGELTAAGPVIVIASSFTNVAYERLPLEEDEQQHLGGGGGSNGGGNLFPEVSAGGGGGGLPFFNLPMNMQPNVQLPVEGWPGNSGGRGPF

>Bra005355

MAGLDLGTTSRYVHNVEGGGVGQFSTDNHHEDGGGAGGNHHHHHHNHNHHQGLDLIASNDNSGLGGGGGASGELVMRRPRGRPAGSKNKPKPPMIVTRESANTLRAHILEVGSGCDVFECISTYARRRQRGICVLSGTGTVTNVSIRQPTAVGAVVTLRGTFEILSLSGSFLPPPAPPGATNLTILLAGAQGQVVGGNVVGELMAAGPVMVMAASFTNVAYERLPLDEHEEQLQVQSGGANMYTEATNGGGGGSLPFFSLPMSLPHMGVDNWPGNSSGAGRAPF

>Bra023007

MAGLDLGSTSRYVHNVGQFSTDNHHEDDGGAGGTLQENINFETFLATTNIESWLQLEPLQVVASDGTSSRRGAVQLEPVEKLVGVRDVDVVKLMFSCSGNHHHQGLDLIVSNDNSGLGGGGGGGSGDLVMRRPRGRPAGSKNKPKPPTIVTRESANTLRAHILEVGSGFDVFECISTYARRRQRGICVMSGTGTVTNVSIRQPTAVGAVLTLRGTFEILSLSGSFLPPPAPPGATSLTIFLAGAQGQVVGGNVVGELMAAGPVMVMAASFTNVAYERLPLDEHEEQLLVQSGGGGGGNIYSEATGGGGGLPFFSLPMSLPHMGGEHWPGNSAGTGRAPF

>Bra017324

MSGLNMGTTSRYVHNVDGGGAGQFSTDNHHEDDGGAGENHHHNLHNHHQGLDLIASNDNSGLGGAGGGGEGSGDLVMRRPRGRPAGSKNKPKPPVIVTRESANTLRAHILEVGSGCDVFECISTYARRRQRGICVLSGTGTVTNVSIRQPTAAGSVVTLRGTFEILSLSGSFLPPPAPPGATSLTIFLAGAQGQVVGGNVVGELVAAGPVMVMAASFTNVAYERLPLDEHEEHLQVQSGGGGGGNMYSEANGGGGGLPFFNLPMSLPHMGVENWQGNSAGAGRAPF

>Bra016853

MAGGTALNSTSVGSKSVPMRNHEATERGNNKNNNNNKNLKALPKPVQPASSMKGEMAKRPRGRPAGSKNKPKPPIIVTQDSPNSLRAHALEISSGNDICETLSDFARRKQRGICILSANGCVTNVTLRQPASSGAIVRLHGRFEILSLLDSILPPPSPVGITGLTIYLAGPQGQVGGGGVVGGLIASGPVVLMVASFMNAVFDSLPLDDDEAASMQNQQYYHNGRSHPLDDIQRLPQNLLTNGHSDSDIYWGPAQRAMSKP

>Bra013649

MDPVQSHGSQSSLPPTFHSRDFQLHLQQQQQQQEFFLHHHQQQRNQPDQDDHQGGGRLNRQIKMDREETSDNMDNMANNSGSEGKDMNLGEGGSGGGSGGDQMTRRPRGRPAGSKNKPKPPIIITRDSANALRTHVMEIGDGCDLVESVATFARRRQRGVCVMSGTGNVMNVTIRQPGSPSPGSVVSLHGRFEILSLSGSFLPPPAPPNATGLSVYLAGGQGQVVGGSGGGPLLCAGPVVVMAASFSNAAYERLPLEEDEMQTPVHGGGGGGGSMEPPPMMGGPMPHQQQAMSAHQGLPPNLLGPTHQGLPQHDNQSYWSTGRPPY

>Bra004732

MAGGAALTPTSVGSKSLLPLRNHEAVAERGTNNNNSNMKALPKAVQPVSSIEGEMAKRPRGRPAGSKNKPKPPIIVTHDSPNSLRAHAVEISSGNDICETLSDYARRKQRGLCILSANGCVNNVTLRQPASSGGIVTLHGRFEILSLLGSILPPPAPLGITGLTIYLAGPQGQVVGGGVVGGLIASGPVVLMAASFMNAVFDRLPLDDDEAASMHNQQYYQNGRSRPLDDIHGLPQNLLTNGNSGSDIYSWGAAQRAMSKP

>Bra019355

MDGTQSSLPPPFHSRDFQLHLQQQQQQQQEFFLHHHQQQRNQTDQDDQQGGGGINRQIKMDREETSDNIDNMANNSGSEGKDTNQGEGGSGGGGGSGGDQMTRRPRGRPAGSKNKPKPPIIITRDSANALRTHVMEIGDGCDLVDSVATFARRRQRGVCVMSGTGNVTNVTIRQPGSPSPGSVVSLHGRFEILSLSGSFLPPPAPPNATGLSVYLAGGQGQVVGGSVVGPLLCAGPVVVMAASFSNAAYERLPLEEDEMQTPVHGGGGGGGGGSMESPPMMGGQMPHQQQAMSAHQGLPPNLLGGSAHQGLPQHDNQPYWSTGRPSF

>Bra000281

MAGGTALTPTSVGSKSLLPLRNHEAAERVNNNNNNNSLKALPKAVQPVSSIDGGMVKRPRGRPAGSKNKPKPPIIATHDSPNSLRAHAVEISSGNDICEALSDYARRKQRGLCIFSANGCVTNVTLRQPASSGAIVTLHGRFEILSLLGSILPPPAPLGITGLTIYLAGHQGQVVGGGVVGGLIASGPVVIMAASFMNAVFDRLPLDDDEAASMQNQQYYQNGRSLPLDGIHGLPQNLLTNGNSGSDMYSWGPAERAMSKP

>Bra040135

MANPWWTGQVNLSGLETTPPSSSQLKTPDLHISMNMAMDSGHNNHHHHHQEVNTNNNNEDDRDNLSGDDHEPREGAVEAPTRRPRGRPAGSNILLIASGTDVIETLATFARRRQRGICILSGNGTVANVTLRQPSVAPVAAAPGGAAVLALQGRFEILSLTGSFLPGPAPPGSTGLTIYLAGGQGQVVGGSVVGPLMAAGPVMLIAATFSNATYERLPLDEEEAAERGGGGSDGGVVPGQLGGVGSPLSSGGGGGHGNQGLPAYNMPGNLASNGGGGGQMSGQEAYGWAQARSGF

>Bra015738

MDGGYDESGGASRYFHNLFRPELHHQLQPQPHLQTHLQPQPHSDDESDSNKDPGPPDSDPVTSGSSQGKRSRGRPPGSKNKPKPPVIVTRDSPNVLRSHVLEISSGADIVESVNTYARRRGRGVSVLSGNGAVSNVVLRQPATTHRSNGGVGAGAGGVVTLHGRFDILSMTGTVLPPPAPPGAGGLSIFVAGAQGQVIGGSVVAPLVASGPVILMAASFSNATFERLPLEDEGGEGGGGGGPQPPTSASPPSGAAQGDLRGNMSGYDQFSGDPHLLGWGAGAASRPSF

>Bra039338

MDQVSRSLPPPFLSRDLHLHPHHQFQHQQQHNHGHDIDQHRIGGLKRDRDSEIDPNEHSSAGKDQNTPGSGGESGGGGGGGDNHITRRPRGRPAGSKNKPKPPVIITRDSANALKSHVMEVANGCDVMESVTVFARRRQRGICVLSGNGAVTNVTIRQPASVPGGGSSVVNLHGRFEILSLSGSFLPPPAPPAASGLTIYLAGGQGQVVGGSVVGPLMASGPVVIMAASFGNAAYERLPLEEDDQDEQQTAGAVANNIDGNATMGGGTQTQPQQLMQDPTSFIQGLPPSLMNSQLPAEAY

WGTPRPSF

>Bra012256

MEGGYEQGGGANSRHFHNLFRPEIHHQQFQPQGGINLTDQHRHQQQHEQHQQPSDDSRESDHSNKDHHQPGGPDSSDRATSSSAPGKRPRGRPPGSKNKAKPPIIVTRDSPNALKSHVLEVSPGADIVESVSTYARRRGRGVSVLGGNGTVSNVTLRQPVTSGNGVGVGAGGVVTLHGRFEILSLTGTVLPPPAPPGAGGLSIFLAGGQGQVVGGVVVAPLVASAPVILMATSFSNAVFERLPIEEEEEEGGDGGAGGAPQMQQAPSASPPSGVTGQGQLGGNVGGYGFSADPHLHGWGAGTPSRPPFN

>Bra011612

MSGYMHPLLGQELHLQRPEDSRTPPDHNNMELNRSEADEAKGETTPTGGGAANSATASGSSSGRRPRGRPAGSKNKPKPPTIITRDSPNVLRSHVLEVTSGSDISEAVSTYATRRGCGVCVLSATGAVTNVTIRQPAAPTGGGVITLHGRFEILSLTGTALPPPAPPGAGGLTVYLAGGQGQVVGGNVAGSLIASGPVVLMAASFANAVYDRLPMEEEETPPPRTTGVQQPQPAASQSSEVTRSEAQMGESNMGGRNGGVAFYNLGMNMNNFQFSGGDIFGLSGGGGGGGGGDGGGVTRPAF

>Bra004911

MDQVSRSLPFLSRDLHLHPHHQFQHQQQHNHVHEIDQHRISGLKRDRDTKIDPNEHSPAGKDQNTPGSGGETGGGDNQITRRPRGRPAGSKNKPKPPIIVTRDSANALKSHVMEVANGCDVMESVTVFARRRQRGVCVLSGNGAVTNVTIRQPATVPGGGSSVVNLHGRFEILSLSGSFLPPPAPPAASGLTIYLAGGQGQVVGGSVVGPLMASGPVMIMAASFGNAAYERLPLEEEDQEEQQTSGAVVNNNNNIDGNGTMGGGTQTQQQQQLMQDPTSFIQGLQPNLMSSSVQLPGEAYWGTPRPSF

>Bra000593

MKGEGNEMFPKLPQNHSLSSHFHLPATIAVDDSSIEVVRRPRGRPPGSKNKPKPQVYVTRDTEPPMSPYILEVPSGNDVVEAINRFCRRKSIGVCVLSGSGSVANITLRQPSPAAPGSTITFHGKFDLLSVSATFLPPPPRTSLSPPVANFFTVSLAGPQGQIIGGFVAGPLISAGTVYVIAASFNNPSYHRLPAEEEHRHSVSARAGEGDGQSPPVSRGGGESEHVAVRGEESCGVSMYSCHMGTHGSDVIWAPTARAPPPY

>Bra008262

MDGGYDQSGGASRYFHGLFRSELHNQLQQQAQAQPQPHLQLQPDDESDSNKDSGRLDSDPVTSGSTPGKRPRGRPPGSKNKLKPPVIVTGDSPNVLRSHVLEVSSGADIVESVNTYARRRGRGVSVLSGNGTVANVVLRQPVTIHGNNGGTGAGVGGVVTLHGKFDILSITGTVLPPPAPPGSGGLSIFLSGGQGQVIGGSVVAPLVASGPVILMAASFSNATFERLPLKDEEEEGGGGGPPPAPTASPPSGPGQGEIRGNPSGYDQFTGDPHSLGWVAGAASRPSF

>Bra003717

MDGGYDQSGHSRYFHNLFRPELQHQLQPQPQPQPQPQPQPQPQSDDESDSNNKYPGQPDSDQVTSGSTSGKRPRGRPPGSKNKPKPPVIVTRDSPNVLRSHVLEVSSGADIIESVNNYARRRGRGVSILSGNGTVANLTLRQPVTTHGNNGGTEAGAGGVVTLRGRFEILSITGAVLPPPAPPGCGGLSIFVAGEQGRVIGGRVVAPLVASGPVILMAASFSNATFERLPLEEEGGEGGGDVGGGVPPPATSETAPSGVAQGELRVNMSGYDQFSGWGAGAASRPSF

>Bra026211

MEPVQRQRPRGRPQGSKNKPKPPVFLTVEPPMSPYILQVPSGNDVVASINRFCRERSIGLCLLSGSGSVADVTLRQPPPAPPGSTITFHGKFDLLSVSAAFLPPPVSSSFTVSLAGPQGQIIGGFVSGPLISAGPVYVVAASLNNPSYYRLPAEVKQENSSASAEAEEGKGQSPPVSGGGGESCHIEGSDVVWTPAAGILTKPSL

>Bra016444

MEGGYEQGGGASRYFHNLFRPEIHHQQQLQPQGGINLFDQHHHQQQQHQQQQEQPSDDSRESDHSNKDHHHQTGRPDSDPATSSSAPGKRPRGRPTGSKNKPKAPIIVTRDSPNTLRSHVLEVSPGADIVESLSTYARRRGRGVSVLGGNGTVSNVTLRQPVTPGNGGGVGAGAGGVVTLHGRFEILSLTGTVLPPPAPPGAGGLSIFLSGGQGQVIGGSVVAPLVASAPVILMAASFSNAVFERLPIEEEEEEGGGGGGGGGGPPQMQHAPSASPPSGVTGQGQLGGNVGGYGFPGDPHLLGWGAGTPSRPPFN

>Bra025854

MEGGYEQGGGASRYFHNLFRPEIHHQQLQQQGGINLFDQHHQQQQHQQQQQQQPSDDSRESDHSNKDHHQPGLPDSDPATSSSAPGKRPRGRPPGSKNKAKPPIIVTRDSPNALRSHVLEVSPGADIVECVSTYARRRGRGVSVLGGNGTVSNVTLRQPVTPGNSGGGAGGGVVTLHGRFEILSLTGTVLPPPSPPGAGAPVILMAASFSNAVFERLPIEEEEERGGGGVGEGEGPPQMQQAPSPSPRSGVTGQGQLGGNVGGYGFSSDPHLLGWGAGTPSRPPFT

>Bra017536

MDSRDIPQQFQPPLFHYPNFNTSAMMGPNSTSQSINHRLTFGSLTPRGTLQQQEEQLDQKTLESLGYVDEVSPSSQPMRSGIDQSQQQVKRKRGRPRKYAPDGTIALGLAPTSPLLGDGDSGRVNTNSAKRARGRPPGSIKKQYDALGTSGGMFTHVIEVQEGEEIVSKVAALSTQGPRTVIVLSAAGAVSRVILHNASGIQNYKGQFEIVTLSGSFSNYEVNGSIERTGSLTVALAGPNAQILGGLVGRLVAVTPVQIIVGSFVDEAKKLKQSTGNNAQGQNPEPASAPANMLNFGSNSQGPSSESSDENESGSPSVQHHDNNNNGIYGNSMAQQQQLRQMQMYNLWPSSGQ

>Bra039309

MDQRDPMGLTGSGSYYIQRGLPGSVPPTFHGSSQQQQGLRHLHNQNSPFGSGSTGFGSPPLHGDPSPAAATGAVPHHVGVHMISPPPPPPTSETPMKRKRGRPRKYGQDGSVSLALSSSSVSTINSSNKRGRGRPPGSGKKQRLSSTGELMMPSSSGMSFTPHVILVSIGEDIASKVIAFSQQGPRAICVLSASGAVSTATLLQSSTPPGAIQYEGRFEILALSISYLVPTDGSFRNRTGNLSVSLASPDGRVIGGAIGGPLIAASPVQVIVGSFVWAAPKIKNKKREEEGSENVQDTNDHQALDHPVPQQHTQGQNMMWSTGSRQMDMRQAHADIDLMRG

>Cpa26.5

MAGLESGPGSRYVQNLFRPELHHLHRPSINPPPPQPPLSSDSKDSHENTYQKPDSDDPTAPSASASNRRPRGRPPGSKNKPKPPIIVTRDTPNALRSHVLEVTTGADIVDSVSNYARRRGRGVSVLSGTGTVANVTLRQPAAPAGSVVTLHGRFDILSLTGTVLPPPAPPGAGGLSIFLAGGQGQVVGGSVVGPLLASGPVVLMAASFSNAVFERLPLDEEEAQALQVQPTASQSSGVTGSAQQLAEGGGGSSSAGAGGGGGGSFFTMGGANVGGYPFSGDLFGWGGNGVRPPF

>Cpa18.123

MDPVTAHGRSLPPPFHARDLHLVHPHHQFQNHHQPAQNSDQDDQQSGNSGLSRGQKRDRDETAGTTTIGIEGKELATTLVGAGEGEITRRPRGRPAGSKNKPKPPIIITRDSANALRSHVMEIANGCDIMESVTTFARRRQRGVCILSGSGTVTNVTLRQPAAPGAVVTLHGRFEILSLSGSFLPPPAPPAASGLSIYLAGGQGQVVGGSVVGPLLASGPVVIMAASFGNAAYERLPLEEEESAAAPVPGGSSGGLGSPEGVGQQQQQQQQLMQDPNAAFIHGLPPNLLNSVQLPAEAYW

GTARPPF

>Cpa44.95

MPGMDPAANSPLLHKSDLEISNNNKRGGDEDEDRDTGDEPREGAVEVGTRRPRGRPPGSKNKPKPPIFVTRDSPNALRSHVMEVASGTDVAESIAIFARRRQRGVCVLSGSGSVANVTLRQPAAPGAVVALHGRFEILSLTGAFLPGPAPPGSTGLTVYLAGGQGQVVGGSVVGSLVAAGPVMVIAATFANATYERLPLEEEEEADQGQIQGGSGNSPPAMGGGGSGLADPSSLPMYNIPPHLVPNGGGQLGQEAYGWNHTRQPY

>Cpa426.2

MSDLETRHYVHQLLGSDPVQLHPQNSNDDAPSTTTSVSATPTFGRRPRGRPQGSKNKTKPPVIITQDTPNALRCHILEISNGSDVVESVSNYATRRGQGVCVLSGTGAVTNVVLRQLAGSGPVTLPGRFEIVSLSGTALPPPAPAGAGGLSVYLAGGQGQVVGGMVAGPLMASGPVVLMATSFQNAAYDRLPLEVEKSATAVQPTASQSSGLTGNGEKIGGDGGGGGARGVSHNLESYPFPGGAGDILGWGFTSV

>Cpa21.4

MFSKLHHPSQHQHQHQHQHHFSHHFQLSRDTSESDDTSRSTGGPTTSNPAQKPVEGLTAIGGSGSGDGASIEVVRRPRGRPPGSKNKPKPPVFITRDFEPPMSPYILEIPGGNDVVEAISRFCRRKSMGICVLTGSGAVANVTLRQPSTTPGATITFHGRFDILSISATFLPPQTAPCPPVPNMFAISLAGPQGQIVGGSVAGALIAAGTVYIIAASFNNPSYHRLPADDEARNTASGSGGEGQSPPVSSAGGGESGHVAAGGAGGESCGMSMYSCHLAPSDVIWAPTARPPPPPPPY

>Cpa157.67

MADFGTSISLYKLQQTSDDSSSEHSPPTLPTLSTGCASGSGSSFSRPGDLTLTVPDHPLPPQIQPATTDAARKPRGRPPGSKNKPKLPVIITRDGDVALSPIVLEISVGSDIIETITSFARRHQIAGFSVLSASGSVSNITIRHPVSHAPTISLRGPFSILSLTGSVLGSVVTSSESGSTAMMPMASSSQPSESSFGLTIAGAQGQVFGGIVVGNLTAASKVTVVAATFQSCPYQKLQLETATSGRGDYIEANHYYHQDANPSSGVHHGGGGLAGVGGESSSHTGSAYGAAASPSTLNCQLPALDVMSWCPTSRSPY

>Cpa41226.1

MANRWWGGQMGLPGLDTSATTSSPMKKPDLGISMTTNNHETRGVSGGAHHEEEDERDHSDDPKEGAIDVATRRPRGRPPGSKNKPKPPIFVTRDSPNALRSHVLEINSGSDVAEALAHFARRRQRGICVLSGSGAVTNVTLRQPSATPSAVMALHGRFEILSLTGAFLPGQVLPGSSSGLTIYLAGGQGQVVGGSVVGQLVASGMVMVIAATFSNATYERLPLEEEEESGAGAAQGQVGGSGSPPGMGGSSDGGRGGHHQGGIGESGGLPVYNNLAPNLVGNGGQINQEAYGWAHGRTPF

>Cpa29.66

MDPVTSHAHSLPPPFHTRDFQLQLQQQQQHHQFLLHHQQQAHNSEDEQSGSSGGLNKAPKRDLEELTNGNGKELVAAAANSEGEITRRARGRPPGSKNKPKPPIIITRDSANALRTHVMEIGDGCDIVESVATFARRRQRGVCIMSGTGTVTNVTLRQPASPGAVVSLHGRFEILSLSGSFLPPPAPPAASGLTIYLAGGQGQVVGGSVVGTLTCSGPVVVMAASFSNAAYERLPLEEEEMQNPAMQGGPIGSPGSGSGQQQQVLSEQNAGLFHGLQPNLMNSMQLPAEAYWATGRPPF

>Cpa50.90

MAGGTPGVVSKTSLNRSQEFEKGSNHRTTNIDAMLMAPKLSKVVQPTEGETARRPRGRPAGSKNKPKPPIIVTRDSPNTLRAHAMEVGSGCDVCESLASFARRKQRGICILSGNGCVTNVTLRQPASSGAIVTLHGRFEILSLLGSVLPPPAPPGITGLTIYLAGAQGQVVGGGVVGAPIASGPVVIMAASFMNATFDRLPLDDDEVAATLQNQHYQNGRHHHLDIPELYGMPQNLITNGNMPPEIYSWAPPRTMSKT

>Cpa300.4

MAGLDLGTASRYVHQLHRPDLHQPDLEDHQEAGGGPTGGQFSDDGASHQGANSGSGDLVARRPRGRPPGSKNKPKPPVIITRESANTLRAHILEVGNGCDVFDCVANYARRRQRGICVLSGSGTVTNVSIRQPAAAGAIVTLHGRFEILSLSGSFLPPPAPPGATSLTIFLAGGQGQVVGGNVVGELMAAGPVIVIAASFTNVAYERLPLEEDDQLQMQSGGGGGGGSGSGGSGGGNSMFAEAAGSGQGGGLPFFNLPLNMPPNVQLPF

>Cpa19.284

MANRWWAGNVGMRNVDQISPPPSLHLRNPEEEHTKFIRNHSANPTTHTPNTVNQSQSHEDEDSRDDNNNTGLELEEATGHDSIEPGSSSGGPGSSGGRRPRGRPPGSKNKPRPPVVITKESPNSLRSHVLEIASGSDIVDCVATFSRRRHRGVSVLSGSGVVTNVTLRQPAAPGGVINLQGRFEILSLSGAFLPTPSPPGATGLTVYLAGGQGQVVGGGVMGALVASGPVIIIAATFSNATYERLPVEDDHGGVQPQQQAGNGSNENQENSSGGGNAQTTQGLGGEATVPPLYNLPQNLQLPNGQLPHDVFWVPPPRPPPSY

>Mdp212634

MAGYNINESASVSAASSAPTSTASPYVHPLFRPQLHLQLQQLHHHPIIPQQQQHHHQQEEQDDDDDQDDDQDHNITKIESSDTAATSSGGGGGGGGTGGSTRRPRGRPAGSKNKPKPPIIVTRDSPNALRSHVLEVSDGADIMDSVSIYARRRGRGVCVLSGSGTVTNVTLRQPAAPPGSVVTLHGRFELLSLSGTVLPPPAPPGAGGLSIFLSGGQGQVVGGNVVGPLMASGPVVLMAASFANAVFERLPLDDPEEGTTTPTGGGGGGSLQIHQPTGSQSSGVSGGLGDGTAGSSGGGGGGGGGAGLFNLGGNMAANYPFPGXDFFGWGGGSGSGSTPRPPF

>Mdp892159

MAGYNINESASVSAASSAPTSTASPYVHPLFRPQLHLQLQQLHHHPIIPQQQQHHHQQEEQDDDDDQDDDQDHNITKIESSDTAATSSGGGGGGGGTGGSTRRPRGRPAGSKNKPKPPIIVTRDSPNALRSHVLEVSDGADIMDSVSIYARRRGRGVCVLSGSGTVTNVTLRQPAAPPGSVVTLHGRFELLSLSGTVLPPPAPPGAGGLSIFLSGGQGQVVGGNVVGPLMASGPVVLMAASFANAVFERLPLDDPEEGTTTPTGGGGGGSLQIHQPTGSQSSGVSGGLGDGTAGSSGGGGGGGGGAGLFNLGGNMAANYPFPGSDFFGWGGGSGSGSTPRPPF

>Mdp859711

MAGYNINESASVSAASSAPTSTASPYVHPLFRPQLHLQLQQLHHHPIIPQQQQHHHQQEEQDDDDDQDDDQDHNITKIESSDTAATSSGGGGGGGGTGGSTRRPRGRPAGSKNKPKPPIIVTRDSPNALRSHVLEVSDGADIMDSVSIYARRRGRGVCVLSGSGTVTNVTLRQPAAPPGSVVTLHGRFELLSLSGTVLPPPAPPGAGGLSIFLSGGQGQVVGGNVVGPLMASGPVVLMAASFANAVFERLPLDDPEEGTTTPTGGGGGGSLQIHQPTGSQSSGVSGGLGDGTAGSSGGGGGGGGGAGLFNLGGNMAANYPFPGXDFFGWGGGSGSGSTPRPPF

>Mdp716521

MDTHSLPPPFHTRDFHLHHQQQHPQFLHQQQNSEDEQTGSSGLNKGQKRERDIDNNDSGGNGGELGKELNVTMSGGDGSEMTRRPRGRPAGSKNKPKPPIIITRDSANALRSHVMEIADGCDIVESVATFARRRQRGVCIMSGTGTVTNVTLRQPASPGSVVTLHGRFEILSLAGSFLPPPAPPAATGLTIYLAGGQGQVVGGSVVGTLIASGPVVIMAASFSNAAYERLPLEEDEGQLPMQGGGGSVGSPTGVGHQNQQQQQPQHQQLLAEAANSNAPLFHGLHPNLLNSMELPAEAAYWATSRPPF

>Mdp934489

MAGLDLGSVPRYVHQLHRQDLHLQQQQQQQTDSEDDAIVKRSTGQFSGDDHHQGGLDLGGINGGSGDIVARRPRGRPPGSKNKPKPPVIITRESANTLRAHILEVGNGCDVFDCVATYARRRQRGICILSGSGTVTNVTLRQPAAAGAVVTLHGRFEILSLSGSFLPPPAPPGATSLTIFLAGGQGQVVGGSVVGELTAAGPVIVIASSFTNVAYERLPLDEEEQLQVQVPQGSGGSGGGGGGGSVGNNPFPDPSSGLPFFNLPLNMQNVQLPIDGWAGNGNNSGGRPLF

>Mdp946489

MAGLDLGSASRYVHQLHRQDLQLQQQHQQQTDSEDDAVANRSTGRFSSGDDHQGSLNLGGINGGSXEIVARRPRGRPPGSKNKPKPPVIITRESANTLRAHILEVGNGCDVFDCVATYARRRQRGICILSGSGTVTNVTLRQPAAVGAVVTLHGRFEILSLSGSFLPPPAPPGATSLTIFLAGGQGQVVGGSVVGELTTAGPVIVIASSFTNVAYERLPLDEEEQLQVQDPQGSGGTGGGGGGVGGVSVGNNPFPDPSSGLPFFNLPLNMQNVQLPFDGWAGDGNNSGDRPPF

>Mdp890692

MDTHSLPPPFHTRDFHLHHQQQHPQFHHQQQNSEDEQTGSSGLNKGQKRERDIDNNDSGGNGGELGKELNVTMSGADGSEMTRRPRGRPAGSKNKPKPPIIITRDSANALRTHVMEIADGCDIVESVATFARRRQRGVCIMSGTGTVTNVTLRQPASPGSIVTLHGRFEILSLAGSFLPPPAPPAATGLTIYLAGGQGQVVGGSVVGTLXASGPVVIMAASFSNAAYERLPLEEDEGQLPMQGGGGGSLGSPSGVGHQNQQQQQQQQQQQLMAEAANSNPPLFHGLPPNLLNSMQLPAEAAYWATGRPPF

>Mdp313865

MDTHSLPPPFHTRDFHLHHQQQHPQFLHQQQNSEDEQTGSSGLNKGQKRERDIDNNDSGGNGGELGKELNVTMSGXDGSEMTRRPRGRPAGSKNKPKPPIIITRDSANALRTHVMEIADGCDIVESVATFARRRQRGVCIMSGTGTVTNVTLRQPASPGSIVTLHGRFEILSLAGSFLPPPAPPAATGLTIYLAGGQGQVVGGSVVGTLTASGPVVIMAASFSNAAYERLPLEEDEGQLPMQGGGGGSLGSPSGVGHQNQQQQQQQQQQQLMAEAANSNPPLFHGLPPNLLNSMQLPAEAAYWATGRPPF

>Mdp272612

MANRWWAGHVGLPGGGVLNETSAVAAAGSSLMKNIIKPDLGISMNNTGTSSLGGSGGDEDDNNSDDPKEGAIEVATRRPRGRPPGSKNKPKPPIFVTRDSPNALRSHVMEISNGADIANSVAQFARRRQRGVCVMSGSGTVTNVTLRQPSAPGAVMALHGRFEILSLTGAFLPGPAPPGSTGMTIYLAGGQGQVVGGSVVGPLVASGPVMVIAATFTNATYERLPLEEEEEQAGGGTSSGTAAGGAGSPPGIGGGGMGDPAALAGVYNFPPNLLSNGGQTQLSHDQQAAYSSWAHGQGGGRPPF

>Mdp899407

MANRWWAGQVGLPGGGVVNETSAVAAAGSSPMKNLMKPDLGISMNTTGTSSLGGSGGDEDDNNSDDPKEGAIEVATRRPRGRPPGSKNKPKPPIFVTRDSPNALRSHVMEISSGADIADSVAQFARRRQRGVCVMSGSGTVINVTLRQPSAPGAVMALHGRFEILYLTGAFLPGPALPGSTGLTIYLAGGQGQVVGGSVVGPLVASGPVMVIAATFSNATYERLPLEEEEEQTGGGTSSGTAAGGAGSPPGIGGGGMGDPAALAGIYNLPSNMLPNGGQAQLSLDQQAAYSSWAHGQGGGRPPF

>Mdp857626

MDPVAAHGRPIPPPFLSRDLHLHPHHQFQQHLHPHHLNHQNSEDEQNGSGINRGIKRDRGGGDENSAGGTTSLEGKDQLGSTSAGEAEITRRPRGRPAGSKNKAKPPIIITRDSANALRSHVMEVANGCDIMESVSTFARRRQRGVCILSGSGTVTNVTLRQPASPGAVVTLHGRFEILSLSGSFLPPPAPPAASCLTIYLAGGQGQVVGGGVVGPLLASGPVVIMAASFGNAAYERLPLEEEEEPAVAGQVQGSGGSLGSPGIGGQEHQQQSLQQQQQQQQLLQDPNAPSLFHGMPQSLLNQLPADAYWGTARPPY

>Mdp150857

MAGGSDLAVPSVVSKTVIDRSQDADKSNSHHKPGNDAMLTAPKLPKAVTLVSEGETLRRPRGRPAGSKNKPKPPIIVTRDSANALRAHAMEVSSGCDVSETLTHFARRKQRGICILSGSGCVTNVTLRQPASSGAIVTLHGRFEILSLLGSILPPPAPPGITGLTIYLAGPQGQVVGGGVVGALIASGPVVIMAASFMNATFDRLPLDEDEVAVALQNQQYQNGRHHHLDISDLYGLPQNLITNW

>Mdp282029

MANRWWAGNVSMGGGGHVDSISSTPPSLHLRNTEEQLDDQDNTTPTNSSTSPHKQNNKHHEDGRDNNDLEGDDQGPNTGSGSHDSLEPGSSNRRPRGRPPGSKNKPKPPVVITKESPNALRSHVLEISSGSDIVDSIATFSQRRHRGVSVLSGSGIVANVTLRQPAAPTGVITLHGRFEILSLSGAFLPAPSPPGATGLTVYLAGGQGQVVGGTVMGALVASGPVMVIAATFTNATYERLPLEEEQGGEGGMQIQQQSGVNSAGTGANSGSRGVGEDPSAMAAYNLPPNLLANGGQIPHDVFWGPPPPRPPPSY

>Mdp931438

MANRWWAGNVSMGGGGGVHVDSISSTPPSLHLRNTEEQLDDQNNTTTPTNSSTSPHKQNKKHHEDDRDNNDQEGDDQGPNTGSGSHDSLEPGSSNRRPRGRPPGSKNKPKPPIVITKESPNALRSHVLEISSGSDIVDSIATFAQRRHRGVSVLSGSGIVANVTLRQPAAPTGVITLHGRFEILSMSGAFLPSPSPPGATGLTVYLAGGQGQVVGGTVMGALVASGPVMVIAATFTNATYERLPLEEEQGGEEGMQLEQQQQQQSGVNSAGTGANSNSQGLGEHTSAMAVYNLPSNLLANGGQIPHDIFWGPPPPRPPPPSY

>Mdp175382

MDPVVTNSPTLSKQRELEISINETNSGRSSGRDDDDDRDHDEPKEGAVEIGSRRPRGRPPGSKNKPKPPIFVTRDSPNSLRSHVMEVAGGADVAESVAQFARRRQRGVCVLSGSGSVANVTLRQPAAPGAVVALHGRFEILSLSGAFLPGPAPPGSTGLTVYLAGGQGQVVGGSVVGSLVAAGPVMVVAATFANATYERLPLEEDEEGGGSGEEEEEEEEEGIIMEVPQLVVGAVEPNWGVGGIISSSNSLILHLDFPIFTVIICLQI

>Mdp726292

MAGLDLGSASRFVHQQLQLPDLHLQRPPDSEDDHTTPNRNNLFSSDHHQNDVDNDNPHHQGFDLVTPNPGSGDSGGRRSRGRPPGSRNKPKPPVIITRESANTLRAHILEVSSGCDVFDSVGTYARKRQRGICVLSGSGMVTNVSLRQPSAAGAVLTLHGRFEILSLTGSFLPPPAPPGATSLTIFLAGGQGQVVGGNVVGALIASGPVIVIASSFTNVAYERLPLEEEEQLQMQQTVPQSSGGGGGGDGSGGGGVSNPLSRSVFGASLL

>Mdp715375

MAGLDLGSASRFVHQQLQLPDLHLQRPPDSEDDHTTPNRNNLFSSDHHQNDVDNDNPHHQGFDLVTPNPGSGDSGGRRSRGRPPGSRNKPKPPVIITRESANTLRAHILEVSSGCDVFDSVGTYARKRQRGICVLSGSGMVTNVSLRQPSAAGAVLTLHGRFEILSLTGSFLPPPAPPGATSLTIFLAGGQGQVVGGNVVGALIASGPVIVIASSFTNVAYERLPLEEEEQLQMQQTVPQSSGGGGGGDGSGGGGVSNPFPDPSSGLPFFNLPLNNMPHQLQVDSWGGNSAVRPPY

>Mdp268369

MAGLDLGSASRFVHQQLQLPDLHLQRPPDSEDDHTTPNRNNLFSSDHHQNDVDDDNPHHQGLDLVTPNSGSGDSGGRRSRGRPPGSRNKPKPPVIITRESANTLRAHILEVTSGCDVFDSVGTYARKRQRGICVLSGSGMVTNVSLRQPTAAGAVVTLHGRFEILSLTGSFLPPPAPPGATSLTIFLAGGQGQVVGGNVVGALIASGPVIVIASSFTNVAYERLPLEEEEQLQMQQPLPQSSGGGGGGEGSGGGGASNPFPDPSSGLPFFNLPLNNMTHQLQVDSWGGNSAGRPPY

>Mdp316599

MDPVATNSSTLNKQXGLEISINENSGRSSGRDEDDDRDHTEPKEGAVEIGSRRPRGRPPGSKNKPKPPIFVXRDSPNSLRSHVMEVASGTDVAESVAQFARRRQRGMCILSGSGSVANVTLRQPAAPGAVVXLHGRFEILSLSGAFLPGPAPPGFTGLTVYLAGGQGQVVGGSVMGSLVAAGPVMVXAATFANATYERLPLEEDEEGGGGGGGHNNGGSPTGGGSSGTQLGSGGHHHQQQQLPDPSSGLPNIYSYHLPPNLIPNGGHGQLGHEAYANWAXS

>Mdp933110

MRGEYVDSKSDSQNIFSKLHHPNQQNPHHHNLHHHPHQFSSPFQVIPHRESQTSEEEDTSRTSSGTATVTTTNPSPHNPHPNSSSDPNNINNPSADGATIEVIRRPRGRPPGSKNKPKPPVIITRDSEPPMSPYILEVHSGNDVVEAVSRFCSRKNIGLCVLTGSGTVANVTLRQPSTTPGATVTFHGRFDILSISATFLSQSLPSCPVSNPSGFTISLAGPQGQIVGGLVAGALIAAGTVYLVAASFSNPSYHRLPVEDEVVRNSVVGEGHSPQVSGGAADSGGHAPQPSQTCGMTMYSGHLPTDVIWAPTARQPPPPPPY

>Mdp144765

MADTGAGGAAAISLQQASDDDSMHSPRSVPTLSGGASGGGGGGCSSSGHKNMVSPLGDIIISNSKKPRGRPPGSKNKPKPPIVITKDSGSAMKAVVLEISAGSDVVETIVQYARRRQVGISVLSGSGAVLNVKLRHPAGPHEPSLSLQGPFNLLSLSGSYIDSFPAVIACSSSPSAAGGGGGSAVVPMSGTYSLLACSSFTICLAGAQGHVFGGIVGGKVVAASTVMVVAATFLDPTLQRLPLPVEGGGEYEGEEDTKPCINEQSCHTYGGGGGGRSSGVASPTCQMNTSPPDHYHQVMAWGPPSRTPY

>Mdp143696

MADYGAGGGAAISLSQASADDSEHSPRSLPTLTGSASGGGGCSSSRHKNMVSPSGEIIISNSKKPRGRPPGSKNKPKAPIVITKDSEAAMKAVXLEISGGSDVVETIEQYARRHHVGISVLXGSGTVLSVTLRHPAGPHAPSLSLQGPFNLLSLSGSYIDSFPTIITCSSSPAAGSAGGGGGGSANVPTSGTCSLLACASFGICLAGAQGHVFGGIVGGKVVAAGTVIVVAATFQNPTLQRLPLPVESGDCGGVVVLTAXAAAGVVVVASPTPLSFRMNTSPAGHHHQIMAWGPPSXSPY

>Mdp172380

MADYGAGGGAAISLSQASADDSEHSPRSLPTLTGSASGGGGCSSSRHKNMVSPSGEIIISNSKKPRGRPPGSKNKPKAPIVITKDSEAAMKAVXLEISGGSDVVETIEQYARRHHVGISVLXGSGTVLSVTLRHPAGPHAPSLSLQGPFNLLSLSGSYIDSFPTIITCSSSPAAGSAGGGGGGSANVPTSGTCSLLACASFGICLAGAQGHVFGGIVGGKVVAAGTVIVVAATFQNPTLQRLPLPVESGDCGGVVVLTAXAAGVVVVASPTPLSFRMNTSPAGHHHQIMAWGPPSXSPY

>Mdp131550

MPNLRRRRHQPNQQRHRHRHQHQPXSPTTPHPTSSADPINNNNPSADGATIEVIRRPRGRPPGSKNKPKPPVIITRDSEPPMSPYILEVPSETTSSKLSLASPSTTPGATVIFHGRFDILSISATFLPHSSPSCPVSIPSGFTISLAGPQGQIVGGLVGGALIAAGTVYLVAASFNNPSYHRLPVEDEVVRNSGSGEVHSPQLSGGAESGGHAPQPSXSCGMSMYSSQLPTDIIWAPTARQPPPPPY

>Mdp230041

MEEQNFTDYFPQTPLANSEPANATETFMESSTMNLGSNEVNGMKLLLESADEIGVRLEFDGDKTGVRFESDGDEHGMKKYKENXSRAAKEGLKETGRDIVVFGNNNGSATRPIILDSNNTNSKGKGKKIEHDDADSIAIIPNPSTLSHLSXETLPKRKRGRPKGSSNKLKPFASTGGFPVYPALGELMPHILTVKLGENILSQLLLLSQSTNRAMCILSAVGVGPFQILSLSGTFVYGSXRNPLEKSWMINVLLANHDGKAFGGSVAGFMIAAEPVQIVVGSFEQGTRKERKKSRRARSSYPSRLPGNGGITRATPLIMIPPKAKNDGICTSPASALLETPANSGSGHLIGANSINPASLPSFGQNVNPAXLSGFGQNVNPASPPDFGQNVNPASPPGFNLKFNPASLPGFGQNVPQSMLGPGTSIDFIPFVP

>Mdp231936

MEEQNFTDYFPQTPLANSEPANATETFMESSTMNLGSNEVNGMKLLLESADEIGVRLEFDGDKTGVRFESDGDEHGMKKYKENXSRAAKEGLKETGRDIVVFGNNNGSATRPIILDSNNTNSKGKGKKIEHDDADSIAIIPNPSTLSHLSXETLPKRKRGRPKGSSNKLKPFASTGGFPVYPALGELMPHILTVKLGENILSQLLLLSQSTNRAMCILSAVGVGPFQILSLSGTFVYGSXRNPLEKSWMINVLLANHDGKAFGGSVAGFMIAAEPVQIVVGSFEQGTRKERKKSRRARSSYPSRXPGNGGITRXTPLIMIPPKAKNDGICTSPASALLETPANSGSGHLIGANSINPASLPSFGQNVNPAXLSGFGQNVNPASPPDFGQNVNPASPPGFNLKFNPASLPGFGQNVPXSMLGPGTSIDFIPFVP

>Ppa18314m

MDPVAAHGRPLPPPFLSRDLHLHPHHQFQHHLHHNNHNSEDEQNSSGGGGLISRGIKRDRDENTSAATTSLEGKELGSTSAGEGEITRRPRGRPAGSKNKAKPPIIITRDSANALRSHVMEVANGCDIMDSVSTFARRRQRGVCILSGSGTVTNVTIRQPASPGSVVTLHGRFEILSLSGSFLPPPAPPAASGLTIYLAGVQGQVVGGGVVGPLLASGPVVIMAASFGNAAYERLPLEEEEPAAAGQVQGSGPLGSPGVGGQEHQQQSIQQQQQQLLQDPNAPSLFHGMPQSLLNSCQLPAEGYWGTARPPY

>Ppa09347m

MAGLDLGSASRFVHQQLQIPDLHLQRPPDSDDDHNTPNRNNLFSSDHHQNDVDDDNPHHQGLDLVTPNPGSGDSGGRRSRGRPPGSRNKPKPPVIITRESANTLRAHILEVSSGCDVFDSVGTYARKRQRGICVLSGSGMVTNVSLRQPAAAGAVVTLHGRFEILSLTGSFLPPPAPPGATSLTIFLAGGSGQVMGGNVVGALIASGPVIVIASSFTNVAYERLPLEEEDQLQMQQPVPQSSGGGGSGGGGGGGGVTNPFPDPSSGLPFFNLPLNNMPHQLQVDSWGGNSAGRPPY

>Ppa08610m

MANRWWAGNVAMGGGHVDSISSTPPSLHLRNTEEQLDDHNTTNTPTNSSTSNPATTPNKQNDEHHEDGRDNNDLEADNQDPNTGSGSHDSLEPGSSNRRPRGRPPGSKNKPKPPIIITKESPNALRSHVLEISSGSDIVDSIATFAQRRHRGVSVLSGSGIVANVTLRHPAAPSGVITLHGRFEILSLSGAFLPSPSPPGATGLTVYLAGGQGQVVGGTVMGALVASGPVMVVAATFTNATYERLPLEDEQAGEGGMQVQQQQQQQQQSGVNSAGTGGNSGSQGLVEHTSSMAIYNLPPNLLPNGQMPPDVFWGPPPPRPPPPSY

>Ppa20689m

MAGYNESLSTTSAPTASRYVHQLFHPDLHLQVHQQQQLHHHHQQQQSDDSHHQDDQDHNNNKIIESSDTAATSSGGGGDGDGGSGSGGPTRRPRGRPAGSKNKPKPPIIVTRDTPNALRSHVLEISAGADIMESVSIYARRRGRGVCVLSGSGTVANVTLRQPAGSVVTLHGRFEILSLSGTVLPPPAPPGAGGLSIFLAGVQGQVVGGCVVGPLLASGPVVLMAASFGNAVFERLPLDDPEEGTPTGGNGGGGGLQVQQPTASQSSGVTGGLGEGTGGNSGGGAGLFNLGGNMAANYPFSGPDLFGWGGGSTPRPPF

>Ppa19786m

MAGLDLGSAPRYVHQLHRQDLHLQQQQQTDSEDDAVVNRSTGQFSAEDHHQGLDLGGINPGSGDIVARRPRGRPPGSKNKPKPPVIITRESANTLRAHILEVGNGCDVFDCVATYARRRQRGICILSGSGTVTNVSLRQPAAAGSVVTLHGRFEILSLSGSFLPPPAPPGATSLTIFLAGVQGQVVGGSVVGELTAAGPVIVIASSFTNVAYERLPLDEEEQLQVQVPQGSGGSGGGGVGVGNNPFPDPSSGLPFFNLPLNMQNVQLPIDGWAGNNSGGRPPF

>Ppa18950m

MANRWWAGQVGLPGGVNETSAAATNSPMKNIIKPDLGISMNNNTTGTSSLGGSGGDDDDDRDNNSDDPKEGAIEVATRRPRGRPPGSKNKPKPPIFVTRDSPNALRSHVMEISNGADIADSVARFARTRQRGVCVLSGSGTVTNVTIRQASPAGSVMALHGRFEILSLTGAFLPGPAPPGSTGMTIYLAGVQGQVVGGSVVGPLVASGPVMVIAATFSNATYERLPLEEEEEVGGNNSGQAAGGGGSPPGIGGSGGGMGDPSIGVGVYNLQPNMLPNGGGQALSHDQGAAYSSWAHGQGGGRAPF

>Ppa19745m

MDSAATNSAALNKQRELEISINENNSGRSSGGDEDRDQDEPKEGAVEIGSRRPRGRPPGSKNKPKPPIFVTRDSPNSLRSHVMEVAGGADVAESVAQFARRRQRGVCVLSGSGSVANVTLRQPAAPGAVVALQGRFEILSLSGAFLPGPAPPGSTGLTVYLAAGNGQVVGGSVVGSLVAAGPVMVVAATFANATYERLPLEEDEEGGGSGGGGHNNGGSGNSPTAGGSSGAQLGSGGHQHQQQLPDPSSGGLPNIYSHHLPPNLIPNGGHGQLGHEAYAWARPPY

>Ppa22234m

MKAHNPATPSTTTFPKPIPKPSPTNHRECQTSEEERHPAATSSGTATVTTNPSAQNPKSSAAADPSNPSADGATIEVIRRPRGRPPGSKNKPKPPVIITRDSEPPMSPYILEVPGGSDIVEAVSRFCCRKNIGLCILTGSGTVANVTLRQPSTTPGATVTFHGRFDILSISATFLPQTTPSCPVSVPSGFTISLAGPQGQIVGGLVAGALVAAGTVYVIAASFNNPSYHRLPGEDEAVRNSGSGDAHSPPLSGGVESGGHAPPSSQSCGMSMYSCHLPTDVLWAPTARQPPPPPPY

>Ppa25027m

MAADYGGGVRAISLSQGGRNSDDDDGSSQHSPRSASGGVVVVGGGSSSSKSKFVSPSGEIIMSKKPRGRPPGSKNKPKPPIVITKNSEAAMKPVVLEISAGSDVIEAIIQYARRRHVGISVLSGSGAVSNVTLVHPAGPHHHPSAPSLLSLLHGPFNLLSLSGSFMDAVPATATCPSSASSKVATSAGGCSSPGSGSFGICLAGAQGQVFGGIVGGKVVAASAVVVVGATFVNPTVLRLPLPGDDHDDHENGVEGGAPAAEYEAQETKPGVNVESHCSVYGGGGGVASSTPMNCQITSVPDQVMPWGPPSRPPY

>Sm71342

MDPGLHGHHHQHHHHHHQFVSPLPLAQAGTSEEENSNSSGPGRGGPGGITTPTTIDKSRGNNKNLKTAATTSSSGGMGAGEIVRKPRGRPPGSKNKPKPPIIITRDSGNAMRPHVLEIAGGCDVGETLAAFARRRQRGLCVLGGSGTVANVTLRQLAAPGSTVTFHGRFEILSLSGAFLPPPAPVAVAGLTVALAGSQPGQVLGGSVVGVLMAASPVLVIAASFVGATYDRLPLEDEDPNQVASNNVSAASGVTSDPCSMALFNLAQNPLANCQLPPDVLAWAAAGNRPPF

>Sm67842

MLELHHQHQQQQQQQQQEIPPHQHSEDEDNSSSGNKELEHPHHHHLHHQIHHHHQQQQQQQQQQQQQQPQHQQSPQAQKSPGGGGGGGGGGGAGSGGEVVAPVRKPRGRPPGSKNKPKPPIIITRDTGSGMRPHVLEIAPNTDIVDAIATFARKRQRALCVLSARGTVSNLTLLRHSPASSTASAPPSSPPSSSAASTGATPSSSRAAAAATSTVSFQGRFELISLSGAFLQQQMPSAGILGAYSGLAVSVAGGPQGQVLGGNVAGPLVSASPVMVIAASFVGPAFDRLPLDDQDGEIPEEQPAAGAAANPSPNSTSGQLGLPQHGNSSPPSIPIHQLMNDPFGLAAAAAAAHHHHHHHQIPPPELLASWAAAAAAGATRPPM

>Sm404546

MAGIPGSFQHQLQLLHQQQQQQQQQQQQQQQHHQHHHLGGGGHSNDDDENHSNNSSGGSKGGGGVVGGGRINNNNSNSKGINNNNNNAALLQAGSSSSSLLLKDHSHSGGGGGGGGGAGGVGGGEVVVRKPRGRPPGSKNKPKPPIIITRDSGNAMRPHVLEIAGGCDVGETLAAFARRRARGLCVLGGSGTVANVTLRQLAAPGSTVTFHGRFEILSISGAFLPPPAPVAVAGLTVALAGAQQGQVLGGSVVGVLMAASPVLVIAASFVGATYDRLPLDESLETGGENPGVQGNHHLVNVSPGGPGGGSGGASQQEMALLNLAQNSLGQLPPEVLAWAAAGNRPPF

>TaAHL1/Taq1

MGSMDGHPLQGNHAYAHVPAGSNNDEDDASPPPSAGGGSSGSGRRPRGRPPGSKNKPKPPVVVTRESPNAMRSHVLEIASGADIVEAIAAFSRRRQRGVSVLSGSGAVTNVTLRQPAGTGAAAVALRGRFEILSLSGAFLPAPAPPGATGLAVYLAGGQGQVVGGSVMGELLASGPVMVIAATFGNATYERLPLDQDAEEGAVLSGSEGAATQLEQQGSGGAAVPPPMYAVPQTPPGDMFGQWGQAAVARPPPTSF

>TaAHL3/Taq3

MATGSSKWWQGPMDFPPQPQPQQMQQHQPLQHQHQPLQLPAVTMPAPAPAVAASPESKQQQQQGQGQGEGQMGAAAGAIVQLRRPGGRPMGSKNKPKPPIIITRDSPDALHSHILEVAPGADVAACVAEYARRRGRGVCVLGASGSVVDVVVRGAASAAPLPGRFELLSMTGTVLPPPAPSEASGLAVMLSAGQGQVLGGCVVGPLVAAGPVTLFAATFANAVYERLPLQDAADADVKPDLSAAPDASVPQEVQAQQPLAISQAMAMGAGYPDHRSPQYPWGGHQGGGI

>Mes030378m

MDPVAAHGRPLPPPFLTRDLHLHHHHQFQQHQQQNSEDEQNGNGSLNRGQKREHDETTADTAEGKELVPAGGDGEISRRPRGRPAGSKNKPKPPIIITRDSANALRSHVMEIANGCDIMESVSTFARRRQRGVCILSGNGTVTNVTLRQPASPGAVVTLHGRFEILSLSGSFLPPPAPPAASGLTIYLAGGQGQVVGGSVVGPLLASGPVVVMAASFGNAAYERLPLEEDEGQAPVPGSGPLGSPGSAGQPQQQQQQQQQQLMQDPNPSLFQGLPPNLLNSVQLPAEAYWGTARPPF

>Mes023060m

MDPVAAHGRPLPPPFHIRDLHLHPFHQFQQHQRQNSDEEQQSGNGSLNRGQKREHDDITNPTATATNTAEGKELVPATAGGDDEITRRPRGRPSGSKNKPKPPIIITRDSANALRSHVMEIANGCDIMETLSTFARRRLRGVSVLSGAGTVTNVTLRQPGSPGAVVTLHGRFEILSLSGSFLPPPAPPVASGLTIYLAGGQGQVIGGSVVGPLLASGPVVIMAASFGNAAYERLPLEEDEEQAPVPGSGPLGSPGIVGQPQQLPQQQQQQQLMQDPNPSLFQGLPPNLLSSVQLPAEGYWGTSRPPF

>Mes012990m

MDPVTAHGHSLPPPFHTRDFQLHQNQFPHHQQHNSEDEQSGSSSGAGLNKSQKRERDEISNNNGEGRELIPAGSGGEINRRPRGRPAGSKNKPKPPIIITRDSANALRTHLMEVADGCDIVESVATFARRRQRGISIMSGTGTVTNVTLRQPASPGAVVTLHGRFEILSLAGSFLPPPAPPAATGLTIYLAGGQGQVVGGSVVGTLTAAGPVVIMAASFSNAAYERLPLEEEDPQLPMQGGAIGSPGAVGQQQQQQQVLGEANAQLFHGLQPNLLNSIQLPTEPYWATGRPPY

>Mes013068m

MAGLDLGTTSRYVHQLHHRPDLHLQHQPEPEDHDSNRHGGGGVAAHFQSDHQHHDDASHQGLDLVAAAANTGPGDLVARRPRGRPPGSKNKPKPPVIITRESANTLRAHILEVGNGCDVFECVANYARRRQRGICILSGAGTVTNVSIRQPAAAGAVVTLHGRFEILSLSGSFLPPPAPPGATSLTIFLAGGQGQVVGGSVVGELTAAGPVIVIAASFTNVAYERLPLEEDEQLQMQSGGGSSGGGVGNNPFPDGAATSGGLPFFNLPLNMPPNVQLPVDGWAGNSGNRAPF

>Mes034268m

MAGGADLTVPSVGSKSIMDPNQESDKGNYHQSSIEAILTAPKLPKAESFVSQAPAGQTIKRPRGRPAGSKNKPKPPIIVTRDSANALRAHAMEVTSGCDVSESLVNFARRKQRGICVLSGSGCVANVTLRQPTSSGAIVTLHGRFEILSLLGSILPPPAPPGIAGLTIYLAGAQGQVVGGGVVGALIASGPVVIMATSFMNATFDRLPLDEDESATIVQNQHYQNGRHHHHHHLDISDLYGVPQNLLTNGTLPPEIYSWAPGRTMTKS

>Mes013326m

MANRWWAGQVGLPGMDTSTSSSSPMKKPDLGISMSNSNRETTESSRREEEKEEEREHSDEPKEGAIDVGTRRPRGRPPGSKNKPKPPIFVTRDSPNTLKSHLMEIANGSDIADSLACFARKRQRGVCVLSGSGMVTNVTLKQPSAPGAIMALHGRFEILSLTGAFLPGPAPPGATGLTIYLAGGQGQVVGGSVVGSLVASGPVMVIAATFSNATYERLPLEEEEEGGGTGSGGQGQIGGGEGGSGGMGDPAASTPVYNNLSPNLMPNGGQMNLEGYGWAHGRQPF

>Mes034448m

MAGLDLGTPSRFVHQLHQRPDLHLQHHLEPDDHDSNRHGGGGGVGAHFSTDHHHQQHEDGSNQGLDLVAGAGNSGPEDIGGRRPRGRPPGSKNKPKPPIIITRESANTLRAHILEVGNGCDVFECIANYARRRQRGICVLSGAGTVTNVTIRQPAAAGAVVTLHGRFEILSLSGSFLPPPAPPGATSLTIFLAGGQGQVVGGSVVGELTAAGPVIVIAASFTNVAYERLPLEEDEQLQMQNGDGSGEGGSGGGVGNNPFPDGAPTSGGLPFFNLPLNMQPNLQLPVDGWTGNSRAPF

>Mes012712m

MANRWWTGNFAMRDVDAISPAPSLHLRNLEEDGAGTASTATSTRTRNQDKEEQEDSKDYNHQESEDLNAAIGPIEPGNGSSSRRPRGRPPGSKNRPKPPVVITKESSSCLCSHVLEIGSGSDITESIATFAQRRHRGVSILSGSGIVTNVTLGQPAVPSGVINLHGRFEILSLSGSFLPAPSPPGATRLTVYLAGEQGQVVGGTVAGALVAAGPVMVIAATFSNATFERLPLEEQEHEGSQLQQQVNSGTNNNSNTTADGSGESSQPAAGEHDSMAVYNLPPNLMLNGQIPHDVFWSPPPS

>Mes011313m

MANRWWAGNVAMRGVDPISPAPSLHLRNPEEDTSCLNRLGRREQDFIDTNTTNSSNSPKTASTPTQNQNQNQEEQEDSKENNQESEDPNTALETVEPGSGSSGRRPRGRPPGSKNKPKPPIVITKESPNSLRSHVLEINSGSDIADSIATFAQRRHRGVSILSGSGVVTNVTLRQPAAPGGVITLHGRFEILSLSGSFLPAPSPPGATGLTVYLAGGQGQVVGGSVVGPLMASGPVMVIAATFSNATFERLPSEEQEQEQEGSQLQEQVNSGTNNNNNNTAAGGVGGNGNNNSGSQSSQPISEHGSMPVYNLPPNLLPNGQMPHEMFWGPPRHPPPNY

>Mes012157m

MKGEYGDPHHSSKHQNASPITMFSKLHHPHHLPFSHHFPLSRESEDDDARSTGALPVASLSPNTTTTDATPISATPIKKQKPAEPNSGDGATIEVIRRPRGRPPGSKNKPKPPVIITRDPEPAMSPYILEVPGGSDVVEAISRFCRRKNVGICVLTGSGTVANVTLRQPSTTPGSIITFHGRFDILSISATFLPQAVSYPVPNTFTISLAGPQGQIVGGLVAGSLVAAGAVYVIAATFDNPSYHRLPVEDEGRNSGSGGGGEGHSPSVSGAGGGGESGHTQGGGESCGMVMYSCHLPSDVIWAPTARPPPPPPPF

>Mes012278m

MKGEYVEAHHPPKHENVTPMNMFSKLHPHPHHQLPFSQHFQLSRESEDDETRSTGAAAVTTPSPNTNPATTTTPSQKQKPTEPNSSAGTDGASIEVVRRPRGRPPGSKNRPKPPVVITRDPEPAMSPYILEVPGGSDVVESISRFCRRKNIGICVLTGSGAVTNVTLRQPSTTPGSTITFHGSFDILSLSATFMPQPVSHPVPNTFTISLAGPQGQIVGGFVAGSLVAAGTVYVIAATFNNPSYHRLPGEDEGRNSGSGGEGQSPSVSGAGGGGGDSGHTQGGGESCGMVMYSCHLPSDVIWAPTARPPPPY

>Mes021597m

MADNVCDKTNTPSRELSHTSDGSYPDQSLLKLPANSSSSSPKPRSLDKPSPDDRIQSAKEVQRKPRGRPPGSKNKPKRPSITTDESESYLKPAILEISAGSDIIEAIIGFALKNDTCITLVSATGSVSNVTFRQQTPDVPPLSLHGTFNLIGLWGSFLGSFDPKNCSSDSSSLLSPSSFGISLAGPERQVFGGIVAGKVVAASIVVVVAATFRNPTFDRLPTDHDEAVETETGVYIPATDFLTDPGMPMAFYGEDIAVPMDCQMSPDILLWDFPPRPYF

>Mes033264m

MADYGTTISLSRELSHTSDASSSDHSPRSVAAVLSTPPSSSSSKRRTLNKPSPDNHHCIRSMVEVQRKPRGRPPGSKNKPKPPIIITKDTESAMKPAILEISAGSDIIDSIISFARRNRTGISIISATGSVSNVTLRQPIPHAPSLSLHGPFNLLDLSGSFLGSLALKQCSSAGSSSLHPSCCFGISLAGAQGQVFGGIVSGKVLAASQVVIVAATFLNPTFHRLPSDNDEAEETKPNVGGPANESCISSGMAVHGVSNPSLMNCQISPDIMHWGPPPRPHY

>Mes013183m

MANRWWAGQVGLPGMDHTSSSSSSPMKKPDLGISMSNNNREAIESGRREEEQEEEREHSDEPKEGAIDIATRRPRGRPPGSKNKPKPPIFVTRDSPNALKSHVLEIANGSDVAESLACFARKRQRGVCVLSGSGMVTNVTLKQPSAPGAVMALHGRFEILSLTGAFLPGPAPPGATGLTIYLAGGQGQVVGGSVMGPLMASGPVMVIAATFSNATYERLPLEEEEEEGGGERGGQGQIGGGEDGGSGGMGDPAAASTAVYNNLPPNLVPNGGQLNLEGYGWAHGRPPF

>Mes027401m

MAGAADLTVPSVGAKVVMDPSQESDKGNYRRSSIEAILMAPKLPKSLPPVSSAPEGETIRRPRGRPAGSKNKPKPPIIVTRDSANALRAHAMEISSGCDVSESLANFARRKQRGICVLSGSGCVTNVTLRQPASTGAIVTLHGRFEILSLLGSILPPPAPPGITGLTIYLAGAQGQVVGGGVVGALIASGPVVIMAASFMNATFDRLPLDEDEITTAVQNQHYQNGHHHLHRHLDISDLYGVPQNLLTNGSTPTEIYSRAPGRTITKS

>Mes031601m

MDPVTAHGHSLPPPFHTRDFQLHQHQFPHHQQHNSEDEQSGSSSGAGLNKSQKRERDETSNNNSEGKELIPTGSGGEINRRPRGRPAGSKNKPKPPIIITRDSANALRTHLMEVADGCDIVESVATFARRRQRGISIMSGTGTVTNVTLRQPASPGAVVTLHGRFEILSLAGSFLPPPAPPAATGLTIYLAGGQGQVVGGSVVGTLTASGPVVIMAASFSNAAYERLPLEEEDPQLPMQGGGIGSPGAVGQQQQQQQQALGEANAQLFHGLQPNLLNSIQLPAEAYWATGRPPY

>Mes013114m

MAGYEPTATGAGSRYIHQLLRPELHLQRPSISDQPSPDSKDNTSPQAKDHRAVDTDAAATSSGSNRRPRGRPPGSKNKPKPPIIVTRDSPNALRSHVLEVSTGSDIMESVSNYARKRGRGVCVLSGNGTVANVTLKQPASPAGSVVTLHGRFEILSLSGTVLPPPAPPGAGGLSIFLSGGQGQVVGGSVVGPLVASGPVVLMAASFANAVFERLPLDEEEGNVQVQSTASQSSGVTGGGGGQLGDGVGGGSGSGSGNGGAFFNMGGNVGNYPFSGDLFGWGGGSGARPPF

>Mes024479m

MAAGYEPTSTGAGSRYVHQLLRPDLHLQRLPFPAQSSPDSKGNTSPQAKDHKSDDTDVAATSSGSNRRSRGRPPGSKNKPKTPIIVTRDSPNALRSHVLEVSTGSDILESVFSYAMKRGRGVCILSGNGTVANVKLKQPASPAASVATLHGRFEILSLSGTVLPPPAPPGAGGLSIFLSGGQGQVVGGNVVGPLLASGPVVLMAASFENAVFERLPLEKEEGNVQVQSTASQSSGVTGGGGAGQLGDGGGGNEYPDLFGWGGSGAKPPN

>Al327335

MVLDMESTGEVVKSTASNGGGITVVRSDAPSDFHVAQRSESSKQSPASVTPPPSQPSSHHTAPPPPQISTATTTTAAMEGISGGLIKKKRGRPRKYGPDGTVVALSPKPISSAPAPSHLPPPSSNVIDFSASEKRSKMKPTNTFNRTKYHHQVENLGEWAPCSVGGNFTPHVITVNAGEDVTMKIISFSQQGPRSICVLSANGVISSVTLRQPDSSGGTLTYEGRFEILSLSGSFMPNDSGGTRSRTGGMSVSLASPDGRVVGGGLGGLLVAASPVQVVVGSFLAGTDQQDQKQKKNKHDFMLSNPTAAIPISSAADHRTIHSVSSLPVNNTWQTSLASDPRNKHSDINVNLT

>Al477717

MEPNESHHHHQQQLHHHHQQQQQQQHQQRLSSPYFHHQLQHHHHPTTVATTASTGNAVPSSNNGLFPPQPQPQHQPNDGSSSLAVYPHSVPSSAVTAPMEPLKRKRGRPRKYVTPEQALAAKKMASSASSSSAKERRELAAVTGGTVSTNSGSSKKSQLGSVGKTGQCFTPHIVNIAPGEDVAQKIMIFANQSKHELCVLSASGTISNASLRQPATAGVNLPHEGQYEILSLSGSYIRTEQGGKTGGLSASLSASDGQIIGGAIGTHLTAAGPVQVILGTFQLDRKKDAAGSGGKGDASNSGSRLTSPASTGQLLGIGFPPGMESTGRNPMRGNDEQQHHHHQPGLGGPHHFMMQAPQGMHMTHSRPAEWRGGGNSGLDGRGGGGYDLSGRIGHESSENGDYEQQIPD

>Al490548

METSDRISSGGVIGAEAPNQFMPSSGKKRRGRPRKYGEANGTPLPSSSTPLLKKRAKGKLNGFAIKMHKTINSSATGERFGVGGGAGSNFTPHIITVHTGEDITMRIISFSQQGPRAICILSANGVISNVTLRHPESCGGTLTYEGRFEILSLSGSFMETENQGSRGRSGGMSVSLAGPDGRVVGGGVAGLLIAATPIQVVVGSFITSDQQDHQIPRKQRVEHTPPTVTSLPPPPASVFSSTNPEREQPPSSFGISSWTNGQDMPRNSATDINISLPAD

>Al495229

MEEREGTNIRTSFGLKQHEAPLLPPPGYHMEPPRSENPNLFPVGQSSTSSVAAAAVKASENVAPPFSLTMPVENSSSDLKKKRGRPRKYNPDGSLAVTLSPMPISSSVPLTSELGSRKRGRGRGRGRGRGRGQGSREPNNDNNNNNWLKNPQMFEFNNTPSSGGGGPAEFVSPSFTPHVLTVNAGEDVTMKIMTFSQQGSRAICILSANGPISNVTLRQSMTSGGTLTYEGHFEILSLTGSFIPSESGGTRSRAGGMSVSLAGPDGRVFGGGLAGLFIAAGPVQVMVGSFIAGQEELQQQQQQQQIKKQRRERLGIPTTTQASNISFGVSVEDPKARYGLNKPVVIQPPPVSAPPVSFSHEPSTNTVHGYYANNTANHIKDLFSPLPEEDEEEDEEDLEGEDDEEFGGHSESDTEVPS

>Al496402

MEEKGGISPSGVVTVKEDEALVPRTEFQQNPSFLQFVSPTTVVSPLPLPPAPSPAPVPATVTPDSAAASTGSDPTKKKRGRPRKYAPDGSLNPRFSRPTLSPTPISSSIPLSGDYQWKRGKAQQQHQPLEFVKKSHKFEYGSPAPTPPPPGLSCYVGANFTTHQFTVNAGEDVTMKVMPYSQQGSRAICILSATGSISNVTLGQPTNAGGTLTYEGRFEILSLSGSFMPTENGGTKGRTGGMSISLAGPNGKIFGGGLAGMLIAAGPVQVVMGSFIVMHQAEQNQKKKPRVMEAFAPPPQQPPQLQQQQPPTFTITTVNSTSPATVEEPKQQPYGGGIVRPMAQMSSSFQNENSTMNNFTPPYHGYGNMNTGTNKDEHEDDDGGEDDDSGDTRSQSLSG

>Al945561

MEEREGTNINNNTSSFGLKQHEATASDGGYSKDPPPRPENPNPFLGQPTTVSAAATVAAVTENAATPFSLTMPAENTSSEQLKKKRGRPRKYNPDGTLAVTLSPMPISSSVPLTSEFPPRKRGRGRGKSNRWLKKSQMFQFDRSPVDTNLAGVGTADFVGANFTPHVLIVNAGEDVTMKIMTFSQQGSRAICILSANGPISNVTLRQSMTSGGTLTYEGRFEILSLTGSFMQNDSGGTRSRAGGMSVCLAGPDGRVFGGGLAGLFLAAGPVQVMVGTFIAGQEQSQLELARERRLRFGAQPSSISFNISAEERKARFERLNKSVAIPAPTTSYTHVNTTNAVHSYYTNSVNHVKDPFSSSIPVGGGVGKVGEEEGEEDEEDDDELEGEEEEFGGDSQSDNEIPS

>Al945842

MESTGEVVKTTTGSDGGVTVVRSNAPSDFHMAPRSETSNPPPTSVAPPPPPPPQKSFTPPAAMDGFSSGPIKKRRGRPRKYRHDGAAVTLSPNPISTAAPTTSHVIDFSTTAEKRGKMKPATPSSFIRPKYQVENLGEWAPSSAAANFTPHIITVNAGEDVTKRIISFSQQGSLAICVLCANGVVSSVTLRQPHSSGGTLTYEGRFEILSLSGTFMPSDSDGTRSRTGGMSVSLASPDGRVVGGGVAGLLVAATPIQVVVGSFLAGTNQQDQRPKQQNHNFMSSPLMPTSSNVADHRTIRPMPSSLPIRTWTPSFPSDPRHKLSHDFNITLT

>AtAHL1

MVLNMESTGEAVRSTTGNDGGITVVRSDAPSDFHVAQRSESSNQSPTSVTPPPPQPSSHHTAPPPLQISTVTTTTTTAAMEGISGGLMKKKRGRPRKYGPDGTVVALSPKPISSAPAPSHLPPPSSHVIDFSASEKRSKVKPTNSFNRTKYHHQVENLGEWAPCSVGGNFTPHIITVNTGEDVTMKIISFSQQGPRSICVLSANGVISSVTLRQPDSSGGTLTYEGRFEILSLSGSFMPNDSGGTRSRTGGMSVSLASPDGRVVGGGLAGLLVAASPVQVVVGSFLAGTDHQDQKPKKNKHDFMLSSPTAAIPISSAADHRTIHSVSSLPVNNNTWQTSLASDPRNKHTDINVNVT

>AtAHL2

METTGEVVKTTTGSDGGVTVVRSNAPSDFHMAPRSETSNTPPNSVAPPPPPPPQNSFTPSAAMDGFSSGPIKKRRGRPRKYGHDGAAVTLSPNPISSAAPTTSHVIDFSTTSEKRGKMKPATPTPSSFIRPKYQVENLGEWSPSSAAANFTPHIITVNAGEDVTKRIISFSQQGSLAICVLCANGVVSSVTLRQPDSSGGTLTYEGRFEILSLSGTFMPSDSDGTRSRTGGMSVSLASPDGRVVGGGVAGLLVAATPIQVVVGTFLGGTNQQEQTPKPHNHNFMSSPLMPTSSNVADHRTIRPMTSSLPISTWTPSFPSDSRHKHSHDFNITLT

>AtAHL3

MEEREGTNINNNITSSFGLKQQHEAAASDGGYSMDPPPRPENPNPFLVPPTTVPAAATVAAAVTENAATPFSLTMPTENTSAEQLKKKRGRPRKYNPDGTLVVTLSPMPISSSVPLTSEFPPRKRGRGRGKSNRWLKKSQMFQFDRSPVDTNLAGVGTADFVGANFTPHVLIVNAGEDVTMKIMTFSQQGSRAICILSANGPISNVTLRQSMTSGGTLTYEGRFEILSLTGSFMQNDSGGTRSRAGGMSVCLAGPDGRVFGGGLAGLFLAAGPVQVMVGTFIAGQEQSQLELAKERRLRFGAQPSSISFNISAEERKARFERLNKSVAIPAPTTSYTHVNTTNAVHSYYTNSVNHVKDPFSSIPVGGGGGGEVGEEEGEEDDDELEGEDEEFGGDSQSDNEIPS

>AtAHL4

MEEREGTNINNIPTSFGLKQHETPLPPPGYPPRSENPNLFPVGQSSTSSAAAAVKPSENVAPPFSLTMPVENSSSELKKKRGRPRKYNPDGSLAVTLSPMPISSSVPLTSEFGSRKRGRGRGRGRGRGRGRGQGQGSREPNNNNNDNNWLKNPQMFEFNNNTPTSGGGGPAEIVSPSFTPHVLTVNAGEDVTMKIMTFSQQGSRAICILSANGPISNVTLRQSMTSGGTLTYEGHFEILSLTGSFIPSESGGTRSRAGGMSVSLAGQDGRVFGGGLAGLFIAAGPVQVMVGSFIAGQEESQQQQQQIKKQRRERLGIPTTTQASNISFGGSAEDPKARYGLNKPVVIQPPPVSAPPVSFSHEPSTNTVHGYYANNTANHIKDLFSSLPGEDREEDEDDLEGEDDEEFGGHSESDTEVPS

>AtAHL6

MEEKGEISPSGVVTVKGDEALVPRTEFQQNPSFLQFVSPTTVVTPLPPPPAPSSAPVPTTVTPGSATASTGSDPTKKKRGRPRKYAPDGSLNPRFLRPTLSPTPISSSIPLSGDYQWKRGKAQQQHQPLEFVKKSHKFEYGSPAPTPPLPGLSCYVGANFTTHQFTVNGGEDVTMKVMPYSQQGSRAICILSATGSISNVTLGQPTNAGGTLTYEGRFEILSLSGSFMPTENGGTKGRAGGMSISLAGPNGNIFGGGLAGMLIAAGPVQVVMGSFIVMHQAEQNQKKKPRVMEAFAPPQPQAPPQLQQQQPPTFTITTVNSTSPSVNTVEEQKPQAYGGGIVRPMAQMPSSFQNDNSTMNNFTPAYHGYGNMNTGTTHKEEHEDEDGGDDDDDSGDTRSQSHSG

>AtAHL7

METSDRISPGGGIGAEVPSAYHMAPRPSDSPANQFMGLSLPPMEAPMPSSGEASGKKRRGRPRKYEANGAPLPSSSVPLVKKRVRGKLNGFDMKKMHKTIGFHSSGERFGVGGGVGGGVGSNFTPHVITVNTGEDITMRIISFSQQGPRAICILSANGVISNVTLRQPDSCGGTLTYEGRFEILSLSGSFMETENQGSKGRSGGMSVSLAGPDGRVVGGGVAGLLIAATPIQVVVGSFITSDQQDHQKPRKQRVEHAPAAVMSVPPPPSPPPPAASVFSPTNPDREQPPSSFGISSWTNGQDMPRNSATDINISLPVD

>AtAHL14

MDPNESHHHHQQQQLHHLHQQQQQQQQQQRLTSPYFHHQLQHHHHLPTTVATTASTGNAVPSSNNGLFPPQPQPQHQPNDGSSSLAVYPHSVPSSAVTAPMEPVKRKRGRPRKYVTPEQALAAKKLASSASSSSAKQRRELAAVTGGTVSTNSGSSKKSQLGSVGKTGQCFTPHIVNIAPGEDVVQKIMMFANQSKHELCVLSASGTISNASLRQPAPSGGNLPYEGQYEILSLSGSYIRTEQGGKSGGLSVSLSASDGQIIGGAIGSHLTAAGPVQVILGTFQLDRKKDAAGSGGKGDASNSGSRLTSPVSSGQLLGMGFPPGMESTGRNPMRGNDEQHDHHHHQAGLGGPHHFMMQAPQGIHMTHSRPSEWRGGGNSGHDGRGGGGYDLSGRIGHESSENGDYEQQIPD

>Bd3g02510.1

METKVETAPATTATASPAAQPAAPETASEVKPLAPAAVLPAVAGSAGRGEGKRKRGRPRKYGPDGGLVRPLKATPISASVPDDDGGGGRYTPAAAVGAVMKRGGGRPVGFVSRAAPVVPVTAAAPTAVVVVSPPPPPPAAANVQTHQQHGPPQGDLVGCASGANFMPHILNVAAGEDINMKVISFSQQGPKAICILSANGLISNVTLRQHDSLGGTVTYEGRFELLSLSGSFTPTDNGGTRDRSGGMSVSLAAADGRVIGGGVAGLLVAASPVQVVVGSFVPSSYHGANKPVIEMKTLAPQHQQPAVGFTISSGGDMDHQDSYSGAGSHPAAAKGNSTSAFRVENWTAAPTPPADEARRTPSTSSEAKVPVSGG

>Bd4g42740.1

MEGRMGIAVAGGGHEAAGLVMFRGDVSMAEAQQGGGEAGKVGYLSSPSSSPSTSLTPSPPPAEEPAGHGGYAAAAAPPPHAWSFGGEQEQEKPGETAGAGDDGNGNGGGGGSMQMAAHRGEHSAAGASSSGRRRGRPRGSGRRQILASLGEWFALSAGGSFTPHVIIVPRGEDVVTRIMSCSQKGPRSVCILSANGTISNVAINQPGSASGDTVTFEGLFEILQLTGSFTMAEEGRRRTGGLSVSLAHPDGRVFGGVVAGMLRAGTPIQVILGSFLPNSLKQHQRRMGLNQQPSTVPALPVIAAPPPVLTAAMPVSQAAAAAPPRMLTAAMPVSQAVAAAPHLTAAMPISQAAPGNGRNHVQPMSVAPLQAHQHGAATGMNLNSSSSGFTMVGWPVNSSTAQQPPMANRTSPDINLCLTPQE

>Bd5g19900.1

MDGREQQQQQARVSSPPAAGGVMMPQHAYGAAPAMPPGSANVMHGMPLGFNPMSSPGASSSMKPAEMPGAMYRPDSAPPGMQQTSGAGAIVVSGSGGGELVKKKRGRPRKYGPDGSIGYVPKPVAGATSEAGAGSNSNPDGKRRGRPPGSGKKKQLAALGSSGTSFTPHIITVKPNEDVASKIMSFSQQGPRTTCILSANGALCTATLRQPATSGGIVTYEGHFDILSLSGSFLLAEDGDTRSRTGGLSVALSGSDGRIVGGCVAGMLMAATPVQVLHSSVLCTLFSTGISQEALGDIAILCFRLWWAVSLLKVKSPRKNNRNASQVLRQCTLLALVHPQLPVLHPMEHQAITLMTRGAPWDLMAARSTTQAIPCKLHMLQRAGRFLGIKAAMIQT

>Cs251430.1

MEEKETGVSRFTVTNDEALDNFELAPRTEALKSTSESKVTEVAVAAPPPGFVAATDTPPVSVAVSSTETKKKRGRPRKYGPDGKRSLTLALSPMPISSSIPLTGEFPNWKRDNEISQAIVKKPQRFEFENPGQRLAYSVGANFTPHVITVNAGEDITMKVMSFSQQESRAICILSANGTISNVTLRQATSSGGTLTYEGRFEILALTGSYMPTQNGATKSRCGGMSVSLAGQDGRVVGGGLAGLLVAAGPVQIVVGSFLPGHQQEQKPKKPRNESTTIFFPPVNTITGEEMKAMYAGGNKPILTTPSYQEQHNPPSPTPVTGFKISSTDNLPLSDQEPKTQSQSNCEVSC

>Cs132930.1

MEPNENQLSSYFHHHQHHHQTPTTTSPTNGLLPPTHHLSAAAASSDAGPHVVYPHSVPSAAVSSSPLEPARRKRGRPRKYGTPEEALAAKKAATASSHSSSSKAKKELASSSSLNAVSASSSFSTPSKKSQLAALGNAGQGFAPHVINVAAGEDVGQKIMQFMQQCKREICILSASGSISNASLRQPAASGGNIAYEGRFEIVSLCGSYVRTDLGGKTGGLSVCLSSAEGHIIGGGVGGPLKAAGPVQVIVGTFVIDPKKEFGGGKGDGSAVKLPSPIGGTSMSNLRYGSNIDSGGNQIRGNDEHQGLGESHFLLQPRGVNLTSPRSTDWRTGLDATNTAYDLSGRTGHHSPENGDYDQIPD

>Cs302370.1

MEGRDGGGASSGVTVVGSDAPSEYKIAPRTSDNPPQTGGSTTPPGTQSTSTPSASAQVSGQPPPPTAASSVPGKKKRGRPRKYGPDGSVSMALSPKPISLSVPPPVIDFSTEKKGKVRPASAVSKSKFEVDNLGDWVPCSLGANFTPHIITVNAGEDVTMKIISFSQQGPRAICILSANGVISSVTLRQPDSSGGTLTYEGRFEILSLSGSFMPSDNGATRSRSGGMSVSLASPDGRVVGGGVAGLLVAASPVQVVVGSFLSGNQHEQKPKKPKHDTISPAPPTAAIPISCVDPKSNLSPSSSFRGDNWSMLPTDSRNKSTDINVSLPSA

>Cs005230.1

MSGSETGVITSREPFGVGVQNSSLHSQSGTQNMRLAFGADGTGYKPVTPSTSPSYQSSMAGVSGNAGIEGSAGGGGGGGSMLPHGFNINSVGSEQIKRKRGRPRKYGPDGSMALALGSGPPSGTGCFPPSNMANSASEALGSPNSSKKTKGRPLGSKKKQQLEALGSAGIGFTPHVIDVKAGEDVSSKIMSFSQNGPRAICILSANGSISNVTLRQPATSGGTVTYEGRFEILSLSGSFLLSENGGQRSRTGGLSVSLSGPDGRVLGGSVAGLLTALSPVQVVVGSFIADGNKEPKPARQNELTTALPMLNTAGFGHLTGGASSPSHGTLSESSDGSPDSPLNNSSGGCNNSNHPQGMSGMPWK

>Gm05g04040.1

MEASGGVSSGVTVVGSDAPSDYHVAPRTDNPAPASGSTTQIPATAGSALSPSHPPHTAAMEAYPATMPAKKKRGRPRKYAPDGSVTMALSPKPISSSAPLPPVIDFSSEKRGKIKPASSVSKAKFELENLGEWVACSVGANFTPHIITVNSGEDVTMKVISFSQQGPRAICILSANGVISSVTLRQPDSSGGTLTYEGRFEILSLSGSFMPNESGGTRSRSGGMSVSLASPDGRVVGGGVAGLLVAASPVQVVVGSFLAGNQHEQKPRKQKHEVISSVTPAAVVPISTLDPVSILSAASSIRNDNWSAMPAEAKDKPADINVSLPAG

>Gm17g14520.1

METSGGVSGGVTLVGSDAPSDYHVAPRTDNPAPASGSTTQIPATAGSVPPSPHPPHTAAMEAYPAKMPAKKKRGRPRKYAPDGSVTMALSPKPISSSAPLPPVIDFSSEKRGKIKPTSSVSKAKFELENLGEWVACSVGANFTPHIITVNSGEDVTMKVISFSQQGPRAICILSANGVISSVTLRQPDSSGGTLTYEGRFEILSLSGSFMPSESGGTRSRSGGMSVSLASPDGRVVGGGVAGLLVAASPVQVVVGSFLAGNQHEQKPRKQRHEVITSVIPAAVVPISTLDPVPILSAASSIRNDNWSAMPAEAKNKPADINVSLPAGFVFM

>Gm01g34410.1

MEAREGISSGVTVIGAEAPSAYHVAPRSEAPNQVHVPDGGGAAATAAPVGVSPVSAGLDGTAVKKKRGRPRKYGPDGSVTMALSPMPISSSAPPSNDFSSGKRGKMRGMDYKPSKKVGLDYLGDLNACSDGTNFMPHIITVNAGEDITMKVISFSQQGPRAICILSANGVISNVTLRQPDSSGGTLTYEGRFEILSLSGSFMPTDNQGTRSRTGGMSVSLASPDGRVVGGGVAGLLVAASPVQVVVGSFLPSSQQEQKIKKSKSSDYGVATVTPTIAVSPTPPPPTNAEKEDVNVMGGAHVLQNSGTLNSNLTPPNAFRRDNWVNMHSMPDSRKSATDINISLPDS

>Gm03g02670.1

MEAREGISSGVTVIGAEAPSAYHVAPRSEAPNQVHVPDGGGGAAATAAPVGVSPVSVGLDGTVKKKRGRPRKYGPDGSVTMALSPMPISSSAPPSNDFSSGKRGKMRGMDYKPSKKVGLDYIGDLNVCSDGTNFMPHIITVNAGEDITMKVISFSQQGPRAICILSANGVISNVTLRQPDSSGGTLTYEGRFEILSLSGSFMPTDNQGTRSRTGGMSVSLASPDGRVVGGGVAGLLVAASPVQVVVGSFLPSSQQEQKIKKPKSSDYAPVTVTPAIAVSSAPPPPTNAEKEDVNVMGGAHVLQNSGTLNSNLTPPNAFRRDNWVNMHSMPDSRKSATDINISLPDS

>Gm03g41230.1

MEPNDNQLTSFFHHHHQQHQHHQPPPPPQTTASPTNGLLPNADGSHILYPHSVASAVSSQLEPAKRKRGRPRKYGTPEQALAAKKAATTLSHSFSVDKKPHSPTFPSSKKSHSFALGNAGQGFTPHVISVAAGEDVGQKIMLFMQQSRREMCILSASGSISNASLRQPATSGGSIAYEGRFEIISLTGSYVRNELGTRTGGLSVCLSNTDGQIIGGGVGGPLKAAGPVQVIVGTFFIDNKKDTGAGVKGDISASKLPSPVGEPVSSLGFRQSVDSPSGNPIRGNDEHQAMGGSHFMIQQLGLHGTPPRSTDWGHPDSRNTGFELTGRIGHGAHQSPENGGYEQIPD

>Gm09g28080.1

MEEREIFSSGHAVKVVEAPQSFHVALNSVQFSGPTVESPVPSPVPASAPAPAPVSASVMNSGSTEGKKKRGRPRKYGPDGKVALSPMPISASIPLTGDFSAWKRGRGKPLESIKKSFKFYEAGGAGPGDGIAYSVGANFTPHILTVNEGEDVTMKIMSFSQQGCQAICILSANGTISNVTLRQPTSSGGTLTYEGRFEILSLSGSYITTENGLTKSRSGGMSISLAAPDGRVMGGGLAGLLVAAGPVQVVVASFVPGHQLEQQKPKKPRVEHISMAAPTYVNPTSAEEIRIGLGGVKPIMTPAAFQVHHIFGNGQSSGNSASDDSAPFPENESNPSHADAGVAC

>Gm09g40520.1

MEGREGISSGVTVIGAEAPSAYHMAPRSEAPSQVPPPVPEATAGAIGVSPASVGLDGTAAKKKRGRPRKYGPDGLNSMALSPIPISSSAPFANEFSSGKQRGKPRAMEYKLPKKVGVDLFGDSVGTNFMPHIITVNTGEDITMKVISFSQQGPRAICILSASGVISNVTLRQPDSSGGTLTYEGRFEILSLSGSFMPTDNQGTRSRSGGMSVSLSSPDGRIVGGGVAGLLVAAGPVQVVVGSFLPNNPQDKKPKKPKSDYAPANVTPSIAVSSAPPPTNGEKEDVMGGHLLHNNSGTTLNSNFSPPSAFRRENWVNMHSMADSMKLVTDINISLPDS

>Gm18g45300.1

MRMEGREGFSSGVTVIGAEAPSAYHMAPRSEAPSQVPPPVPEATAGAIGVSPVSVGLDGTAAKKKRGRPRKYGPDGLNSMALSPMPISSSAPFANNFSSGKRGKSRGMEYKLLKKVGVDLFGDSVGTNFMPHIITVNTGEDITMKVISFSQQGPRAICILSASGVISNVTLRQPDSSGGTLTYEGRFEILSLSGSFMPTDNQGSRSRSGGMSVSLSSPDGRVVGGGVAGLLVAAGPVQGLQGWIQNSLQHPISSVMLKTLPNNLPQMHPQAIEGFQMKEAELHA

>Gm10g07550.1

MESAATALNNIINNNNTVPAVSPPPPPPPSSEPQPMNMNVNMNMNVVNTTEGTTPTPTPTTMVPPTTMVVPAVPAPTTTTLAVATTTPGSLDLFGKKKRGRPRKYDADGNLRVSARPTPTPPSGFTLSTPSEYSSSKRERGKHYNTTFANNSYQQQLYSSSLGDVFAITAAGDFVAHVLNAYTGEDVAGKILSFAQKGPRGICILSANGAISNVTIRQPGSSGGILTYEAWTALGAFMCQVISVCLFLWGWTKYLSLPHVLDICSKLVYWVYVKKLVYLFISSVPVSDLTLEMGNKGRFEILSLSGSFTVVDNSGMKSRTGGLSVSLAGPDGRVIGGGVAGLLTAAGPIQIVVGSFMQNCCKTQKRKYQREQQIVAATPTSAGPEIVTAAIPISQANAADGENFLIPIPIYQIPDQNQRESISVSSDKQNLDATPDAAATWNGSEEYSDQRTSPDINISLPDE

>Gm10g32150.1

MEGRENFGVVVGDEAPESFHVAPRIENNLDFSRATVPAPAPPTEGKKKRGRPRKYGPDGKPALGAVTALSPMPISSSIPLTGEFSAWKRGLYRGWGGGGGGSIWYSLFSIMRRRGLHYLTKETVESKWRDREGIAYSVGANFTPHVLTVNAGEDVTMKIMSFSQQGSRAICILSATGTISNVTLRQPSSCGGTLTYEGRFEILSLSGSFMPTENGVTRSRSGGMSVSLAGPDGRVMGGGLAGLLVAAGPVQVVVASFLPGHQLEHKTKKQRVGHVSTISPSPVNLITSEEIIVSFGGVKPIMTPAAFQEENIASFNNGQDYRNSSVDDKDPLPEKESNLSQSNAEAVC

>Gm13g21430.1

MEPTTTLNNIINNNNTVPAGSPPPPPPPSVPQPMNMNVNMNMNVGNTEGTTPAVPTPTPAPTPAPTTMVPAVPAPTTTTPGSLDLFGKKKRGRPRKYDADGNLRVSATPPPPPGFTLSTPSSEFSSSKRGREKKLFFSFGSTSTRLFQDYCYAHTKSKIVLCWVFANTAGGDFVPHVVTVYTGEDVAGKIVSFAQKGPRGICILSANGAISNVTIRQPGSSGGILTYEACLFMSSANIEYGCRIGQHWVNLCQVISVCLFLWGWTKYLYLPYVLDIRSKLVYWGRFEILSLSGSFTVADNSGMKSRTGGLSVSLAGPDGRVIGGGVAGLLTAAGPIQIVVGSFMQNGYKAQKRKYQREQQIVATPTSAGPEIVTAVRPISQTNADGENFLIPMSQMPDQNQRESVSVSSDKQNLDATPDAATWNGSEEYSDQRTSPDINISLPDE

>Gm16g32940.1

MEEREIFSSGHAVNLLEAPHSFHVALNSVQFSGPTVETPAPAPAPAPAPAPAPVPVIAPVMNSGSTEGKKKRGRPRKYGPDGKVALSPMPISASIPFTGDFSAWKRGRGKPLESIKKTFKFYEAGGAGSGDGIAYSVGANFTPHILTVNDGEDVTMKIMSFSQQGYRAICILSANGTISNVTLRQPTSSGGTLTYEGRFEILSLSGSYITTENGLTKSRSGGMSISLAGPDGRVMGGGLAGLLVAAGPVQVVVASFLPGHQLEQQKPKKPRVEHIISMAAPMHVNPTSAAEEIRIGLGGVKPIMTPAAFQVDHIFGNGQSSGNSASDDSASFPENESNPSHADAGVAC

>Gm19g43850.1

MEPIDNHLTSFFHHHQQQQQHHQHQHQHPPPPPPTTASPTNGLLPNADGSHMLYPHSVASAVSSQLEPAKRKRGRPRKYGTPEQALAAKKAATTSSQSFSADKKPHSPTFPSSSFTSSKKSLSFALGNAGQGFTPHVISVAAGEDVGQKIMLFMQQSRREMCILSASGSISNASLRQPATSGGSITYEGRFEIISLTGSYVRNELGTRTGGLSVCLSNTDGQIIGGGVGGPLKAAGPVQVIVGTFFIDNKKDNGAGLKGDASASKLPSPVSEPVSSLGFRQSVDSSSGNPIRGNDEHQAMDGSHFMIQQLGLHGTPPRSTDWGRPDSRNTGFELTGFLSAGRTGHGAHQSPENGGYDQIPD

>Mt5g011870.1

MKGASDTPTDYPAAPRTRIPDFASGPAADSTSQGGIPPMQPVAPAKKKRGRPRKYRPDGSLSLAIPPKPTSSSIGEAAKFELENPGSRMLNYVVVSSSLGNEQSEQMLKTQENEVTPTSTPTAAPPVSTAGQLPASSVSATFTPHIIIVNAGEDVPMKIMSFCQQGPEAICILYVNGVISKVVISRPQSSRTLFTYEDLRCTLLIICCFCVILSIMQIEVHGRYEIRTLSGSFMPKEKCGRRSISGGMSVSLVDLHGHVVGGRVAGPLVAASPVNVVVGSFLPSEHEQKLKTQNNEVISTPAAPMSTAGPNT

>Mt8g106590.1

MEASGGVSGGVTVVGSDAPSEYHVAPRTDNQTPTTGSAVQLLAAVQAGAPPQQPPYTAVLTAAPAVTTVPEKKKRGRPRKYAADGSVTAALSPKPISSSAPLPPVIDFTAEKRAKVKPVSSVSKANFELENIGEWVPCSVGSNFTPHIITVNAGEDVTMKVISFSQQGPRAVCILSANGVIKSVTLRQPDSSGGTLTYEGLFEILSLSGSFMPNESGGGTRSRSGGMSVSLASPDGRVVGGGVAGLLVAASPVQVVVGSFMAGNQHEQKPRNQKHDVVSTVSSPAVVPIPTLDPIPILSSVTSFRGDNWSAVPASDAKDKPATDINVSLPGG

>Mt7g034750.1

MEERENFGAVNVVGGNEVPVSFQAAPRIDNNLDFSMATVPVSLPETALKKKRGRPRKYGPDGKPAPGAVTALSPMPISSSIPLTGEFSAWKRGRGKPVESMKKSSFKFDFESPPVQVVGGGVSEGIAYSVGANFTAYVLTVNSGEDVTMKIMSSQQGSRAICILSATGTISNVTLRQSTSSGGTLTYEGRFEILSLSGSFMPTENGITRSRSGGMSVSLAGPDGRVLGGGLAGLLIASGPVQVVVGSFLPGHHLEHNSKKQRVEHTISTITHNNPPPHVNHHVSNEVSFGGVKPFMTPAAFQEENIVSFNNNAQDSRNTSSAEDRDPLPEKDSNHSQSTT

>Mt1g073540.1

MHNNNVSVGSPQQLVTTNTLPQEMNMNISVGNSIVTTIGTSIVALEAPTVTPTTTTPGSLDLFGKKKRGRPRKYDADGNLNPSYKKIVKTTTPILTSPPGFTLSTNEFASKKGRGKSTGFVNYQTFSSFVRVQGEVFPSTAAVDFAPHVVTVYAGEDVGGKILSFAQKSPRGICILSANGAISKVALGQPGSTGVNSKKQCNGKAYHRQCPLAREVVTQGRFEILSLSGSYTASDNSGIRTREGGLSVSLAGPDGRVIGGAVAGVLIAAGPIQV

>Mt1g093350.1

MEDTNNHNNQVTSLFEQQSTVNVTPELNGTTTDGSHHSVTENKKKRGRPRKYESPEEAIAGRKAIAARKAAAAAAAAANATATTSFSSPNFTKPKKFHSSSLGNSREGFNIHFVTVAPGEDIGQNIMMLMQKNSRCEMCILSASGSISSATLRQPATSGGNITYEGRFDIISLTGSYVRNELDGRSGGLSVCLSHSDGQLVGGSIAGPLKAASPVQVIAGTFSIPSKDPGAGIKGDVSTSKLPSPVGEPTSNLGFRPAINSSNGNAIPGNKEHQVIGGSHFMPQQYGVNVVPFHPSDWGSRPDSRNAGFELIGRTGHGANHSPENGNYS

>Mt1g046700.1

MDSHELQQPHPRPHHLQLENVVLDSPNPVTNTVPLAMMEPTIFPQLNMNTNQPPHFEPFNNNIVPSTLKQCVGASSGSGSIQKKRGRPREYFLDGYIASIAKRRVTGTDFSQHVITVNPGDDIVAKLKTCCQGGPNTEMCILSAHGLVGTVALHQPGRIFICEGQFEILSLSGMLEVFDNNNGFKRMNYFTVSLVEPNSNVFGGVVDKLIAASLVKVKVACFTLDDKNGSSSNLNLGPSSIPISQFAAFGTPTSATTQGPSSISLSNNENIPLGLGHGIYGNDSQPIPTLSMYQQTLARQTQQ

>Mt6g067180.1

MEEREIFGSGHAVNVNQAPPGFNLAQNTLNFAGSTGELPAPVPVAGGVEVKKKRGRPRKSESGSKPALSPMPISASIPLTGDFSGWKSGGGGGGGVVKPFESIKKPLKLNDFDEDNGISPFGSNFKTHVLTVNSGEDVSMKIMSLSQQEYHTISILSATGTISNVTLRQSDACGGTSTYEGVFEILSLSGSFVPTENGLTKSRSGRMSVSLAGPNGRVFGGALAGLLVAAGSVQVVVASFFPEKENPKRQRVDHSAPTAPPTSSHINNHVSAEELRTDIGGMKPIMSPAGFNFASFGNGQGSGNSSSSADDEHV

>Os03g01540.1

MDESTAYRNLRGPSPFVLAMKTRLAPPPQLSDPPPPPLPAVPPSLLPPLLPPPEKRRRGRPRNCDRLTAPPGFFAPLPPPPPPQPQPPTLPAPHGQGQFGGLQPHLLQIDAGEEIIPKITALSKSNGRVICVLSVLGAVQEATLLLSSGVTSYHKGPLEIIRLFGSILTPNDQGCLRVTLASGDSSVIGGVITGPLKAATPVQVVVASFYSDVYWPNRTPKIIAPYPNSQSTIRNGSTLSSEHVNPGYVSYTAVDQHESSEVDVKPSLGMLNLASLDRHESSEVDVKPSLGMLNLASL

>Os11g05160.1

MHMEGGEGIAVAGAGGGHEAGFGLFRAADVTMTEAQEAAKEYQSSPSSPSTSPTPSPPPVAASGHGGEAAATPTMWSLGGEKMPSEAAGDNGMQMSGHSEHASLSSGRRRGRPKGSGRRQILATLGEWYALSAGGSFTPHVIIVGTGEDVAGRIMSFSQKGPRSICILSANGTISNVALSQPGSSGSTFTYEGRFEILQLTGSFTMAEEGGRRRTGGLSVSLAGPDGRVVGGVVAGMLRAASPIQVIVGSFLPNSLKQHQRRMGLQQQPSAAPALPPPMAPPPVLTAAMPISQAAPGTNGCHAPQVSSMHPQAHTGVMEHSATASGAMNLNSSSSTGFTMVGWPVSSQSMGHRPSPDINVCLTPQE

>Pt02s14950.1

MEGREGLSAGVTVIGAEAPSNYHVAPRTENPGQFVVSPPAVEVPPVGAGLTGGTTEKKKRGRPRKYGPDGAVARALSPMPISASAPSPGGDYSAGKPGKVWPGSYEKKKYKKLGMENLGEWAANSVGTNFTPHVITVNAGEDVTMKVISFSQQGPRAICILSANGVISNVTLRQPDSSGGTLTYEGRFEILSLSGSFMPTESQGTRSRSGGMSVSLASPDGRVVGGSVAGLLVAASPVQVVVGSFLAGNHQDQKPKKPKIDSIPATFAPAPVIPVSIAEREESVGTPHGQQQNSSSFQRENWATMHSMQDVRNSVTDINISLPEG

>Pt19s02230.1

MELNDPRQQQHHHFTSYFSSTPTTTNTPSPPNGLLPPHHPTDSTTPTGSHLLYPHSMGPSTTATVTGGGAPVEATSAKRKRGRPRKYGTPELALAAKKTATSASVAASRERKEQHQAGSSSTTSSFSGSSSKKSQHVLGTAGHGFTPHVITVAAGEDVGQKIIQFLQQSTREMCILSASGSVMNVSLRQPATSGGNISYEGRFEIISLSGSYIRTDMGGRAGGLSVCLSDSNGQIIGGGVGGPLKAAGPVQVIVGTFVLDNKKDGSGKGDASGSKLPSPVKASVPSFGFRLPVESSVRNPARGNDDLLTVGGGNPFTMQPSTMHLLSARTMDWRSSPDVRTTAGYDFTGRTGHGGSQSPVNGDYD

>Pt13s04130.1

MEPNDSHQQQQHFTSYFSTTTTTTTTTTPSPTNGLLPPHQPTDSTTPTGPHMLYPHSMGPSTTATVTGGGGAPVEAAAAAAAAKRKRGRPRKYGTPEQALAAKKTASSNSAAAYREKKEHQAGSSSTISSFSAYSSKKSQHASLGNAGHGFTPHVITVAEGEDVTQKIMHFLQQSMREMCILSASGSILSASLSQPATSGGNISYEGRYEIISLCGSYVRTEMGGRAGGLSVCLSDTNGQIIGGGVGGPLKAAGPVQVIVGTFMLDNKKGGSGKGDASGSKLPSPVGASVPSFGFRSPVESSLMNPARANDDHPTIGGNPFTMQPTSMHLTPTRPIDWMSGPDVRTSGYDFTGRTGHGGPQSPENGDYE

>Rc34.m008894

METREGLTSGVTVIGAEAPSTYHVAPRTENSSQIAGSPAVAMSQASLGLTGTTEKKKRGRPRKYGPDGTVARALSPMPISSSAPPGGDFSSGKPGKVWSGGFEKKKYKKMGMENSGDWASGSVGTNFTPHVITVNAGEDVTMKVISFSQQGPRAICILSANGVISNVTLRQPDSSGGTLTYEGRFEILSLSGSFMPTESQGTRSRSGGMSVSLASPDGRVVGGGVAGLLVAASPVQVVVGSFLPGNHQDQKPKKIKIDPVPASITPAQTIAIPIPVTNAERDDSMGGHGLQNSSSFRRENWTTMQPVQEMRTSGTDINISLPEG

>Rc20.m70

MEPNDTQHHHHPHHHLSSSSYFTTSTTPAPATTTPSPTNGLLPPPPHDTGGGGGTHMVYPHSVGPSTAAVSSAPVESPRRKRGRPRKYGTPEQALAAKKTASSSSNAVAARERREAAAASSPSYSGFSSRKSQQLVALGNAGQGFTPHVISVSAGEDVAQKIMLFMQQCRREMCILSASGSISNASLRQPATSGGNITYEGRFEIISLSGSYVRTEIGGRAGGLSVCLSNSDGQIIGGGIGGPLIAGGPVQVIIGTFVVDNKKDVGSGGKVDASSSKLPSPGGGASMSNIGFRTPTDTSGRHTFRGNDDHQTMGGNPFMIPPRGMHDWSSGSEARVNATFELTGRRGHGARQSPENGDYEQYPD

>Vv01019937001

MMPAKKKRGRPRKYGPDGTVTMALSPKPISSSAPGPPVIDFSVEKRGKIRPVGSASKSKMELENLGEWVACSVGANFTPHIITVNSGEDVTMKIISFSQQGPRAICILSANGVISSVTLRQPDSSGGTLTYEGRFEILSLSGSFMPSDSGGTRSRSGGMSVSLASPDGRVVGGGVAGLLVAASPVQVVVGSFLTGNQHEQKPKKQKIEPISAARPTAAVPISTTPKPNLSSSTSFRGDNWSPLPSDSRNKATDINVSLPGG

>Vv01036214001

MEPNDTRLTSYFHHHQQQPQPPPPPPPQPQPHHQTQNPVAATAASPSNGLLPPSERPPLGYHHSVPSAVTSPPETVRRKRGRPRKYGTSEQGLSAKKSPSSSVPVPKKKEQGLGGSSKKSQLVSLGNAGQSFTPHVITVASGEDVAQKIMFFMQQSKREICIMSASGSISNASLRQPATSGGNVAYEGRFEILSLTGSYVRTEIGGRTGGLSVCLSNTDGEIIGGGVGGPLKAAGPVQVIVGTFLVDSKKDTSTGLKADASPKFTSPVGGASVSNVGFRSAVESSGRIPVMGNDDHQGIGGSHFMIQSRGMQMAPTRPTDWRSGPDARINVGYDLAGRGGRGACQSPENGDYEQIPD

>Zm2G006252_T01

MQMEGREGIAVGRESGHGSMFRAADIAMAEAQEAAAKGYQSSPSSPSTSPTPSPPAAAAGHAGDAAATPLAWSLGGETPSGAVVVDNGTQTAGQSEHASLSSGRRRGRPRGSGRRQILATLGEWYALSAGGSFTPHVIIVGTGEDVAARIMSFSQKGPRSVCILSANGSISNVTLRQPDASGSTFTYEGRFEILQLMGSFTMAEEGRRRTGGLSVSLAGPDGRVVGGVVAGMLRAASPIQVIVGSFLPNSLKQHQRRRMSMQQPSPVPALPAPVAPPPPVLTAATPISQAGPGNGFHAPPPPHSAAPPQPHANAMNLNATTTGFTMVGWPASSPQPMAYRASPDINVSLTPQE

>Bra019359

MESTGEVVRTSDVSDGGVMVVRSNAPSDFHMAPRSETSNPPPASVSPPQNSFAPTAPPPTTEGFSRGPMKKKRGRPRKYGHDGAPVALSPNPISSAAPTTSHVIDFSASEKRGKVKPAATPSSFIRTKYQVENLGEWAPSSAGANFTPHIITVNAGEDVTKKIISFSQQGSLAICVLCANGVVSSVTLRQPDSSGGTLTYEGRFEILSLSGSFMPSDSDGTRSRTGGMTVSLASPDGRVVGGGVAGLLVAATPIQVVVGSFLAGTNQQDQNPRKQNHNFVSSPMPTTSNAADHGTIRPTSSSHQIGTWTPTLASDPRHKPSHDINITLT

>Bra028265

MEEREGTSNNIISSFGLNQHVAPPPPTGGGGVYHMDPPRSENPNPFPVGLPNTTAASASASVAAAKASENAAPPFSLTMPVENTSSELKKKRGRPRKYNPDGSLAVTLSPMPISSSVPLTTEFSSRKRGRGRGRGRGRGRGRGLVEPPINNNNWVKNPQMFEFDNSSPVVRTPEVVTSASFTPHVLTVNAGEDVTMKIMTFSQQSSRAICILSANGPVSNVTLRQSMTSGGTLTYEGHFEILSLTGSFIPSESGGTRSRAGGMSVPLAGPDGRVFGGGLAGLFIAAGPVQVVVGRFVAGQEESQQQQQQQQMKTQRRERFGIPTSTQASNISFGGGSAEDPKARYGLNKPVVIQAPPMSAPPVPFSQHEPQPSTNAVQGYYTNNTAEQIRDLFSSLPGEDDDEDDDLEGEDGDDGEFGDHSESDTEVPS

>Bra040133

MEPHESHQQQRLSSPYFHHHLQHHHHHPSTVSSANAVPPPNNGLFPPPPPPQANDGSSSHAVFPLSSAVTAPSEPLKRKRGRPRKYVTPEQALEAKKMASAASSSSAKERREQAAGGTTTSSSPGSSKKSHLGSVGKTGQSFTPHIVNIAPGEDVAQKIVMFAQQSKHELCVLSASGTISTASLRQPGGNISYEGQYEIISLSGSFIRSEQGGKTGGLCVSLSRSDGQIIGGAVGTHLTAAGPVQVILGTFQLDRKKDGVKGEASNSGSRLTSPSSTGPGPGPGPLLGMGFRCVMESPGRNPMRGNDEQHHHHQQTGMGGSHHHFMMQAPQGMHMTHPRPSEWGGGGGSSGHDGRGGGGGYDLSGRLGHESFENGDDEQQMPD

>Bra029164

MEEREGTSNMITSFGLKQHVAPPPPPSGGGVYQMDPPRSENPNPFPAGLPNTTAAAASASAVAAKATENAAPPFSLTMPVENSSAELAKKKRGRPRKYNPDGSLAVTLSPMPLSSSVPLSTGFASQKRGRGRGRGRGRGRGRVEQPPNNNSWVKNPQMFEFQNSSPVVGAADVVTSASFTPHVLTVNAGEDVTMKIMTFSQQEGSRAICILSANGPISNVTLRQSTTSGGTLTYEGHFEILSLTGSFIPSESGGTRSRAGGMSVSLAGPDGRVFGGGLAGLFIAAGPVQVMVGTFVAGGQEETQQQQQQMKKQRGERFGIPTTTQASNISFGGGSAEDPKARYNGPNKPVVIQPPPVSAPPMSFQHEQSTNAAQGYYRNNTADHIRDLFSSLPGEDDEEDEENLEGEDGEDFGVHTESDTEVPS

>Bra013886

MEEREGTNNTTTSLNQAAAASSDAYPMDPPRPDNPNPFSAPPTTSSVGNAAPQFSLTMQAETASSEQKKKRGRPRKYNPDGTLAVTLSPMPISSSVPLTSGLPPRKRGRGRGRSSQWLKKPEMFQFDRSPVETSFAGVGTAADFVGANFTPHMLTVNAGEDVTMKIMTFSQQGSRAICILSANGLISNVTLRQTTTSGGTLTYEGRFEILSLTGLFMQNDTGGTRSRAGGMSVCLAGPDGRVFGGGLPGLFLAAGPVQVMVATFIAAQEQSHLQLAQERRQRFGAQPPSISFNITAEERKARFERLNNSVTVPAPTPPSYPHDNTGNAVHSYYTNSVNHVKDPFSSIHGGGEDEGEEDEGDGNELEGEDEEFGESDNEIPS

>Bra013646

METTREVVKTTGGIDGGLTVVRSNAPSEFHIAPKSETSNPRPTSVAPSLQPPQNSFASTGPPPATEGFSTRQMKKKRGRPRKYGHDGAAVTLSPNPISSAAPTTSHVIDFSAAEKRGRVKPATERSFVRTKYQVENLGEWAPSSAGANFTPHIITVNAGEDVTKRIISFSQQGSLAICVLCANGVVSSVTLRQPDSCGGTLTYEGRYEILSLSGSFMPIDSDGTRSRTGGMTVSLACPDGRVVGGGVAGSLVAATPIQLVVGSFIAGTNQQHQNPKNQNHSFTPSPIPTIRPTSSSLQMDTTSRF

>Bra035899

MEEKGGMSPSGVITVKGDETLAPRTEFQQNPSFLPSVRPTTVVPPLPPAPATVTPGSAAAAPPPSISSAGTDLTKKKRGRPRKYAPDGSLNPRASRPTLSPTPISSSIPFSGDYHHHSHWKRGKAHQQPVEFIKRSNKFEYESSPAPPPAGLSCYMGANFTTHQFTVNAGEDVTMKVMPYSQGSRAICILSATGTISNVTLRQPTTSGGTLTYEGRFEILSLSGSFMPTENCGTKGRSGGMSISLAGPNGKIIGGGLAGMLIAAGPVQVIMGSFIVMHQAEQTHKKKPRIMEACPPPPQPQQQPPTFTITTVNSTSPAVATVEEPKQQTYGGGGTMRPLSQMPSSFHNDSITTTYHGYGNMNTGATNYKEEDDYENGGDDDSGDTRSLSNSG

>Bra019174

MEEREGTNNVTSFGLNQQQQHEAAASSYPMDPPRPDNPNPFSAPPITSSSSAAAAVENAAPQFSLTMPAAETGSSEQKKKRGRPRKYNPDGTLAVTLSPMPISSSVPLSSEFPPRKRGRGRGRSSRWLKKSQMFQFDRSPVVTNLTGAGVAAAADFAGANFTPYVLTVNAGEDVTMKIMTFSQQGSRAICILSANGPISNVTLRQSTTSGGTLTYEGRFEILSLTGSFMQNDSGGTRSRAGGMSVCLAGPDGRVFGGGLAGLFLAAGPVQVMVGTFIAGQEQSQLQLAKERRQRFGSQPSSISFNFTAEERKARFERLNNSVAIAAPTPSYQHENTANAVHSYYTNSVNHVKEPFSSMPGDGGHEEGEGEEDDNELEGDEDGEFGGDSQSDNEIPS

>Bra027801

MDGREAMAFPGSHYYLQRGGGGAFTNLSPSQVASGLHAPPGMRPMPNPNVHHPLANNPGPQFPMADHRLSDFGHSIHMGMSSSVAVEQQPPPMMETPMVKKKRGRPRKYAPEGQVSLGLSPMPSSSSASNKAKDSSAVTDPNAPKRARGRPPGTGRKQRLANLGEWMNTSAGLAFAPHVISVEAGEDVVSKVMAFSQQRPRALCVMSGTGTVSSVTLRQPATTEASLTFEGRFEILSLGGSYLVNEGGGPKSRTGGLSVSLSGPEGHVLGGGIGMLIAASLVQVVACSFVYGGIAKPNNTNKTIKQENKPKEEHDNSEMEPTPANAPEEAHETPQDFSAQGMSGWPGSGEGSVSGSGRSLDSSRNLLTDIDLTRG

>Bra022555

MDEREGTSSSSNSNNNITSFGLNQHVAPPPPNSGVYQMGPPRSENPNPFPAGLPNTNAASASASAVAATAPENAASPFSLTMPVGNSSSELKMKKRGRPRKYNPDGPLAVTLSPMPISSSVPLTTGFAPPQRGGRGQGRGRERGQARVDPPSNNNRLTNPQMFGFNNSSPVVGTSEVVCASFTPHVLTVNVGEDVTMKIMTFSQQGSRAICILSGNGAVSSVTLRQSFTSAGTLTYEGHFEILSLTGSFITSETGGIRSRAGGMSVSLSGPDGHVFGGGLSGLLLAAGPVQVTVGTFEAGKEEPQHQQMQNLRRERLGIPMTTQDSNISFGGGSAEDPKARYGLNKPVVIQAPPVSAPPVSLPHEPNTNAAQGYYTNNTANQIRDLFTSLPGEDDEDFEGEDDDEEFGSDSESDTEVPN

>Bra033121

MRWSLPAKLDQRPPPAGNGGGITVVGSDSPSDFHIAARSESSNPSPKCVAPPLPQSHHTNQLAIVAPPPQISMAATTMMEGISGVPMKKKRGRPRKYGPEGAVVALSPKPISSAPAPSHPLPASSHVIDFSASEKRSKMKPTNSFNRTKFHHQVENLGEWVPCSVGGNFTPHVIAVNAGEDVTMKIISFSQQGPRAICVLSANGVISSVTLRQHDSSGGTLTYEGRFEILSLSGSFMPNDIGGTRSRTGGMSISLASPDGRVVGGGVAGLLVAASPVQVNHFLIFPVQRATAVFNRRLKFHVFVESNRWL

>Bra029271

MEEKGGVSPSGVITVKGDEEATEFQQNPSLLQPVGPTTVVSRTPSPPPSPPPPPPPTSVTPVSAAAAPPPLISSAGLDLTKKKRGRPRKYAPDGSLNPRYLRPTLSPTPISTSIPFSGDYSHWKQGKAQQYLPVELIKKPHMFEYGSSPAFPPPPGLSCYVDANFTTHQFTVNAGEDVMMKAMPYSQGSRTICILSATGSISNVTLRQAAISGGTLTYEGWFEILSLSGSFMPTEQGGTKGWSGGMSISLAGPNGKIIGGGLAGMLIAASPVQVIMGSFIVINQAEQTQNKKPRIMEASPPPQQQQHPTFNITNVNSTSPVVATIDDPKQQTDGGGGMMRPVAQTPFHNDNSAMNNFTTTNHGYGNVNTSTNKEEADYADGGDDDQPDDDSCDTRSLSNGD

>Bra007595

MDRREAMSGSYYIQRGMPGSAPPPPQTQQPFHGSQGFHHFSNPNSPFVSNPNLNQVGGGGSTAFVSPPLPIESSPADSSAAVAAAAPPPSSGEKRKRGRPRKYGQEGPVSLALSPSMSPNSNKRGRGRPPGSGKKQRLSSSGELMPSSSGMSFTPHVIAVSVGEDIASKVLSFSQQGPRATCVLSVSGAVSTATILQPSPSQGAIKYEGRFELLSLSISYLNATDNDYSNRTGNLSVSLASPDGRVTGGGIAGPLIAASPVQVIVGSFLWAVPKGKVKKRDEASEDVQDADGLENNDNNTAPTSPPQQSQNLDQTPVGMWSTGSRSMDMHHAHMDIDLMRG

>Bra010070

MEDKSGISPSGVITVKGDEALASRTEFQQSPSFLQFVSPTTVVTPPPPPPPAPTPASTTVNPGSAAAPPPPPISSAGLDLTKKKRGRPRKYAPDGSLNPRASRPTLSPTPISSSIPFSGDYNHHSHWKRGKAQQQHVDIIKKSHNFEYGSSPAPPPPPPGLSCYVGANFTTHQFTVNAGEDVTMKVMPYSQGSRAICILSATGCISNVTLRQATTSGGTLTYEGRFEILSLSGSFMPTDNGGTKGRSGGMSISLAGPNGKIIGGGLAGMLIAAGPVQVVMGSFIVMHQAEQTQKKKPRIIEASPPPLQRPPPGFTITTVNSTSPLVATVEEPKQQTYGGGGGIMRPISQMPSSFNNDNSGMNNFTTTFQGYGNMNTGTNKDEDDYDDGGDDDSGDTRSLSTSG

>Bra037321

METSDKISFGVTVIGAEGPSTFHMAPRPSDEIPNQFLGSSLAPMEAPMASASSGDALGKKKRGRPRKYEANGALLPMQICSSASLVKKKRGRGKFNGLDMKMHKRMGFHTSGERFGVGFGSNFTPHIITVNAGEDINMRVISFSQQGPKAICILSANGVISNVTLRHPDSCGGTLTYEGRFEILSLSGTFMETENIGSRGRSGGMSVSLAGPDGRVVGGGVAGLLIAATPIQVVVGSFVTGDQQEHKKPLRKQRVDHTTSSTVMALPPPPPPSVFSSSNPEREQPLPSSLDISSWNNGEDNLRSLDTDINVSSPAH

>Bra027642

MDGREAMSFPSHYYLPRGAFTNLSPSQVASGLHAPPPGMRPMSNPNVHHPQANNPGPHFSMADHRHSDFGHGIHMGMASSAAVQPPPLPQQPPPMEQPLVKKKRGRPRKYAPDGQVSLRLSPVACSSAGSNKAKDSSAVTDPNAPKRARGRPPGTGRKQRLANLGEWMNTSAGFAFAPHVISVGAGEDIVSKVMSFSQQRPRALCIMSGTGTVSSVTLRQPATTEPSLTYEGRFEILSLGGSYLVNEEGGSKSRTGGLSVSLSGPEGHVIGGGIGMLIAASLVQVVACSFVYGGVPKSNNNNNKTIKQEGEPKEEHNDSEMETTPTNPEAAAEQVAQQTPQNFSGQGMSGWPGSGEGSVSGSGRSLDSSRNLLTDIDLTRG

>Bra025716

MDSKETQQQQNRNAAAALAGPTSTSQAMHNRSSVGALSLRQPQALQGVMPDGSPYSASVATQQPWRQVKRGRGRPRKYAPPDGGDSGGGANAGPPARGGEEGSFTAHVININAGEDIAAKLLAFVNQKPRHVCVLSALGDVSVAELSNNPLGLGLVKYEGPYVITAMSGVFSSTESNGTVTTTGNLNVSLAGPDFKTVGGRVGGMLVAGSPFQVIVGSFVPEGVKLSAASGPDNVLNSSGGGGPGLPQSQGPSKSSEENASKSPGNNAQ

>Bra020612

MEPNERHHHHHHHQEHHLTSPYYHPFHHHTPTTVSAAPSDNGNFPPPPNDGSSSSYHHSAPSSAPIEPVKRKRGRPRKYDTPAQALAAKKLASSASSSSAREKREQTAAAAGVSPPSKPGSKKSLSGSSGKSGQSFTPHIVNITPGEDVAQKIIHFAEQSKHELCILSASGTISDASLSHLATGTSASYQGQYEILSLSGSYIRGEHGGKTGGLSVCLSSSDGQIVGGGVGGLLKAAGPVQVILGTFQLERKKDGRNGVKGDDASGSGDLLPSSPSGAESLHGYRPVMEPSGRNSNDEHCTMTSGGAHFMMQPPRGMHMTHARSSEWDGAGYDLSGMRGNGSLENGEYKWAD

>Bra009999

MEPNERHHHQQQQQQQQQQHHHHHLTSPYYHPFHHHNPTTVSAAPPTTSTNNGNLTSPPPSNDGSSSSLPVYPHSVPSSAVTAPIDPVKRKRGRPRKYDTPAQALAAKKLASSASNSSARERREQAAAAGVSPPPSKSGSRKGLSGYVGKTGQSFTPHIVSITPGEDVAQKIILFAEQSKHEICILSASGAISSASLSHIATGTSVSYQGHYEILSLSGSYIRSEHGGKTGGLSICLSGSDGQIIGGRVGGLLKAAGPVQVIVGTFQLEKKKDGGNDVKGDDASGSGSLLPSPSGTESLHVYHPSMESSGRNLNNEHHTMTSGGALGGGAHFMMQPPQGMHMSHARPSEWGGAGYDLSGMRGNGSSENGDYE

>Bra009999

MEPNERHHHQQQQQQQQQQHHHHHLTSPYYHPFHHHNPTTVSAAPPTTSTNNGNLTSPPPSNDGSSSSLPVYPHSVPSSAVTAPIDPVKRKRGRPRKYDTPAQALAAKKLASSASNSSARERREQAAAAGVSPPPSKSGSRKGLSGYVGKTGQSFTPHIVSITPGEDVAQKIILFAEQSKHEICILSASGAISSASLSHIATGTSVSYQGHYEILSLSGSYIRSEHGGKTGGLSICLSGSDGQIIGGRVGGLLKAAGPVQVIVGTFQLEKKKDGGNDVKGDDASGSGSLLPSPSGTESLHVYHPSMESSGRNLNNEHHTMTSGGALGGGAHFMMQPPQGMHMSHARPSEWGGAGYDLSGMRGNGSSENGDYE

>Cpa74.23

MDGREAMALAAGSAQYYIHRAGAVGSASAPQTGALHVPAGFRPLSNQTVQGQPNAGSTFGIEPRHANFAHAINMGISSGVPPGEPVKKKRGRPRKYAPDGQVSLGLSPLPARPKPSSGSDSQTPKRARGRPPGTGRKQQLATLGEWMNRSAGLAFAPHVISIAVGEDIVAKILSFSQQRPRAVCILSGTGTVSLVTLRQPASNVPTVTYEGRFEILCLSGSYLLDEAGGPRSRTGGVSASLSSPDGHVIGGGVGTLIAASLVQVVVCSFVYGSSKIKNKQVASSKDDREAAVSQLVNKSTTPASAPATQSFNAVTMGGWAWVLGRLI

>Cpa18.240

MDRRDAMSLPGSASYYMQRGITGSGSGSQSALHGSPGIHPLSSQNVQFQSNMGGTSMGSTLPVEPPSTMLPHGVNMGAPPGMPLGEPVKRKRGRPRKYGPDGTVSLALSPSTSTHPGTITPSQKRGRGRPLGTGKKQQLASLGEWLSGSAGMGFTPHIITIATGEDIATKIMAFSQQGPRAICVLSANGAVSTVTLRQPSSSGGTVTYEGRFEILCLSGSYLVTSDSRNRTGGLSVSLASPDGRVIGGGVGGVLIAASPVQVIVGSFLWGGSKMKNKKSEGSEGMRDVDHQTVDNNPVTPASVQSSQNLIPTSVGGMWTGSRPMDMCNTHVDIDLMRG

>Cpa349.2

MEAREGMSSGVTVIGAEAPSAYHMAPRPENPSQFAGSPPVDASPVSVGLTGTLGKKKRGRPRKYGPDGTVATALSPMPISSSVPPAGDFSAGKRGRGKLSGFQKQLKKLGLDISGEGAASSVGTNFTPHVITVNAGEDVTMKIISFSQQGPRAICILSANGLISNVTLRQPDSSGGTLTYEGRFEILSLSGSFMPTESQGTRSRSGGMSVSLASPDGRVVGGGVAGLLIAASPVQVVIGSFLPSDHQEQKPKRQKIESIPATVVSVTTAPASLSANMEVEEGIGGLTQQNSSASKQNIAASPSFHRESWATMQEPRNSTTDINISLPAG

>Cpa29.71

MEATREAVSGTSGGVTVVGSDAPSEYHIAPRSDNPNPSTGSAQPPPPQPASQGGAPPPPPPPPAAPVSAGLPMKKKRGRPRKYGPDGSVTMALSPKPISSAAPPPVIDFSVEKRGKVKPASSFSKTKYELENLGEWVACSVGANFTPHIITVNSGEDVTMKIISFSQQGPRAICILSANGVISSVTLRQPDSSGGTLTYEGRFEILSLSGSFMPNDNGGTRSRSGGMSVSLASPDGRVVGGGVAGLLVAASPVQVVVGSFLAGNQHEQKQKKQKHEIIKTTITTTAIPVSTADPRATPTLSSSSLRGENWSSLPSDSRNKSTDINVSLPGG

>Cpa5.94

MNEKESTVSGSPGNSETESPPPVTSNMAAVQPQVVNMNMNTNMNMNTNTNTNTNTNTNTNTNTNTNTNTNTNTNTNMAVGVDRTTLATTMGTTATATVTGTMMVTTAGSGGLGSSGSGKKKRGRPRKYDADGNLRVPYHVGLSSSSPGFSLSPSSPSNDYSNSKKCKGRPPGSGNWQLLASLGELFASTAGGDFTPHVVTVHTGEDVAGKILSFSQKGPRGICILSANGAVSNGRFEILSLSGSFTVSDSGGVRSRTGGLSVSLAGPDGRVIGGGIAGLLTAASPIQIVVGSFMPNGYKMYKKKLQREQTSSTSFPLGTDSLTAARPISQAKPEGETCLAPGSPVKGQSHGEADSSRNDVKTANSLHAAGWILGHDSNHQRTSPDINVSVPSE

>Mdp388292

MEEKESTFSGSPGNSETESPPVTQKVSMPMAAMAAVDATVSSEENYNNTPVSETTQATEVAAAASVGRGDLSGKKKRGRPRKYDADGNLRPGYNSNNKSNKGGAVPPPPPGFYLSYPLSSEFSSSSKRGRGKPSGSGNWQILASLGGLFANTAGGDFTPHVVNVSTGEDVASKIFSISHKGRGVCVLSANGAVSNVTIRQPGSSGGILTYEGRFELLSLSGSFTVTEIGGVRSRTGGLSVSLAGPDGRVIGGGIAGVLTAASPIQIVVGSFMPNGYKAHKVRKHYRENTVGSPISSAPDTVTAATPISQAQPETESRLNAISPLPANSHGGEANKSMIQMHGTSVISTNAGWNGADLKSEHRPSPDINVSVPGEEKKHFASSNCLIPQSKKMKTYEENLGNFSSLLNRWQQVLMQRXNCNTIALQXLSSAVLRKKPKGPEDGEPSNQANRNTTTLQPLFPAVLRKKPKGSAIRTPRTVSDVVRHVGPDHQQIRQRLV

>Mdp511968

MEPNENQLSSYFQHPTTTTAPAATSTAATTPSPTNGLLPNTHSTYGSHMLYSHSVPSSAVTSPLEPAKRKRGRPRKYGTPEQALAAKKAATTSSHSSSSKEKKDQHGAVSPSFSGSTKKSQQFSLGNAGQGFTPHVLTVAAGEDVGQKIMFFMQQSKREICILSASGTISNASLRQPATSGGNITYEGRFEIISLSGSYVRTDLGGRAGGLSVCLSNTDGQIIGGGVGGPLKAAGPVQVIVGTFQIDAKKDATAGVKGDASAAKLPSPGEMMSGGFHPTLDPSGRPLVRGNEDQQPMGGSHFMLQGMHVAPSRPTDWRGGPDARGTGAYELTGRGVRAPHQSPENGDYDQIQD

>Mdp537302

MDGREAMGLSGGSASYYIHRGGGVGGSMPGSQATGGGGGLHHAPPGFRPISSTLMQPQSNVRVSSVGSTFSVEPSRPNFQHRGSTMNVAPGVPSAQPVKKKRGRPRKYGPDGPVSLGLSPMSATPTPTPGSTSPTPKRSXGRPPGSGRKQQLATLGDWMNTSAGLAFAPHVITIGAGEDIAAKLLLFAQQRPRALCILSGNGAVSSVTLREPASTGVSVTYEGRFQILCLSGSYLVAEDGGPRNRTGGISVSLSNPNGHVIGGAVAVLIAATPVQVVLCSFVYGGSKTKNKQLADPNSDENSEAQHSEKLGLPSSAPPTQNYNPSGAGIWPGSRQVDLRNAHTGIDLTRG

>Mdp123732

MEEKDNKLVSGVAVSGEQAPENYRIAPRTENPSPSGGPTTVVAPMTSPMSLTGTGTDGKKKRGRPRKYGPDSKPVRSSALSPMPISSSILLPGEFSAWKRGRGKPVDSIKKSHKHDVFENSGKQIERVIEVPACSIDNSFDLNHSPQYDHSTLETSSYVKEIPSPEDYEQFFEWPLGDKIAYSVGANFTPHVLNVHSGEDVTMKIMSFSQQGARAICILSANGTISNVTLRQASSSGGTLTYEGRFEILSLSGSYIPTENAGTKSRSGGMSVALAGPDGRVVGGGLAGMLIAAGPVQVVVGSFLQGHQQEQKPKKQRVEPVSSSIVPIIVNAMSGEEMKVYGGVKPILTAPSFHGDQSNSPNPVHGFKNSASESKSLLSEDDSKGSGQANCEVSY

>Mdp927762

MEEKDNKLVSGVAVSGENAPDDYRIAPRNENPSPCGGPTTAAAPTASPMSLTGIGTDGKKKRGRPRKYGPDSKPVPSSALSPMPISSSIPLTGEFSAWKRGRGKPVDSIKKSHKHDVFGNSGEKIAYSVGANFTPHILTVHSGEDVTMKIMSFSQQGARAICILSANGTISNVTLRQASSSGGTLTYEGRFEILSLSGSYIPTENAVTKSRSGGMSVALAGPDGRVVGGGLAGMLIAAGPVQVVVGSFLQGHQLEQKPKKQRLEPVSSSIVPIIVNAVSGEEMKVYGGVKPILMAPSFHGDHSNSLNPVHSYKNSASESKSLLLQEESKGSGQANCEVSY

>Mdp325245

MEGREGVNSSGVTVVGSDAPSDYHVAPRSENPTHNAGSTPQAPVAPPQAAAGSMPMKKKRGRPRKYGPDGSVTMALSPKPISSSAPPPPPVIDFSAEKRGKVKPSSVSKTKYEVENLGEWVACSVGANFTPHIITVNSGEDVMMKIISFSQQGPRAICVLSANGVISSVTLRQPDSSGGTLTYEGRFEILSLSGSFMPNETGGTRSRSGGMSVSLASPDGRVVGGGVAGLLVAASPVQ

>Mdp322198

MEQMFEGPVGYQAFPNSIQAGTSPAMAPPPVSGGVNGAITSGKKKRGRPRKYGPYGTAPAAKKPSSPDGTVPPATGKCSSDQKGKAPIGYVYYNHLVFTHLHFIACSLGEDWGSIETTLYNFTPYMITVNSGEDVAWKIAQTAQQNTGRGIIIMSATGTLKEVTLINPAHGISPIKHEGQFNIIQISGSFMMYDKKGTCSGGMSITLADSDGRSVEGSAGGSLIAATPVQVCIGSFTIGQQTVTPQAVKGPTAPEMTKVHVLL

>Mdp174858

MEEMFEGPVGYQAFPNSIQAGTSPAMAPPPASGGANGASTSGKKKRGRPRKYGPYGTASAAKKPSSPDGTVPPATGKCSSEQKGKAPMAEPIGEDWGSIETTLNNFTPYMITVNSGEDVAWKIAQTAQQNTGRGIIIMSATGXLKEVTLINPAHGISPIKXEGQFNIIQISGSFMMYDNKGTCSGGMSITLVDSDGRSVEGSVGGSLIAATPVQVCIGSFTIGQQTITPQAVKGPTAPEKT

>Ppa08335m

MEEKDNLVSGVAVSGEEAPDTYRIAPRNENPSPSGGPTMAAAATASPMSLALTGTEVKKKRGRPRKYGPDKTVSSALSPMPISSSIPLTGEFSAWKRGRGRPVDSVKKSHKYDVFESSGEKIAYSVGANFTPHVLTVHAGEDVTMKIMSFSQQGSRAICILSANGTISNVTLRQPSSSGGTLTYEGRFEILSLSGSYIAIENAGTKSRSGGMSVALAGPDGRVVGGGLAGMLIAAGPVQVVVGSFLPGHQQEQKPKKQRLEPVSSSIVPIVVNAVSGEEMKVCGGVKPILTSPSFHGNNSTSVNPMHSFKNSAPESKSLSEEESKGPGQPNCEVSY

>Ppa07786m

MEPNENQLSSYFQHPTTTTGTGTAATVTATNTASPTNGLLPNTHSTDGSHMVYSHSVPSSAVTSPLEPAKRKRGRPRKYGTPEQALAAKKAATTSSHSSSSKEKKDHHGSASPSYSGSTKKSQQFSLGNAGQGFTPHVLTVAAGEDVGQKIMFFMQQSKREICILSASGTISNASLRQPATSGGNITYEGRFEIISLSGSYVRTDLGGRAGGLSVCLSSTDGQIIGGGVGGPLKAAGPVQVIVGTFMVDAKKDVTAGVKGDASATKLPTAGEMMNVSFRSAVDSSGRTLVRGNDDQQAIGGSHFMIQGMHVAPSRPTDWRGGPDARGTGAYELTGRAGRAAHQSPENGDYDQIPD

>Ppa08806m

MEGREGVNSSGVTVVASDAPSDYHVAPRSENTTHNAGSTPPAPVAPPPAAALPAAASLPMKKKRGRPRKYGPDGSVTMALSPKPISSSAPPPVIDFSAEKRGKVKPTSSVSKTKYEVENLGEWVACSVGANFTPHIITVNSGEDVMMKIISFSQQGPRAICVLSANGVISSVTLRQPDSSGGTLTYEGRFEILSLSGSFMPNETGGTRSRSGGMSVSLASPDGRVVGGGVAGLLVAASPVQVVVGSFLSGNQHEQKPKKQKHDYISNATPTMAVPISSVDPKPNFSSSTSFRGDNWSSLPSDPKTKTDINVSLPGGVI

>Mes010698m

MEARETVSSSGGGGVTVVGSDVPSEYQIAPRSADNPSSAPGSAPPPPPPPAVVPPPSTAATMPLKKKRGRPRKYGPDGSVTMALSPKPISSAAPALPPVIDFSVEKQRKIKPVSKAKYVLENLGEWVACSVGANFTPHIITVNAGEDVTMKIISFSQQGPRAICILSANGVISSVTLRQPDSSGGTLTYEGRFEILSLSGSFMPTESGGTRSRSGGMSVSLASPDGRVVGGGVAGLLVAASPVQVVVGSFLAGNQHEQKPKKQKPDSISISTVVSPTIAVPISTADPKSNLSTAAVPISTADPKPNLSSPTFRGTADPKPNLSSPTFHGDSWSPLPSDSRNKPTDINVSLPAG

>Mes010640m

MEPNESQHHHHLSSYFTTTTPATTTPSPTNGLLPPPPNTTSDSGGGPHMVYPHSVGPSSASVTTAPVEPVRRKRGRPRKYGTPEQALAAKKTASSHSVSKEKREGASSSSPSYSGSSRKSQQLFALGNAGQGFTPHVITIAAGEDVAQKLMMFMQQSRREMCILSASGSISHASLRQPATSGGNITYEGRFEIISLSGSYVRTDIGGRTGGLSVCLSNTDGQIIGGGVGGPLTAGGPVQVIVGTFLLDTKKDVNTGVKVDASASKLPTPIGGASISNIGFHSPVESSGRNPIRGNDDHPTMGGNPFMTHPRGMHVAPSRSTDWRIGPDARVNSGYDLTGRVGHGAHQSPENGDYE

>Mes010812m

MEPNESQHHLSSYFATTTTTPSPTNGLMPPHPNSTSDSGGGPHMLYPHSVGPSSAAVATAPVEPPRRKRGRPRKYGTPEQALAAKKTASSSNSVPKEKREGATSYSGSSRKSQQLFALGNAGQGFIPHVITVAAGEDVAQKLMMFMQQSKREMCILSASGSISNASLRQPATSGGNITYEGRFEIISISGSYVRTDIGGRTGGLSVCLSNTDGQLIGGGVGGPLTAGGPVQVIVGTFLLDNKKDASGGVKVDASTNKLPSPVGGASISNIGFLSPVESSGRNPVTGNDDHANIGGNPFMIHPRGMHVAPSRTPDWLSGPDPRVNAGFELTGRVGHGAYQSPENGDYEQLPD

>Mes010939m

MDGREAMAFASGSAPYYIHRGGGVGATGSGSQTGALHTQPGFRPLSSPNLAVQSNFRPGSSGPAFSAEPSNANFGHGIDVPVSSGVSMSEPMKKKRGRPRKYAPDGQVSLGLSPMPLKPKPSSGQDPLSPRRRKGRPPGSGRKQQLALLGDWMNNSAGLAFSPHVISVGAGEDVVAKLLSFAQQRARAVCILSGTGTVSSVTLRQPASTEPTVTFEGHFEILCLSGSYLIAEDGSPRDRTGGISASLSSPDGHVIGGGIGILIAAGPIQVVACSFVQGGSKSKDRHVGRPKLNKDSASQPDDKSATPKSAIPMNLQQNFTPSPMHGWPVSRSVDLRNPHTDIDLTQG

>Mes010247m

MEAKEGVSLGVTVKGDEAPENFRVAPRTENSISNLNPSHNPIFSNSNPNPHSNPDPAPNANPTPNTSQFGGPTVISSPATAGTEVKKKRGRPRKYGPDGTLVTALSPMPISSSIPLSGELSAWKRGRGRPLESVKKQYKYENESTGDRIAYFVGANFTPHVLTVNVGEDVMMKVMSFSQQGARAICILSANGTISNVTLRQATSSGGTLTYEGRFEILSLSGSFMPTDNGVTKSRSGGMSVSLAGPDGRVLGGGLAGMLVAAGPVQVVVGSFIPGHQQEQKHKKQRTEISPAIAPISVLSPEELKGAYGGVKPVLIPSSSFHASDNSVPLNPIQAFGNSASDNKNSSPDDESKGPNLSNCEVSS

>Mes010659m

MEEKEGISPGLAVKGVDARDSFGVAPRTENSVPNLNPNPNPVPNSNPNLNPNPTPNPNSSQFGGPLVTSLPASLGTEVKKKRGRPRKYGPDGTLATALSPMPISSSIPLTGELSAWKRGRARPLESAKKQYKYEYESTGGIAYFVGANFMPHVITVNAGEDVTMKIMSFSQQGVRAICILSANGTISNVTLRQPTSSGGTLTYEGRFEILSLSGSFMLTNNGGTKSRSGGMSVSLAGPDGRVVGGGLAGMLVAAGPVQVVVGSFLLGHQQEQKHKKQRSEIAPARASVSVLSAEGMKGAYGGVKPVLIAPSFHGDNSASLNPMQAFRNSSSDDKTSSPEDESKDPEQSKCEVSC

>Mes011818m

METREGLTSGVTVIGAEAPSTYHVAPRTENLSQMTVSPAVAVSPVSVGLTGTTEKKKRGRPRKYGPDGTVARALSPMPISSSAPSGGDFSAGKPCKMWPGSFEKKKYKKMGLENSGDWASTSVGTNFTPHVITVNAGEDVTMKVISFSQQGPRAICILSANGVISNVTLRQPDSSGGTLTYEGRFEILSLSGSFMPTESQGTRSRSGGMSVSLASPDGRVVGGGVAGLLIAASPVQVVVGSFLPGNQQDLKPKKPKIDAVTAPITPTPTIAAAPVPVSNPEKEESVGGNGQQISSSLRRENWTTMQSVQDLRKSGTDINVSLAEA

>Mes011214m

MLMNFCMETREGLTSGVTVIGAEAPSTYHVAPRTENPSQMGLTGAVSPVSVGLAGAVSTVSVGLTGTTEKKKRGRPRKYGPDDAVARALSPIPISSSAPPGEAFSGGKPSKVWPGSFEKKKYKKVGMENSGDWASTSVGTNFTPHIITVNAGEDVTMKVISFSQQGPRAICILSANGVISNVTLRQPDSSGGTLTYEGRFEILSLSGSFMPTESQGTRSRSGGMSVSLVSPDGRVVGGGVAGLLVAASSVQVVVGSFLPGNQQDTKPKKFKIDSIPATLTTVPAIAASPVPASNPEREENMCGNGQQNSSFFGRENWTAMQAVQDMRTSGTDINISLPED

>Al324562

MDRREAMALSGSGSYYIQRGIPGSGPPPAPQTQPTFHGSQGFHHFTNSNSPFGSNPGGVSTGFVPPPLPVESSPADSSAAAGAVVVPPSGDTSLKRKRGRPRKYGQDGSVSLALSPSVSNVSPNSNKRGRGRPPGSGKKQRLSSIGEMMPSSSGMSFTPHVIVVSIGEDIASKVISFSHQGPRAICVLSASGAVSTATLLQPAPSHGTITYEGLFELISLSTSYLNTTDNDYPNRTGSLAVSLASSDGRVIGGGIGGPLIAASQVQVIVGSFIWAIPKGKIKKREETSEDVQDTAALDNNDNTAATSPPVPQQSQNLVQTPVGIWSTGSRSMDMHHPHMDIDLMRG

>Al482309

MSGSETGFMAATRESVQFTMALHQQQQQRSEAQPQQSQNMQSSFGGDDGADLYRQPMRSASPPQQYQPNSAGENPVLNMNMPGAEHGAVTGSEPVKKRRGRPRKYGPESGETSLGLFSGAPSFTVSQPVSGGGGGEKKMRGRPPGSSSKRLKLQALGSTGIGFTPHVLTVMTGEDVSSKIMALAHNGPRAVCVMSANGAISNVTLRQSGTSGGTVTYEGRFEILSLSGSFHLLENDGQRSRTGGLSVSLSSPDGNVLGGSVAGLLIAASPVQIVVGSFIPDGEKEPKQHVGQMGLSSPTLPRVAPTQVLMTPGSPQSRGTMSESSCGGGHGSPIHQGTGSYSWK

>Al483725

MDRRDAMGLSGSGSYYIHRGLSGSGPPTFHGSSQQQGLRHLPNQNSPFGPGSTGFGSPSPATTAGGAGALPHHIGVNMIAPPPPPSETPMKRKRGRPRKYGQDGPVSLALSSSPVSTITPNNSNKRGRGRPPGSGKKQRMASIGELMPSSSGMSFTPHVIAVSIGEDIASKVISFSQQGPRAICVLSASGAVSTATLLQPSAPGAIKYEGRFEILALSTSYLVATDGSFRNRTGNLSVSLASPDGRVIGGAIGGPLIAASPVQVIIGSFIWAAPKIKSKKREEEASEVVQDTDDHHVLDNNNNTISPVPQQQPSQNLIWSTGSRQMDMRHAHADIDLMRG

>Al494313

MDSRDIPPSQNQLQPPPGMLMSHYRNPNAAAAALMVPTSTSQSIQHHHRLPFSNQQQQQSQTFHQQQQMDQKTLESLGFGDGSPSSQPMRFGIEDQNQNQQLQVKKKRGRPRKYTPDGSIALGLAPTSPLLSAASNSYGGGDGGVGDSGGGGGNGNSADPPAKRNRGRPPGSSKKQLDALGGTAGVGFTPHVIEVKTGEDIASKVMAFSEQGPRTICILSASGAVGRVTLRQASHSSGIVTYEGRFEIITLSGSFLNYEVNGSTNRSGNLSVSLAGPDGRIVGGSVVGPLVAATQVQVIVGSFVAEAKKPKPSSVNNARGQNPEPASAPANMLNFGSVSQGPSSESSEENESGSPAMHRDNTNGIYGAQQQQPLHPHQMQLYHHLWPNHGQ

>Al946418

MLMSHHNSYNRNPNAAAAAVLMGHNTSTSQAMHQRLPFGSMSPHQPQQHQYHHPQPQQQIDQKTLESLGFDGSPSSVAATTQQQSMRFGIDHQQVKKKRGRPRKYAADGGGSNIALGLAPTSPLPTASNSYGGGNEGGGTGGDSGGANANSSDPPAKRNRGRPPGSGKKQLDALGGTGGVGFTPHVIEVKTGEDIATKVMAFTNQGPRAICILSATGAVTNVKLRQATNPSGIVKYEGRFEIISLSGSFLNSESNGTVTKTGNLSVSLAGQDGGIVGGSVAGMLVAGSQVQVIVGSFVPDGRKQKQSAGRAQNTPEPASAPANMLSFGGGGGGPGSPRSQGQQHSSESSEENESNSPLHRGTNNNNNNSNNNNNNHGLFGNATPQPLHQIPMQMYHLWPGSSPQ

>AtAHL5

MDGREAMAFPGSHSQFYLQRGVFTNLTPSQVASGLHAPPPPPGMRPMSNPNIHHPQASNPGPPFSMAEHRHSDFGHSIHMGMASPAAVQPTLQLPPPPSEQPMVKKKRGRPRKYVPDGQVSLGLSPMPCVSKKSKDSSSMSDPNAPKRARGRPPGTGRKQRLANLGEWMNTSAGLAFAPHVISVGSGEDIVSKVLSFSQKRPRALCIMSGTGTVSSVTLREPASTTPSLTFEGRFEILSLGGSYLVNEEGGSKSRTGGLSVSLSGPEGHVIGGGIGMLIAASLVQVVACSFVYGASAKSNNNNNKTIKQEIKPKQEPTNSEMETTPGSAPEAAASTGQHTPQNFPAQGMSGWPVSGSGSGRSLDSSRNPLTDIDLTRG

>AtAHL8

MDSRDIPPSHNQLQPPPGMLMSHYRNPNAAASPLMVPTSTSQPIQHPRLPFGNQQQSQTFHQQQQQQMDQKTLESLGFGDGSPSSQPMRFGIDDQNQQLQVKKKRGRPRKYTPDGSIALGLAPTSPLLSAASNSYGEGGVGDSGGNGNSVDPPVKRNRGRPPGSSKKQLDALGGTSGVGFTPHVIEVNTGEDIASKVMAFSDQGSRTICILSASGAVSRVMLRQASHSSGIVTYEGRFEIITLSGSVLNYEVNGSTNRSGNLSVALAGPDGGIVGGSVVGNLVAATQVQVIVGSFVAEAKKPKQSSVNIARGQNPEPASAPANMLNFGSVSQGPSSESSEENESGSPAMHRDNNNGIYGAQQQQQQQPLHPHQMQMYQHLWSNHGQ

>AtAHL9

MDRRDAMGLSGSGSYYIHRGLSGSGPPTFHGSPQQQQGLRHLPNQNSPFGSGSTGFGSPSLHGDPSLATAAGGAGALPHHIGVNMIAPPPPPSETPMKRKRGRPRKYGQDGSVSLALSSSSVSTITPNNSNKRGRGRPPGSGKKQRMASVGELMPSSSGMSFTPHVIAVSIGEDIASKVIAFSQQGPRAICVLSASGAVSTATLIQPSASPGAIKYEGRFEILALSTSYIVATDGSFRNRTGNLSVSLASPDGRVIGGAIGGPLIAASPVQVIVGSFIWAAPKIKSKKREEEASEVVQETDDHHVLDNNNNTISPVPQQQPNQNLIWSTGSRQMDMRHAHADIDLMRG

>AtAHL10

MSGSETGLMAATRESMQFTMALHQQQQHSQAQPQQSQNRPLSFGGDDGTALYKQPMRSVSPPQQYQPNSAGENSVLNMNLPGGESGGMTGTGSEPVKKRRGRPRKYGPDSGEMSLGLNPGAPSFTVSQPSSGGDGGEKKRGRPPGSSSKRLKLQALGSTGIGFTPHVLTVLAGEDVSSKIMALTHNGPRAVCVLSANGAISNVTLRQSATSGGTVTYEGRFEILSLSGSFHLLENNGQRSRTGGLSVSLSSPDGNVLGGSVAGLLIAASPVQIVVGSFLPDGEKEPKQHVGQMGLSSPVLPRVAPTQVLMTPSSPQSRGTMSESSCGGGHGSPIHQSTGGPYNNTINMPWK

>AtAHL11

MDRRDAMALSGSGSYYIQRGIPGSGPPPPQTQPTFHGSQGFHHFTNSISPFGSNPNPNPNPGGVSTGFVSPPLPVDSSPADSSAAAAGALVAPPSGDTSVKRKRGRPRKYGQDGGSVSLALSPSISNVSPNSNKRGRGRPPGSGKKQRLSSIGEMMPSSTGMSFTPHVIVVSIGEDIASKVISFSHQGPRAICVLSASGAVSTATLLQPAPSHGTIIYEGLFELISLSTSYLNTTDNDYPNRTGSLAVSLASPDGRVIGGGIGGPLIAASQVQVIVGSFIWAIPKGKIKKREETSEDVQDTDALENNNDNTAATSPPVPQQSQNIVQTPVGIWSTGSRSMDMHHPHMDIDLMRG

>AtAHL12

MDGREAMAFPGSHSQYYLQRGAFTNLAPSQVASGLHAPPPHTGLRPMSNPNIHHPQANNPGPPFSDFGHTIHMGVVSSASDADVQPPPPPPPPEEPMVKRKRGRPRKYGEPMVSNKSRDSSPMSDPNEPKRARGRPPGTGRKQRLANLGEWMNTSAGLAFAPHVISIGAGEDIAAKVLSFSQQRPRALCIMSGTGTISSVTLCKPGSTDRHLTYEGPFEIISFGGSYLVNEEGGSRSRTGGLSVSLSRPDGSIIAGGVDMLIAANLVQVVACSFVYGARAKTHNNNNKTIRQEKEPNEEDNNSEMETTPGSAAEPAASAGQQTPQNFSSQGIRGWPGSGSGSGRSLDICRNPLTDFDLTRG

>AtAHL13

MDSREIHHQQQQQQQQQQQQQQQQQHLQQQQQPPPGMLMSHHNSYNRNPNAAAAVLMGHNTSTSQAMHQRLPFGGSMSPHQPQQHQYHHPQPQQQIDQKTLESLGFDGSPSSVAATQQHSMRFGIDHQQVKKKRGRPRKYAADGGGGGGGGSNIALGLAPTSPLPSASNSYGGGNEGGGGGDSAGANANSSDPPAKRNRGRPPGSGKKQLDALGGTGGVGFTPHVIEVKTGEDIATKILAFTNQGPRAICILSATGAVTNVMLRQANNSNPTGTVKYEGRFEIISLSGSFLNSESNGTVTKTGNLSVSLAGHEGRIVGGCVDGMLVAGSQVQVIVGSFVPDGRKQKQSAGRAQNTPEPASAPANMLSFGGVGGPGSPRSQGQQHSSESSEENESNSPLHRRSNNNNSNNHGIFGNSTPQPLHQIPMQMYQNLWPGNSPQ

>Bd3g34030.1

MEAKDVAPLTTAATTAAVPAAPVPAPASQSQPLPPATSNAHAHQPPPHPFAHHQPPPPLQQQQPAPGSANPSAPMPGSGGMRLSFDQVVGKAAPGQHHHAPGPMLYAAPPPHAAGAPVRPQGGVGMGMGDMMRKKRGRPRKYAPDGSMALALAPLSSASGGSPMQPGQQQQQQHGGFSISSPPSDPNAKRRGRPPGSGKKKQFEALGSWGISFTPHILSVKAGEDVASKIMSFSQQGPRTVCILSANGAISNVTLRQPATSGGLVTYEGRFEIISLSGSFLLAEDGDTRSRTGGLSVALAGSDGRVLGGCVAGQLTAATPVQVVVASFIAEGKKSKLAEARKVEPMSAPPQMANYVPAPVASPPSEGTSSASSDDSGSPINHGGMPYNHSGQHQHPQQQQQHMPPAYASGGWSHSAHHQNNNRHDGDVKMMSN

>Bd3g40180.1

MEVRSEQGLMAGRDLFGIPKSAPAPVPSSAAMQQSVRMAYTADGTPVFAPVSSAVAPPGYQPVAAAPGSNMSTAAGAAGGNGVAALRDMGGPLAKKKRGRPRKYGPDAAVSLALVTVPPGAAGPTVVPQGASGPFSPTAPGSVVPSASPEGGKKRGRPKGSTNKPRVNVPGPVGVGFTPHVITVQAGEDVSAKIMSFSQHGTRAVCVLSANGAISNVTLRQTATSGGTVTYEGRFEILSLSGSFLVTDNGGQRSLTGGLSVSLAGPDGRLLGGGVAGLLIAASPIQIVVGSFNSDGRKEQKPQVMPKLQVSSEPTPLKVVPATGMGPNSPPSRGTLSESSGGTASPRHQGYTATNNNQPPILSSMPWK

>Bd3g55950.1

MDGRESAAASGANFSPFYVRPWGMGAARAAAGNPDGLHGPPPVGYRQHLDAVSAGYSFQQPHFGGSHIGQEYHHDHVEGSPHVVQHTAGMDIVAVGVDAKGGDQGSVEGQDEQVKKKRGRPRKYKPDRAVTLGLSPSPSTPHSSSSGMGAMVTTPGAGFGSGTGSGGSGSGALTEKRGRGRPPGSGKMQQLASLGTWFLGSVGTGFTPHVIIISAGEDVAARIMSFSQQGPRAICIISATGAVSTATLYQDSDSGAVTYEGRFEILCLSGSYLVLDEGGTRKRSGGLCIALCGPDHRVIGGSVSGVLTAAGTVQVIVGSFMYGGGSKKSKAKAEQDMENEEKNGGAEDTPTMALTEHNMPPHPMSGWPGLMNQMDSSSPMYGGSKKNKGKAEQDMENEEHNVGGEKTPAVALPEQHNMNMPPHPMSGWPPGLMRQTDSRSSNIDINSIRE

>Bd5g26720.1

MDGKDHLIAPSDLPQFYAAQQQQQQHHHRMLSNAGQQVSQASPLSGGMHHSVIRPMPNMSMSPTAILQSIGGAQMPPFHTMDAAPSPSSMMQHGGGAMGGSGVSGPGTATAMASPPEPVKRKRGRPRKYGPDGAMNKMSSSSLSSSHHQQQMMGAPPPRLGSLDMVGGMDVDAANKKRRGRPPGTGKKLSSPTKKPSGNAFSGSAGTSFTPHIITASPSEDVAGKIAAFATQSPRAVCVLSAMGSVSRVVLRHPADHASSVSRAPPSYNNPAIYEGLYEILSLSGSYNLNEDQQNQSDGISVTLCSPERHVIGGVLGGALVAASTVQVVLGSFVHGGSRAKSKKSGKQPNFGFDSLSGGGTDASPSSGHNQNLTPPSVVTTTGGWPSSGIFDTRSSNIDINSSRG

>Bd1g29110.1

MDSSDPCYYIHHRPSPAATAQPPPTAAFDDFRHHVAASPAFSFQMPQDQEPMPQHHAASASASASAGASAEDPSEQVKKKRGRPRKYKPPPDGLSPPSSTSALVTVPATPGSGPGPGGSGGPSEKRRGRPPGSGKMQQLASLGKCFLGSVGTGFTPHVIIIPSGEDVAARIMSFSQQGPRAVCIMSATGAVSTATLHQDASSGSVITYEGRFEILCLSGSYLVIDDGGSRTRNGGLCIALCGADHRVIGGSVGGVLTAAGTVQVIVGSFMYSGSKKNKKGKAEQEAETEEANGGGEEEAPSLMTMPHEDLSSDAMMGGWPDMMRQMDSRSCSIDMNSVRE

>Bd1g29120.1

MDSRDSCYYIHHRPSPSLRPPATAQQPPTAAFDDYRHHVDASPAFSFQMLQDQEPMSQHHAASASASAGATAEDPSEQVKKKRGRPRKYNPPPDGLSPPSSSALVKVPATPGPGGSGGPSEKRRGRPPGSGKMQQLASLGKWFLGSVGTGFTPHVIIIPSGEDIAARIMSFSQQGPRAVCIMSATGAVSTPTLHQDASSGSAITYEGRFEILCLSGSYLVIDDGGSRTRNGGLCIALCGADHRVIGGSVGGVLTAAGTVQVIVGSFMYAGSKNKKGKAEQEAETEEANGSGEEETPSLMTMPHEDLSSDAMMSGWPDMMRQMDSRSCSIDMNSVRE

>Cs303060.1

MDGREGMALSGGSASYYIHRGGGVGGSGSGLPTAGSHASPVFRPMANQGVLSHSNLRGNSVGSTYTVEPSHSNYLRGMGINVSAGVNSSEPVKKKRGRPRKYAPDGQVSLGLSPMSAGSKLTPGSNSSTPRRRRGRPPGSGRKQQLALLGDWMNNSAGLAFAPHVIHVGAGEDIVAKVLSFAQQRPRAVCVLSGNGTVSSVTLRQPASTGVSVTYEGHFQILCLSGSYLVAEDGGPRSRTGGISVSLASPDGHVIGGGVAVLTAAGPVQVVVCSFVYGPKIKNKQVAGPKSNDGSGHEHHDNLVSPTSAPSTQTYNPSSMGVWPGSRSVDVRNPRTGIDLTRG

>Cs313210.1

MAVGGQPAYSPANNNASSTIALNQPSAQMIPPSSRFPFNHPVIPPSSVPLDSLNVSPYDGSHSANFNVDSGKKRRGRPRKYAPDANNIALGLAPTPTVASSLPHGDLTATPDSEQPARKTRGRPPGSGKKQSNSIGSGGTGFTPHVLLAKPGEDVAAKILSFSQQGPRTVFILSANGTLSNATLRHSASSGGSVSYEGHYDIISLSGSFLLSENNGTRSRTGGLSVLLAGSNGQVLGGGVAGMLMASSQVQVIVGSFLEDDKKSNTSMLNSGSSSAPSQMINFGGGGGGGLAAAAASPPSLGGSSGESSGENGDSPLNNRHPGMFNNSSQPIHNLQMYQLWAGQTQQ

>Cs321780.1

MDSRERSMPSVHQHHQQSTPPNRMIPNNASYSANMPNSNNTSPLINPNSAAAQMMSSASRFPFNSMMGSSSKPSESPNAASYDGSQSELRTGGFNIDSGKKKRGRPRKYSPDGNIALGLSPTPITSSAVPADSAGMHSPDPRPKKNRGRPPGTGKRQMDALGTGGVGFTPHVILVKPGEDIASKVMAFSQQGPRTVCILSAHGAVCNVTLQPALSSGSVSYEGRYEIISLSGSFLISENNGNRSRSGGLSVSLASADGQVLGGITNMLTAASTVQVIVGSFLVDGKKLGASIQKSGPSSTSPNMLNFGTPVAAGCPSEGASNNSSDDNGGSPLSRGPGMYTNANQPIHNMQMYQQLWASRNQ

>Cs135450.1

MDRRDPMALSGSQSFYMQRGISNSGSGAQGLRSSTNPNVAFQTNTGGNNVGSGLPMDPNSGISPYGGNVGAQSGGVVASEPVKRKRGRPRKYGTEGTVSLALSPSPSAVNPATVASSPKRGRGRPPGSGKKQQLASLCETLSGSAGMGFTPHVITIGIGEDVAAKIMSFSQQGPRVVCILSANGAVSTVTLRQPSTSGGTVTYEGRFEIICLSGSYALGEIAGSRNRTGGLSVSLASPDGRVIGGGVGGALVAATPVQVIVGSFMWGSSKSKYKKREAIEGVIDSDHQSVDHAVAIASVQQNQNLTPTSSVSMWPSSQSLDMRNAHIDIDLMRG

>Gm01g42870.1

MDGREGMAFPGGSVPYYMQHRGGGVSGSGPGTGTQSGGFQPPSGFRALSNVSPGSAFKVESHSYSHSQSQPQHASFSHGINIGSSPDGGGGGPSSGEPVKKKRGRPRKYGPDGSVSLMLSPMSATASSTPGSGTSSEKRPRGRPPGSGRKQQLATLGEWMNSSAGLAFSPHVITVGVDEDIVAKLLSFARQRPRAVCILTGTGTISSVTLRQPASTSIGVTYEGRFQILCLSGSYLVAEEGGPHNRTGGMSVSLSSPDGHIIGGGVTRLVASSPVQVVACSFVYGGSKPKTKQVTTTTTEDTSSEPQSSDKLASPGSVPPPNQNYTSSPAPGIWPASSRPVEVKSAHAHTGIDLTRG

>Gm11g02610.1

MDGREGMAFPGGSAPYYMQHRGGGVGGSVPGTGTQSGGFQPPSGFRALSNVSPGSAFKVESQPQHASFSHGINTGSSPDGGSGVPSSGEPVKKKRGRPRKYGPDGSVSLMLSPMSATANSTPGSGTSSEKRPRGRPPGSGRKQQLATLGEWMNNSAGLAFSPHVITVGVGEDIVAKLLSFARQRPRAVCILTGTGTISSVTLRQPASTSISVTYEGRFQILCLSGSYLVAEEGGPHNRTGGMSVSLSSPDGHIIGGGVTRLVAASPVQVVACSFVYGGSKPKTKEVTTTTTTTKDSSSEPQSSDKLASPASAPPPNQNYTSSSPSPGIWPASSRPVEVKSAHPHTGIDLTRG

>Gm05g23660.1

MDAREPLRPPPPTIMAGPTSYGSANIGPNSSATVSAMLAPATAQFPFAAVPQQHQPPPSSEPFPASAYDGSSSPMKACSLAKKKRGRPRKYSPDGNIALRLAPTHASPPAAASGGGGGGDSAGMASADAPAKKHRGRPPGSGKKQLDALGAGGVGFTPHVILVESGEDITAKIMAFSQQGPRTVCILSAIGAIGNVTLQQSAMTGGIATYEGRFEIISLSGSLQQSENNSERSRTCTLNVTLAGSDGRVLGGGVAGTLIAASTVQVIVGSFIADAKKSSSNALKSGSSSAPPPQMLTFGSSMTPNSPTSQGPSTESSEEQDHSPFCRGPGPGSGHGLYNNASQPVHNMPMYHHPLWAGQSHQ

>Gm05g37880.1

MDGREAMAFSDGSAPYYMHRVGVGGSGSGFQPAPGFRPLSNTGIQAESNARGGQGQGGGSVGSNSPFSVEPPQGHANFNHGIGIGAPSREPVKKKRGRPRKYGPDGAVSLRLSPMSAPANSTQDASETTPSQKKARGRPPGSGRKQQLAALGEWMNSSAGLAFSPHVITIGVGEDIVAKLLSLSQQRPRALCIMSGTGTVSSVTLRQPASTNASVTFEGRFQILCLSGSYLVAEDGGPLNRTGGISVSLSSPDGHVIGGGVAVLIAGSPVQVMLCSFVYGGSKTMSKQATTLKDESSEPPPQHNDKLASPASAPAPPGQNFLPLSAANLWPGSRPAELKSAHMHTGIDLTHG

>Gm17g16640.1

MDVRETPRPPPPTMMVGPTSYGSTNLGPNFSSSSAAAMLAPATARFPFGAVPPPQQHQPPPSSEPFPASAAYDGSSSPMKPCSLAKKKRGRPRKYSPDGSIALGLAPTHTSPPASAAAGGGSAGDSAGTASADAPAKKHRGRPPGSGKKQLDALGAGGVGFTPHVIMVESGEDITAKIMAFSQQGPRTVCILSAIGAIGNVTLRQPAMSGGIATYEVLCSNLKITVIVAEHALLNVTLAGSDGRVLGGGVAGTLTAASTVIVGSFIAFAKKSSSSALKSGSSSAPPPQMLTFGAPMTPNSPTSQGPSTESSEEQDHSPFCRGPGPGHGLYNNASQPVHNMPMYHHPLWAGQSHQ

>Gm11g04610.1

MNSREQPQPQQPPQPQPPNMTVGPTMYPSMFSSSNNNPPPSEPLNNDTNDNDNSAFEALKPCALAASESSKKKRGRPRKYSPDGNIALGLGPTHAPASSADPPAKKHRGRPPGSGKKQMDALGIPGTGFTPHVITAEVGEDIAAKLVAFCEQGPRTVCTLSANGATRNVTIRAPDMPAGTVAYEGPFEIISLKAATLQSDNNRMAALSVSLAGPDGRVLGGEVVGALTAATAVQVLLSNDRSG

>Gm06g01700.1

MSGSDMAGREQFTVGMHKPQQQQQQQQQPQLHQNMRMDYAADGTAVFAPPTVTVNINGGDSSPAVPPGLGLPQPQPMMVNSPEPIKRKRGRPRKYGPDGGMTLGALKTTTPPGGGVPVGQSGGAFPAGPLSDSASAGTVKRRGRPRGSVNKNKKNDSSNSSKYSGPGSWFTPHVITVNAGEDLSARIMTISQSSSRNICILTANGAISNVTLRQPASSGGTVTYEGRFEILSLGGSFFLAGTERAGGLSVSLSGPDGRVLGGGVAGLLIAASPVQIVLASFVSDVRKHLKRAKKTENEKVSTAGGQSSSPSRGTLSESSGGVGSGSPLNQSTGACNNTIENSTTPTQSFQGMPWK

>Gm04g01620.1

MSGSDMASREQFTVGMHKPQPQPQQQQPQLHQNNMRMDYAAADGTAVFAPPTVTVNINGGESSPAVPPGLGLAQPQPQPQPMMVNSSEPIKRKRGRPRKYGPHGGMALALNTTTPPGGAAVPVGQSAGIVKRRGRPRGSVNKNKKNNSSKYSGPGSWFTPHVITVKAGEDLSARIMTISQSSSRNICILTANGAISNVTLRQPASSGGTVTYEGRFEILSLGGSFFLAGTERAGGLSVSLSGPDGRVLGGGVAGLLVAASPVQIVLASFVSDVRKHFKHAKQMQNAKVSIAAGQSSSPSRGTLSESSGGVGSGSPLNQSTGACNNTMNNCTTPTQSFQGMPWK

>Gm20g35480.1

MEGRENFGVVVGDEAPESFHVAPRIENNLDFSRATVPAPAPATEGKKKRGRPRKYGPDGKPALGAVTALSPMPISSSIPLTGEFSAWKSGRGRPVESIKKSSFKFEVESPGPVEGIAYSVGANFTPHVLTVNAGEDVTMKIMTFSQQGSRAICILSATGTISNVTLRQPSSCGGTLTYEGLFEILSLSGSFMPTENGVTRSRSGGMSVSLAGPDGRVMGGGLAGLLVAAGPVQVVVASFLPGHQLEHKTKKQRVEHVSTISPSPVNLITSEEIKVSFGGVKPIMTPAAFQEENIASFNNVQDSRNSSADDKDPLPEKESNLSQSNAEAAC

>Gm03g01320.1

MDRGDQMTLPGSASYYMQRGIPGAGNQPVLHNSPNIGPLSNPNLPCQSSIGGGGTIGSTLPLESSGISAPCVNVSAPSGTLPGETVKRKRGRPRKYGSDGAVSLALTPTPASHPGALAQGQKRGRGRPPGSGKKQQLASLGELMSGSAGMGFTPHIITIAVGEDIATKIMSFSQQGPRAICILSANGAVSTVTLRQPSTSGGTVTYEGRFEIVCLSGSYLVADSGGSRNRTGGLSVSLASPDGRVVGGGVGGVLIAASPVQVILGSFSWGASKTKIKKKEGSEGAEVALETDHQTVHNPVAVNSISPNQNLTPTSSLSPWPASRSLDMRNSHIDIDLMRG

>Gm09g39650.1

MDRGDQMALSGSYYMQQRGIPGSGGQPELHISPNMRPLSNPNLPFQSSIGGGTIGSTLPLESSAISAHGVNVGAPTGAPLGEPVKRKRGRPRKYGTDGSVSLALTPTPTSSSHPGALSQSQKRGRGRPPGTGKKQQLASLGELMSGSAGMGFTPHIINIASGEDIATKIMAFSQQGPRVVCILSANGAVSTVTLRQPSTSGGTVTYEGRFEIVCLSGSYLVTENGGSRNRTGGLSVSLASPDGRVIGGGVGGVLIASSPVQVVVGSFLWGGSKTKNKKKESSEGAEVAVESDHQGVHNPVSLNSISQNQNLPPTPPSLSPWSTSRPLDMRNSHVDIDLMRG

>Gm18g46540.1

MDRGDQMALSGSYYMQQRGIPGSGAPPELHISPNMRPISNPNLPFQSSIGGGTIGSTLPLESSAISAHGVNVGAPTGAPPGEPVKRKRGRPRKYGTDGSVSLALTPTPTSSSYPGALTQSQKRGRGRPPGTGKKQQLASLGELMSGSAGMGFTPHIINIASGEDITTKIMAFSQQGARAVCILSANGAVSTVTLRQPSTSGGTVTYEGRFEIVCLSGSYLVTDNGGSRNRTGGLSVSLASPDGRVIGGGVGGVLIASSPVQVVVGSFLWGGSKTKNKKKESSEGSEVAVESDHQGVHNPVSLNSSISPNQNLPPTPPSLNPWSTSRPLDMRNSHVDIDLMRG

>Gm07g07870.1

MDRGDQMTFPGSASYYMQRGIPGAGNQPELHNSPNIRPLSNSNLPFQSSIGGGGTIGSTLPLESSGISAPCVNVSAPSGAVPGETVKRKRGRPRKYGPDGAVSLALTPTPASHPGALAQGQKRGRGRPPGSGKKQQLASLGELMSGSAGMGFTPHIITIAVGEDIATKIMAFSQQGPRAICILSANGAVSTVTLRQPSTSGGTVTYEGRFEIVCLSGSYLVADSGGTRNRTVALSVSLASPDGRVIGGGVGGVLIAASPVQVILGSFSWGASKTKIKKKEGSEGAEVAMETDHQTVHNPVAVNSISPNQNLTPTSSLSPWPASRPLDMRNSHIDIDLMRG

>Mt5g010440.1

MDEREAMSFSDGSGSYYMHKERVFQQPPPGFRALSNPHGGSDGSTFSVEHEHGSFSHGAVVPYSGEQSVKKKRGRPRKYGPDVPVSLRLSPMSATANSTPDSEKRPRGRPPGSGRKQQLAALGEWMNSSAGQAFSPHVITIGPQEDIVEKLLLFSQHRPRALCVLSGTGTVSSVTLRQPASTSVSVTYEGRFQILCLSGSYLVAEDGGPHNRTGGISVSLSSMDGHVIGGGVARLIAASPVQVVVCSFVYGGSKPKTTKQETAVKDDDDSEPQSSDKLASPGSEPPNQNYTASGTGTMWHGSRTVDVKSTQPHTGIDLMNG

>Mt5g013530.1

MDSHELEPPHPHPHHLQPENVAPNPLAKYVPTTMMELATTQFPILNMESNPSPFFDSFNNGFGPSTLKPCVGASSGSGSIKKKRGRPRKYFLDDNITLSLGSGPIHDATITYPSNSIVKKSTRGRGRPRGSFKKKQEVEVLGVTGTSFFPHLIIVNPGEDIVEKLMTCCQGGSNTEMSILSAHGLVGIVSLHREGRIVTYEDKFELLSLLGTLEPSDNSGGCKKMSNFKVSLLTPNSHLLAGVVVDKLIAASLVKITVGSFTLSGKKASSNNLKVGPSLTPSSQFAAPAGVISQGPSFGSSSGNETSPFSQGSGIYNNANQLIPTISMYQRLLARQVQLVLVDLFTSFQIKHIVYSMLPNFSSTVMESI

>Mt7g084950.1

MALSNSASYYMQQRVLPGSGAQPELHVSPSFNQLSNPNLPFQSNIGGGGSNIGTTLPLESSAISSQGVNMSGHTGVPSGETVKRKRGRPRKYGADRVVSLALSPSPTPSSNPGTMTQGGPKRGRGRPPGSGKKQQLASFGELMSGSAGTGFIPHVIEIASGEDIAAKILTFSQVRARALCVLSSSGSVSSVIIREPSISGGTLKYEGHFHIMSMSGCYVPTENGSSRNRDGGLSISLLGPDGRLFGGAVGGPLVAASPVQVMIGSFLWGRLKAKNKKKESSEDAEGTVESDHQGAHNPAALNSISPNQNLTPTSSLSPWSAASRQMDMGNSHADIDLMRG

>Mt4g149520.1

MDGREAMAFSGGPGSYYMHRGGAGVAGSGSGGFQLPPPGFRPLPNTGIIAQPNARGQGGDTSSMFSLETQSHNSHANFNHGINIGASSGAPSSDPVKKKRGRPRKYGPDGSVSLKLSPTSAPAKSTQEDSTTPSEKRGRGRPRGSGRKQQLAALGDWMTSSAGLAFSPHVITIGVGEDIAAKLLSLSQQRPRALCILSGNGIVTSVTLRQPASTNIGVTYEGKFQILSLSGSYLVAEDSGPSNRTGGISVSLSSRDGHVIGGSVAKLIAGSLIQVVVCSFVYGGGSKVKTKQETAANGESSEPHNDDKLASPASAPPGQNYVSSLTGMWPGSQPSDVKSVHAHTGFDLTRG

>Mt5g014540.1

MDSCEPPHSPHPYQLQPKNIVPVGPNPFTNTSPITMITPTTAQFPLSNINTNPLPQYEHLSLMLFVGASSSGSGSFKRKRGRPRKYFPNGKITLGSSLDPTHAASFASPSSSAVKKNTSGRGRGRPRKYFPNGKITLGSSLDPTHAATFASPSSSAVKKNTSIRGKGKPRGSFKKKLPIEMSGVTNGSGFSPHVIIVNRGEDIVAKVGAFCQGGPNTDMCILSAHGLVGNAALYQSGSVVTYEGRFEIISLSGNLEVSDNTTKFKKMGYFKVSLEGHGSRLLAGVVADKLIAASLVKVTIGVFTLDCKKASSNYLKLGSSSVPPSQIAAFGTLTSDAYQGPSSDSSGDNDNIPFNQLPGINNNATHPIPTMSMYQELWDRQTQQSTGRIPNRGRYIVPIHLVKAPTIIYVVTCHPLSSACEKILDG

>Os10g42230.2

MEAKDVSPLVTVPPAPAAAAPPPAAAPAPPPSQPPPPPLPFAQQAPPPAANPAAAPMRLSFDQMAGKAPGGEQQHHHHPGPMLYAAAPAGGAAPPPQGGNVMGMGELMRKKRGRPRKYAPDGSMALALAPISSASGGAAPPPPPPGHQPHGFSISSPASDPNAKRRGRPPGSGKKKQFEALGSWGIAFTPHILTVKAGEDVASKIMAFSQQGPRTVCILSANGAISNVTLRQPATSGGLVTYEGRFEIISLSGSFLLAEDGDTRSRTGGLSVALAGSDGRVLGGCVAGMLMAATPVQVVVASFIAEGKKSKPVETRKVEPMSAPPQMATYVPAPVASPPSEGTSSGSSDDSGSPINHSGMPYNHSGQQQQHQQHQHMPPAYASGGWSLSAHHQNRHDSDMKMMSN

>Os04g49990.2

MDGREQQQQPRVSSPPPAGGGVMMPQHPYGAAPAMPPGSANVMHGVPLSFNPMASPTASSPMKPADMSGTMYRTDPVVQGMQQQPGSGGGGTAVGGGELVKKKRGRPRKYGPDGNIGLGLKPAAAAGTEAGGPSGGAGSNSNPDGKRRGRPPGSGKKKQLDALGSSGTSFTPHIITVKPNEDVASKIMAFSQQGPRTTCIISANGALCTATLRQPATSGGIVTYEGHFDILSLSGSFLLAEDGDTRSRTGGLSVALAGSDGRIVGGCVAGMLMAATPVQVVVGSFIAEGKKGKEEHLKREPTSAPTPNHAAGFGAATAASPPSDGSSSDHSDDPGSPMGPNGSTFNNSGHPMHSSYAPVSWSLSGNQGRYDPDLKMMTD

>Os04g58730.1

MDGKELLSPSELSYYAHQQHQHQHQQHQQQHRMLGGGGGGGGHSASPLAGMHGGPSVIRPMPNMGMSPTAILQSIGPGPLAGMQFQMDAAPPPPPLMHNSMASVSASAGAGSPTVPPSATPMEPVKRKRGRPRKYGPDGTMKVSTAAAAQHQQQMLSAPPRMGSVSGADMVGGGSGMDDSAQKKRRGRPPGTGKKQQLSSPVKLSGGNAFSGSAGTSFTPHIITASPSEDVAGKIVAFANHSSRAVCVLSATGSVSRVVLRHPADGAMSRVHASSHYKNPAIYEGLYEILSMSGCYNLMNEGQSDGLSVTLCSPERHIIGGVLGGALVAASTVQVVLGSFVQGGSKPKSKKAGKQQQQQAAAAAFSSDSLTGGGQDASPSSGHNQNLTPPPPVTTTGGWPSSGIFDTRSSNIDINSSRG

>Os02g03270.1

MEPATETTATSPAAAAAAQPEAEAEAEAESAATPVAVAAAAAATYQPPAPVVPVAVAGAAAGRGNGKRKRGRPRKYGPDGSLLRPLKATPISASVPDDSGGGQYTPAAAVGAVMKRGRGRPVGFVSRASPVSVAVTAATSTAAVVVSSPATHTQTPLGPLGELVACASGANFTPHIINVAAGEDVNMKVISFSQQGPRAICILSANGVISNVTLRQQDTLGGTVTYEGRFELLSLSGSFTPTDSGGTRSRSGGMSVSLAATDGRVIGGGVAGLLVAASPVQVVVGSFLPSYQLDQNATKKPVIEITTVPPPPPAIGFTISSGDPMEDSYSGSHGQHRSGAAAATTTTAKANSTSAFRVENWTPPAPPAAEAARTKTPSSEAKVPVPGA

>Os02g57820.1

MDGRESTVASGSNFSSFYVQHRGIGVPGGSGHPAGLHGPPPGGYRQHLDAVSAGYPFQPPHIGGSHIGQGYHHVDASAPVAQHGSGGGGGGMDIGMGVEMSADAKGDQGSGAGQDEPVKKKRGRPRKYKPDGAVTLGLSPSSSTPHSSTSAMGTMVTTPGSGFGSGAGSGGSGSGALTEKRGRGRPPGSGKMQQLASLGKWFLGSVGTGFTPHVIIISPGEDVAARIMSFSQQGPRAVCIISATGAVSTATLHQDSNSGGVVTYEGRFEILCLSGSYLVIEEGGSRTRSGGLCIALCGPDHRVIGGSVGGVLTAAGTVQVIVGSFMYGGTKKNKAKAEQETENNEEPIGGEEETPTMALPDHNMPHHTMGGWSAGLMRQMDSRTPNIDINSIRE

>Os08g02490.1

MEAKSGEASVAPVAVATEATAATVSFQPQAAVAEQGSSSGGVLVPPPPMAAGGGGVVVAAAPVAGVVKVGKKRGRPRKYGPDGSLIRPLNATPISASVPMAASAVGPYTPASAVGAAMKRGRGRPLDFASTAKLHHHHQHQHHHQQQQFGFHFDSIGEMVACSAGANFTPHIITVAPGEDVTMKVISFSQQGPRAICILSANGVISNVTLRQPDSSGGTLTYEGRFELLSLSGSFMPTENSGTRSRSGGMSVSLASPDGRVVGGGVAGLLVAASPVQIVVGSFLPSYQMEQKNKKPRVEAAPALAQTPPAVPISSTDTHSSEQGQHSSVAPRTTNIVTSAYNPDQSWASPAQSIPDSARTPSGDVKVTASGA

>Os08g40150.2

MEVRSEQGLMAGRDLFGMPKSPPAAAAAPPQSVRMAYTSDGTPVFAPVSAAVSAPPGYQPGGAAGGNGAAALADSGGEPVAKKKRGRPRKYGPDGSMSLGLVTSPTAAASTPVAQGVPGPFSPTQPKPPASFLSSGWPDGVKKRGRPKGSTNKPRIDAVGSAGVGFTPHVITVLAGEDVSAKIMSFAQHGNRAVCVLSANGAISNVTLRQTATSGGTVTYEGRFEILSLSGSFLLTDHGGQRSRTGGLSVSLAGPDGRLLGGGVAGLLIAATPVQIVVGSFNSEGKKEPKQHAHSEPASAPSKAVPTAGMGPNSPPSRGTLSESSGGAGSPLHPGIAPPSSNSQPPFLSSMPWK

>Os09g31470.1

MMEVTQVRASSEMAGGREPFVLPKSPPAAAPAPPPPSSGGMQSVRMAYTADGTPIFAPVNSAPAPAPAATYPPAGGNGAAALDAGEPVVKKKRGRPRKYGPDGSMSLALVPVSTAAVAASGPFSPAAAAKSPDAVSSAPPPGAKKRGRPKGSTNKKHVPSFGIGDIGSAGAGFTPHVIFVKAGEDVSAKIMSFSQHGTRGVCVLSANGAISNVTLRQAATSGGTVTYEGRFEILSLSGSFLLSENGGHRSRTGGLSVSLAGPDGRVLGGGVAGLLTAASPVQIVVGSFNTEGKKGPKLHAPSDPMSAPLKMVPMSGTGPSSPPSRGTLSESSGGPGSPLNQGVTASNHGQPGLPSLSWK

>Os06g22030.1

MQGGAMSAAATATASEAAAYGGVGMSKSGALQPQPPHGAAAAVRLAYTHDGIAVYKHTPPPPVYQTPAAVAAPSPPVRGNGGAPASAEQHKRKRGRPRKYAVTDVPLAVVPPSPPKAAAAAGASAAQSPATPTLPPGFSSGLAAYGGAAASQPAPRQAPPASGRVLPHKKRGRPPGSGNKQQQRPQHKKAAAPGSSVIGLKPSVITVQVGEDVVSRVMSFTKNGWAVCVLSANGAVSNMTLRQAGSSGATTVNYEGHFEILSLSGSYLLSESVGLSSRAGGLSVSLAGPDGRVLGGGVAGPLNAATPVQVVIGSFLADVKKGHKQAMPSGAPYPGVSTPTSRGTPSGSSGGPGSPLNQSASGSFNTSNQQALADFPWR

>Pp1s190_33V6.1

MQDPTDVSQRPGIEGMSSPQRGGMAGGFAQGLHTPALVRHQPPPSSLPAISLSQGAQVGAPGMSTSVFETGPVGPHPRSIIAARPPSSSGINMGMIATPIVGAGGETRGEQPPKRKRGRPRKFATGGELSSGALGSVYPVLPALMPASSSPYTPSPEKRGRGRPPGSGKKQQLAALGVVLAGTGQGFTPHILTVSTGEDVSTRIMQFAQHGPRAMCVLSANGAISNVTLRQQSSSGGTVTYEGRYEILSLTGSYLSTELGGGARQRTGGLSVSLAGSDGRVIGGGVAGMLTAASPIQVVVASFLSDTFKAQAKGESPLGSTPGGSSGAGSAPGSTPGSALGGQRPPTARSEPKTSSALGPRQVSPVPPPTGQVSVQRPQVMSMFQPMAGWQPAVPGDGRRTDINISLPGG

>Pp1s469_4V6.1

MVGMQESADAANRSGGGGMGSPQRAGMASGVGHGLHTAAPVRHSPPPSSSPSPSPGITPSQSAQGGAPAVSTPVPEAGPSGLHPHPIMAVAPPASLGMSLGIVGTSITGPRSASGGEQPLKRKRGRPRKFSTGSEFSPGTPGAGYPVFPAIMPAPSSPYTPSPDKRGRGRPTGSGKRQQLAALGVVLAGTGQGFTPHILTVNTGEDVATKIMQFAQHGPRAMCVLSANGAISNVTLRQQLSSGGTVTYEGRYEILSLSGSYLPTDLGGGARQRTGGLSVSLAGSDGRVIGGGVAGMLTAASPIQVVVGSFLSDAYKSQPKSDSPLSSTPGGSSGGGFVPGLGLGGPRPPPLRPEMKPSPGLFSNSKAPSPQQTSPAPPSPTGQVSAQRPQTMGLFQPMASWQPPALGDGRRTDINISLPGG

>Pp1s157_16V6.2

MEGRDLVEGMQRPGGGSGGSMSSPQSGGMGGVPGQGLHTPTAIRPPPPPSLSSITPLHSMSSALPESGPSGPRPIKTVAPPSSSGMSMVMVDPGAGGGNGSGSGAGAEQTRKRKRGRPRKYETGAGLTPGVPGGGFPVLPSLLPGPSSSPYSSPDRRGRGRPLGSGKKQQLAALGVVLAGSGQGFTPHILTVNTGEDVATKIMQFAQHGPRATCVLSANGAISNVTFRQQSSSGGTVTYEGRFEILSLSGSYLPTDLGGGARQRTGGLSVSLAGIDGSVIGGGVAGMLTAASPIQVVVGSFLSDSFKTQPRSKSPLSSGPGGSSGAGTAPGSAPGAQRPTPPRLEPKPSSASGPQQAPSVPSLPGQSSVPRPQAMSMFQPTFTWQFPPVSGEGRRTDINISLPGG

>Pp1s11_60V6.1

MNSSQPPAEGTRPAPASAPAPVLPMPSALVMSMGMALGGVSSRGETVKRKRGRPRKYVGNEPGGAASAAGGTPVNMQLALHTPNSGPSGSPFTPTGVKRGRGRPLGSSRKLHQLVSFPSAGSWAGQNFTPHIITIAAGEDIAAKIYSFAQHGPRAVCVMSANGAISTAILRQQSSSGGNVTYEGRYEILSLMGSFLPTEQGANSRQRTGGLSVSLACSDGRVIGGGVAGVLTAASPIQVVVGSFIFEPEKAAVKVGNGQQPSMGYSLGADFSAALTPASAPRPLKTSPVSATPASTPAHQQAVTPPSHPSSGQSSQQLFHQSMGLFQQPMPWSNPPLAEARRTDINISLPGG

>Pt0001s09300.1

MDGREAMPFSSGSSPYHIHRGSGFLGPGYGSQHGVSHPPPGFRSLSNPQLAAQSNVRSGSTVPAFSIEPPDVNFGHGINMAATSEVQVGEPVKKKRGRPRKYGLVGQVSLGLSPLPNKPKPSSGEDSSTSKRNRGRPPGSGRKQQLATLGEWMNSSAGVAFSPHVISIEVGEDIVSKLLSFSQQRPRAVCILSGTGTVSSVTLRQPASSGSSITYEGRFEILCLSGSYLVAEDGGPRNRTGGISASLSSPDGHVIGGAIAMLIAASPVQVVACSFVYGVSKKDKQVSHPINEKDSTSWPDDNLDNLKAVTPTSMPPQSFTSSPTSIWPGPRAVDMRNPHTDIDLTRG

>Pt0003s12660.1

MDGRETMAFPSGSSSYYIHRGSGILGSGSGSQHDPLHPPTGFRSLSSPHLASQSNVRPGSSAPAFSIEPPNANFGHGINMAATSEVQVGEPVKKKRGRPRKYGLDGQVSLGLSSFPDKAKPSSGEDSSTSKRNRGRPPGSGRKQQLATLGEWMNSSAGLAFSPHVVSIGVGEDIVSKLLSFSQQRPRAVCILSGTGTVSSVTLRQPASSGPPITYEGRFEILCLSGSYLIAEDGGPRNRTGGISASFSSPDGHVIGGAIAMLIAASPVQVVVCTFLYGGSKKDKQVGRPKNKKDSASQLDDNSANLKSATPTSTPQSFTPSLISVWPGPRPADIRNPHTDIDLTRG

>Pt0012s14350.1

MIYEVMEAKEGIAVSSGVTVKAEEAPDGFRVAPRNENSSPSPNPNPNPNQNNNHNPNPNSNSNSNSNPSPNPDPGQLGAPQVGASPVSAVGTDTAGKKKRGRPRKYAPDGTLALALSPMPISSSIPLTGDYYAWKRGRGRPLESVKKQHNYEYESTGDKIAYFVGTNFMPHVITVNAGEDVTMKVMSFSQQGARAICILSANGTISNVTLRQPTSSGGTLTYEGRFEILSLSGSFMPSENGGTKGRSGGMSVSLAGPDGRVVGGGLAGLLVAAGPVQVVVGSFLLGHQQESKHKKQRIEPALAVIPATIPATINVISPEEMKGSYGGVRPIAIPSPLHGDNPASLNPMQA

>Pt0002s00650.1

MSGSETGVMTSRDPFSVTGLQHKTEVPSQPVIQNMRLAFSADGAAVYKPITTATTTTAASPTYQPGGVEGSAVGASVSPHWINVGGSGGDPMKRKRGRPGKYGPDGTMALAIASAPQSVAVTPLTSSGLSSPPAQAQAQVQPLVPTPSPGSDVGVAGPAVALGGSVSPTGVKKARGRPPGSSKKQQLDALGSAGIGFTPHVITVKAGEDVSSKIMSFSQHGPRAVCILSANGAISNVTLRQQATSGGTVTYEGRFEILALSGSYLPSENGGQRSRTGGLSVCLSGPDGRVLGGSVAGLLMAAAPVQVVVSSFIADGRKVSKSANHMEPSSATSKLPPTGGSTGVSSPPSRGTLSESSGGPGSPLNQSTGACNNNPQGISNMPWK

>Pt0014s07790.1

MDRRDAMAISGSASFYMHRGITSSGSMNVSSNINTLSNTNVAFQPNIGANTMGSTLPMEHPVAISPHGVNVGVPSTMPPSGEPVKRKRGRPRKYGPDGAVSLALSSSLSTHPGTITPSQKRGRGRPPGTGRKQQLASLGEWLSGSAGMGFTPHIITIAVGEDIATKIMSFSQQGPRAVCILSANGAVSTVTLRQPSTSGGTVTYEGRFEILCLSGSYLLTNDGGSRNRSGGLSVSLASPDGRVIGGGVGGVLIAASPVQVIVGSFLWGGGSKTKNKKVEGPEGARDSDHQTVENPVTPTSVQPSQNLTPTSSMGVWPGSRPVDMRSTHVDIDLMRG

>Pt0002s15960.1

MDRRDTMTISGSASFFMQGSGTHPSLNVSSGINTLSNINAPFQPNMGANTMGSALLMEHPAAISVGELSTMVSGQPEKRKRGRPRKYGPDGAVSLALSPSLSTHPETSIPSQKRGRGRPPGTGRKQQLASLGEWLSGSAGMGFTPHIITIAVGEDIATKIMSFSQQGPRAICILSANGAVSTVTLHQPSTSGGTVTYEGRFEILCLSGSYLFSKDGGSRNRTGGLSVSLASPDGCVIGGGVGGVLIAASPVQVIAGSFLWGGSKTKNKKVEGAEVARDSDHQTVENPVTPTSVQPSLNLTPTSSMGVWPGSRSVDMRNTHVDIDLMRG

>Pt0004s20060.1

MDYMEEKNIIVSDETPIVTTTKDHAPPGSQVATGGSDPTLEPNNPGGGVVGGSGGSGSEGVVESTVKRKRGRPRKYDVDANLVSSPPPPQGLSSSLSSYEKRGRGRPRGSGKLQLLASLGGFAAETAGGSFTPHVVPVYTGEDIVSKIIELSQKGARAVCILSATGVVSSVIMRQPGPSGGILRYDGRFEILSLSGSFTFGETGGSNRKNGMLSVSLAKPDGRVFGGGVAGSLIAAGPIQLVIASFKQNIGKGIKRRQSADPPAAPSLPANSDVVRVPVKIAGTTDGEDNCTTPTSALSEPRNEEAGNTVISNQQANTDSQNSSGQNVLQSQKQILLVSPGYENL

>Rc29092.m000442

MEEKESTVSGSPMGSETDSPQPVSSMVAVEPPPQQVMMNINMNMGAPGENVMILERSSNVSATTAASIASSGGSGGGGGGNNNNNSLDLFGKKKRGRPRKYDSEGNLRVQPFNHYQAVSAATGALTSPPPTTPAFSFSPSPPDHGFNSSSKRGRGRPPGSGNWQLLASLGELFANTAGGDFTPHVVTVNTGEDVAGKIHSFAQKGPRGICILSANGAVSNVTIRQPGSSGGILTYEGRFEILSLSGSFTVSENGGVRSRTGGLSVSLASPDGRVIGGGIAGLLLAASPIQIVMGSFMPNGYKVHKKKHHRENTVIRGTQGVVSEASPISQAKPNGETCLISASPVPEQSHGGTENSANDQQIPNATNSLSVCWNGSEPTSDQRPSPDINVSVLNEEDVQGAPYTISLL

>Rc30.m013803

MSGSETGVMTSTTREPFGVVSPQPVIQNMRLAFGADGSSVYKPMTTATNSPSYQPSPSAASPGGFVEGGSLGINVNMGSGNDAMKRKRGRPRKYGPDGTMALALVSAPQSVGITQPAGGGGFSTPTSAAATSVGPSTTTIAANPSLPSGSGGGSVSPTGIKKGRGRPPGSNKKQQLEALGSAGFGFTPHIITVKAGEDVSSKIMSFSQHGPRAVCILSANGAISNVTLRQPATSGGSVTYEGRFEILSLSGSFLPSENGGQRSRTGGLSVSLSGPDGRVLGGGVAGLLLAASPVQVVVASFISDDRKELKSPNHLEPLSAMNRLTPVMGTTGPSSPPSRGTFSESSGGPGSPLNQSTGACNNSNLQGISSMPWK

>Rc34.m009012

MDRRDAMAMSGSASFYMQRGMTGSGSGTQSGLNVSSGINPLTSTNVSFQSNVGANTIGSTLPLETSTAIPPHGVNVGASSLMPPPGEPVKRKRGRPRKYGPDGTVSLALSPSLSTHPGTITPTQKRGRGRPPGTGRKQQLASLGEWLSGSAGMGFTPHIITIAVGEDIATKIMSFSQQGPRAICILSANGAVSTVTLRQPSTSGGSVTYEGRFEILCLSGSYLVTSNGGSRNRTGGLSVSLASPDGRVIGGGVGGMLIAASPVQVIVGSFLWGGSKAKNKKGEGPEGARDSDHQTVENPVTPSSVPPSQNLTPTSSIGLWPGSQSLDMRNTHVDIDLMRG

>Rc27.m44

MDSREAQQHQHQHQQPPHPQQQQQSNMMLGGYSNNAHPAMTMINPNIPPSGFPFNSVGPPRTQPSKQPSSDGGLFDGSSPPSSSGMRFSMDPAKKKRGRPRKYTPDGNIALGLSPTPISSSATSLPPHVADSGSGVGVGIGTPAIASDPPSKRNRGRPPGSGKKQLDALGGVGGVGFTPHVITVKAGEDIASKIMAFSQQGPRTVCILSANGAICNVTLRQPAMSGGTVTYEGRYEIISLSGSFLLSENNGNRSRSGGLSVSLAGSDGRVLGGGVAGMLMAASPVQVIVGSFIADGKKSNSNIHKSGPSSAPTSQMLNFGAPMTTSSPPSQGVSSESSDENGSSPLNRDPPIYSNATQPLHNMNMYHQLWAAQNPH

>Rc28592.m000283

MSGYGGGGGGGGGGEIERNQSPTVTTTATTSNLAFRADGSVYKPIMPPTDDGNPFHHVPLHYNHHQDMMVGGGGGGGIGIGLDALNMNNNHSEPIKRKRGRPRKYSPPPHGNIDLTSPPQHQLYQCGFQSPTPSSTAPKKARGRPPGSARKNHLPNLGSGGTGFTPHVIFVKAGEDVLLKIMSFSQNGPRGVCILSAYGTISNVTLRQATTIGGTVTYEGRFEILSLSGSFLLSENSGQRSRTGGLSVLLSGPDGRVLGGGVAGLLTAASSVQVIVGSFISEDSKGSKLWINQHETMSAPGASVAGSPPSRGTFSESSGGPGSPPNQSTGACNNSNTQGMPNVAWK

>Rc29991.m000634

MEDNNSNTVSAATQVSPATTKEDDQQVREAGQDCGGGTVEAYLESDHIRVSSRRAEITVKRKRGRPRKFDHHHHHHHIQMDHENTMSNVSPSSSNFLRSCEKRGRGRPRGSGRLQLLAALGGFAAETAGGILIPHVITVNTGEDIVSKISSFAQRGPRAVCVLSATGVVSCVIIRQPGSSGGLLRCEGHFEILSLSGSFTFRETSTARRKIGVLSVTLAKPDGQVFGGGVVGSLIASGPIQLIVASFKQNISKELKLRQSSESSTCSVPGNTEMVRVPIQIVGITDGGGDQENCTPTSPVVEPRNAEAENTIITKQHMKPVPQNGSGQTVVQSPQQISDEKVSPDINVSVAEI

>Sb07g027510.1

MEVRSEQGLMAGRDLFGMPKSPPAPVPGPPASAAMQSVRMAYTADGTAVYAPVSSSAATPSYQPQGPAHGTSMSAATVVGGNGASAVPGMGEPVAKKKRGRPRKYGPDGSMALALVPASAATGSPATGSPATGQGSSGPFSPAGLNPASSLLVVSPDGFKKRGRPKGSTNKPRVDAAGSSGAGFTPHVITVQAGEDVSSKIMSFSQHGTRAICVLSANGAISNVTLRQTATSGGTVTYEGRFEILSLSGSFLLVENGGQRSRTGGLSVSLAGPDGRLLGGGVAGLLIAASPVQIVLGSFNSGGKKESKKHAPSEPTSVPLKVAPTTGMGPNSPPSRGTLSELSGGTGSPPPLHQGMAASNNNQPPFLSSMPWK

>Sb01g028400.1

METKDVSPLPATAAAAAAAPVPAPASQPPPAPPSMAPPPQQHQHQHQHQHQPPPPFAQQAAPASSPAGSMPGGMRLSFDQMSGKAAGEQHHHSAPMLYAASPQSAPGAGAPGAPGANVLGMGELMRKKRGRPRKYAPDGSMALALAPISSASAGGAAAPGQQQQHGGFSISSPPSDPNAKRRGRPPGSGKKKQFEALGSWGIAFTPHILSVKAGEDVASKIMTFSQQGPRTVCILSANGAISNVTLRQPATSGGLVTYEGRFEIISLSGSFLLAEDGDTRSRTGGLSVALAGSDGRVLGGCVAGMLMAATPVQVVVASFIAEGKKSKPAEARKVEPMAAPPPQMATFVPAPVATSPPSEGTSSASSDDSGSPINHSAMPFNHSSQHQHPHQHQHMPPAYASGGWSLSVHQQNRHDSDMKMMSN

>Sb02g028500.1

MMEVSPSPEQGVMAGREPFGLPKTPATPPSSGVTQSLRMAYTTDGTAIFTPVSSAPPATATYHQPVAASSLAGVGGNGGAPVHSGGAGEPVAKKKRGRPRKYGPDGSMSLALVPVPASIAAAPAPAPAAPGASGPFSPSGPKALNTAPSASPDGAKKRGRPKGSTNKKHVPALGPTGAGFTPHLIFVKAGEDVSAKIMSFSQHGTRAVCILSANGAISNVTLRQSATSGGTVTYEGRFEILSLSGSFLLSENGGHRSRTGGLSVSLAGPDGRVLGGSVAGLLTAASPVQIVVGSFDADGKKEPKRKKLAPSPSDPSPAPLKLAPATTGVAAGPSSPPSRGTLSLSESSSGAPSPPHAGASGGGGGHGQQQQPGVFSGLSWK

>Sb10g012730.1

MSSAMAAASEAAYGGGVQKGSLQQHQAPQPSPGSVFYTPDGIAVYAKPAIPPFYQQSAGSNAIVPAAPGLAHSSATSEPFKRKRGRPRKYGPADGAVPLAIVPPSQPPTAAAPAASEASPTIPPGFAPSPQGGGVVSPQASPAPQPPAASGAPAVKKRGRPPGPSSKKQQPQAAAPGPGWAGWKPHIFTVQAGEDVASRVMSFSGNGWAVCILTANGAVSNVTLRQGESSGGTVTYEGRFEILSLAGSYLLSESAGMSSRTGGLSVSLAGPDGRVLGGAVAGPLTAASPVQVVIGSFLADTKMELDPGSAPEKHAFGRFPTASSPSRGTESLGGHASPPNTTGSFSTSTQPPGFPSFPPWK

>Sb06g026920.1

MRKHATALGDYVTAPGMPQASTGNVRHWKGLAFSSTMERSPGTWSPMNMKPTDMPPGAMYRADSDAPPGLQQQQHPGSGGGGAIVAVSGGELVKKKRGRPRKYGPDGSIGLGLKSAAAAGTEAAGGQSGGGGGSSSNPDGKRRGRPPGSGKKKQLDALGSSGTSFTPHIITVKPNEDVASKIMAFSQQGPRTTCIISANGALCTATLRQPATSGGIVTYEGHFDILSLSGSFLLAEDGDTRSRTGGLSVALAGSDGRIVGGCVAGMLMAATPVQVVVGSFIAEGKKPKEEQPKREPTSVPPHTAGFGAASTASPPSDGTSSEHSDDPGSPMGPNGSTFTNAGLPLHSTFAPAGWSLSGNQSRYDPDLKMMTD

>Sb06g033450.1

MDGKELISQSDLQSFYQQQQQQQQHRAFGGGGGHHSPSSLAGMHSVIRPMPNMPNMNMSPTAILNSIGGGSLAGMQFQMDPPPPPLLHTTTAAAAMGGSASGPGAVPPVHAEPVKRKRGRPRKYGPDGTMKQQQQQQQAAASQQHLVAAPPRMGSLSSGPDMLGGSGMEDAAQKKRRGRPPGTGKKHQPSPSQGNAFAGSAGTSFTPHIITVSPSEDVAAKIVAFATQSSRAVCVLSAMGSVSRAVLRHPADGSPMARVHAASPQPYKNNPAIYEGFYEILSLTGSYNLAEGSQAQGQGQQSGGLSVTLCSPERNVIGGVLGGPLVAASTVQVVLGTFHQGGSRSKSKKAGKLQQQQQAATATAAAFSSDSLTGGQEASPSSGHNQNLTPPPSVTGGWPTSGIFDTRSSSIDINSSRG

>Sb04g002140.1

MEITEASPSPAPAAQPAPAPAPETATTMTTLASSQPAPAAPEAALSVAAVAGRGDGKRKRGRPRKYGPDGTPLRPLNATPISASAPDDAGVGQYTPAAAVGAVMKRGRGRPVGFISRVTPISVAVTAAAPTPAVVVSAPPPAPAPAPHSQLAPLGELVACASGANFTPHIINVAAGEAPHIEILKEELQTSRNAATTLRGRFELLSLSGSFTPTDSGGTRSRSGGMSVSLAAADGRVIGGGVAGLLVAASPVQVVVGSFLPSYQMDQNANKKPVIEIKTVPPPPPATVGFTISSGDMDDAYSGSHQPRSVGAKGSSTMALFKVENWTAPAPDQAKKTPPPPPTSEAKVPVPGG

>Sb07g001760.1

MAVAPVTAAPPEATVSFQQAAPAAEMGSSSAVLVPPLATAAAVAGGGGGAMALGPVLMKVPKKRGRPRKYGPDGSLIRPLNATPISASAPMPTAVAPGQYTPASAVGAAMKRGRGRPLDFAAAAAKQQQQQQQHHHQHHHLQHPNVLAGDMVACSAGANFTPHIITVAPGEDVTMKVISFSQQGPRAICILSANGVISNVTLRQPDSSGGTLTYEGRFELLSLSGSFMPTENNGTRSRSGGMSVSLASPDGRVVGGGVAGLLVAASPVQIVVGSFLPSYQMEQKNKKPRVDAAPATVPQTPPAVPISSTDTHSSEQGQQAQRGMTSTGAYGADQSWASSAQQPMAEVSRTPSSGDLKMTASGS

>Sb04g037880.1

MDGRESTVTSGPNFSSFYAQHRGIGAPGVPGHSSGLHGPPPGGYRQHLDAVSAGYAFQTPHVGGPHIGQGYHHVEASHHVAQHSAGGGSSSGGGGMDIGMGVAVSADVKGDQGSGPGQDEQVKKKRGRPRKYKPDGAVTLGLSPSSSSTPHSSSPGMGTMVCTPGSGFGSGASGGSGSGAPSEKRGRGRPPGSGKMQQLASLGKWFLGSVGTGFTPHVIIIQPGEDVAARIMAFSQQGPRAVCIISATGAVSTATLHQDSDSGGVVTYEGRFEILCLSGSYLVLDDGGTRTRSGGLCIALCGPDHRVIGGSVGGVLTAAGTVQVIVGSFMYGGSKKNKAKAEADIEPEEANAGDEEVAPAMALADHSSMAPPPEMSGGWASGMMRQIDSRTPNIDINSIRE

>Vv01013426001

MSGSETGIMTTREPFSMGLQKNAVPSQPVIQNMRLAFSPDGAAVYKPVSGTSPPYQSSGGTGGDGSTGGAIIPHGLNMNMGSEPLKRKRGRPRKYGPDGTMALALSPAPSGVNVSQSGGAFSSPPASAGSASPSSLKKARGRPPGSSKKQQMEALGSAGVGFTPHVITVKAGEDVSSKIMSFSQHGPRAVCILSANGAISNVTLRQPATSGGTVTYEGRFEILSLSGSFLLSENGGQRSRTGGLSVSLSGPDGRVLGGGVAGLLTAASPVQVVVGSFIADGRKESKSASQVEPSSAPPKIAPVGGGGGVTGTSSPPSRGTLSESSGGPGSPLNQSTGACNNSNPPGMTSIPWK

>Vv01013200001

MDSHEPQQPQQQQHPPHGMMMGPNSYHTNMANTSPMMNPNSAAIMQNNRFSFTSMVASKPVDSPYGDGSSTGLRPCGFNIEPAKKKRGRPRKYAPDGNIALGLAPTPIPSTAAHGDATGTPSSEPPAKRNRGRPPGSGKKQLDALGAAGVGFTPHVITVNVGEDIASKIMAFSQQGPRTVCILSANGAICNVTLRQPAMSGGTISYEGRFDIISLSGSFLLSEDNGSRHRTGGLSVSLAGSDGRVLGGGVAGMLTAATPVQVVVGSFIADGKKTNTNQSGSSSAPPAQMLNFGAPVVPASPSQGGSSESSDENGGSPLNRGPLPYNNVSQPIHQMPMYAAMGWPNSTMKMLPN

>Vv01026888001

MDRRDAMAMPGSGSYYMQRGMAGSGSGSGPQPGLHGSPGIRSLSNPSMPFQPNIGGGGSMGSTLPVEPSSVISTHGVNVGAPSTLLPPSEPVKRKRGRPRKYGPDGTVSLALSPSSATSPGTLTASTQKRGRGRPPGTGRKQQLASLGEWLSGSAGMGFTPHVITVAVGEDVATKIMSFSQQGPRAICILSANGAVSTVTLRQPSTSGGTVTYEGRFEILCLSGSYLLTDNGGSRNRTGGLSVSLASPDGRVIGGGVGGMLTAASPVQVIVGSFIWGNSKTKNKMGESVEGAGDSERQTVDHPITTPTTVPASQNLTPASSMGVWPGSRQLDMRNSPVDIDLMRG

>Vv01033765001

MERSLGGGDDGGGVGGNVGGRISGGGGGGSGSFDLLGRKKRGRPRKYDADGNLRLSYAVSPPPGFTLSSPSSDFSSKRGRGRPPGSGNWQLLASLGELFANTAGGDFTPHVVTVNTGEDVASKILSFSQKGPRGICVLSANGAVSNVTIRQPGSSGGILTYEGRFEILSLSGSFTVSDSGGARSRTGGLSVSLAGPDGRVIGGGIAGILTAAGPIQIVVGSFMPNGYKTHKRKHHREPTTTSIIPPAPDTVTAARPISQAAPEVGPCLNSTSPSHGQSHGEADDSVNNKQISNVISLQSAAWNGSEHKSEQRPSPDINLTVPSE

>Vv01027617001

MEGREGMTSGVTVIGAEAPSDYEMVARTENPSQIAGSPAVDASPVSVGFTGTVGKKKRGRPRKYQPDGMASMTLSPMPISSSAPLSGNFSSGKRGRGRPVGSESKQKQKVGSENSGNWSAISDGVNFTPHIITVNAGEDVTMKLISFSQQGPRAVCILSANGVISNVTLRQQDSSGGTLTYEGRFEILSLTGSFVPTESGGTRNRAGGMSVSLASPDGRVVGGGVAGLLIAASPVLVVVGSFLPDNAPVQKPKKMKSVSAQTATPVSVQTTTPPVVTSTPKEEGVGGQGQPSSSALKPDIASPSSIQRENWASMQSMQDSRKSGTDINISLPGG

>Vv01018513001

MEGTEGINSGVTVKGEEAPDTYRVAARSENPSEFGGSTMTAVVAMPSSEMKKKRGRPRKYGPGGSLTMALSPMPISSSIPLTGEFSAWKRGRGRPVDSFKKQHKSESESAGERVAYSVGANFTPHVITVNAGEDVTMKIISFSQQGSRAICILSANGAISNVTLRQPNSSGGTLTYEGRFEILSLSGSFMPSESGGTKSRSGGMSVSLAGPDGRVLGGGLAGLLVAAGPVQVLVGSFLPGHQQEQKPKKQRIEPVQAAIPATVNSMPREETLGANGGPNLNLTSPSSFHGDTWASLNSMQGSRNLDIENKLPVSEGETKGPSQ

>Vv01019763001

MDGREAMALSGSPPYYIHRGVVGSASLSGSVDHSQTNFPHGFNMAVPSGVPPAEPVKRKRGRPRKYGPDGNVSLGLSPMSARPSLGSGSVTPTQKRGRGRPPGTGRKQQLATLGEWMNSSAGLAFAPHVISMAVGEDIATRILSFSQQRPRALCILSASGTVSAVTLRQPTSSSGTVTYEGRFEILCLSGSYLPAETGGPRNRIGGISVSLCSPDGHVIGGGVGGMLIAASPVQVVACSFVYGGSKTKNKNGDEPKGDQNSGLQPSESAAPSSVPLGQHFAPISAMGMWPSSRQVDLRNPHTDIDLTRG

>Zm2G072117_T03

METKDVSPLAAATTTTAAPVPAPTSQPPPPAPSSMAPPQHQHQHHPPPPFAQQAAPVPSPAASMPGGMRLSFDQMSGKAPGEQHHHSAPMLYTVPPPQSAAGAGAPGANVLGMGELMRKKRGRPRKYAPDGSMALALAPISSASAGGAAAPGQQQHGGGFSISSPPSDPNAKRRGRPPGSGKKKQFEALGSWGIAFTPHILTVKAGEDVASKIMTFSQQGPRTVCILSANGAISNVTLRQPATSGGLVTYEGRFEIISLSGSFLLAEDGDTRSRTGGLSVALAGSDGRVLGGCVAGMLMAATPVQVVVASFIAEGKKSKPAEARKVEPMAAPPPQMATFVPPPLATSPPSEGTSSASSDDSGSPIHHSAMPFSNSSQHQHPHQHQHQHMPPAYASGGWSLSVHQQNRHDSDMKMMSN

>ZmAC225193.3_FGT003

METKDVSPLAAAAAAPVLAPASQQQQQQPPQPQQHHHQPPQPQHQHQHQPPPPPFPQQAAPAAASSPAASMPGGMRLSFDHMPGKAPGEQHHHHHHAAPPMLYAAPPQGAGAPGGSVLGMGELMRKKRGRPRKYAPDGSMALALAPISSASAGGGGGAAAPGQQQQHGGFSIGSPPSDPSAKRRGRPPGSGKKKQFEALGSWGIAFTPHILAVKAGEDVASKIMTFSQQGPRTVCILSANGAISNVTLRQPATSGGLVTYEGRFEIISLSGSFLLAEDGDTRSRTGGLSVALAGSDGRVLGGCVAGMLMAATPVQVVVASFIAEGKKSKPAEARKVEPMAAPPPPPPQMAAFVPAPVATSPPSEGTSSASSDDSGSPINHGAMPFSHSSQHQHPHQHQQHMPPAYASGGWSLSVHQQNRHDSDMKMMSN

>Zm2G062591_T02

MEAKPGEASVGHAPAPMAAAAPVMAAPPLEETVSFQQPLPAPAAAAERGSSSAVLVPPLATAAGGGAMALVPVLMKVPKKRGRPRKYGPDGSLIRPLNATPISASAPLPAAVAPGHYTPASAVGAAMKRGRGRPLDFAAAAAKQHQQHHHQLYQHQQQQFGFHFDSIGDMGACSAGANFTPHIITVAPGEDVMTKVISFSQQGPRAICVLSANGVISTVTLCQPDSSGGTLTYEGRFELLSLSGSFMPTENGGTRSRSGGMSVSLASPDGRVVGGGVAGLLVAASPVQIVVGSFLPSYQMEQKNKKPRVDAAPAAAVPQTPPAVPISSTDTRSSEQGQQSSASQRGVNMTVPGGTYGADQSWVLSPAPQQPMAEVSRTPSSGDLKMTTSGS

>Zm2G123887_T01

MDGRESTVASGPNFSSFYTQHRGIGAPGVPGHSAVLHDPPPAGYRQHLDAVSAGYAFQTPQVGGSHIGQGYHHVEASPHVAQYSSGGGTSSGGDMDIGMGSAVCTNVKGELGSGPAQDEQVKKKRGRPRKYKPDGAVTLGLSPSSSLTPHSASLGMGTMISAPGSGFGSEGSGASGLGAPSEKRGRGRPPGSGKMQQLASLGKWFLGSVGTGFTPHVIIIQPGEDVAARIMAFSQQGPRAVCIISATGAVSAATLHQDSESGSVVTYEGRFEILCLSGSYLVVDEGGGARTRSGGLCIALCGPDNRVIGGSVGGVLMAAGAVQVIVGSFMYGGGSKKNKVKAELDAEPEEANAGDQEVALAEHSSMAPHPAMSGGGGWASGMMRQMESRTPNIDINSIRE

>Zm2G073199_T04

MEVRSEQGLMAGRDLFGLTKSPPAPAPPSSAAMQSVRMAYTADGTAVFAPVSSSPATPSYQPQGAAHGASMSAATVVGGNGAPAAPSMGEPLAKKKRGRPRKYGPDGSMALAMVPASAASGSPATGQGFSGPFSPPALNPASSLVVASPDGFKKRGRPKGSTNKPRVDAAGSSGAGFTPHVITVQAGEDVASKIMSFSQHGTHGVCVLSANGSISNVTLRQTATSGRTVTYEGQFEILSLSGSFFLAEDGVQRSRNGSLSVSLAGPDGRLLGGGVAGLLVAASPVQIVLGSFNSGGGKEPQKQAPSEPTSAPPRVAPTAGMGGPSSPSSRGTLSESSGGAGSPPPLHRAMAASASNSNQPPFLSSMPWR

>Zm2G086876_T01

MDAKELISQSDLQSFYQQQQQQHRAFGGHHSPSSLAGMHSVIRPMPNMNMSPTAILNSISGGSLAGMQFQMDPPPPLLHNINATGAVTPAPAEPLKRKRGRPRKYGPDGTMRQQQQQQAASSQQQLVATQPRICSLSSGPDMLGSSGMEDPAQKKRRGRPPGTGKKHQPSTSQGPGNAFAGSAGTSFTPHIITASPSEDVAAKIVAFASQSSKAVCVLSAMGSVSRAVLRHPADGSPMARVHASPQPYKNPAVYEGFYEILSLTGSYNLAQGGGLSVTLCSPERNVIGGVLGGPLVAAGTVQVVLGSFHQGGSRSKSKKAGKQQQAAAFSPDSLTGGQEASPSSGHNQNLTPPPSVTGGWPTSGIFDTRSSSIDINSSRG

>Zm2G159926_T02

MMEVSPSPEQGVMAGREPFGLPKTPATPPSSGGTQGLRMAYTTDGTAIFTPVSSVPPATATYQPVGGSAASASSLAGVGGNGGAPVHSGGAGEPGTKKKRGRPRKYGPDGSMSLALVPASMAGEPAPALGASGPFSPNGPKAPNTAPSASPDGAKKRGRPKGSTNKKHVAALGPAGAGFTPHLIFVKAGEDVSAKIMSFSQHGTRAVCILSANGAISNVTLRQSATSGGTVTYEGRFEILSLSGSFLLSENGGQSRTGGLSVSLAGPDGRVLGGCVAGLLTAASPVQIVVGSFDAGGKKQPKQQQQQQLAPSPAPLNLAPTGVAAGPSSPPSRGTLSLSESSSGAPPSPPHAGASGGHGQQQQQPQPGGFSGLFWK

>Zm2G124638_T06

MSSSPRNGWWSAMEVRSEQGLMAGRDLFGMPKSPPAPVSGPPASAAMQSVRMAYTSDGTAVFAPMRSSAATPSYQPQGAAHGASMSAATIIGGNGAAAAPSMGEPVPKKKRGRPRKYGPDGSMALALPVSAATGSPTTGQGSSGPFSPAGSNLTNSLLVASPDGFKKRGRPKGSTNKPRMDAAGSSGAGFTPHVITVQAGEDVSSKIMSFSQHGPRAVCVLSANGAISNVTLRQTATSGGTVTYEGRFEILSLSSFLLVEDGGQRSRTGGLSVSLAGPDGRVLGGGVAGLLVAASPVQIVLGSFNSGGKKEAKKHAPSGPTPAPLKVAPPTTTRMGPNSSSPPSRGGTLSESSGGAGSPPPPLHQGMAASSSNDQPPFLSSMPWK

s

>Zm2G074107_T01

MDDKELISQSDLQSFYQQQQQHHRAFGGGGGGQHSPSSLAGMHSVIRPMPNMNMSPSAILNSIGGGSFTGMQFQMDPPPPPLLHTAAMGASAPTSTPGAVPAAPTEPVKRKRGRPRKYGPDGTMKQQQLVAAQPRIGPSGPNMISSAGIEDSSQKKRRGRPPGTAKKHQPSPSQGNAFAGSAGTSFTPHIITASPSEDVAAKIVAFATQSSRAVCVLSAMGSVSRAVLRHPADGSPMARVHASPQPYNNSPAIYEGFYEIMSLTGSYNLAEGSQQEQCQGQGQPSGGLSVTLCSPERNVIGGVLGGPLVAAGTVQVVLGSFHQGGSKSKSKKGGKQQQAPAPAAAFSSDSLTGGQEASPSSGHNQNLTPPPPPPSVTGGWPTSGMFDTSIDINSSRG

>Zm2G164489_T02

MDGRESTVASGPNFSSFFAQHRGIGAPGVPGHSQGLHAPPPGGYRQHLDAVSAGYAFQTPHVGGPSIGQGYEASPHAAQHSAGGGSGSGGCGGMDIGMGAAVSADVKGDQGSGPGQDEQVKKKRGRPRKYKPDGSVTLGLSPTSSSTPHSSSSGMGTMVNTPGSGFGSGGSGGSGSGAPSEKRGRGRPPGSGKMQQLASLGKWFLGSVGTGFTPHVIIIQPGEDVAARIMAFSQQGPRAVCIISATGAISTATLHQDSDSGGVVTYEGRFEILCLSGSYLVVEDGGTRTRSGGLCIALCGPDHRVIGGSVGGVLTAAGTVQVIVGSFMYGGSKKNKVKAEVDMEPEEVAPAEHSGMVPPAMSGGGWEAGMMRQMDSRTPAIDINSIRG

>Zm2G089562_T01

MSAMAAAGEAEYGECVQKGSLQQHQAPQPSPGALFYTHDGVAVYRNPVMPAFYQQPAGSNVVVPAAPGPAHSPASSEPFKRKRGRPRKYAPADGAVPLAIVPPSQPPTARAPATSEASPTVPPGFSPSPQSGGVVSRQASPAPAPASGAPDVKKRGRPSGPSSKKQQPQAAAPGPGWTGLKPHIFTVQAGEDVASRAMSFSGNGWAVCILTANGTVSNVTLRQGESSGGTVTYEGRFEILSLAGSYLLSESTGMSSRTGGLSVSLASPDGHVLGGAVAGPLTAASPVQVVIGSFLADTKMELDPGSAPEKHVFSRFQTTSSPSRGTESSGGHASPPNTTGSFSTSTQPGFPSFPTWK

>Bra022946

MNMPVEMTGSEPVKKRRGRPRKYGPELGLVPGAPSFTQAQTSGGGSGEGGSSAQKRMRGRPRGSSNRKKLQALGSTGVGFVPHVLTVGTGEDVSSKIMAFSQNGPRTVCVLSANGSISNVTLRQFATSGGTVTYEGRFEILSLSGSFLLVENNGHRSRTGGLSVTLSAPDGHVLGGCVAGLLIAASPVQIVVGSFIPEGQKELASPTLLPRVAPSHVLMNPSSPQSRGAMSESSIGGHGSPLHQSNTGGPYNNTNNPSMPWK

>Bra021865

MSGSETGLTAANREPMPFTMSLHHQQHNQPPPPPQPQQNSQNMQLSFTGADRTAVYKPMSSDSSPPQQYQHNSVTGLNMNVPVMGGEQRVKKRRGRPRKYEPGSGGASMGFVPGPPSYTVSQPSGGDGGGGGASPTVKRMRGRPSGSSNRPKLQALGSTGVGFTPHVLTVNTGEDVSSKIMAFSQNGPRAVCVLSANGAISNVTLRQAATSGGTVTYEGRFEILSLSGSFLLLENNGHRSRTGGLSVSLSAPDGNVLGGCVAGLLIAATPVQIVVGSFIPDGQKEHVGQMELASPALPRVAPSHVLTTPNSQQARGGMSESSCGGHGSPLHQSAGGPYNNNSNNLSMSWK

>Bra013270

MDSRELHHQHQQQLQQQQQLQPPPGMLMGSFNRNPNASLMGPTSTSQAMMHHHHRSSSLPFGSLSPHHLQMDQKTLESLGFEGSPSTHQQSMRFGIEQQQQVKKKRGRPRKYTPDANNIALALAPTSPLPSASNSYGGGNDGAGDSGGCGGANSTDPPAKRNRGRPPGSGKKQLDALGGTGGVGFTPHVIEVKTGEDIAMKVVAFTHQGPRAICILSATGAVSSVMFRQSSNPNGVVKYEGPYEIISMSGSFLNTESNGTVTKTGSLSVSLARPDGQVVGGCVAGMLVAGSQVQVVVGSFVPEVKKPKQSAGRVQNTPEPASAPANMLSFGGGGGQEALGLRDSSNIRASHQRKTKVTLRCTVVATTITTTIIMDYLETLRRNHFTKCLCSRCTILTSGLATILNKQSWFIAMLRLHISPSELSTS

>Bra021027

MGSYNRNPNAAAAAAALMGPTSTSQAMHHRLPFGSLAPHQPQHHQQQQQQLHPHQHHQPQPQHQMDQKTLESLGFEGSPSSVAAQQQQQPMRFGIEPQAKKKRGRPRKYAADGNIGLALAPTSPASNSYGGGAEGGGGGGGGDSGGGGNANSSDLPAKRNRGRPPGSGKKQLDALGGTGGVGFTPHVIEVKTGEDIAMKVVAFTQQGPRAICILSATGAVSTVMLRQANNPNGAVKFEGPYEIISMSGSFLNTESNGTVTKTGSLSVSLARPDGQVVGGCVAGMLVAGSQVQVVVGSFVADGKKQKQSAGRVQNTPEPASAPANMLTFGGSGGGGGGGGGGQGSPRSQGQQHSSESSEENESNSPLHRGNNNNNNHHGLFGNSTPQQLHQMPPMQQQMYHHHLWPGHNPQ

>Bra025511

MLVKKKRGRPRKYVADNNEGCDLELSPMQSLQKPNISSPVSDPTAPKRARGRPPGTGRKQRLANLGEWMNTSAGFAFATHVISVEAGEDIVSKVLSFSQQRPRALCIMSGTGTASAFTLRQTGSSAPTLSFQGHFDILSVQGCYLVNEEGGSKSRTGGISVSLSRHDGFLIGGTVGTLIAASLVQVVACSFVYGSAKAKVIKQESGSKEDNTTKKENSMETPASEQRSPRATESAAEAAQTPLDYSSPGWAGPGGGGSRTTDSRNNNHLTDIDLTRG

>Bra014444

MDRREAMGSYFIQRGMPGPGPPPPPSQTQQPFQGSQGFHHFSNPNYQTQGGGSTGFVSPPLQMESSPVDSSAVAPPPPGETSLKRKRGRPRKYGQDGSVSLALSPSVGSSSMSPNSNKRGRGRPPGSGKKQRLASIGDLMPSSSGMSFTPHVIVVSVGEDIASKVLSFSQQGPRAICVLSVIGAVSTATLIQPAPSHGAITYEGRFELVSLSLSYLNSNDNDYSTRTGNLAVSLASSDGRVIGGGIGGPLIAASHVQVIVGSFLWAVPKGKIKKRDEDVQDTDALENNNDNTAAPASPPVPQSLVQTPVGMWSTGSRSMDMHHAHMDIDLMRG

>Bra003446

MDQREAMALSGSGYYYIQRGMPGSAPPQTQPSFYGSQGFQQFSNPSSPFGSTGFVYPPLPVETSQVDSLTPVALPPSGETFVKRKRGRPRKYGQDGSVSLALSPSLSSSMSPNSNKRGRGRPPGSGKKQRLSSTGNKPLFLQLYVKGSVNGGMRLVYNPHSCLVCQDIASKVMSFSQQSPRAICVLSVTGAVSTATILQRSLSHGAIKYEGRFELLSLSTSYPNATDNDYPNSTVNLAVSLACPDYRVIGGGVGGPLIAASSVQVIIGSYIWAIPKGKIKKRDEDVQETDALDDNTAATSPDVPQQSHNLVQTPVGMWSTGSRSMDMHHAHMDIDLMRG

>Bra025717

MDSNETHQQQQNRNAADALAGPTAPSQAMHNRSSVGALSLRKPQPLQGVMLHGSPYSASIEKRGRGRPRKYAPPDVNVNVNVNDGGGANAGPPAKRRGRPLGSRTKQPRKASGGGGGPLTAHVINVNTGEDIAMKVVAFVNQEPRDVCILSVSGAVSSAVIQSHNPFGLVKLEGLYVITHMSATFSNTESNGGTVTRTGNLKVSLAGPDFAVLGGFVGGMLVAGSPVQVIVGTFVREGVKLSTDSASAHVLNSDGGGGPGLPQSQGPSESNASKSLGNSTPQPPHHLPPQQSSISKWFMGPSH

>Cpa26.15

MAGSETGVMTSREPFGVGLQMSPVPSQPVMQNMRLAFSSDGTAVYKSMNATSPSYQPNSTGATPVGVVEGGSTGGHAMTPGLNMNMGSEPMKRKRGRPRKYGPDGAMGLALMPGQQSVAGTQSIGAGGFSSPPLTASASLPSGASVSPTSLKKARGRPPGSTKKQQLEALGPAGVAFTPHVITVKAGEDVSSKVMSFSQHGPRAVCILTANGAISNVTLRQPATSGGTVTYEGRFEILSLTGSFLLTENGGQRSRTGGLSVMLSGPDGRVLGGCVAGLLTAASPVQVVVGSFLADGNKDLKQSGNQMDPLSAPPKLVPVGGPAGPSSPPSRGTLCESSGGAGSPLNQSTGACNNNNPQSMVTMPWK

>Mdp477220

MSGSETGVMTSREPFLQKSPLQSQSAIQSMRLNFSPDGGSALYKPVAAATSPAYQSSAAASGGGAVMPGGAGEGAVMAPAAAAAAAGLNMNMGTEPMKKKRGRPRKYGPXGTMALALSPSAPPXTVTQPSGGAFSPPPLPPAPAPPSGGGSASPPPTSTSTKKSRGRPPGSXKKQQLDALGSPGFGFTPHVITVKAGEDVWSKIMSFSQNGPRAVCILSATGAISNVTLRQPATSGGTVTYEGRFEILSLSGTFLLSEIGGQRSRTGGLSVSLSGPDGRVLGGGVAGLLIAACPVQVVVGSFAADGRKESKTANEMEPSSVAPKFDPGSGPTGASSSQSRGTLSESSGGPGSPLNQSTGTCNNNNPQGMSSMPWK

>Mdp155558

MSGSETGVMTSREPFLQKSPLQSQSAIQSMRLNFSPDGGSALYKPVAAATSPAYQSSAAASGGGAVMPGGAGEGAVMAPAAAAAAAGLNMNMGTEPMKKKRGRPRKYGPXGTMALALSPSAPPXTVTQPSGGAFSPPPLPPAPAPPSGGGSASPPPTSTSTKKSRGRPPGSXKKQQLDALGSPGFGFTPHVITVKAGEDVWSKIMSFSQNGPRAVCILSATGAISNVTLRQPATSGGTVTYEGRFEILSLSGTFLLSEIGGQRSRTGGLSVSLSGPDGRVLGGGVAGLLIAACPVQVVVGSFAADGRKESKTANEMEPSSVAPKFDPGSGPNRGK

>Mdp133746

MDRRDPMALSGSASYFTSRGITGSGTLSGLHGSPGIHPLSNPNIAFQSNIGGTNIGSTLPVEPSSAISYHGVNVGAPTVAPPGESFKRKRGRPRKYGPDGTVSLALSPAASANPGTVSSSPKRGRGRPPGSGKKQQLASLGGLLSGSAGIGFTPHIISIAVGEDVATKIMAFSQQGPRAVCVLSANGAVSTVTLRQPSTSGGTVTYEGRFEIICLSGSYLLNEIGSSRNXSGGLSVSLASPDGXVIGGGVGGMLIAATPVQVILGSFTWGGLKTKNKKKEAVEGVTDLEHETVDNSVALNSIQPDQSLSQSASLAAWXASQPLDMHNSHVDIDLMRG

>Mdp231744

MDRRDPMALSGSASYFTSRGITGSGTLSGLHGSPGIHPLSNPNIAFQSNIGGTNIGSTLPVEPSSAISYHGVNVGAPTVAPPGESXKRKRGRPRKYGPDGTVSLALSPAASANPGTVSSSPKRGRGRPPGSGKKQQLASLGGLLSGSAGIGFTPHIISIAVGEDVATKIMAFSQQGPRAVCILSANGAVSTVTLRQPSTSGGTVTYEGRFEIICLSGSYLLNEIGSSRNXSGGLSVSLASPDGXVIGGGVGGMLIAATPVQVILGSFTWGGLKTKNKKKEAVEGVTDLEHETVDNSVALNSIQPDQSLSQSASLAAWXASQPLDMHNSHVDIDLMRG

>Mdp598703

MDRRDPMALSGSASYFTSRGITGSGTQSGIHGSPGIHPLSNTNIAFQSNIEGSNIGSTLPVGPSSAISQHGVNLGAPSAVPPGESXKRKRGRPRKYGPDGTVSLALSPAAXANPGTVSSSPKRGRGRPPGSGKKQQLVSHGGLLSGSAGMGFTPHIISIAVGEDVATKIMAFSQQGPRAVCILSANGAVSTVTLRQPSTSGGTVTYEGRFEIICLSGSYLLTEIGGSRNRNGGLSVSLASPDGRVIGGGVGGMLIAATPVQVIVGSFXWGGSKTKNKKREAVEGVTDLEHETVDNSXALNSIQPDQSVSQSASLAAWQASRPLDMRNSHVDIDLMRG

>Mdp131485

MDRRDPMALSGSASYFTSRGITGSGTQSGIHGSPGIHPLSNTNIAFQSNIEGSNIGSTLPVGPSSAISQHGVNLGAPSAVPPGESXKRKRGRPRKYGPDGTVSLALSPAAXANPGTVSSSPKRGRGRPPGSGKKQQLVSHGGLLSGSAGXGFTPHIISIAVGEDVATKIMAFSQQGPRAVCILSANGAVSTVTLRQPSTSGGTVTYEGRFEIICLSGSYLLTEIGGSRNRNGGLSVSLASPDGRVIGGGVGGMLIAATPVQVIVGSFLWGGSKTKNKKREAVEGVTDLEHETVDNSIALNSIQPDQSVSQ

>Mdp546289

MSGSETGVMTSREPFLQKNPIQSQSAIQSMRLNFSADGGSALYKSVATATSPAYQSSVTSAAASGGGAVVPGAAGEGAVMAPAATAGLNMNMGTEPMKKKRGRPRKYGPDGTMALALSPSVPPVTVSSSSVGAFSPAPAPPSGGGSASPPPTSTSTKKSRGRPPGSSKKQQLDALGAPGFGFTPHVITVKAGEDVWSKIMSFSQNGPRAVCILSATGAISNVTLRQPATSGGTVTYEVCEIAGCLVCWPFLGRFEILSLSGSFLLSEIGGQRSRTGGLSVSLSGPDGRVLGGGVAGLLTAACPVQVVVGSFVADGRKESKTANQMDPLSVAPKFDPGSGPTGANSPQSRGTLSESSGGPGSPLNQSTGACNNNNLQGMSSMPWK

>Mdp230111

MDSRDVPQQQPPQPPQQPNMMVGLPSYPTSIPTAGINPNSGSMMGGPNPGRFPFNAVAQQQQQPTSKPQMDSLSPSPYDGTLRPCGSGGGFNIDSSSASAAKKKRGRPRKYSPDGNIALGLTSTQIPSXASAAAGTHGESSGTMSSDPPAKKNRGRPPGSGKKQLDALGACGVGFTPHVIMVQAGEEEEKSKGSSATGADKGSNERIVTIIILNLVQLASTGDIAAKVMAFSQQGPRTVCILSANGAICNVTLRQPAMSGGTVTYEGRYEIISLSGSYLFSENNGNRSRSGGLSVSLAGSDGQVLGGGVAGMLMAASPVQVIVGSFIADGKKSNSNLVKSGTSSPPASQMLNFGAPMTAASPSSQGGGSSESSDENGSSPLNNSNRGPVLYSNANQPIHNMQMYQLWGQAQQ

>Ppa08388m

MDRRDPMALSGSASYFTSRGLTQSGLHGSQGIHPLSNPNTAFQSNLGGGNIGSALPIEPSSGITPHGVNVGVPSMLPPGEPVKRKRGRPRKYGPDGTVSLALSPSSSANPGMVTSTPKRGRGRPPGSGKKQQLASLGELLSGSAGMGFTPHIITIAMGEDIATKIMSFSQQGPRALCILSANGAVSTVTLRQPSTSGGTVTYEGRFEIICLSGSYLLTESGGSRNRTGGLSVSLASPDGRVIGGGVGGMLIAASPVQVIVGSFIWGSSKTKSKKREAVEGATDLDHQTVDNSVALNSISQDQSLSQSASLAAWQASRPLDIRNTHVDIDLMRG

>Ppa08093m

MDGREAMGLSGGSAQYYIHRGGVGGSMPGSQAGGGLHTPPGFRHMSNTVLQPQSNVRVSSVGSTFSVEPSRPNFPHHGISMNVTPGVPSGEPVKKKRGRPRKYGPDGPVSLGLSPMSATPNPRPGSTSPTPKRSRGRPPGSGRKQQLATLGDWMNTSAGLAFAPHVITIGAGEDIAAKLLLFSQQRPRALCILSGSGTASSVTLRQPASTGVSVTFEGRFQILCLSGSYLVAEDGGPRNRTGGISVSLSSPDGHVIGGAVAMLIAATPVQVVLCSFVYGGSKTKNKQVAGPNSDENSEPQHNEKLALPSNTPPTQNYNPSGAGIWPGSRQVDLRNPHTGIDLTRG

>Ppa07231m

MDSREVPQQQQPPPPPQQQSMMVGPPSYQTSMPNSNLNPNSGPMMGGPNPARFPFNAVPQPQQQQQQPTSKPQMDSLSPSPYDGSLRPCGSGGGFSIDSSSASAAKKKRGRPRKYSPDGNIALGLAPTQMPSTASTAAAGPHGESSGTMSSDPPAKKNRGRPPGSGKKQLDALGAGGVGFTPHVIMVQAGEDIAAKVMSFSQQGPRTVCILSANGAICNVTLRQPAMSGGTVTYEGRFEIISLSGSYLFSENNGNRSRSGGLSVSLAGSDGQVLGGGVAGMLVAASPVQVIVGSFIADGKKSNSNFLKSGPSSPPPSQMLNFGAPMTAASPSSQGASSESSDENGSSPLNRGPVLYNNASQPIHNMQMYQLWGQAQQ

>Ppa07321m

MSGSETGVMTSREPFSVGGLQKSPLQSQAAIQNMRLNFSPDGSAAAALYKPVAAATSPTYQSSAAAGGSAPVPLAAGEGSPGAAVMAPAPAAAGLNMNMGSEPMKRKRGRPRKYGPDGTMALSLSPSAASVTVTQSSGGAFSPPPPHPPPPSVGSASPTSIKKARGRPPGSTKKQQLDALGSVGFGFSPHVITVKAGEDVSAKIMSFSQNGPRAVCILSANGAISNVTLRQPATSGGTVTYEGRFEILTLSGSFLLSESSGQRSRTGGLSVSLSGPDGRVLGGGVAGLLTAASPVQVVVGSFVADGRKEPKTTNQLEPVAPKLAPSSGPTGASSPQSRGTLSESSGGPGSPLNQSTGGCNNSNPQGMSSMPWK

>Ppa26997m

MEENNMVTTSQPPQTESKPNNEVETFMEPVQMNIEPNQDLDQSGAPIGRVELTGTDVLVKRKRGRPRKYEMVGEEGNVVGLVSASPSSTYSGSYSESLPKRGRGRPKGSGKLQLLSPRGGLSVDPAGGGFYTQVLTAETGEDIVHKILSLSETNPRSLCILTATGVVCSAVIRQPSSYTGILRFKGRFQILTLSGSFVYDATQNRRGKNGMLSVALCHPDGNIFGGAVAGALIAAEPVQKWNCQCCSKDSFNSNTSKELKRRHSAESSTSTSMLGNSSCLAMVPLLMPPPTIVHDESCISPTSALLEFPSHSGAGNVIAANRNMNPATLPGFDQNALQPMPDPTTSPHIDTFIP

>Sm410086

MALVPQQLGGGGGGGGGGGSGGGSGGVMGGGVVGSVRPGEPVKRKRGRPRKYGDGASGSSSVSLALTPLSSVSPISSVTTTPTEKRRGRPPGSGKKQQLAALGSAGQGFTPHVITIAAGEDVATKIMSFSQTGPRAVCVLSANGAISNVTLRQPATSGGTVTYEGRFEILSLSGSFLLTESGGTRSRTGGLSVSLAGPDGRVVGGGVAGLLMAATPVQVVVGSFIADTRKSVPLRAMDSPQGASASGSMQRPTKNESGARSPDSPHMEQQSSGAGGGPGGGAGGGAGGGGGGGGGGGGGSLLQHSQHSQHSVQGMGSLGGWSSSQTLAEPRRDTDINIPLPNG

>Mes022768m

MIGIQEKCQERESYGGGGERNQSPTITPTSNMNLAFRADGVTDVYKPIMPITNDDTLHHVQSIPPHHHEMMLMGCGGIGIGVGGDGLDMNTAEPIKRKRGRPRKYSPPHGNVNLNLTSPLSHHHQHHEPHQSPLLHSGFQSPSSPSSTSKKARGRPPGSGRKNQLTLGSGVGFAPHVITVKAGEDVLLKIMSFSQNGPRGVCILSANGAVSNVTLHQPATSGGTVTYEGRFEILSLSGSFLPSESSGQRGRTGGLSVLLAGPDGRVLGGVAVLLTAASSVQVIVGSFISEDWKESSLGINQPETLYAPGASIAGSPTSRGTFSESSVGLGSPPNHSTGGCNNSTLLGMPNVPWK

>Mes011562m

MSMINPNIPPPAAAGFPFNSVCPPRPQSKPPSSDGLFDGSSPPSSTGMRFSMEPAKKKRGRPRKYTPDGNIALGLSPTPISSSPNSLVHADSGGGTGTPGVASEPSSKRNRGRPPGSGKKQLDALGGVGGVGFTPHVITVKAGEDIASKIMAFSQQGPRTVCILSANGAICNVTLRQPAMSGGTVTYEGRFEIISLSGSFLLSENDGSRNRTSALSVSLAGSDGRVLGGGVAGVLTAASPVQVIVGSFIADGKKSNSTTSKSGPSSAPTTQMLHFGAPLTTSSPTHGPSSESSDDNGSSPSPLNRDHGIYNNASQPIHGMNMYQLWASHNPH

>Mes010930m

MDGREAMALASGSAPYFIHRGGGIVGSGSGSQIGPIHVPPGFRPLSNPNLAAQSNARPGSSGPAFSMEPSNANFAHGINIAVPSGVPVGEPVKKKRGRPRKYAPDGQVSLGLSPMPVKSKPPSGPDPLTPRRGRGRPPGSGRKQQLALLGDWMNASAGVAFSPHVICIGSGEDIVAKLLSFAQQRPRAVCILSGTGTVSSVTLRQPASSGPTVTFEGRFEILCLSGSFLVAEDGGPRNRTGGISASLSSPDGHVIGGAIARLIAAGPVQVVVCSFVHGGSKNKDKQVGRLKLNKDSTSPPGDKSATPKSAIPINQPQNFTPSPMNIWPVSRSVDLRNPHTDIDLTRG

>Mes009338m

MEEKESTVSGSPGDSGTDSPPPVSSVVVPQVMNINMNMAAENMVMERSVIPSATTTPPTTGSDGGSGGPVSGSVDLFGKKKRGRPRKYDSDGNLRMQPFYHQPVAGGALTSPPGFSFSPSSPPPSDGLLYSSSKRGRGRPPGSGNWQLLASLGELFANTAGGDFTPHVVTVNTGEDVAGKILSFGQKGPRGICVLSANGAVSNVTIRQPGSSGGILTYEGRFEILSLSGSFTVTETGGVRSRTGGLSVSLASPDGRVIGGGIAGLLLAASPIQIVVGSFMPNGYKTHKRKHHRENASSSVISGAQGVVTEANPISQSKPADGETCLISESPLTEHSHGGTDNNGSEQQMPNATISFTPFWNGSTPASNQRPSPDINLSVPSEEDAQGIVL

>Mes021769m

MEQKESTVSGSPGDSETNSPPVSRAEAPQVMNINMNMAAEDMSVEGSFVAATTIPPALTSDGGSGNQASSSLDLFGNKKKRGRPRKYDSDGNLRVQPFHHPHHQAGPGGALTLQPGFSFSPSSPPSDGLKSPLKRGRGRPPGSGNWQLLASLGELYANTAGGDFTPHVITVNTGEDVAGKILSLAHKGPRGICILSANGAVSNVTIRQPGSSGGVLTYEGRFEILSLSGSFTVSETGGVRRSGGLSVSLAGTDGRVIGGGVAGLLLAASPIQMVVGSFMPNGYKAQKRRNHSDNATGSRTPAAQHVLTEARTIFQPKLEGETCLISSSSLQEERPRGKAHCASDQQIPNATSPLTLSLNGSQHI

>Mes011226m

MDRRDAMAMSGPASFYMQRGMGGSGSGTQSGLNVSSGINALTSSNVSFHSNVGANAIGSTLPIENPTALQPHGANVGAPSVMPPSGEPMKRKRGRPRKYGPDGTVSLALSPPLSTHPGTITPTQKRGRGRPPGTGRKQQLASLGEWLSGSAGMGFTPHIITIAVGEDIATKIMSFSQQGPRAICVLSANGAVSTVTLRQPSSSGGTVTYEGRFEILCLSGSYLVTNNSGARNRAGGLSVSLASPDGRVIGGGVGGMLIAASPVQVIVGSFLWGGLKTKNKKGEAPEGARDLDHQTVEHPITPTSIPPSQNLPPTSSVGLWPGSQPVDMRNTHVDIDLMRG

>Mes008582m

MKALDSSLVFSFLSLYNFRHQLFSILNHWMSGSETGVMTSTARDPFGVGLQKSQVTSQPSIQNMRLAFSADGTAVYKPVTTAASPSYQPTPSGAAGGGAGGVEGSAGGTVVSSHGINVNMNMGTAPETMKKKRGRPRKYGPDGTMALALVPASQSVTQTSGGGFSSPHPPAGTAVTSPLPSGGPVSPTGSKKARGRPPGSSKKQQLEALGSAGVGFTPHVITVKAGEFCHQGLIILTDVSSKIMSFSQHGPRAVCILSANGAISNVTLRQQASSGGTVTYEGRFEILSLSGSFLPSENGGQRSRTGGLSVSLSGPDGRVLGGGVAGLLTAASPVQVVVASFISDGRKESKLANQIEPLSAVTKFAPALGTTGPSSPPSRGTLSESSGGPGSPLNQSTGACNNSNPQGISILPWK

>Mes022700m

MAREAADACMEDKDMISTTSQQSPVTIKHDPQVTGVVDDEEKEPNFGGGSGDSGGGGAIVAGSGGGSVIVEMTGKRKRGRPKKFDMDSEIISPLPSPPPGFPSSLSRTFEKRGRGRPRGSGRLQLLASLGSFAAETAGGSFIPHVVPVNTGEDIVSKISSFAERGSRAICILSATGVVSSVVIRQPGLSGGILRYEGRFEILSLCGSFTFDETSGANGKTGMLSVSLAKPDGRVFGGGIVGSLIASGPIQLIVASFKQNICKELKLRQLAESAAAAGCVLGNSEKVRSSFEIAGTIEGEGHCTSPTSPHSEQTNGTEAFTADPQNIFAQVEPPLDSRNEDL

>Mes010359m

MSEREQYGGGSERNQTPSIAPTSNMNLTFSADGANAVYKPIMPITSDDPFHHVQSISAHHHEMLMLGGGDVSVGVGVGVGVGVGVGVDGLNVNNAAPIKRKRGRPRKYSPPHGHFGLNLTSPLSQLHHNNYHDPHQSPLLHHSEFQSPLSPSSTAKKARGRPPGSGRKNQLAALGSAVVGFVPHVITVKAGEDVLLKIMSFPQNGPRAVCILSASGAISNVTLCQTATSGGTVTYEGRFEILSLSGSFFPSESSGQRSRTGGLSVLLAGPDGRVLGGGVAGLLTAASSVQIIVGSFISEEWKESRPGINQPETMYGPGASIAGSPTSRGTFSESSGGPGSPPNQSTGGCNSNPQGMPNVPWK

>Mes011242m

MDRRDAMALSGSASFYMHRGMAGSGTGTQSGLNVSSGINPLTSSSVPFQSNVGANTIGSTLPIETSTAIPPHGVNVGAPSAMPPSSEPVKRKRGRPRKYGPDGSVSLALSSSLSTHPGTITPTQKRGRGRPPGTGRKQQLASLGEWLSGSAGMGFTPHIITIAVGEDIATKIMSFSQQGPRAICILSANGAVSTVTLRQPSTSGGTVTYEGRFEILCLSGSYLVTNNGGSRNRTGGLSVSLASPDGRVIGGGIGGMLIAASPVQVIVGSFIWGGSKTKNKKGEGPEGARDMDHQAVENPVTPTSVPPGQNLTPTSSVGLWPGSQSLDMRNAHVDIDLMRG

>Mes027591m

MEDKSMTSADSQLSPVAMKNDPQVSQVEHQEAKEPDLGGGGGAAAAAAVAVSGGSSVTVGAGKRKRGRPKKFVMDSGTTSLPVPCPPPPPPPPPPDFTSSLSKTCEKRGRGRPLGSGKLQLLASLGDLAAETAGGNFIPLVARVDPGEDIISLISSFAEMGPRAVCVLSASGVVSKVVIHPPGSDGGVLQYEGLFEILTLSGSFAFDETSGERRKTGVLTVSLAKPNGQVFGGGVVGSLIAYGPIQLILGSFKQNVFNELKLKQLAEKSAVAAGSPLGDSETERSPFPNAGTTEAEGHCTTPTSALLETANDTEAGNTTTDDPENVVKPVEPISDPGNEDC
