# Supplementary material for: Insights into the evolution and diversification of the AT-hook Motif Nuclear Localized gene family in land plants
Source: BMC Plant Biol. 2014 Oct 14;14:266. doi: 10.1186/s12870-014-0266-7 (PMC4209074; doi:10.1186/s12870-014-0266-7)
Supplement: Additional file 5: — Amino acid sequences of wheat TaAHL1, TaAHL3 and soybean Gm06g01650.1. The AT-hook motif is underlined with green. The PPC domain is underlined with blue. [file 12870_2014_266_MOESM5_ESM.pdf]

>TaAHL1/Taq1

atggggagcatggacggccacccgctccagggcaaccacgcctacgcccacgtccctgccggcagcaacaacgacgag
gacgacgcgtcgccgccgccctcggccggcgggggctcctcggggtcgggccgccggccgcgcggcaggcccccgggg
tccaagaacaagcccaagccgcccgtggtggtgacgcgggagagccccaacgcgatgcgctcccacgtgctggagatcg
ccagcggggccgacatcgtggaggccatcgcggccttctcccgccgcaggcagcgcggcgtctccgtcctcagcgggagc
ggcgccgtcaccaacgtcacgctgcgccagcccgcggggaccggggcggccgccgtcgccctaagggggcgcttcgag
atactctccctgtccggcgccttcctcccggcgccggcgccgcccggggcgacggggctcgccgtctacctcgccggcgggc
aggggcaggtggtcgggggcagcgtgatgggggagcttctcgcgtccggccccgtcatggtgatcgcggccacgttcggca
acgccacgtacgagaggctgccgctggatcaagacgccgaggagggcgccgtgctgtccgggtcggagggcgccgcca
cgcagctggagcagcagggcagcggaggcgccgccgtgcccccaccgatgtacgccgtgccgcagacgccgcccggc
gacatgttcgggcagtggggacaggcagccgtggcgcggcctccgccgacgtcattctag

>TaAHL3/Taq3

atggccaccggcagcagcaagtggtggcaagggccaatggacttcccgccgcagccgcagccgcagcagatgcagcag
catcagccgctccagcaccagcaccagccgctgcagctgccggcggtgaccatgccggcgccggctccagcggtggccg
cctcgccggagagcaagcagcagcagcagcagggccagggccagggcgaggggcagatgggggcggcggcggggg
ccatcgtgcagctgcggaggccgggggccggccgatggggtccaagaacaagcccaagccgccgataatcatcacgcg
cgacagccccgacgcgctccactcgcacatcctggaggtggccccgggcgcggacgtcgccgcctgcgtcgccgagtacg
cgcgccgccgcggccggggcgtctgcgtgctgggcgcgtccggctccgtcgtggacgtcgtcgtgcgcggcgcggcctcgg
ccgcgcccctcccggggcgcttcgagctcctctccatgaccggcaccgtgctcccgcccccggcgccgtccgaggcgtccgg
cctcgcagtcatgctctccgccgggcagggccaggtcctcggtggctgcgtcgtggggccgctcgtcgccgccgggcccgtc
accctcttcgccgccaccttcgccaacgccgtctacgagcgcctgcccctccaggacgccgccgacgccgacgtcaagccc
gacctctccgccgcccccgacgcctcggtcccgcaagaagtgcaagcgcagcagccactggcgatctcccaggccatggc
catgggcgcgggctaccccgaccaccgctcaccgcagtacccgtggggaggccaccagggaggcggcatctga

>Gm06g01650.1_Partial

ccccagtccgacgacgacgacggagaaggacccttctccacccagcggcgccctcgcgggcggcccatgggctc
caagaacaagcccaagccacccgtcatagtcacgcgcgacagccccaacgtcctccgctcgcacgttcttgaggtc
tcctccggcgccgacgtggtggagagcctctccaactatgcgcgccgccgagggaggggagtgtccgtgctcagtg
gctccggcacggtggccaacgtcgtgctccgtcaaccggccgggagcgtcctcacgctccacggccggtttgagat
agtttccatgacggggacggtgctcccgcctccggcgccacccggttcggacgggttgtcggtttatctgtcaggggc
gcagggacaggtggttgggggcgtggtggtggcccctctggtggcttcgagtcacgtggttttggtggctgcttccttcg
ctaacgccatgttcgaaaggttacccttgcccttgaatcagcatgatgatgatgatcaaggtgaagtatttggatgggg
tggaactggaactacatcatcaacatcaactgcaccacccaaaactcatcctttcta
