# Supplementary material for: Insights into the evolution and diversification of the AT-hook Motif Nuclear Localized gene family in land plants
Source: BMC Plant Biol. 2014 Oct 14;14:266. doi: 10.1186/s12870-014-0266-7 (PMC4209074; doi:10.1186/s12870-014-0266-7)
Supplement: Additional file 6: — Alignment of two Arabidopsis thaliana AHLs, AtAHL27 and AtAHL29, with soybean Gm06g01650.1. The AT-hook motif is underlined with green. The PPC domain is underlined with blue. [file 12870_2014_266_MOESM6_ESM.pdf]

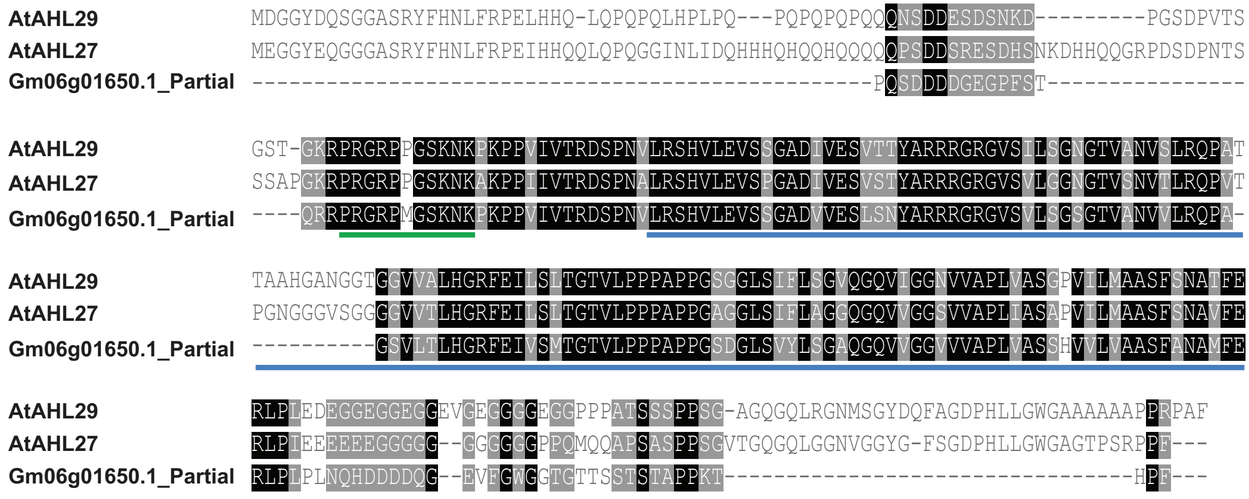
AtAHL29 MDGGYDQSGGASRYFHNLFRPELHHQ-LQPQPQLHPLPQ---PQPQPQPQQQNSDDESDSNKD---------PGSDPVTS
AtAHL27 MEGGYEQGGGASRYFHNLFRPEIHHQQLQPQGGINLIDQHHHQHQQHQQQQPSDDSRESDHSNKDHHQQGRPDSDPNTS
Gm06g01650.1_Partial --PQSDDDDGEGPFST----------------
AtAHL29 GST-GKRPRGRPPGSKNKPKPPVIVTRDSPNVLRSHVLEVSSGADIVESVTTYARRRGRGVSILSGNGTVANVSLRQPAT
AtAHL27 SSAPGKRPRGRPPGSKNKAKPPIIVTRDSPNALRSHVLEVSPGADIVESVSTYARRRGRGVSVLGGNGTVSNVTLRQPVT
Gm06g01650.1_Partial ----QRRPRGRPMGSKNKPKPPVIVTRDSPNVLRSHVLEVSSGADVVESLSNYARRRGRGVSVLSGSGTVANVVLRQPA-
AtAHL29 TAAHGANGGTGGVVALHGRFEILSLTGTVLPPPAPPGSGGLSIFLSGVQGQVIGGNVVAPLVASGPVILMAASFSNATFE
AtAHL27 PGNGGGVSGGGGVVTLHGRFEILSLTGTVLPPPAPPGAGGLSIFLAGGQGQVVGGSVVAPLIASAPVILMAASFSNAVFE
Gm06g01650.1_Partial ----------GSVLTLHGRFEIVSMTGTVLPPPAPPGSDGLSVYLSGAQGQVVGGVVVAPLVASSHVVLVAASFANAMFE
AtAHL29 RLPLEDEGGEGGEGGEVGEGGGEGGPPPATSSSPPSG-AGQGQLRGNMSGYDQFAGDPHLLGWGAAAAAAPPRPAF
AtAHL27 RLPIEEEEEEGGGGG--GGGGGPPQMQQAPSASPPSGVTGQGQLGGNVGGYG-FSGDPHLLGWGAGTPSRPPF---
Gm06g01650.1_Partial RLPLPLNQHDDDDQG--EVFGWGGTGTTSSTSTAPPKT-------------------------------HPF---
