# Supplementary material for: Insights into the evolution and diversification of the AT-hook Motif Nuclear Localized gene family in land plants
Source: BMC Plant Biol. 2014 Oct 14;14:266. doi: 10.1186/s12870-014-0266-7 (PMC4209074; doi:10.1186/s12870-014-0266-7)
Supplement: Additional file 8: — PPC/DUF296 Genes Identified in Selected Picoeukaryotes and Prokaryotes. [file 12870_2014_266_MOESM8_ESM.doc]

| **Additional File 8. *PPC/DUF296* Genes Identified in Selected Picoeukaryotes and Prokaryotes** | | |
| --- | --- | --- |
| **NCBI/GenBank Ref Number**  **Picoeukaryotes** | | |
| *Micromonas pusilla CCMP1545* | | XP_003060030.1 |
| *Ostreococcus tauri* | | XP_003082961.1 |
| *Ostreococcus lucimarinus* | | XP_001422535.1 |
|  | |  |
| **Archaea** | | |
| *Methanosarcina acetivorans* | | AAM05077.1 |
| *Methanopyrus kandleri* | | AAM01827.1 |
| *Methanobacterium thermoautotrophicum* | | AAB85719 |
| *Halobacterium salinarum* | | AAG20444 |
| *Archaeoglobus fulgidus* | | AAB91134 |
| *Aeropyrum pernix* | | BAA80508 |
| *Thermoplasma acidophilum* | | CAC12014 |
| *Pyrococcus horikoshii* | | BAA29895 |
|  | | |
| **Bacteria** |  | |
| *Agrobacterium tumefaciens* | | AAK89291 |
| *Bacteroides thetaiotaomicron* | | AAO76223 |
| *Clostridium tetani* | | AAO035778 |
| *Escherichia coli CFT073* | | AAN81954 |
| *Escherichia coli O157* | | AAG58054 |
| *Rhizobium loti* | | BAB53068 |
| *Staphylococcus aureus* | | BAB56856 |
| *Streptomyces coelicolor* | | CAB62688 |
| *Thermoanaerobacter tengcongensis* | | AAM23579 |
|  | |  |
| ***Volvox carteri*** | | Not Detected |
| **Fungi** | | Not Detected |
| **Hornworts** | | Not Detected |
| **Liverworts** | | Not Detected |
