# Supplementary material for: Insights into the evolution and diversification of the AT-hook Motif Nuclear Localized gene family in land plants
Source: BMC Plant Biol. 2014 Oct 14;14:266. doi: 10.1186/s12870-014-0266-7 (PMC4209074; doi:10.1186/s12870-014-0266-7)
Supplement: Additional file 9: — Intron-mediated transcriptional enhancement of AHL genes in Arabidopsis thaliana . (a) Topology of the exon and intron arrangement of Arabidopsis thaliana AHL genes. (b) The intron-mediated enhancement scores of the first/second/third/fourth intron were shown. The grey line represents a score of 10, which indicates moderate capability of transcriptional enhancement. (c) The intron-mediated enhancement scores of each intron in AtAHLs were listed. The abilities of transcriptional enhancement were categorized with strong (orange color), relatively strong (light orange), moderate (light blue) and weak (no color). [file 12870_2014_266_MOESM9_ESM.pdf]

(a)

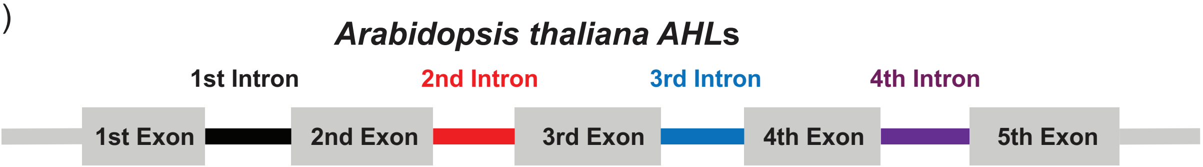

(b)

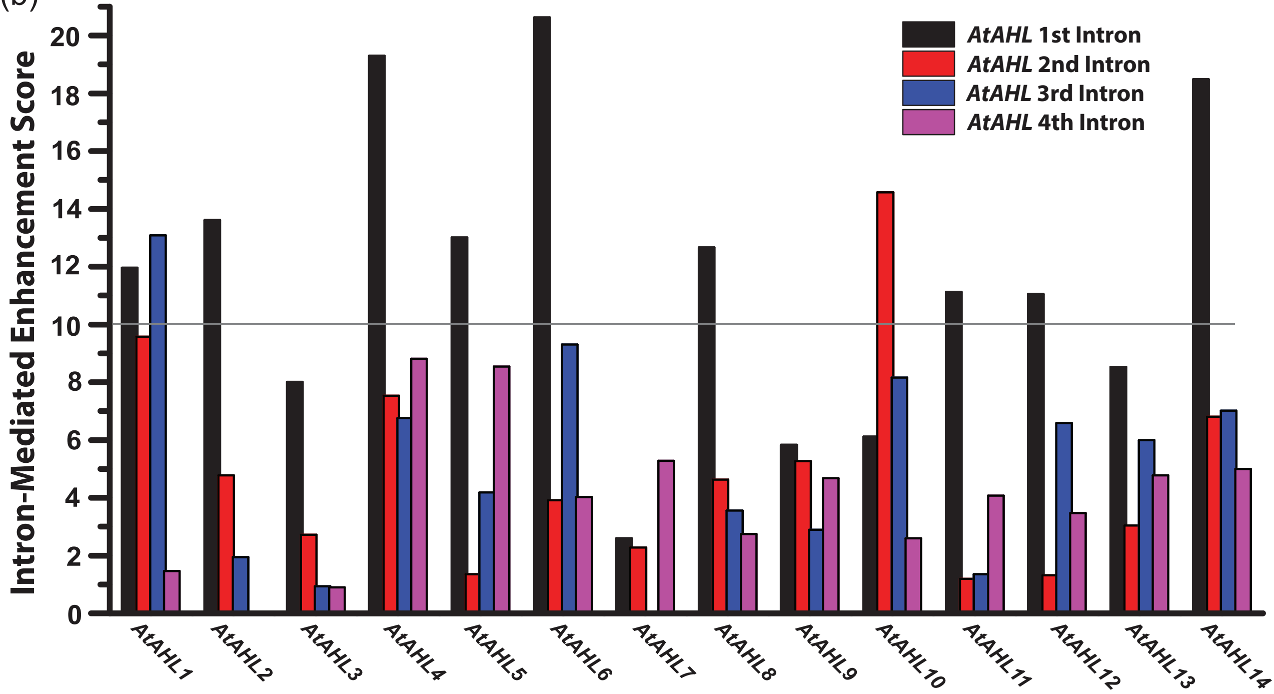

(c)

	Intron-Mediated Enhancement Score			
	1st Intron	2nd Intron	3rd Intron	4th Intron
AtAHL1	11.97	9.58	13.08	1.47
AtAHL2	13.61	4.78	1.94	NA
AtAHL3	8.01	2.72	0.93	0.9
AtAHL4	19.3	7.53	6.76	8.81
AtAHL5	13.01	1.35	4.19	8.54
AtAHL6	20.63	3.91	9.3	4.03
AtAHL7	2.6	2.28	0	5.28
AtAHL8	12.67	4.63	3.56	2.74
AtAHL9	5.84	5.27	2.89	4.68
AtAHL10	6.12	14.58	8.16	2.6
AtAHL11	11.13	1.19	1.36	4.07
AtAHL12	11.06	1.32	6.59	3.47
AtAHL13	8.53	3.04	5.99	4.78
AtAHL14	18.49	6.81	7.02	5
